# Genomic-Wide Identification and Characterization of the Uridine Diphosphate Glycosyltransferase Family in *Eucommia ulmoides* Oliver

**DOI:** 10.3390/plants10091934

**Published:** 2021-09-17

**Authors:** Dan Ouyang, Lan-Chun Wang, Ting Tang, Hong Feng

**Affiliations:** 1 Key Laboratory of Molecular Biology and Biotechnology of Sichuan Province, Department of Biotechnology, College of Life Sciences, Sichuan University, Chengdu 610064, China; ouyangd@stu.scu.edu.cn (D.O.); wanglc@stu.scu.edu.cn (L.-C.W.); tangting@stu.scu.edu.cn (T.T.); 2 Key Laboratory for Bio-Resources and Eco-Environment of Ministry of Education, Department of Biotechnology, Sichuan University, 29 Wangjiang Road, Chengdu 610064, China

**Keywords:** *Eucommia* *ulmoides*, UDP-glycosyltransferase, transcriptome, expression, lignan, flavonoid, biosynthesis

## Abstract

*Eucommia ulmoides* Oliver is a woody plant with great economic and medicinal value. Its dried bark has a long history of use as a traditional medicinal material in East Asia, which led to many glycosides, such as aucubin, geniposide, hyperoside, astragalin, and pinoresinol diglucoside, being recognized as pharmacologically active ingredients. Uridine diphosphate glycosyltransferases (UGTs) catalyze a glycosyl-transferring reaction from the donor molecule uridine-5′-diphosphate-glucose (UDPG) to the substrate, which plays an important role in many biological processes, such as plant growth and development, secondary metabolism, and environmental adaptation. In order to explore the biosynthetic pathways of glycosides in *E. ulmoides*, 91 putative *Eu*UGT genes were identified throughout the complete genome of *E. ulmoides* through function annotation and an UDPGT domain search. Phylogenetic analysis categorized them into 14 groups. We also performed GO annotations on all the *Eu*UGTs to gain insights into their functions in *E. ulmoides*. In addition, transcriptomic analysis indicated that most *Eu*UGTs showed different expression patterns across diverse organs and various growing seasons. By protein–protein interaction predication, a biosynthetic routine of flavonoids and their glycosides was also proposed. Undoubtedly, these results will help in future research into the biosynthetic pathways of glycoside compounds in *E. ulmoides*.

## 1. Introduction

Glycosyltransferases (GT, EC 2.4.x.y) catalyze to transfer a glycosyl moiety from a donor molecule to an acceptor substrate to form a glycosidic bond, which is employed by cells for the biosynthesis of glycolipids, glycoproteins, hormones, and various glycosides. GTs are ubiquitous in all kinds of organisms, from bacteria to animals. To date, 114 families of glycosyltransferases containing more than 850,000 members are recognized and classified in the CAZy database (http://www.cazy.org, accessed on 5 August 2021) [1,2], among which, the largest family (GT1) transfers sugars onto numerous small molecules.

In plants, GTs typically utilize uridine diphosphate (UDP)-activated sugars as donor molecules, by which glycosylation reactions not only synthesize essential substances for growth, but also glycosylate various secondary metabolites, such as monolignols, anthocyanins, and terpenoids [3,4]. Plant UDP-glycosyltransferases (UGTs) have a conserved motif of 44 residues called PSPG box (putative secondary plant glycosyltransferase box) in the *C*-terminal, which is featured as a UDP-sugar binding domain [5]. In light of their important roles in many cellular processes, genomic-wide identification and phylogenetic analyses of UGTs were carried out for many plant species. For example, 123, 147, 179, 180 and 137 UGTs have been identified in *Arabidopsis thaliana* [6], *Zea mays* [7], *Triticum aestivum* [8], *Oryza sativa* [9], and *Linum usitatissimum* [10], respectively. Through phylogenetic analyses, plant UGTs were classified into 18 groups (A to R) [6,11], indicating the significant divergence and expansion of the UGT family during evolution [12]. Moreover, identification of the UGT gene family is also helpful for screening out the UGTs that may be included in specific metabolic pathways. For example, a combination of transcriptome and metabolite analyses of the adventitious roots treated with methyl-jasmonate led to the identification of several putative UGTs involved in ginsenoside biosynthesis in Korean ginseng (*Panax ginseng*) [13]. Through comparative transcriptome analysis between two closely related *Ilex* species, a UGT was recognized as a candidate for participation in triterpenoid biosynthesis [14]. In addition, sequence analysis of the UGT genes in *Camellia sinensis* allowed the identification of two genes encoding flavonol 3-*O*-glucosyltransferase and flavonol 3-*O*-galactosyltransferase [15].

*Eucommia ulmoides* Oliver is a kind of woody plant with high economic and remedial value, since its bark has a long history of use as a traditional medicine in East Asia [16]. In recent years, many studies were carried out on *E. ulmoides*, especially in pharmacology. It was found that the extracted components of *E. ulmoides* perform a wide range of pharmacological activities, such as serving as an anti-hypertension, anti-hyperlipidemia, anti-diabetic, anti-bacterial, anti-inflammation, and antioxidant agent, as well as protecting of the kidneys and liver from injury [17,18,19]. Various compounds with pharmacological potential from various tissues of *E. ulmoides* were subsequently identified, such as chlorogenic acid, geniposide, aucubin, rutin, hyperoside, astragalin, and pinoresinol diglucoside (PDG), etc. [16]. Many of these components are glycosides. For example, geniposide, a kind of bioactive iridoid glycoside, creates anti-inflammatory effects by reducing the release of inflammatory cytokines [20] and can also alleviate obesity-related cardiac injury by activating AMPK-α and the Sirt1 pathway [21]. Another glycoside of aucubin in *E. ulmoides* was shown to have anti-inflammatory effects, which may inhibit pulmonary inflammation and fibrosis in a mouse model established by the intratracheal injection of bleomycin [22]. Moreover, aucubin was also shown to have liver-protecting properties and to suppress DNA replication of HBV in vitro [23]. In addition, the PDG found in *E. ulmoides* is a natural hypotensive compound, which could protect against oxidative low-density lipoprotein-induced dysfunction in vein endothelial cells [24], thus becoming a promising drug for the treatment of cardiovascular disease.

Although many natural glycosides with pharmacological potential have been isolated and identified in *E. ulmoides*, almost none of the *Eu*UGTs have been biochemically characterized up until now. For instance, a *Eu*UGT cDNA was cloned and another *Eu*UGT was recombinantly expressed in *Escherichia coli* [25,26]. However, their catalytic activity and substrate were not definitively confirmed. In order to identify the candidate UGTs involved in the biosynthesis of glycosides in *E. ulmoides*, a genome-wide comprehensive analysis of *Eu*UGT genes should be the first step. The genome-wide identification of a gene family has been successfully applied to numerous gene families in plants. For example, the AT-Hook Motif Nuclear Localized gene family and β-Ketoacyl CoA synthetase gene family were identified in *Glycine max* L. [27] and *Hordeum vulgare* L. [28], respectively, from which the biochemical characteristics, gene structure, evolutionary relationships, and expression patterns of these gene families’ members could be deduced. Furthermore, the assignment of biological functions to each member of the gene family is attractive. Through genome-wide analysis and comparative transcriptomics, the PIN-formed and brassinazole-resistant gene families were identified in wheat, which were recognized as playing crucial roles in response to diverse biotic and abiotic stresses [29,30]. In addition, a genome-wide strategy was also used to characterize long non-coding RNAs and natural antisense transcripts [31,32].

In this work, 91 UGTs were identified through a comprehensive search of the draft genome of *E. ulmoide*s, and then compared with the UGTs of *A. thaliana* and *L. usitatissimum* L. based on a phylogenetic analysis. Further, the expression pattern of *Eu*UGTs was also analyzed using third-party transcriptome data available in a public database. Hopefully, these data can promote future research on UDP-glycosyltransferase in order to elucidate their catalytic mechanism and the metabolic pathways of the glycosides in *E. ulmoides*.

## 2. Results

### 2.1. Genome-Wide Identification of Putative UGTs in E. ulmoides

By blasting the *E. ulmoides* draft genome [33] with the UDPGT domain (PF00201.18), 91 putative *Eu*UGT genes were identified and verified manually (Supplementary file S1 and S2), 81 of which were previously annotated as UGTs [33]. However, the other genes annotated as UDP-glycosyltransferase/glucosyltransferase, glycosyltransferase, or UGT were excluded due to the absence of a PSPG box. All of the putative *Eu*UGTs contained a conserved PSPG motif at their *C*-terminal. The organization of the 91 *Eu*UGT genes was also examined. It was shown that the majority of *Eu*UGT genes (48, 52.7%) had no intron. Among the *Eu*UGT genes containing introns, 37 out of 43 (86.05%) had only one intron. Four *Eu*UGT genes contained two introns and one gene had three introns (Figure S1).

The amino acid similarity between individual pairs of these sequences varied from 21% to 100%. Among them, three pairs of *Eu*UGTs (GWHPAAAL014233 and GWHPAAAL012139; GWHPAAAL022859 and GWHPAAAL018716; GWHPAAAL025228 and GWHPAAAL008406) had 100% amino acid sequence identity and the same gene structure (Table S1), despite being localized in different scaffolds. The other 29 *Eu*UGTs were found to be located in tandem in the same scaffold with a high similarity of up to 73% in the amino acid sequence and almost identical gene structure (Table S1).

The protein sequence lengths and molecular weights of the *Eu*UGTs ranged from 261 (GWHPAAAL023819) to 569 (GWHPAAAL006284) amino acid residues, and 29.1 (GWHPAAAL23819) to 64.3 (GWHPAAAL006284) kDa, respectively. The estimations of theoretical pI ranged from 4.82 (GWHPAAAL014996) to 8.89 (GWHPAAAL005638). Out of the 91 *Eu*UGTs, 17 were predicted as stable proteins, and 74 proteins were predicted as unstable proteins. The grand average hydropathicities of 91 *Eu*UGTs were calculated to suggest that most of the *Eu*UGTs (83) are hydrophilic proteins. The subcellular locations of the 91 *Eu*UGTs were also predicated, with 63 in the cytoplasm, 14 in the chloroplast, 11 on the plasma membrane, 1 in extracellular space, 1 in the lysosome, and 1 in the mitochondria. Detailed information is summarized in Table S2.

### 2.2. Phylogenetic Analysis of EuUGTs

**Figure 1:**
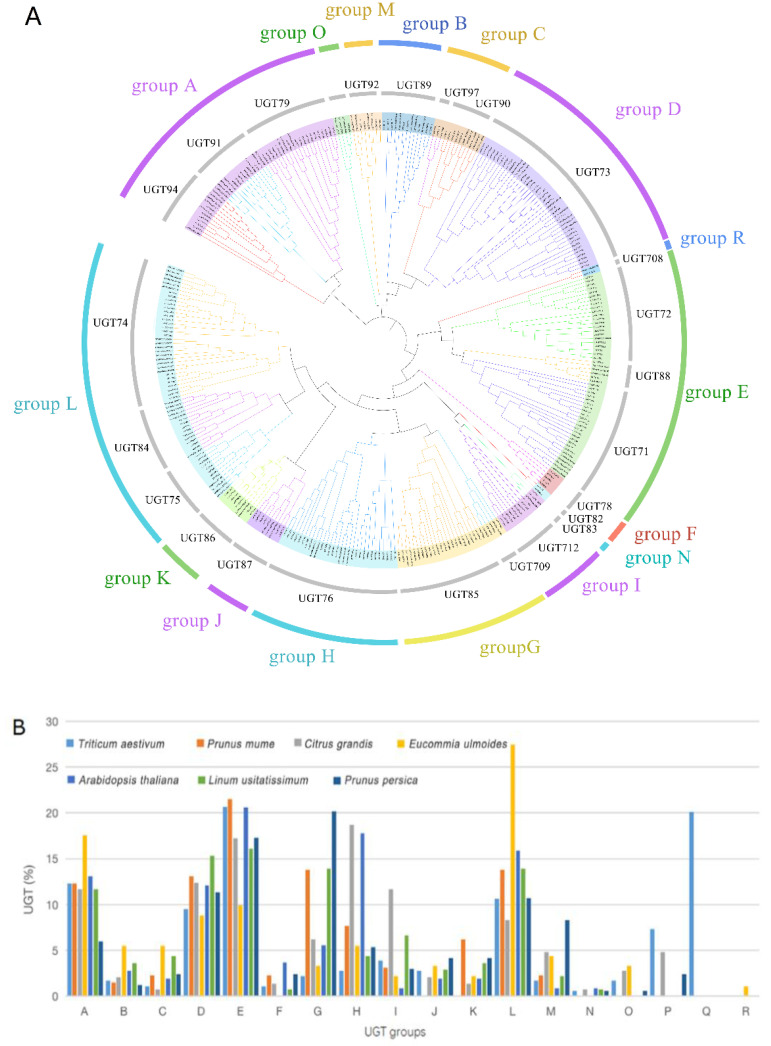
Phylogenetic tree of UGTs in E. ulmoides, L. usitatissimum and A. thaliana constructed by neighbor-joining method (**A**) and the distribution of members in the phylogenetic groups among selected plants species (**B**). In (**A**), each color arc corresponds to a phylogenetic group and each gray arc to a class of plant UGTs according to the UGT nomenclature system [5].

Originally, UGTs of the model plant *A. thaliana* were classified into 14 phylogenetic groups (A~N) based on the homology of their amino acid sequences [6]. Subsequently, four new groups (O, P, Q and R) were found in *P. ginseng* [13], *O. sativa* [9], and *Malus domestica* [34]. Based on this, 91 *Eu*UGTs were later clustered with the selected 246 UGTs (for more detail, see the Materials and Methods section) from other plants to construct a phylogenetic tree (Figure 1A). All of the UGTs mentioned above were divided into 16 groups (A-N, O and R), which are identical to the 18 known groups, excluding groups P and Q. The UGTs of groups P and Q were discovered in the other plants, such as *Punica granatum* L. [11], *T. aestivum* [8], and *Camellia sinensis* [15]. The *Eu*UGTs were categorized into 14 groups, such as A to E, G to M, and O and R. In the set of *Eu*UGTs analyzed, there were no *Eu*UGTs from groups F, N, Q, or P.

The distribution of UGTs in each group were compared among the selected plant species [12] (Figure 1B). Most of the *Eu*UGTs were categorized into the groups A, D, E, and L. The number of *Eu*UGTs in these groups are believed to expand rapidly during the evolution of higher plants [9,12]. In general, the UGTs of group E and L have a wide range of substrates. Group E has been identified in most plants [9], indicating the existence of Group E is conservative during plant evolution. Group E mainly contains flavonoids glycosyltransferases, abscisic acid glycosyltransferases, and monolignol glycosyltransferases. Group L appears only in vascular plants [9]. Substrates of members of Group L contain auxin, anthranilate, anthocyanin, phenylpropanoids, xenbiotics, triterpenes, and coumarins, etc [11]. The enzymes of group A are more specific to the substrates, such as flavonoids, anthocyanidins, and triterpenes. Members of groups B, C, and D are mainly flavonol-7-*O*-glycosyltransferases, benzoic acid glycosyltransferases, and brassinosteroids glycosyltransferases. Most of group F are flavonoid glycosyltransferases [11]. Three *Eu*UGTs were clustered into group O, which are generally associated with the *O*-glycosylation of the hydroxylated isoprenoid side chain of cytokinin [12]. Group O is absent in *A. thaliana* and *L. usitatissimum*, and may have been lost during the evolution of these two species [12]. The members of group R are found in few plants, but *E. ulmoides* has a single UGT occurring in group R, which has been reported to catalyze the *C*-glycosylation and *O*-glycosylation of flavonoids in maize [35] and gallic acid in bamboo [36], respectively.

### 2.3. Analysis of Conserved Motifs and Prediction of Cis-Acting Elements

**Figure 2:**
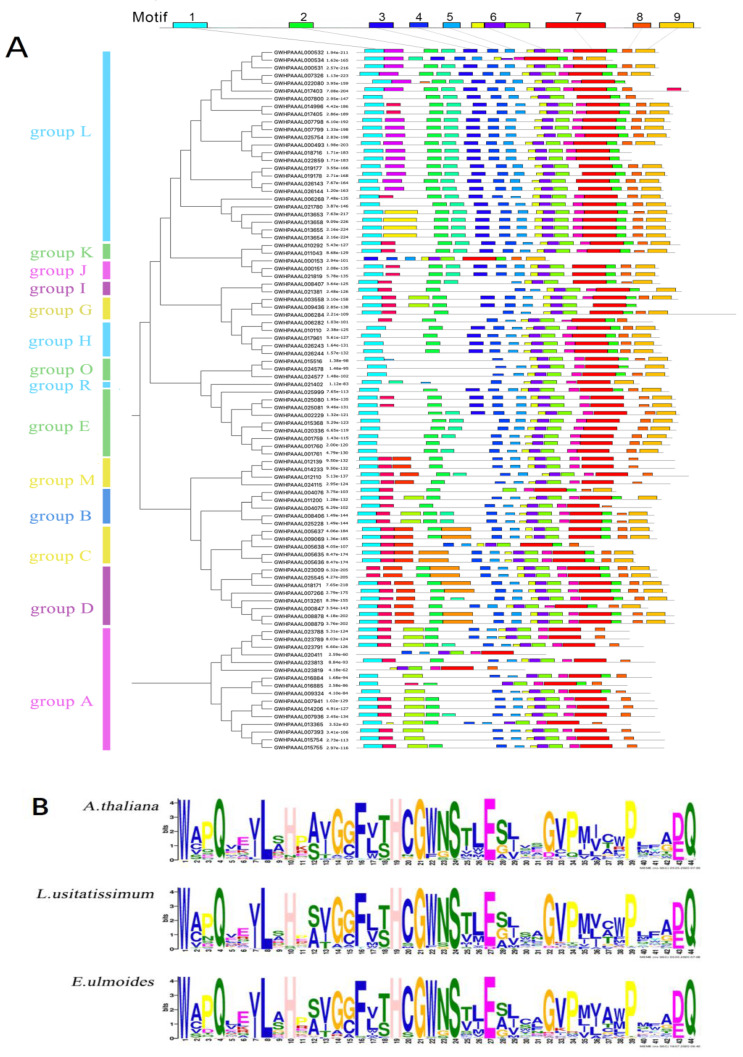
Conservative motifs of *Eu*UGTs (**A**) and the Weblogo of PSPG box (motif 7) within the UGTs in *A. thaliana*, *L. usitatissimum*, and *E. ulmoides* (**B**).

**Figure 3:**
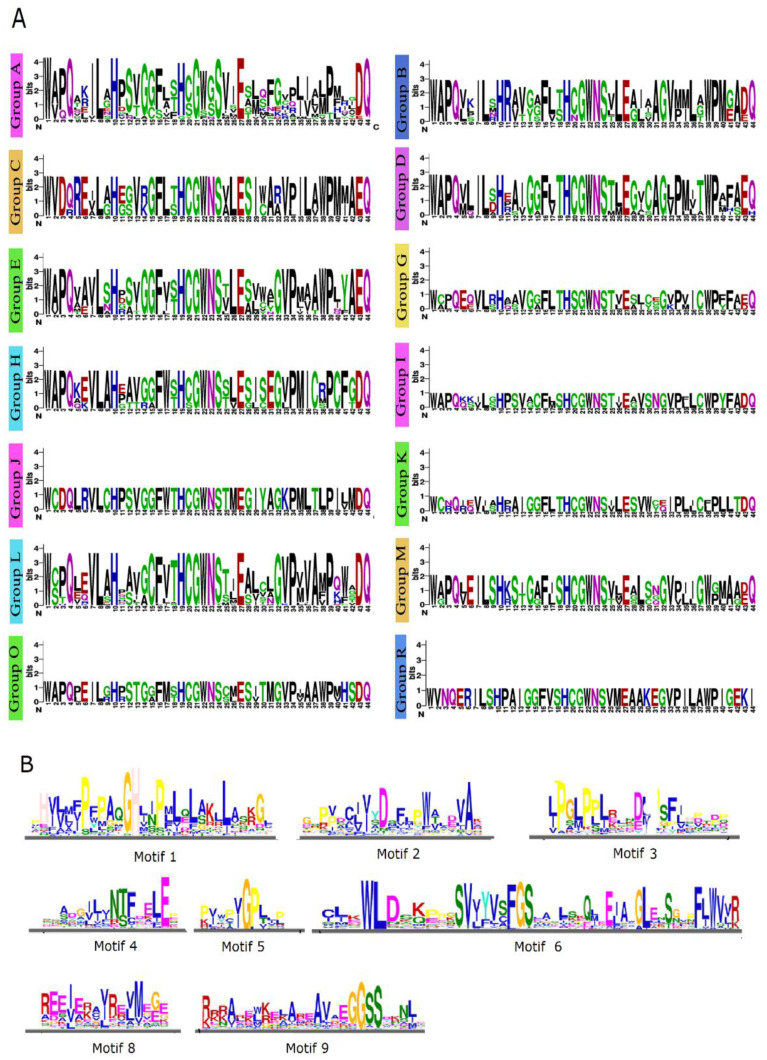
Conservative motifs of *Eu*UGTs. (**A**) The Weblogo of motifs of motif 7 (PSPG box) in each phylogenetic group of *Eu*UGTs; (**B**) the Weblogo of the other motifs in *Eu*UGTs.

All of the *Eu*UGTs’ amino acid sequences were submitted to the MEME website for analysis of the conserved motifs. Like the previous analysis [6,37], nine conserved motifs were identified within almost all of the *Eu*UGTs (Figure 2A). However, some *Eu*UGTs may lack one or more motifs. For example, motifs 2 and 3 are missing from three *Eu*UGTs (GWHPAAAL015516, GWHPAAAL024578, and GWHPAAAL024577) in group O (Figure 2A). Motif 5 is considered to separate the UGT sequence into the *N*-terminal and *C*-terminal regions. The *C*-terminal is better conserved than the other terminals. Motif 7—also called the PSPG box—is the UGT-defining consensus sequence and exists across all the putative *Eu*UGTs (Figure 2B). It is almost identical to the PSPG motif within the UGTs of *A. thaliana* and *L. usitatissimum*. The consensus sequence is highly conserved, especially at sites such as 1W, 4Q, 8L, 10H, 16F, 19H, 21GWNS24, 27E, 39P, 43D/E and 44Q. Further analysis showed some differences within the PSPG box in each group of *Eu*UGTs (Figure 3A). In addition to a few positions of motif 7 conserved in most *Eu*UGTs, there are also positions conserved within *Eu*UGT proteins from the same group, such as: 14G in group A; 14G and 28G in group D; 26L, 38W, 42A in group E; and 36V in group L.

Except for the PSPG box, the other eight motifs of the EuUGTs were found to be somewhat conservative (Figure 3B). However, the catalytic functions of these motifs remained unclear until now. By comparing the 3-D structures of several GT-B fold enzymes, it was found that these enzymes showed high structural similarity despite their lower sequence identity [38]. Therefore, it could be speculated that these motifs may play a role in maintaining the stability of the GT-B fold.

**Figure 4:**
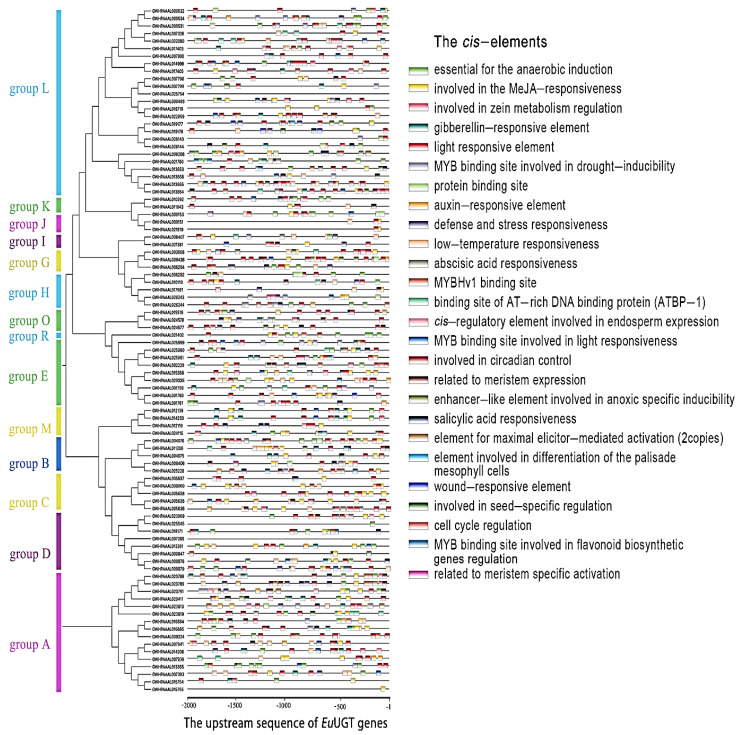
The predicated *cis*-acting elements localized within the 2000-bp upstream of *Eu*UGT genes.

The *cis*-acting elements, including the promoter, enhancer, silencer, and various response elements, are important in the regulation of gene transcription. Therefore, the 2000 bp 5′-upstream sequence of each *Eu*UGT gene coding sequence was retrieved and used to predicate the putative *cis*-acting elements on the PlantCARE webserver (http://bioinformatics.psb.ugent.be/webtools/plantcare/html/, accessed on 8 December 2020). Overall, 26 various *cis*-acting elements and their locations were predicted (Figure 4). Almost all the *Eu*UGT genes have one or more *cis*-elements, except for GWHPAAAL015755 and GWHPAAAL018171. It is worth noting that some *Eu*UGT genes have multiple tandem elements, such as light-responsive elements (GWHPAAAL014996 and GWHPAAAL018716), low-temperature-responsive elements (GWHPAAAL011200), plant-hormone-responsive elements (GWHPAAAL014206), and wound-responsive elements (GWHPAAAL019177), etc. Therefore, the expression of the *Eu*UGT genes is supposed to be extensively regulated.

### 2.4. GO Annotation of EuUGTs

**Figure 5:**
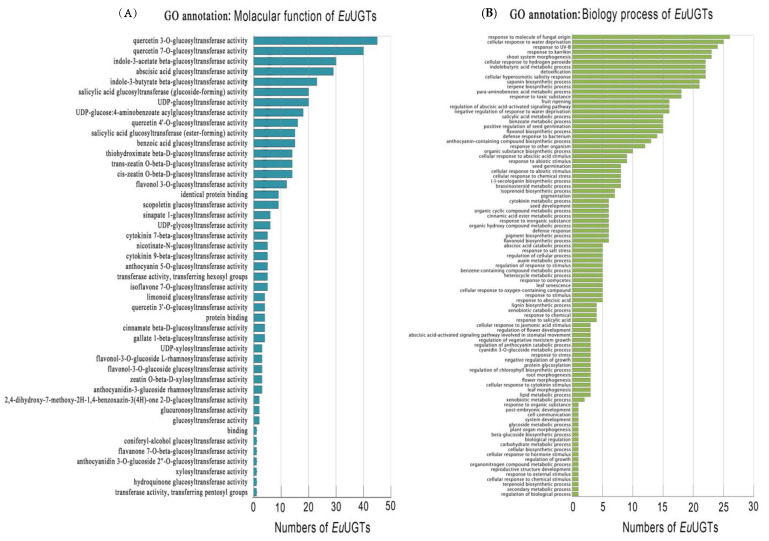
GO annotation of *Eu*UGTs. (**A**) Enrichment of *Eu*UGTs in molecular function; (**B**) enrichment of *Eu*UGTs in biological processes.

Through GO ontology annotation, the putative *Eu*UGTs were categorized into 45 molecular functions and 93 biological processes (Figure 5A,B, Table S3). Most of the *Eu*UGTs were annotated as flavonoids glucosyltransferase (quercetin 3-*O*-glucosyltransferase and quercetin 7-*O*-glucosyltransferase) and hormonal glycosyltransferase (indole-3-acetate beta-glucosyltransferase, indole-3-butyrate beta-glucosyltransferase, and abscisic acid glucosyltransferase). In addition, GWHPAAAL025999 was annotated as coniferyl alcohol glycosyltransferase, which may be related to the biosynthesis of lignans, such as PDG, a unique and valuable secondary metabolite in *E. ulmoides*. In view of these bioprocesses, most of the *Eu*UGTs were enriched to be involved in the responses to the various stresses, suggesting that the glycoside molecules could participate in the defense of abiotic and biotic stresses.

### 2.5. Transcriptome Analysis for Organ-Specific and Time-Specific Gene Expression

**Figure 6:**
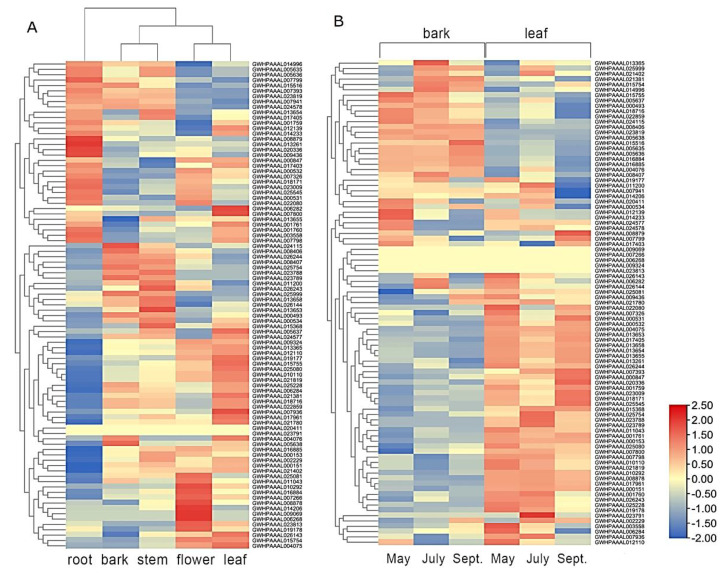
The transcriptome analysis of *Eu*UGT genes. (**A**) Heatmap of *Eu*UGT gene transcription in various organs. (**B**) Heatmap of *Eu*UGT gene transcription over various seasons. The TPM value of each *Eu*UGT gene was calculated by Stringtie and normalized with RowScale method by TBtools, and then used to construct the heatmap. In each row, red color represents higher expression level, blue color shows lower expression.

The third-party transcriptome data [33] were used to analyze the expression patterns of the *Eu*UGT genes in various organs and in different seasons. The relative transcriptional levels of the *Eu*UGT genes are summarized in Table S4, with TPM values ranging from 0 to 277,336, indicating that most of the *Eu*UGT genes were differentially expressed. Figure 6A shows the expression patterns of *Eu*UGT genes in various organs. Globally, many *Eu*UGT genes exhibited an organ-specific expression pattern, which was highly expressed in one or two organ(s), but lower in others. Based on the expression levels of all of the *Eu*UGT genes, the five organs could be roughly clustered into three groups: Root, bark/stem, and flower/leaf. The root showed a greater difference from the other four organs in terms of *Eu*UGT gene transcription. Some *Eu*UGT genes could be recognized as organ-specific based on their transcriptional level. For example, GWHPAAAL009436 and GWHPAAAL020336 tended to be expressed at higher levels in the root than in other organs; GWHPAAAL023788 was only expressed in the bark and stem. In contrast, GWHPAAAL007266 was highly expressed in the flower, but had extremely low expression in the other four organs. It was observed that all the eight *Eu*UGT genes from group D were expressed primarily in the root and flower. Meanwhile, 6 out of the 16 *Eu*UGT genes belonging to group A had higher expression in the flower than in the other organs. In addition, there were more highly expressed genes in the leaf than in the other four organs, which may be consistent with the fact that plant leaves contain many polysaccharides [39]. 

Furthermore, several *Eu*UGTs, such as GWHPAAAL015368, GWHPAAAL017961, GWHPAAAL000493, GWHPAAAL018716, GWHPAAAL022859, GWHPAAAL025754, GWHPAAAL025999, and GWHPAAAL07799, had higher expression levels in all five organs, with a TPM value greater than ten thousand, suggesting that these genes might be widely involved in the entire growth and development process.

The contents of many secondary metabolites exhibited a fluctuation over the seasons in *E. ulmoides*. For example, the content of some active ingredients, including PDG, aucubin, and chlorogenic acid, was reported to be higher from May to July than during the other seasons [40,41]. Therefore, we analyzed UGT gene expression in the bark and leaves during various seasons (May, July, and September). A large number of the *Eu*UGT genes did not show obvious fluctuations in transcription over the seasons in bark or leaves (Figure 6B). Nevertheless, a small fraction of the genes exhibited a season-dependent expression pattern. For example, the expression of genes such as GWHPAAAL000847, GWHPAAAL000531, GWHPAAAL020336, GWHPAAAL025545, and GWHPAAAL018171 in the leaf increased significantly in September compared to May. In contrast, some genes’ expression levels in the leaf were much higher in May compared to September. A similar situation occurred in the bark. Genes including GWHPAAAL025080, GWHPAAAL025081, GWHPAAAL025999, GWHPAAAL021381, GWHPAAAL007800, and GWHPAAAL015516 had significantly increased expression in the bark during September compared to May.

### 2.6. RT-qPCR Analysis of the Selected EuUGT Genes

**Figure 7:**
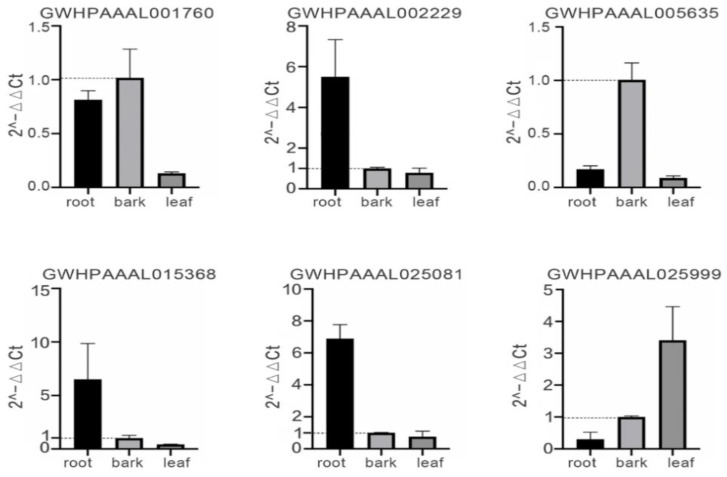
Quantitative real-time PCR analysis of six selected *Eu*UGT genes in root, bark, and leaves of *E. ulmoides.* The relative expression level of each *Eu*UGT gene was normalized to that in the bark. The β-actin was used as the reference gene.

Lignans, generated by the polymerization of three major monolignols (ρ-coumaryl, coniferyl, and sinapyl alcohols), constitute a major class of secondary metabolites [42,43]. In *E. ulmoides*, up to 46 lignans were identified in various organs or tissues. Most of these lignans belong to glycosidic compounds [16]. In order to discover the key UGT genes possibly involved in the glycosylation of lignans in *E. ulmoides*, RT-qPCR was performed to detect the expression level of six selected *Eu*UGTs belonging to groups E and C in root, bark, and leaves, which were sampled from three 18-year-old trees in the harvesting season (in June). Figure 7 shows the relative expression levels of the six genes in the three samples. Among them, GWHPAAAL002229, GWHPAAAL015368, and GWHPAAAL025081 were expressed more efficiently in root than that in the bark and leaves. The expression levels in the root were 5.5, 6.4 and 6.9 times as high as those in bark, and 7.0, 15.3 and 9.1 times as high as those in the leaves, respectively. The transcriptional level of GWHPAAAL005635 was increased by 5.9- and 11.3-fold in bark compared to the root and leaf, respectively. GWHPAAAL001760 had a higher expression level in the bark and root than in the leaves. The expression of GWHPAAAL025999 was noticeably up-regulated in leaf, but its transcripts were observed at a high level in all three organs.

### 2.7. Construction of Protein–Protein Interaction (PPI) Network of EuUGTs

**Figure 8:**
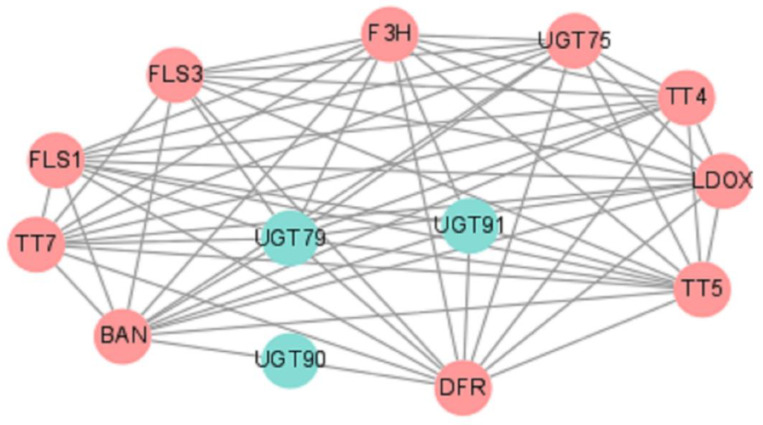
Protein–protein interaction network of *Eu*UGTs with *A. thaliana* as reference predicted by using String web server. The blue circles represent the input *Eu*UGTs; the red circles show the protein partners. BAN, NAD(P)-binding Rossmann-fold superfamily protein; DFR, dihydroflavonol reductase; FLS1, flavonol synthase/flavanone 3-hydroxylase; FLS3, flavonol synthase; TT4, flavonol synthase; TT5, chalcone-flavanone isomerase; TT7, cytochrome P450; F3H, naringenin,2-oxoglutarate 3-dioxygenase; LDOX, leucoanthocyanidin dioxygenase; and UGT75, anthocyanidin 5-*O*-glucosyltransferase.

All 91 *Eu*UGTs were used to construct the PPI network on the String web server (https://string-db.org/, accessed on 9 September 2021) [44]. As a result, a PPI network was obtained (Figure 8), in which nine *Eu*UGTs constituting three nodes (in blue circle) were predicted to be interacted with ten protein partners. All of the proteins included in this network are summarized in Table S5. The UGT79 (GWHPAAAL009324) was predicted to be interacted with 10 protein partners, such as BAN [NAD(P)-binding Rossmann-fold superfamily protein], DFR (dihydroflavonol reductase), FLS1 (flavonol synthase/flavanone 3-hydroxylase), FLS3 (flavonol synthase), TT4 (flavonol synthase), TT5 (chalcone-flavanone isomerase), TT7 (cytochrome P450), F3H (naringenin-2-oxoglutarate 3-dioxygenase), LDOX (leucoanthocyanidin dioxygenase), and UGT75 (anthocyanidin 5-*O*-glucosyltransferase). UGT91 (GWHPAAAL007941, GWHPAAAL015754 and GWHPAAAL015755) were interacted with five proteins (BAN, FDR, FLS1, TT5, and F3H). UGT90 (GWHPAAAL005635, GWHPAAAL005636, GWHPAAAL005637, GWHPAAAL005638, and GWHPAAAL009069) were only interacted with two proteins (DFR and BAN).

In view of the catalytic function of the proteins in the network, three possible biological processes were assigned in the biosynthesis of flavonoid, anthocyanin-containing compound, and pigment. For example, a large fraction of the protein partners, such as DFR, FLS1, TT5, TT4, TT7, FLS3, is involved in the biosynthesis of flavonoid; UGT75 and LDOX are included in the biosynthesis of anthocyanin-containing compound, which is downstream of the flavonoid biosynthesis [45].

## 3. Discussion

*E. ulmoides* is one of the important medical plants in East Asia. More than 46 lignans and their glycosides were identified in different tissues or organs of *E. ulmoides* [16]. However, no enzyme was found to be involved in their biosynthesis. Therefore, we performed a comprehensive analysis on the UGTs in silico. By searching the draft genome of *E. ulmoides*, 91 UGT genes were identified, which is less than the number in other plants, which in general have more than 100 UGTs. For example, *A. thaliana* contains 123 UGTs [6]; the tree *Quercus robur* has 244 UGTs [46]. In addition, three *Eu*UGTs (GWHPAAAL000153, GWHPAAAL020411, and GWHPAAAL023819) are clearly missing an *N*-terminal. This may be due to the incomplete genome of *E. ulmoides*. The *E. ulmoides* draft genome was assembled previously, with a size of 1.18 Gb which consisted of 29,348 scaffolds with an N50 of less than 1.03 Mb [33]. More than 10 pairs of *Eu*UGT sequences exhibited high similarity (Table S1). The same phenomenon was found in the other plants and is thought to be a result of tandem duplication during long-term evolution [2,6,47]. It may also reflect that the plant UGTs expand in order to produce more secondary metabolites [11,12]. As in other plant species, *Eu*UGT genes are mostly intronless, except for a small fraction of family members containing one to three introns, which may be derived from gain or loss events during their evolution [47].

The *Eu*UGTs were categorized into 14 previously identified groups, such as A to E, G to M, and O and R. Although the amnio acid sequence similarity is low among the UGT groups, the protein structure of the *Eu*UGTs was shown to be highly conserved. Nine motifs could be distinguished among the *Eu*UGTs (Figure 2A). Among these, motif 7 (PSPG box) was previously discussed extensively in an analysis of plant UGTs due to the highly conserved amino acid residues [5,6]. All 91 of the *Eu*UGTs also have a PSPG box with nearly identical conservative amino acid residues (Figure 2B). The function of the PSPG box is to serve as a binding domain of UDP-sugar [5]. Masada et al. (2007) replaced the PSPG box of *Ca*UGT2 with the *Nt*GT1b’s, leading to the loss of catalytic activity in the *Ca*UGT2 variant, indicating its important role in catalysis [48]. Further, site-directed mutagenesis of a non-conservative Cys377 in the PSPG box of *Ca*UGT2 to Arg led to the variant losing catalytic activity [48], suggesting that the non-conservative residues in the PSPG box in *Ca*UGT2 may also participate in substrate recognition. As Figure 2A shows, the other eight motifs were found to be conservative to a certain extent, but almost no studies have focused on them. Through *C*-terminal domain swapping, it was found that the affinity of the chimeric enzyme of *At*UGT71C1 and *At*UGT71C2 to the substrate etoposide changed significantly, suggesting that the substrate specificity of UGT may have resided in the *N*-terminal as well as the *C*-terminal domains [49]. Taken together, both the *C*-terminal and *N*-terminal domains of plant UGTs may be involved in substrate recognition, which is an important possibility to explore further in the future.

The *cis*-elements within the 2000 bp upstream sequence of each *Eu*UGT gene were identified. It was shown that up to 26 *cis*-elements were recognized, suggesting that *Eu*UGTs’ transcription might be extensively regulated. For example, after being treated with methyl jasmonate, several UGTs in the adventitious roots of *P. ginseng* were up-regulated [13]; in *Brassica* and *Arabidopsis*, changes in the transcriptional level of many UGTs happened in response to various stresses [50]. Our transcriptome analysis also confirmed that the transcription of most *Eu*UGT genes was organ-specific (Figure 6). In addition, it is worth noting that many of the *cis*-elements were enriched to be related to stress responses, such as low temperatures, wounds, and the stresses caused by bacteria and fungi, etc. GO annotation also categorized these *Eu*UGTs into the biological processes of stress responses (Figure 5B). Taken together, the tissue-specific expression of plant UGTs suggests that specific glycosylation may largely take place in a given tissue or organ. For example, several UGT genes were reported to be highly expressed in germinating seeds of *Cicer arietinum* and were localized in the regions of rapidly dividing cells; thus the authors suggested that these tissue-specific expressed UGTs may be involved in cell cycle regulation [51]. Furthermore, the transcriptomic data may help to identify the UGTs potentially involved in the biosynthetic pathways of a given glycoside [13,14,52].

Since many bioactive compounds isolated from *E. ulmoides* belong to lignan and their glycosylated derivatives, such as aucubin, PDG, etc., it is interesting to identify the genes involved in their biosynthesis [25,26,53]. For example, PDG is a kind of natural glycosylated lignan existing in several plant species, such as *E. ulmoides* [16], *L. usitatissimum* [54], *Actinidia arguta* [55], and *Valeriana officinalis* [56]. Previous studies have proposed that the upstream biosynthesis pathway of PDG starts from phenylalanine, then synthesizes cinnamic acid, coumaric acid, coffee acid, ferulic acid, and finally coniferyl alcohol, through the phenylpropanoid pathway [57]. This pathway has been reconstituted in *E. coli*, which could utilize glucose as a substrate to produce coniferyl alcohol [58]. Coniferyl alcohol has been proposed as a precursor molecule to the synthesis of PDG through dimerization to form pinoresinol, which is then glycosylated to produce PDG [59,60]. The pathway of PDG biosynthesis from coniferyl alcohol in plants is not defined and no enzyme has been identified to catalyze the glycosylation of pinoresinol to produce PDG. However, a coniferyl alcohol glucosyltransferase was purified and characterized biochemically from the cambial sap of spruce (*Picea abie* L.) [61]. Two genes, UGT71A18 (found in *Forsythia* *koreana* [62]) and UGT71C1 (found in *A. thaliana* [63]), were shown to catalyze the glycosylation of pinoresinol to produce pinoresinol monoglucoside. In addition, some other UGTs were also found to be related to the lignin biosynthesis [52,64,65,66]. For this purpose, we screened out six *Eu*UGTs for an expression analysis by combining the sequence characteristics and their expression patterns in various organs. GWHPAAAL001760 and GWHPAAAL015368 were clustered into group E with a sequence similarity close to UGT71, and two proteins belonging to this clade in *F.* *koreana* and *A. thaliana* were reported to catalyze pinoresinol to form pinoresinol monoglucoside [62,63]. In addition, GWHPAAAL025999 was annotated to be coniferyl alcohol glucosyltransferase. The other two *Eu*UGTs (GWHPAAAL002229 and GWHPAAAL025081) were also selected from group E. As shown in our transcriptomic data, another *Eu*UGT (GWHPAAAL005635) showed higher transcription levels in the root and bark, which were previously reported to contain higher PDG content [67,68]. Thus, an RT-qPCR was performed to verify the transcription levels of the selected UGT genes in root, bark, and leaves. It was shown that five selected *Eu*UGT genes, with the exception of GWHPAAAL025999, were transcribed much more efficiently in root or bark than in the leaf, which is consistent with the higher content of PDG and other glycosidic metabolites in the bark and root [67,68]. However, a deviation appears between the qPCR data and transcriptome profiling for some *Eu*UGT genes. For example, the highest expression of the GWHPAAAL005636 gene was observed in bark when using a qPCR analysis but appeared to be in the root when using a transcriptome analysis. These differences may arise from the different sampling locations and trees used in each analysis. Taken together, these results suggest that these *Eu*UGTs could be candidates for future research on the biosynthesis of PDG and other glucosides. The catalytic functions of the selected *Eu*UGTs should be verified experimentally. For this purpose, our laboratory is studying the cloning and expression of the cDNAs and the encoding these *Eu*UGTs.

**Figure 9:**
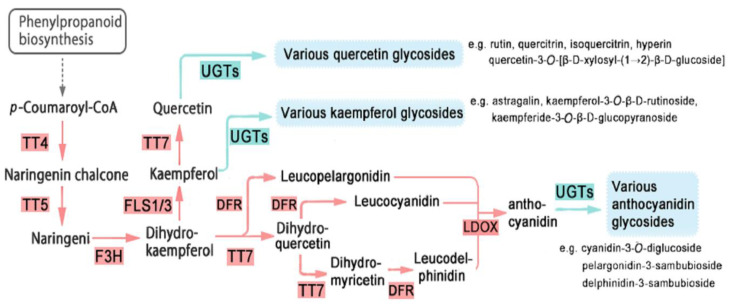
A proposal scheme of biosynthesis of flavonoids and their glycosides in *E. ulmoides*. BAN, NAD(P)-binding Rossmann-fold superfamily protein; DFR, dihydroflavonol reductase; FLS1, flavonol synthase/flavanone 3-hydroxylase; FLS3, flavonol synthase; TT4, flavonol synthase; TT5, chalcone-flavanone isomerase; TT7, cytochrome P450; F3H, naringenin,2-oxoglutarate 3-dioxygenase; LDOX, leucoanthocyanidin dioxygenase; and UGT75, anthocyanidin 5-*O*-glucosyltransferase; UGT, UDP-Glycosyltransferase.

In addition to the lignan compounds, various organs or tissues of *E. ulmoides* also contain plenty of flavonoids and their glycoside derives, such as quercetin, hyperin, and rutin [16]. Thus, we construct a PPI network with 91 *Eu*UGTs to identify the UGTs that may be involved in biosynthesis of flavonoids and their glycosides. Through the PPI network, a biosynthetic pathway of flavonoids in *E. ulmoides* could be schemed based on the previous proposal [45,69]. As shown in Figure 9, almost all the key enzymes can be deduced in *E. ulmoides* (Table S5) in this pathway. In the PPI network, UGTs constitute three nodes. UGT79 (GWHPAAAL009324) from group A was annotated to encode anthocyanidin 5-*O*-glucosyltransferase, which may participate in the biosynthesis of anthocyanidin glycosides. The other two nodes consist of UGT90 (belonging to group C, containing four *Eu*UGT members: GWHPAAAL005635, GWHPAAAL005636, GWHPAAAL005637, GWHPAAAL005638, GWHPAAAL009069) and UGT91 (belonging to group A, containing three members: GWHPAAAL007941, GWHPAAAL015754, and GWHPAAAL015755) in *E. ulmoides*. All of the above *Eu*UGTs may be involved in the conversion of quercetin or kaempferol into various quercetin or kaempferol glycosides, respectively, such as isoquercitrin, rutin and so on. However, the exact *Eu*UGT cannot be assigned into each glycosylation reaction such as that present in Figure 9, which must be confirmed experimentally.

## 4. Materials and Methods

### 4.1. Data Resources Used

The draft genome and annotation of *E. ulmoides* [33] were downloaded from the National Genomics Data Center under BioProject accession PRJCA000677 (https://bigd.big.ac.cn/bioproject/browse/PRJCA000677, accessed on 17 March 2020). The transcriptome data of *E. ulmoides* were downloaded from NCBI under BioProject accession PRJNA357336. One hundred and seven UGT sequences of *A. thaliana* (two UGT80s not included) were obtained from Uniprot (https://www.uniprot.org/, accessed on 17 March 2020) and 137 UGT sequences of *Linum usitatissimum* L. were obtained from NCBI (JN088282 to JN088418). In addition, the UGT708C1 (XP_007216617) of *Prunus persica* [70] and the UGTPg36 (AKA44596.1) of *P. ginseng* [71] were used to construct the phylogenetic tree.

### 4.2. Genome-Wide Identification of Putative UGTs in E. ulmoides

The hidden Markov model UDPGT (PF00201.18) obtained from the Pfam database (http://pfam.xfam.org/, accessed on 5 May 2020) was used to search for the putative UGTs against the *E. ulmoides* draft genome by employing the HMMER-3.3 software with a cut-off *E*-value 1 × 10^−5^ [72]. Key word searches were also performed through genome annotation using the words ‘UGT’, ‘glycosyltransferase’, and ‘glucosyltransferase’. All sequences screened by the two above methods were merged and de-duplicated. ClustalW was used to perform multiple sequence alignment and the MEME web server (http://meme-suite.org/tools/meme, accessed on 6 May 2020) [73] was used to find conserved motifs within the candidate *Eu*UGTs. We re-defined the complete sequence of *Eu*UGTs, since parts of genes appear to lack the *N*-terminal or the *C*-terminal when compared to the UGTs of *A. thaliana* and *L. usitatissimum.* The upstream and downstream sequences of putative UGT genes were extracted by Python 3.7 in order to re-annotate them using FGENESH and Softberry Online Services (http://www.softberry.com/, accessed on 1 September 2020) [74]. The transcriptome was assembled with and without the reference genome, using Trinity software [75] in order to help to determine the correct CDS of putative genes. After removing sequences with conservative motifs of less than 3 and without a complete PSPG box, the putative *Eu*UGTs were obtained and further verified by matching the Pfam families on the HMMER website (https://www.ebi.ac.uk/Tools/hmmer/search/hmmscan, accessed on 13 September 2020).

### 4.3. Prediction of the Cellular Location, Physical and Chemical Properties of EuUGTs

The subcellular localization of putative *Eu*UGTs was analyzed by the CELLO v2.5 online server (http://cello.life.nctu.edu.tw/, accessed on 18 September 2020) [76,77]. The Protparam tool on the ExPASy web server (https://web.expasy.org/protparam/, accessed on 18 September 2020) [78] was used to the calculate sequence length, molecular weight, theoretical pI, instability index, and the grand average of hydropathicity of the *Eu*UGTs.

### 4.4. Phylogenetic Analysis of UGTs among A. thaliana, L. usitatissimum and E. ulmoides

The amino acid sequences of the selected UGTs, including 107 UGTs of *A. thaliana*, 137 UGTs of *L. usitatissimum*, and 91 putative UGTs of *E. ulmoides*, along with UGT708C1 as a member of phylogenetic group R [70] and UGTPg36 as a member of phylogenetic group O [71], were aligned by ClustalW in MEGAX software [79]. Then, the output file was used to construct a neighbor-joining tree with the p-distance method and pairwise deletion.

### 4.5. Analysis of Conserved Motifs and Prediction of Cis-Acting Elements

MEME web server [73] was used to search conserved motifs within all the *Eu*UGTs. The 44 residues of the PSPG box at the *C*-terminus were selected to draw a motif logo using the online Weblogo server (https://weblogo.berkeley.edu/logo.cgi, accessed on 18 September 2020) [80]. PlantCARE (https://bioinformatics.psb.ugent.be/webtools/plantcare/html, accessed on 20 September 2020) was applied to predict the *cis*-acting elements within the 2000 bp upstream of each *Eu*UGT gene [81].

### 4.6. GO Annotation of EuUGTs

The protein sequences of putative *Eu*UGTs were used to blast against 109 UGTs of *A. thaliana* using Omicsbox software followed with Blast2Go [82] to obtain the molecular function and biological process of each *Eu*UGT.

### 4.7. Transcriptome Analysis for Organ-Specific and Season-Specific Gene Expression

The RNA-seq datasets generated from different organs and seasons were downloaded from the NCBI SRA database (https://www.ncbi.nlm.nih.gov/sra/, accessed on 12 July 2020) [33]. The information on plant samples and the RNA-seq are summarized in Table S6. The expression level of each *Eu*UGT gene were calculated in TPM value by using the Hisat2 [83], Samtools, and Stringtie [84] software, during which 91 *Eu*UGT gene sequences were input as references. The heatmaps of *Eu*UGT gene expression patterns were constructed by TBtools [85].

### 4.8. Quantitative Real-Time PCR Analysis of the Selected EuUGT Genes

In June 2020, three plant samples of *E. ulmoides*, including bark, leaves, and root, were collected from three 18-year-old trees at a local *E. ulmoides* forest farm (103° E, 30° N) in Chengdu, China. A piece of bark approximately 5 × 5 cm^2^ in size was peeled from each individual trunk at approximately 0.5 m above the ground. The roots were collected from the fibrous roots with a diameter of approximately 5 mm. Three mature leaves were sampled on an individual tree. All of the plant materials were immediately immersed in liquid nitrogen after sampling. The plant materials were smashed in liquid nitrogen, and then used to isolate the complete RNAs using the CTAB protocol [86]. Reverse transcription and the real-time PCR were performed using the PrimeScript RT kit and TB Green Premix Ex Taq II (Takara Bio Co. Dalian, China) following the manufacturer’s guides, respectively. Six *Eu*UGT genes were selected to confirm the organ-specific expression, with β-actin as the reference gene. The primers are listed in Table S7. The qPCR reaction was performed with the following parameters: An initial denaturation for 30 s at 95 °C; then, 45 cycles, including denaturation, for 5 s at 95 °C; annealing for 30 s at 55 °C; and extension for 30 s at 72 °C. The result was calculated with the following formula [87]:

2^−^^ΔΔCt^ = 2^−(^^ΔCt(root/leaf) −^
^ΔCt(bark))^; ΔCt(root/bark/leaf) = Ct(root/bark/leaf) − Ct(actin)

### 4.9. Construction of Protein–Protein Interaction (PPI) Network of EuUGTs

In order to exploit the biological functions of *Eu*UGTs, a PPI network was constructed. Briefly, 91 sequences of *Eu*UGTs were input into the String web server with *A. thaliana* as the reference (https://string-db.org/, accessed on 9 September 2021) [44] to predict the PPI network. The generated result was imported into Cytoscape [88] for plotting.

## 5. Conclusions

An increasing number of studies have shown that UGTs play an important role in various biological processes in organisms. Therefore, the identification and in silico analysis of UGT gene families in different species have attracted many researchers. Up to now, almost no studies have been performed on the UGT family of *E. ulmoides*, an important pharmacological woody plant. Here, we identified 91 *Eu*UGTs in *E. ulmoides* through genome-wide analysis, which could be clustered into 14 out of the 18 previously described phylogenetic groups (A to R). However, no *Eu*UGT was categorized into groups F, N, P, or Q. Group L was the largest group of *Eu*UGTs, which has a wide variety of substrates. Through transcriptome analysis, differentially expressed patterns were observed for most of the *Eu*UGT genes, which tended to be expressed at higher levels in one or two organs. The organ-specific high expression of the *Eu*UGT genes may be due to the specific metabolism requirements in the organs or in response to various stresses. Through the in silico analysis of the protein–protein interaction network of *Eu*UGTs, a set of proteins including some UGTs were identified, perhaps to constitute a biosynthetic pathway of flavonoids and their glycosides in *E. ulmoides*. Combining multiple types of information from the phylogenetic analysis, the structural characteristics, and the function predictions was helpful to our exploration of the UGTs of interest. Our results could lay the foundation for further studies on UGTs and the biosynthesis of secondary metabolites of *E. ulmoides*.

## Data Availability

Not applicable.

## Supplementary Materials

The following are available online at https://www.mdpi.com/article/10.3390/plants10091934/s1, Supplementary file S1: List of the nucleotide sequences of 91 *Eu*UGT genes; Supplementary file S2: List of the amino acid sequences of 91 *Eu*UGTs; Figure S1: Organization of the *Eu*UGT genes; Table S1: Characteristics of *Eu*UGT genes with highly similar sequences; Table S2: Physical and chemical features and subcellular location of *Eu*UGTs; Table S3: GO annotation of *Eu*UGTs; Table S4: The transcriptional level of *Eu*UGTs in TPM value in various organs and on various seasons; Table S5: Summary of proteins involved in the protein–protein interaction network; Table S6: Summary of RNA-seq datasets used for transcriptome analysis; Table S7: The nucleotide sequences of primers used for RT-qPCR.
