# Supplementary material for: Genomic-Wide Identification and Characterization of the Uridine Diphosphate Glycosyltransferase Family in Eucommia ulmoides Oliver
Source: Plants (Basel). 2021 Sep 17;10(9):1934. doi: 10.3390/plants10091934 (PMC8471388; doi:10.3390/plants10091934)
Supplement: Supplementary file 1 [file plants-10-01934-s001.zip › plants-1355377-supplementary/Supplementary File 1 (nt).pdf]

List of the nucleotide sequences of *Eu*UGT genes

>GWHPAAAL000151
ATGGTACCGATTAGTACGGAACCAATCCGTAAATGCCACGTGGTCGCCGTTCCTTACCCCGGCAGGGGCC
ACGTTAACCCCATGATGAACATCTGCAAGCTGATAGCCTCCAAATTCGACCCCGACCGTCTCGTCATCAC
GTTCGTCGTCACCGAGGAATGGCTGGGTCTAATCGGCTCCGAGATCAAGCCGGCGAACGTCCGCTTCGCC
GCCATTCCGAATGTCGTGCCGTCGGAGCGGGTTCGAGCCGCCGACATGGTTGGGTTCGTCACGGCGGTTA
TGACTAAAATGGAACAGCCGTTTGAGGAGGTCCTGGATCGGATGCAATCTCCGGCGAGTGTGATAATAGC
GGATACTTTCCTGCGGTGGGCGGTGGAGGTGGGGAACCGGAGGAGTATTCCGGTGGCGTCGCTGTGGACG
ATGCCTGCGATGGTATTTTCGGTATTTTACAATTTTGAGCTTGTGGTTGAGAATGGGCAATTTCCCATTG
AATTGTGTGAAAGAGGAGAGGAGCGCGTGGATTACTTACCCGGAATTCCCTCAATTCGTCTCTCCGACCT
CCCAACGATCATTCACCGAAGAGATCAGAACCTCCTCCGCCTCTGCCTTACCGCTTTCGCCAATGTTTCC
AGAGCACGGTACCTCTTATTCACCACCTGCTCCGATCTCGAACCCCAAGTCATCGCCGCCTTGAAGACAC
AATTTCCGTTCCCGTTGTACTGTCTCGGCCCGACCATACCCTATTTCAACCTCCAACAACCACCCCCAAT
CACCCCCAACCACCACGACTACCTAAAATGGCTAGACTCAAAACCACCAAATTCAGTTCTGTACGTGTCA
CTGGGGAGTTTTCTGTCGGTTTCGAGCTCCCAAATGGACGAAATCGAAGCCGGATTGGGCGGCAGCGGCG
TGAATTACTTGTGGGTAAACCGTGGTCATGCTGGCGGCGTCGGTGGAGGAGGAAGGATGGTGGTGCGGTG
GTGCGACCAGCTAAGGGTTTTGTGCCATCCGTCGGTGGGTGGATTCTGGACGCACTGCGGGTGGAATTCG
ACGATGGAGGGGATTTACGCCGGGAAGCCGATGCTGACGCTTCCGATACTGATGGATCAGGTGCCAAACA
GCAAGTGCGTGGTGGAGGATTGGAAGATCGGATGGGAGGTGAAGAGAGAGGAGGGTAATTTGGTGAGTAG
AGAGAAGATTGTGGAGGTGGTGCGGAGGTTTATGGATGAGGGAAGTGAGGAGAGGGAGGAGATGATGAGG
AGGGCGGAAGAGCTACGTCGATTGTGCCGGACGGCGCTCGTCCGAGGAGGTTCTTCCCACGAGAATCTCC
AAGCTTTTGTCGATGGAATTGCCAAATTTTAA
>GWHPAAAL000153
ATGGCAGAAAGAGGAGAGGAACGCGTGGATTACCTCCCCGGAATTCCCTCAATTCGTCTCGCCGACCTCC
CGACGATCATCCACCAAAGTGATCAGAACCTCCTCCGCCTCTGCCTTACCGCTTTCGCCAATGTTTCCAG
AGCACAGTACCTCTTATTCACCACCTGCTCCGATCTCGAACCCCAAGTCATCGCCACCTTGAAGACACAA
TTTCCGTTCCCGCTGTACTGTCTCGGCCCGACCATACCCTATTTCAACCTCCAACAACCACCGCAAACCA
CCCCCAACCACCACGACTACCTAAAATGGCTAGACTCAAAACCACCAAATTCAGTTCTGTACGTGTCACT
AGGGAGTTTTCTGTCGGTTTCGACCTCCCAAATGGACGAAATCGAAGCCGGATTGGGCGGGAGCAGCGTG
AATTACTTGTGGGTAAACCGCGGCGATGCTGGCGGCGTCGGCGGAGGAGGAGGGATGGTGGTGCGGTGGT
GCGACCAGCTAAGGGTTTTGTGCCATCCGTCGGTGGGTGGGTTCTGGACGCACTGCGGGTGGAATTCGAC
GATGGAGGGGATTTACGCCGGGAAGCCGATGCTGACGCTTCCGATAGTTATGGATCAGGTGCCGAACAGT
AAGTGCGTGGTGGAGGATTGGAAGATCGGATGGGAGGTCAAGAGAGAGGAGGGTAATTTGGTGAGGAGAG
AGAAGATTGTGGAGGTGGTGAGGAGGTTTATGGATGAGGGAAGTGAGGAGAGGGAGGAGTTGGTGAGGAG
GGCGGAAGAGCTACGGCGGTTGTGCCGGACGGCGCTCGGCAGAGGAGGTTCATCCCATGAGAATCTCCAA
GCTTTTGTCGATGGAATTGCCAAATTTTAA
>GWHPAAAL000493
ATGGAGCAGCGAGAGTACAGAGCCCATGTCCTGGCAATTCCATTTCCGGCACCAGGCCATGTTAACCCAC
TTCTCCAATTCTGCAAGCGCCTGGAATTCAAGGGCTTGAAGGCGACTCTCGCCATCACCAAATTCCTGTC
CAAATCCATGCAATCCAGACTCGGGTCGATCAAGATCGACACGATCTCCGACGGGTACGATGAGATTGGA
CGGCACAACGCTGAGAGCCTCGGCGTATGGATCTCGACCTTCAAGGAAGTAGGTTCGAAATCCGTGGCGG
ATCTCATCCATAAGTTTCGGAGCTTGGGTCATCCGATCGATTGCATCGTCTACGATTCGTTTCTCCCTTG

GGTTCTGGATGTCGCCAAGGAGAACGGAGTTGCCGGAGCTTCGTTCATGACCCAGAAGTGTGCGGTGAAT
CACATCAACTATCATGTCTACCATAAGAAGATCCCCTACCCCTTTTCTTCTCCGACGTACTCGATTCCGG
GATTGCCGTCGCTCGATCTCGAGTACATGCCCGGATTTATGCTCGTCCACCCCGATTACTACGAGCTGGT
TCTCAGTCAATTCTCGACTGTGGATCGGGCTGATTACGTGTTCGTCAATACTTTCTACAAATTGGAGGCT
GAGGTATTGGATGAAATGTCAAAGGTCCTTCCAGTGAAACCAATAGGACCAACAGTCCCATCTTTCTACT
TGGACAACAGGGTTGAAGATGACAAAAACTATGGGCTCAACCTCCACCAACTGGACTCGTCGGTTTGCCT
CAATTGGCTCAGCTCTAAGCCTGCAAGGTCTGTGGTTTACGTGTCGTTTGGCAGCGTGGCCATATCCAGT
CCAGCAGCCAACGAAATGGAGGAGATAGCGTGGGGGTTGAAGAACAGCAATTGCTCCTTCCTGTGGGTGG
TGAAGGCTAACGAAGAGCCAAATCTCCCCCAGAATTTCAAGGAGGAGATGTCCGAAAAAGGATTGATCGT
GGCGTGGAGTCCCCAGATAGAGGTACTGTCACACCATTCAGTTGGGTGCTTCCTGACTCACTGTGGCTGG
AATTCAACCATCGAAGCTATTTGTCTGGGCGTTCCAATGGTGGCTGTGCCCAAATGCACGGATCAAACCA
CCAACTCGAAGTCCATACAGGATGTTTGGAAAGTTGGGGTCAAAGTTAAGGCATCCGAAGATGGGATTGC
TAGAAGAGAAGAGATTGAGCGGTGTATTAAGGTTGTAATGGAGGGGGAGAGCGGGGAGGAATTGAAGAAG
AATGCGGCCAAATGGAGTGAGTTGGCTAAGGAGGCGGTGAGCGAGGGCGGAACCTCTGATGAAAACATCA
ATGAATTTGTGTCTCAATTGACAAACTCCTCATCAACCCAATCTTGA
>GWHPAAAL000531
ATGGAAAGAGATTTAGATGAAATGGGTAGCAAAAAACCTCACGTTCTAGTAATCCCATTTCCAGCACAAG
GTCACTTTAGTCCTGCACTGCAACTCTCCAAGCGCTTAGCCTCAAAAGGGCTTAGGGTCACTTTCGTCAC
CTCCATGAGCAGCGCGAAGTCCAGTAGTTCCATTAGGATGGAGCAAATTGGCGATGGCTACGAAGAAGGT
GAAGAACCTGAGAGCAAAGAAGCCTTTACTGAGCATATCCAAACTGTATTCTCGCAAAGCCTAGGTGATA
TAATTGAGAAACAAAAACAAATCGGTTATCCTATTAAACTAGTTATTTATGATTCTGTTATGCCGTGGGC
ACTAGAGATAGCACATAAGTTGGGCATTTATGGAGCTCCATTCTTCACTCAACCCTGCGCTGTATGTGCC
ATCTATTACCATATGCAACAAGGGACTCTGGAAGTTTCTCTTGAAGGAACAAAAGTCTCCCTACCTTCAT
TGCCGCCATTGGAGAAAAATGATCTGCCATCTTTCATCTCTAACATGTTCTCACACGAATTTCTCACGAA
AACGATATTGGGCCAATTCTCCAATTTCCATGAAGCAGATTGGATTTTATTCAACACCTTCGACAAGCTG
GAAGATGAGGTAGTGAAGTGGATGGCAAGCCAGTGGCCTATCAAAACAATGGGACCAACTATTCCATCTT
TGTACTTAGACAAGCGACTTAAGGAAGACAAAGAATATGGCCTCAGCCTCTTCACTCCAAACACCGAGGC
TAGTATGAGGTGGCTGGACACGAAAGAACAAGGTTCGGTGGTTTATGTCTCATTTGGGAGCTTGGCCATC
CTTGATGAAACTCAAATGGAGGAATTATCATGGGGCCTTTTGCAGACCAACAACTACTTCCTGTGGGTGG
TCAGAGCTTCCGAAGAGAGCAAGCTTCCCAACAATTTCATGGCGAAGACCTCAGAGAAAGGGTTGGTGGT
CGATTGGTGCCCGCAGCTTGAGGTCTTGGCTCACCAGGCGGTAGGATGTTTCGTGACTCACTGTGGTTGG
AATTCGACGCTTGAAGCTTTGTGCCTTGGGGTGCCAATGGTTGCTATGCCCCAGTGGGTTGATCAAACGA
CTAATGCGAAGTTCGTGGCTGATGTATGGAAAACAGGGGTTCGAGTGGAGGTCAATGATAAGGGGATTGT
TACGAGAGAAGAGATTAAAAAACGAATCGGAGAAGTCATGGTGGGAGAAACGAGGGAAGAACTAAGAAAT
AATGCTAGCAGGTGGAAAAACTTAACGAGAGAGGCAGTGGATGAAGGTGGGAGTTCTGATAAGAATATTG
AGGAATTTATTTCTAAACTAGTATGCTCCTAA
>GWHPAAAL000532
ATGGAAAGAGATTTAGATGAAATGGGTAGCAAAAATCCACATGTTCTAGTAGTCCCATTTCCAGCACAAG
GTCACTTTAGTCCAGCGCTGCAACTCTCCAAGCGCTTAGCCTCAAAAGGGCTTAGGGTCACCTTCGTCAC
CACCATTAGCAACGCCAAGTCTAGGGGTTCCATTAGGATGGAGCGAATTACCGATGACTACGAAGAAGGT
GAAGAACCCGAGAGCATAGAAGCCTTTATCGAGCGTATGAAAGTCGTAATATCACAAAGCCTAGGTGATA
TAATTGAGAAACAAAAACATGCCGGTTACCCTGTTAAACTAGTTGTTTATGATTCTATTATGCCATGGGC
ACTAGAGATAGCACATAAGTTGGGCGTTTATGGAGCTCCATTCTTCACTAACCCCTGCACTGTATGTGCT
ATCTTTTACCATATGCAGCAAGGGACTGTGGAAGTTCCTCTTGAAGGAACAAAAGTCTCCCTGCCTTCAT

TGCCGCCATTGGAGGAAAATGATCTGCCATCTTTCATCTCTAACTTGGTCGCACCTGAATTTATCAAGAA
AATGTCATTGGGTCAATTCTCCAATTTCCATAAAGCAGATTGGATCTTATTAAACACCTTTGACAAGTTG
GAAGATGAGGTAGTGAAGTGGATGGCAAGCCAATGGCCTATCAGAACCATCGGACCAACTATTCCATCCA
TGTACTTAGACAAGCGACTTAAGGAAGACAAAGAATACGGCCTCAGCCTCTTCACTCCAAACACCAAGGC
TAGCATGAAGTGGTTGGACACGAAGGAACAAGATTCGGTGGTTTATGTCTCATTTGGGAGCCTGGCCACC
CTTGATGAAACTCAAATAGAAGAATTATCATGGGGCCTTGTGAATAGCAACAACTACTTTTTGTGGGTGG
TCAGAGCTTCCGAAGAGAGCAAGCTTCCCAGCGATTTTATGGCCAAGACCCTAGAGAAAGGGCTGGTGGT
CAATTGGTGCCCCCAGCTTGAGGTCTTGGCTCACCAGGCGGTGGGATGTTTCGTGACTCACTGTGGTTGG
AACTCGACGCTTGAAGCTTTGTGCCTTGGGGTGCCAGTGGTTGCTATGCCCCAGTGGGGTGATCAAACGA
CTAATGCGAAGTTCGTGGCCGATGTGTGGAAGGTAGGGGTTCGAGTGGAGGCCAATGATAAGGGGATTGT
TACGAGAGAAGAGATTGAGAAACAAATTGGAGTAGTCATGGCGGGAGAAACGAGGGAAGAACTGAAAAAG
AATGCTAGTAGTTGGAAAAACCTGGCAAGAGAGGCAGCAGATGAAGGCGGGAGTTCTGATAAGAATATTG
AAGAATTTGTTTATGAACTAGTATGCTCCTAA
>GWHPAAAL000534
ATGGCAAGAGATTTAGATGAAATGGGTAGCAAAAAACCTCATGTTCTTGTAGTCCCATTTCCAGCACAAG
GTCACTTTAGTCCAACACTGCGACTCTCCAAGCGCTTAGCCTCAAAAGGACATAGGGTCACTTTCGTCAC
CACCATTAGCAGTGCCAAGTCCAGTGGTTCCATTAGGATGGAGCAAATTACCAATGACTACGAAGAAGAG
ATAGCACATAAGTCGGGCGTTTATAGAGCTCTATTCTTCACTCAACCCTACGATGTATGTGCTATCTTTT
ACCATATGCAACAAGGGACTCTAGAAGTTCCTCTTGAAGAAATAAAAGTCTCTCTACCATCATTGCCGCT
ATTGGAGGAAAATGATATGCCATCTTTCATCTCTAACTTGTTCTCACACGAAATTATCAAGAAAACAGTA
TTGGGCCAATTCTCCAATTTCCATAAAGCAGATTGGATCTTATTGAACACCTTTGACAAGCTAGAAGATG
AGGTAGTGAAGTGGATGGCAAACCAGTGGCCTATCAAAACCATCGGACTAACTATTCCATCCATGTACTT
AGACAAGCAACTTAAGGAAGACAAAGAATACGGCCTCAGCCTCTTCACTCCAAACATCGAGACTAGCATG
AAGTGGTTGGACACAAAGGAACAAGGTTCAGTGAGCAACAACTACTTTCTATGGGTGGTCAGAGCTTCTG
AAGACAGCAAGTTTCCCAACAATTTCATGGCCAAGACCTCAGAGAAAAGGCTGGTGGTCAATTGGGGCCC
TCAACTTGAGGTCTTGGCTCACCAGGCAGTGGGATGTTTCGTGACTCACTGTGGTTGGAACTCAACGCTT
GAAGTTTTGTGCCTTGGGGTGCCAATGGTTGCTATGCCCCAGTGGACTGATCAAACGACTAATGCGAAGT
TCGTGGCTGATGTGTGGAAGGCAGGGGTTCGAGTAGAGGTTAATGATGAGGGGATTATTACGAGAGAAGA
GATTAATAAACGAATTGGACAAGTCATGGCAGGAGAAACGAGAGAAGAACTGAGAAAGAATGCTAGCAGT
TGGAGAAACCTGGCGAGAGAGGCAGTAGATGAAGGTGGGAGTTCTGATAAGAATATTGAAGAATTTGTTT
CTAAACTAGTACAAATGCCAATGTATAATCTATAA
>GWHPAAAL000847
ATGGCCTCTAAACCCCATTTCATCTTGGTACCCCTATTGGCCCAAGGCCACATGATCCCCATGATCGACA
CCGCGAAGCTCTTGGCTCATCAAGGCGTCCTCATCACGATCATCACGACGCCCGTCAATGCCGACAGCAT
CAAACCAACAGTAAATCGCGCCGTCAACACAGGCCTGGAAATCCGAGTTGCCAGACTCCGTTTTCCATGC
GCTGAGGCTGGGCTGCCGGAGGGCTGCGAGAATTTGGACATGCTCCCTTCGCCGGAGGCCGGCATCAAGT
TTTTCAATGCGCTCGGCATGCTGAAGGCACCGATGGAGAACCTGCTCGGCGAGATCGAGCCCTCGGCCAC
ATGCATAATCTCCGACATTTGTCCGCCGTGGACGATGCACGTGGCTAAGAAATTTGCATCCCGAGAATTG
TTTTCTCCGGCACCGATCGAGCTAACCAAAGTCCAGGTTCCCTGCACCACAAATCCGACGTCGGCGGGTT
TGAAAGATCTGAGCGACCAAATCGTGGAGGCTGAGCAGGCGGCATATGGGATGGTGGTGAATTCCTTTGA
AGAAATGGAACCAGAGTACGTGCAATACTACAAACAGGTAACCGGCAAAAAGGTGTGGTGCATCGGCCCT
GTTTCGCTATGTAACAAGCTTGACTCCGACAAAGCGGAAAGAGGCAAAAGGGCTTCGATTGAGGATACCG
ATTGCCTCAAATGGCTCGATTCGCACAACCCAGATTCCGTAATCTTCGCCTGCCTCGGAAGCCTCCACTA
CCTTTCGACTTCGCAATTAATAGAGCTGGGGCTCGCTCTCGAATCGTCAAATCGACCCTTCATTTGGGTC

ATCGGATTTGTGTCCGACGAACTAGGAAAGTGGTTATCGGAACAACGATTCGAGGAAAGGAACAAAGGAA
AAGGCTTGATAATCCACGGCTGGGCACCGCAGGTGCTTATTCTTTCGCACCCTTCGATTGGGGGTTTCTT
GACGCACTGCGGATGGAACTCCATGCTCGAAGGGCTGTCCGCCGGCGTTCCGATGATCACGTGGCCTCTC
CACTCGGAGCACTTCTACAACGAGAAACTGATTCTGCAAGTGCTCAGGGTCGGAGTTAGGGTCGGGGTGG
AGTTTCCGGTGCTGATCGGGGAGGCATTAAATGTTGGGTTTATTGTGAAGAAGGAAGAGATCGAGGCGGC
TGTGGAGAGGCTAGTGGGTGGAAAGGAAGCCGGAGAAATAAGAAAGAGGGTCGGAGAGCTGCAGGGGAAG
GCAAAGATGGCAATGAAAGACGGGGGTTCTTCTCATATGCGCCTGGAAATGTTCATCCAGGATATGGTGG
AACAATCTTCTTTGCAGAAAATATAA
>GWHPAAAL001759
ATGGAAAACAAGGAGTTGGTGTTCATCCCCATCCCGGCGATGGGTCACCTCATATCCATGTTAGAAACCG
CCAAGCTTCTCGTCAATCGTGACCAACGTCTCTCTGCAACGGTCCTCCTCTTCAAACTGCCCCAAGACGC
CGGCTTCGCTACCTATATGCAGTCACTTTCCTCTTCAGCTTTCGCCGCTCGCCTGAGATTAGTCATTCTA
CCGGTTCCCGAAAAACCACCGTCGGAGTCGTCCAAGCCTGATATCATAGAAAGCCACATGCCAAACGTCA
GGAACGCCGTCCGAGAAATCGGAGAATCCCGTGTGGCCGGTTTTGTCGTCGACATGGTCTGCTACAAAAT
GATCGACGTGGCCGACGAGTTTGGATTTCCGACTTACACTTACTTCACGTCCAGCGCCGGGTATCTGGGT
CTCCTATTCCACTTCCAGAGTCTCAAGGATCCCTACAATCTCGCTTCCAATTACAAGGATTGGGATTCCG
ATAAAAAGGAATTGTTGGTTCCGAGTTTCGTCAATCCCGTCCCAGAGAAAATCCTGCCGTTGGAGATGCT
CGACCACCAAGTTCCGCTAGTCCTCGAGCTCGCCCGGCGGCTCGTAGAACCCAAAGGGGTTTTGGTCAAC
ACATTCTCCGAGCTTGAATCTCACGCGCTTCATTCCCTCTCCGGCGGAAACAATCCGCCTGTTTATCCGA
TCGGACCCATATTACTCGCGTCGGAACCCCGCTATAATTGCCTGCAGGGCAACGAAGAAAGTATCATGAA
ATGGCTTGGAAAGCAGCTGCCCTCTTCCGTAGTGTTCTTGTGCTTCGGAAGCGCCGGAAGCTTCCCTGAG
GCGCAAATCAAAGAAATTGCGTACGCGCTTGAAAACAGCGGCCACCGGTTCTTGTGGTCCCTACGGCGAC
CACAGGCGGAAGGGCAGAGGTATCCAACCGAATACGACGACCCATCTGAAGTCCTGCCGGAAGGATTCTT
AGAACGGACGATCAATACAGGGAGGGTGATCGGATGGGCTCCACAGGTGGCGGTGCTGTCCCACCCGTCG
ATCGGAGGTTTCATCTCGCACTGCGGTTGGAATTCGTTGTTGGAGAGCTTGTGGTTCGGAGTGCCGGTGG
CAGCGTGGCCAATTTACGCGGAGCAGCATGTAAATGCTTTCCAGATGGTGAGGGAATTGGGGTTAGCGAT
GGAAATAAAGATGGATTACCGGAAGACAAATTCAGTGCTTGTGACGGCGGAGGAAATCGAGAGTGGTATA
AGGCGGCTCATGGCGGAGGAGTGTGGCGGCGGTGGTGGTGGAATTAGAGAGAAGTTGAGAGATATGAAGG
AGAATGGAAGGATGGCGGTGGCTGAAGGCGGCTCATCGTACATCTCACTCTCGCGTTTCATTGAGAATGT
TTTAGGCCCACACACTCAATAA
>GWHPAAAL001760
ATGGAAAACAAGGAGTTGGTGTTCATTCCCATGCCGGCGATGGGCCACCTCGTGTCCATGTTAGAAACCG
CGAAGCTTCTCATCAAACGTGACCAACGTCTCTCCGTAACGGTCCTAATCTTCAAACTTCCCTTCGACAC
CGGCATCGACACCTATATCCAGTCGCTTTCCTCTTCATCTTCCGCCACTCGCCTGAAATTAGTCAACCTC
CCAGACCCCCAAAAACCACCGTCGGAGGAGGAGGAGGAGATTCCCAAATCGTTCTTGTCTGATCTCATAG
ACAGCCACAAGCCACACGTCCGGAACGCCGTCCGAGATATCGGCGAATCCCGTGTAGCGGCATTCGTGGT
CGACATGGTCTGCTACAACATGATCGACGTCGCCGGCGAGTTCGGCCTTCCGACTTACACTTTCTTCCCG
TCCAGCGCCGGATTTCTGGGTCTCTTGTTCCACCTCCAGATTCTAATGGATCACCACAATCGGGACGTGT
CCGATTACAGGGATTGGGATTTAGATAAGGAATTGTCGGTCCCGAGTTACATCAATCCCGTTCCGGCGAA
AGTACTGCCGTTGGCGATGCTCGACAAACAAGAAGGTGGTGTCTCCCCAGTAGTCACGCTCGCCCGGAGG
CTACGAGAAACCAAAGGGATTTTGATCAACACATTCCCGGAGCTTGAATCTCACGCGCTTCATTACCTCT
TGAGTGGAAACAACCCCCCTGTTTATCCAATCGGACCCGTATTACTCGTACCTGAACGCAGCTTTAACGG
AGTGCACGGCTACAACGAGGGTATCATGATATGGCTGGAAAATCAGCGGCCCTCTTCCGTGCTGTTCTTG
TGCTTCGGAAGCGCGGGAAGCTTCAACGAGGCACAAATTAAGGAAATTGCCCACGCGCTTGAAAACAGCG

GCCACCGGTTCTTGTGGTCCCTACGGCGACCACCGCCAGAAGAGAAGAAAATTGAGCTACCGACCGACTA
CACCGACCCGTTGGAAGTCCTGCCGGAAGGGTTCTTAGAACGGACGGTCAATACAGGGAGACTTATCGGG
TGGGCTCCGCAGGTGGCGGTGCTTTCCCACCCGTCGGTCGGAGGTTTTATCTCGCACTGCGGTTGGAACT
CGGTGTTGGAGAGCTTGTGGTTCGGAGTGCCGATGGCAGCGTGGCCGAATTACGCGGAGCAGCAGATAAA
TGCTTTCCAGATGGTGAGGGATTTGGGATTGGCGGTGGACATAAAGATGGAGTACCGGAAGGAGAATCCA
GTGTTGGTGAAGGCGGAAGAAATCGAGAGTGGTATAAGGCGGCTCATGACGGAGGAGAGTGGTGGTGGAA
TTAGAGAGAAGTTGAGAGAGATGAAAGAGAAAGGAAGGATGGCGGTGGCGGAAGGCGGCTCATCCTACAC
CGCACTCGCGCAGTTCATTGAAAATGTTCTACGGCCTTCACAGATGATAGATCGTAAGTGGTGA
>GWHPAAAL001761
ATGGAAAATAATGAGTTGGTGTTCATCCCCATGCCGGTGATGGGTCACCTCCTATCCATGTTAGAAACCG
CGAAGCTTCTCATAAATCGTGACCAACGTCTCTCTGTAACGGTCCTCCTCTTCAAACTGCCCTTCGACAC
TGGCGTCGACAACTATATCCAGTCGCTTTCTTCTTCGGCTTCCACCTCTCGCCTGAGATTCATCAACCTC
CCGGACACCGAGAAACCCTCGTCGGAGGAGATTCCCAAAACGTTCATGATTGACCTCATAAAAAGCCTCA
AGCCACACGTCAGGAACGCCGTCCGAGAAATTGGCGAATCCCGTGTAGCCGGGTTCGTGGTTGATATGTT
TTGCCACGACATGATCGAAGTCGCCGAGGAGTTTGGTCTCCCGACGTACGCTTTCTTCACGTCAAGCGCA
GGGTTTCTGGGTCTGGTGTTCCACGTCCAGATTCTCAAGGACCACCACAATCAGGACATTTCCGATTACA
AGGATTGCGATAAGGAATTGTCTGTCCCCAGCTTCGTCAACCCCGTTCCGGCGAAAGTCCTGCCAACGAC
GATGCTTGAAAAAGAAGAAGGCGGTTTGTCGATCGTCCTATCACTTGCACGGAGGTTACGAGAAACCAAA
GGGATTTTGGTCAACACGTTCGCTGAGCTTGAATCCCACGCGTTTAATTCACTTTCGTGTGGAAACAACC
CGCCACATTATCCAATCGGACCCATATTACTCGAACCTGAACGCAGCGACGAGAGCGTGCAGGGATATGA
AGAGAGCATCATGAACTGGCTTGACGATCAGCCTCCGTCGTCCGTATTGTTCTTGTGCTTCGGAAGCATG
GGAAGCTTCAAAGAGGCGCAAATTAAAGAAATTGCCCACGCGCTTGAAAACAGCGGCCACCGGTTCTTGT
GGTCTCTGCGCCGGCCACCGCCGGAAGGGAAGAAAATAGGGTTTCCGACCGACTATGACGACCCGTCGGA
AGTCCTGCCGGAAGGGTTCATAGAGCGGACGGCCGATAACGGGAGAGTGATCGGGTGGGCCCCGCAGGTG
GCGGTGCTGTCCCACCCGTCCATCGGAGGTTTCGTCTCGCACTGCGGTTGGAATTCGACGTTGGAGAGCT
TGTGGTTTGGAGTGCCGATTGCGGCATGGCCGATTTACGCGGAGCAGCAGATAAACGCTTTCCAAATGGT
GAGGGATTTAGGGTTGGCGGTAGATATAAAGATGGAGTACCGGAAGGAGAATCCAGTGTTGGTGACGGCG
GAGGAAATCGAGAGTGGGATAAGGCGGCTCATGGCGGAGGAGAGTGGTGGTGGAATTAGAGAGAAGGTGA
GAGAGATGAAAGAGAAAGGAAGGATGGCGGTGGCGGAAGGCGGCTCATCCTACACCGCGCTCTCGCGTTT
CATTGAGAATGTTCTACAGCCACACACTTGA
>GWHPAAAL002229
ATGAAATCTGATGCAATAGTGCTCTACCCGGCTCCCAGCATTGGCCATTTGGTCTCCATGGTAGAGTTGG
GTAAGCTTATACTCAACCGTTACTCCCACAAGCTCGGATTCTCAATCATCGTCTTCCTCACAACCGGAAA
CCTTTCCGACAACGAAACCACCTCCTACATCCACCACGTCTCCAACACAAACCCATCCATCACCTTCCGC
CGCTTCCCCTTCGTCTCCGTCGATACCTCCCCCACACGCAGCCCTGCCGCCATTCACTTCGACTTCATTC
GCCTCAATGCCGACAATGTTCTCCACGCTATTCGAGAAATCCCCAATGTCATCCGCGCACTCGTTATAGA
TTTCTTTTGCGCCTCAGCAATCCCCATAGCCAGGGAGCTCAGACACCCTGTTTACTTTTTCTTCACGTCT
GGTGCTTATGCCCTAGCCTTCTATCTCTACTTCCCCACCGTCCACAAACAGGTGGACCAGAGCTTCAAGC
ACCTCAACAAAACGTTTCTTCAGTTCCCTGGATTTCCTCCAATTCGAGCTTCTCACGTGCCTGAACCGGT
GCTCGATCGAGACGATCCTGCTTATGCTGACATGGTATGCTTTTGCGATCATCTTCCCAAGTCTGACGGA
ATCATAGTGAACACATTTGACGCTCTTGAGCCAATGGCTTGCGAAGCAATCGCATCAGGTGCTTGCGTTC
CTGATGCAACTACTCCCCCAATTTACTGTATTGGGCCTTTGATAGCCAACACAAGTGAAGGTGGTGGTGA
AAATGGAAATGCCAGTCTCGGTAGGAGTAGTGAGCGTGAAGCCAAGCACGAGTGGTTGTCATGGCTTGAT
GCTCAGCCAAAAGGTAGCGTCGTCTTCTTGTGTTTCGGCAGTAGGGGTTCGTTCACTAGGAGGCAGCTTA

AAGAAATGGCTCATGGGTTAGAAAGAAGTGGACAGAGATTCATGTGGGTCTTAAAATCCCCACCTTGTGA
TGCTGCTGCTCCAGCAACCGAGTTCTGTTTGGAAGATGTGTTGCCCGAGGGGTTTCTGGAAAGAACAAAG
AAGACAGGAATGGTGGTGAGGTCATGGGTTCCACAAGTGGCAGTGCTGAATCATGGATCGGTGGGTGGGT
TTGTGACTCATTGCGGGTGGAATTCGGTGCTGGAAGCGGTGGTTGCGGGGGTGCCAATGGTGGCTTGGCC
GCTATATGCGGAGCAGCATGTGAACGGGGCCGCACTGGTGGAGAACATGAAGATGGCTATTCCTTTGGGA
CAAGAGAAAGAAGAAGACGATGATGGGTTTGTGTCGGCTGCTGAATTGGAGAAAGCACTTAGAGAGTTGA
TGGAGTCGGAGGGAGGAAGAGAGAGGAGCTTGAAGATGAGAGAGATGGCCATGGCAGCTTGGGAGAATAA
CGGTTCATCCACCACATCGCTAGCTAAGTTCGTCGAGTGGCTGGAGGGTTCTTAA
>GWHPAAAL003558
ATGGCTTTGATTCCAGCCAGCGACAAGCCCCACGCCGTGTGCATACCGTTGCCGGCACAGGGCCACATTA
ACCCAATGCTCAAGTTAGCAAAGATCCTCCATAGCCGAGGCTTCCACATCACCTTCGTCAACACCCACTT
CAACCGCAACCGCCTCCTCCGTTCACGTGGACCCTCCGCCCTAGACGGCCTGCCGGACTTCCGATTTGAC
TCCATCCCCGATGGCCTCCCGCCGTCTGACGCCGACGCCACCCAGGACATCCCTTCGCTGTGCGAGTCCA
CGCCGAAGCACTGTCTCGAACCCTTCTGCGACCTCATCACCAGAACCAATGAAGACGCTGGAGTACCTCC
AGTAAGCTGTATCGTCTCCGATGCGGTGATGAGTTTTACGCTGAAGGCGGCGGAGAGGTTTGGGTTACCG
GAAGTGCTGTTCTGGCCGACGAGCGCGTGTGGGCTGTTGGGGTATACACAATATCACAAGCTCGTTGAAA
GAGGATACACCCCACTCAAAGATATCAGTTACGTGACAAATGGGTATATGGAAACAACAATAGATTGGAT
CGCCGGAATGAAAGACATCCGGTTAAGGGATCTACCCAGTTTTATTCGAACCACCGACCCCAAAGACACC
ATGCTCAATTTCCTCATCAGCGAATGCGCCGCCATCCCCACCGCCTCCGCCCTGATCCTCAACACCTTCG
ACGCCTTGGAACGCGAATCCATCGACGCCCTCTCCGCCGTCCACCCCCGGATCTACACCGTCGGCCCTCT
CAACCTGATGCGAAATCAGATTAACGACGATCGAATCGAGTCAATCGGGTCGAATCTCTGGAAAGAGGAT
CCAGGCTGCATCGAGTGGCTGGATTCGAAGGAACCCGGGTCGGTAGTGTACGTCAATTTCGGTAGCATCA
CCGTGGTGACGACGGGTCAGCTCACTGAGTTCGCCTGGGGACTCGCCAACAGTGGCCGGCCCTTCCTGTG
GATACTCCGACCCGACATAGTCGCCGGAGATAAGGCGATGATTCCGCCGGAATTTCTGGCGGAGACAAAA
GGGAGGGGTATGCTGTCGAGTTGGTGCCTGCAGGAGCAGGTGCTGAGGCATCAGGCAGTGGGTGGATTCT
TAACCCACAGCGGGTGGAACTCCACCGTGGAGAGTCTATGCGGCGGCGTTCCGGTGATCTGCTGGCCCTT
CTTTGCAGAACAGCAGACGAACTGCCGGTACAGCTGTAGGGAATGGGGGGTGGGGATGGAGATAGATAAT
AATGTGAAGAGGGACGAAGTGGAGGCGACGGTGAGGGAGCTGATGGAGGGAGAGAAGGGGAAGGAAATGA
AAAGGAGGGCGGCGGAGTGGAAGAAGAAGGCGGAGGAGGCCACCGGAGCTGGTGGGTCTTCTCAGGTGAA
TCTTGAAAAACTATTTAGTGAGGTGCTCTTGGTAAACTCTGATGAGTGA
>GWHPAAAL004075
ATGTCCGACTCCGGCAACAAACGACCTCACGTCCTCGTCTTTCCTTACCCTGCGCCGGGACACATCATCC
CTCTCCTCGATCTCACCCAACTCTTGCTCTCCAGCGGCCTCACCGTGACCGTAATCGTCACCCCCGAGTT
TGTCTCTCTACTCGATCCCCTCCTTTCGTCTCACCCTTCCTCCTCTATCCAACCCCTCGTTCTCTCTCTA
CCGAAAGCCCCGGCTTCCTTGGTGCCTAGCCTTAGAGCCACAGGCGAGCTATACGATCCGATCCTCCAGT
GGTTTAAATCCCACCCTTCGCCGCCGGTGGCAATCGTGTCCGATTTCTTTCTGGGTTGGACCGAAAAACT
AGGGAAGGAACTCGGCGTGCCGCGTCTGGTTTTCTGGGCGTCTTGTGCTCTTTCTTCATTGATTTCCAGC
TCCATTTGGCGGGATCGGCCGAAGGTGGACGATCCGGCCGACGAGAATTTTCTTCTTTCCTTCCCGGAGA
TACCGAATTCTCCGAAATACCCCCTATGGCAGATGTCCACGCTTTTCCGTATGTTCAAGGAAGGAGACCC
AGACTGGGAATTCATCCGAGAAGGGATGGTGGCGAACACAGTGAGTTGGGGTGTCGTGTTCAACTCGTTC
ACTGAGTTGGAGGGGGTATACATCGATCACACAAAGAAAGAAATGGGCCACGATCGGGTTTGGGCTGTGG
GCCCGCTTTTACCACTAGACGGGGCCCCCACTTCCCGTGGTGGTTCTAGCGCCGTGGCGCCGCATAAGGT
GACGGTTTGGCTTGATGATAAACCGGATGACTCGGTGGTCTATATATGCTTCGGAAGCCGTATGGAGCTG
ACGACTGCGCAAAGGGACGCGCTGGCAGCTGCACTGGAGTCTAGCGGGGTGCACTTCATCTGGATCGTTA

AAGCAGGAATCGACGATGGAGACCCGATCCCGAACGGGTTTGAGGATCGTGTGGCGGGAAAAGGTTTAAT
AATCAGGGGGTGGGCCCCGCAGGTGCCGATACTGATGCACCGGGCGACCGGCGCGTTCGTGACTCACTGC
GGGTGGAACTCGGTGCTGGAAGGGATTTCCGCCGGGGTGATGATGCTGACGTGGCCAATGGGTGCCGACC
AATTCTACAATGCCAATTTGTTGGTTGACGAGTTACACGTGGCGGCTCGGGCTTGCGAAGGTGGGGATGA
GAAGGTTCCTGACTCGAGCGAGTTGGCTCGGTTGTTGGCCGAGTCGGTGAGTGGTAAGAGACTGGAGAGA
AGTCGAGTTGTGCAGCTGCGTGATGCGGCGGTGGAGGCGGTGAGGGGAGGAAGTTCAAGCAAAGACATCG
ATGATTTAGTTAAAGAGCTTGGTGAGCTTGGTTGTGGAAAAGCTTGA
>GWHPAAAL004076
ATGGCGATCTCCGGCTCCGGCAAACATGTCTTAATATTCCCGTACCCATCGCAGGGCCACCTGATCCCGC
TCCTCCACCTCGCCCACCGCCTTGCTGCCCGCGGCCTCACCATAACAATTTTAGTCTCCCCTCAAACCCT
CCCTCTCCTCCACCCTCTTCTCTCCGACCACCCTTCCATGGAAACCCTTGTATTGCCTTTCTCCACCCAC
CCTTCCATCCCAGCCGGAATCTACGACTGCAGGGGCCACCTCCCCGACTCCGTCCGCTCCACTTTGTTCG
CCATATCCGATCTCTACGAACCAGTCCTCGACTGGTTCCGAAACCACCCATCGCCGCCGGTGGCGATTCT
CTCCGACAAGTTCATGGGGTGGACCAAAATCTCGCATGCCAACTCGGAATTCATTGATTGGCGTTCTCCC
CTGTCGGCGCCATGGCCGTCTCATTGGTTTATTCCCATTGGCGAGATTTACCGGCGAGAAACATCGGCGG
CAGTGAAAATGACGCACTGGTTTCATTTCATGAGGAGCTACGTGGAAGGAGACCCAATTTCGGAATTCAT
CAAAGAAGGGATGCAGGCTAATATGGCGAGTTGGGGACTCGTGATCAATACTTTCAATGAACTGGAAGGG
GTTTATCTGGATCATCTAAAGGAGAAAATGGGTCACGATCGTGTTTGGGCGCAAATGGAGGCCATTGCAA
TCGGCCTAGAAAAAAGTGAGGCCTACTTCTTATGGTCCATTAGGGGTTCCACTGAAGGAGTTATCGACGG
CGAGTTCGGGGAGGTTCCCGCCGGATTCGAAGATCGGACGTCGGCGAGAGGACTCGTCGTGAAGGGGTGG
GCACCGCAGCTGTCGATATTGCGTCATCGAGCTGTGGGATCCTTCCTGACGCACAATGGGTGGAACTCTA
CGCTGGAAGCGCTGGTGGCTGGGGTGCCAATGCTGGCGTGGCCGATGGGAGCAGAGCAGTTCATGAATGC
GACCTTATTAGTGGATCAGGCGAAAGTGGCGGTAAGAGTGGCGGAGGGTGCACGGGATGTTCCGAATCCG
TTTGAGTTGGCTCGGGCGGTGACTCGAGTGATGGATGGCCGGAGCGTCGAGAGAGTTCAGGCGGTGAGGT
TGCGTGAAGAGGCATTGAATGCCGTCAAAGAAGGTGGGAGCTCATACCAGAATTTCGATGGGTTGGCCAT
GCATTTGTTCAACAATGAATAG
>GWHPAAAL005635
ATGGCAACGTTTCAATCGGAATCGTCTCCTCATATTCTTCTGTTTCCGTTCATGTCAAAGGGCCACACCA
TCCCAATCCTCCACCTGGCTCGCCTTCTCATCCACCGCAACGCCGCCGTGACCATCTTCACCACCCCCGC
CAACCGCCCCTTCGTTTGTCACTCTCTCTCCGACACCACTGCGACCGTAGTTGACCTCCCGTTTCCGGAA
AATATCCCCGGAGTTCTCCCAGGCGTGGAGAGCACCGACAAACTCCCACGGCTTCCTTGGCTGGACCGTG
GAATCGGCGGCCGAATTCGGCATCCCGCGGCCCGCTATTACGGTATGAATAACTTCTCCGGAGCTATTTG
TCACGACGTGATGACGCATCGTGACCTCATCGAACCCGGGTCCGGCGATGAACTGTTCCCGGTCCCCGGT
TTTCCATGGATAAAGGTCGCCGGAAACGATTTCGAACCGGCGCTGTCAGCTTCCCCTCCTGCTGGCGATG
GCTATGGCCTCCAATGGGAGTTTACGATTGAGGTGGTAGAAGCAACTTCCAAGAGCTACGGTGTTGTCGT
GAACAGCTTTTACGAGCTCGAGCCGGCCTACTTGAATTACTGGAATTCCAATATTAGGCCCAAGGCCTGG
TGCGTGGGCCCGCTCTGCCTGGCGGAGCGGCCGCGTGTCAAACCGGAGAACCACCGGAAACCGTCGTGGA
TCCAGTGGCTGGACGAAAAGCTGGAAGAAGGCAAACCTGTTCTCTACGTGGCGTTCGGTTCCCAAGCGGA
GATCTCGCCGGAACAATTCCGAGAGATCAAAACCGGGTTGGAGAAATCCGAATCGAATTTCTTGTGGGTC
GTCAGAGGAGGCCTGTCGGAACTCATCGACGACGATGGGTTCGAGGAGAGAGTGAGAGAAAGGGGTCTGG
TAGTGAGAGAGTGGGTCGATCAGAGGGAGATATTGGGACATGGAAGCGTAAAAGGGTTTCTGAGTCACTG
CGGGTGGAACTCGGCGTTAGAGAGCATATGCGCGGCGGTGCCGATTCTTGCGTGGCCGATGATGGCGGAG
CAGCCGCTGAACGCGAGGATGGTTGTGGATGAGATAAAGGTGGGGCTGAGGGTGGAGGCGTGCGATGGGT
CGGTGAGGGGGTTCGTGAAGTGGGAGAGTTTGGAGAAGATGGTGAGGGAATTGATGGAGGGAGAGATGGG

GAAGGAGGTGAGGAAGAAGGTGAAGGAGGTGGGAGCGGCGGCGGTGGCGGCGGTTCGAGGAGGTGGGTCG
TCGTGGAAGACGTTAAATGAGCTCATTAATGAGTTATCGTGA
>GWHPAAAL005636
ATGGCAACGTTTCAATCGGAATCGTCTCCTCATATTCTTCTGTTTCCGTTCATGTCAAAGGGCCACACCA
TCCCAATCCTCCACCTGGCTCGCCTTCTCATCCACCGCAACGCCGCCGTGACCATCTTCACCACCCCCGC
CAACCGCCCCTTCGTTTGTCACTCTCTCTCCGACACCACTGCGACCGTAGTTGACCTCCCGTTTCCGGAA
AATATCCCCGGAGTTCTCCCAGGCGTGGAGAGCACCGACAAACTCCCACGGCTTCCTTGGCTGGACCGTG
GAATCGGCGGCCGAATTCGGCATCCCGCGGCCCGCTATTACGGTATGAATAACTTCTCCGGAGCTATTTG
TCACGACGTGATGACGCATCGTGACCTCATCGAACCCGGGTCCGGCGATGAACTGTTCCCGGTCCCCGGT
TTTCCATGGATAAAGGTCGCCGGAAACGATTTCGAACCGGCGCTGTCAGCTTCCCCTCCTGCTGGCGATG
GCTATGGCCTCCAATGGGAGTTTACGATTGAGGTGGTAGAAGCAACTTCCAAGAGCTACGGTGTTGTCGT
GAACAGCTTTTACGAGCTCGAGCCGGCCTACTTGAATTACTGGAATTCCAATATTAGGCCCAAGGCCTGG
TGCGTGGGCCCGCTCTGCCTGGCGGAGCGGCCGCGTGTCAAACCGGAGAACCACCGGAAACCGTCGTGGA
TCCAGTGGCTGGACGAAAAGCTGGAAGAAGGCAAACCTGTTCTCTACGTGGCGTTCGGTTCCCAAGCGGA
GATCTCGCCGGAACAATTCCGAGAGATCAAAACCGGGTTGGAGAAATCCGAATCGAATTTCTTGTGGGTC
GTCAGAGGAGGCCTGTCGGAACTCATCGACGACGATGGGTTCGAGGAGAGAGTGAGAGAAAGGGGTCTGG
TAGTGAGAGAGTGGGTCGATCAGAGGGAGATATTGGGACATGGAAGCGTAAAAGGGTTTCTGAGTCACTG
CGGGTGGAACTCGGCGTTAGAGAGCATATGCGCGGCGGTGCCGATTCTTGCGTGGCCGATGATGGCGGAG
CAGCCGCTGAACGCGAGGATGGTTGTGGATGAGATAAAGGTGGGGCTGAGGGTGGAGGCGTGCGATGGGT
CGGTGAGGGGGTTCGTGAAGTGGGAGAGTTTGGAGAAGATGGTGAGGGAATTGATGGAGGGAGAGATGGG
GAAGGAGGTGAGGAAGAAGGTGAAGGAGGTGGGAGCGGCGGCGGTGGCGGCGGTTCGAGGAGGTGGGTCG
TCGTGGAAGACGTTAAATGAGCTCATTAATGAGTTATCGTGA
>GWHPAAAL005637
ATGGCTTATCCTCCACCTTCTCCCGCACTGCACATTGCTCTCTTCCCCTTCATGTCCAAAGGCCACACCA
TTCCGATCATCCACCTAACAAGACTCCTCCTGAACCGCGGCCATAAAACCACCGTTTTCACCACCCCAGC
AAACCGCCCCTTCATCTCTGACTCCCTCACCGGCACCGACGCCTCCGTCATTGTTCTCCCCTTCCCGGAA
AACATCCCAGGAATACCGTCCGGCACCGAAAGCACCGACAAACTACCGTCAATGTCCCTCTTCGTCCAGT
TCGCCAACGCCACAAAACTCATCCAACCTCACTTCGAGCAAGCCATCAAGTCACTCCCCAACCTCAACTG
CATGATATCCGATGGCTTCCTCAGCTGGACCCTCCAATCGGCATCTAAATTCGGCATCCCAAGGCTAGTC
TTCTACGGTATGAACAACTACTCCATGGCACTAAACTACGACGTGACCGTCAATAGGCTCCTCTCGATAC
CCAAATCGGACAACGAGCCATTCGTCGTCCCTAACTTCCCCTGGATCAAACTCACCAAGAATGACTTCGA
AGAGCCCTTTAACGAGGTTGAACCAAAGGGACCTTATGTGGAATTCACCATCGAACAGAGTCTAGCCACG
TTTAATAGCTACGGTCTGATCGTGAACAGCTTCTTCGAGCTCGAGAAGCCGTTCGTGGAGTACTGGAATC
GCGCGAGCAAACCGAAAGCTTGGTGCATTGGGCCTCTCTGTTTCGCAGAACCGGAAATGATCATCGAAAG
AAAGCGCAAACCAAAGCCCAAGTGGATCGATTGGCTGGACCAAAAGCGAGACCAGGGAAAATCCGTTCTG
TACGTTGCGTTTGGGTCTCAGGCGGAAATTTCACAGAAGCAGCTGCATGAAATAGCAATGGGGTTAGAGA
AATCGGAGGTGAACTTTCTATGGGTGATGAGAAAGAAAGACACGGCCATTGAGGGAGTGCCGGAGAGAGG
AATGGTAGTGAGAGACTGGGTTGATCAGAGGGAGGTGTTGGCACACGAGGGGGTGCGGGGGTTTCTGACG
CACTGCGGTTGGAACTCGGTGTTGGAGAGCATATGGGCAAGGGTTCCGATACTGGCGTGGCCGATGATAG
CGGAGCAGCACCTGAACGCGAGGATGGTGGTGGAGGAGATCAAAATCGGATTAAGAGTGGAGACGATTGA
CGGCTCTGTAAGAGGGTTTGTGAAGAGGGAAAGCTTGGAGAAGATGGTGAGGGAGCTGATGGAGGGGGAT
ATGGGGAAGGAGGTGAGGAAGAAGGTGGAGGAGGTCGGAGAGGCGGCCGGAAAAGCAATGACAGAGGGTG
GGTCCTCCTGGGATGCCCTGAATCAGCTTATTGATGAGATAAAAAGCACATAG
>GWHPAAAL005638

ATGGCTTCTCCTCCACGTTCTCCCACACCACACATAGCTCTCTTCCCTCTCATGTCCAAAGGCCACACCA
TTCCCATTATCCACCTAACAAGGCTCTTCCTGAACCACGGCTTTAAAACCACCGTTTTCACCACTCCAAC
AAACCGCCCCTTCATCTCCGACTCCCTCGCCAGCACCGATGCTTCCGTCGTCATTCTCCCCTTCCCAGAA
AACATTCCTAGAATACCCTCCGGCACACAAAGCACCAACAAACTATCGTCAATAGGAAGCCATCAAGTCT
CTCCCCAATCTCGTCTGCATGATATCGGACGGCTTCCCCATAAAAACGACTTTGACGAGCCCTTCAACGA
GATCGAACCAAAGGGACCTTATGCGAAATTCATCATGGAACAGGGTGTAGCCGACTCTAACAGCTATGGT
ATGATCGTGAACAGCTTCTACGAGCTCGAGAGGCCGTTTGTGGAGTACTGGAATCGTGAGGGCAAACTGA
AAGCTTGGTGCGTTGGGCCTCTCTGTTTTGCCGAACCGGAGATGATCATCAAAAGACAGAGCAACCCTAA
GCCCAAATGGATCGATTGGCTGGACAAAAAGCGAGACCAGGGAAAATCGGTTTTGTACGTTGCGTTTGGG
TCTCAAGCGGAAATTTCACCGGAGCAGCTGCATAAAATAGTGATGGGGTTAGAGAAATTGGAGGTGAACT
TTTTATGGGTGAAGAGAATGAAAGATACATATATTGTGGGAATGACGGAGAGAGGAATGGTAGTGAGAGT
CTGGGTTGATCGGAGGGAGGCGCTGGCGCACGAGGGGGTGCGGGGGTTTCTGACGCACTGCGGTTGGAAC
TCAGTGTTGGAGAGCATATGGGCAAGGGTTCTGATACTGGTGTGGCCGATGATGGCAGAGCAGCAGCTGA
ACACAAGGATGGTAGCGCAGGAGATCAAAATCGGGTTAAGAGTGGAGATGATTGACGGCTCCCTAAGAGG
GTTTGTGAAGAGGGAAAGCTTGGAGAAGATGGTGAGGGAGCTGATGGAGGGGGATATGGGGAAGGAGGTG
AGGAAGAAGGTGGAGGAGGTCGGACGGGTCCTCTTGGGATGCTCTGAATCAGTTTATTGA
>GWHPAAAL006268
ATGACTCGCAAAGAAGTGCATGTCCTCGTGGTCACGCTACCATTCCAGGGCCACATCAACCCCATGCTCC
GCCTCGCCAAGCGCCTCGTCTCCAAAGGCCTTTACGTCACCTTCGCCACCACCGAGACCGCCCGTCAGCG
GCTACTCGGCGGCAGTTCCGCTGCCACCTCTATCACCAGGTTCCAATTTGAGTTCTTCAAAGACGGTCTC
GGTCCGGAAGTGGACCGCGACAAGAACCTAGACATCTTACTGCTCGAAGCTCTGCCCACCAAAGGTGTCG
AGAATTTCTCGAATCTCATCGCCGATTTGAGCAAAAGGGGGAGAAAATTCTCGTGCGTCATCAACCAGCC
TTTCGTCCCCTGGTCGGCGGCCGTGGCGGCGGAGCACGGCATCCCCACCGCCACGCTTTTCATTCAGCCT
TTCTCTCTCTTCTCGATTTATTACCGTTATCACAAGGGATTAAACTTGTTTCCGTCGTTGGAAAATCCCA
ACAACACCCTGAAATTGCCGGGATTGCCCCTTATGAAAGTAAGTGATCTTCCGACGTTTGTTCGCCCGGA
GAGCCCTTACCATTTCCGGAAACTTTTGGCTGAATCTGTGAAGAGTCTCGAGAAGGTGAAATGGGTTTTG
GGGAATTCATTCCAGGATTTGGAGAAAGGGGTGTTGGAGGCCATGGAAGAAGATTCCATTAACAATTGGC
TTCCCGTGGGTCCCTTTTTGGCGTCGGCGTTGCTCGGAGAAAAAGAGGAAGGGAATGTGGCAGTGGGTGG
GGCCGACATGTGGAAAGCCGAAGACTCGTGTCTGGAGTGGCTCGACGGCAAGCCGGTTTCATCGGTGGTT
TACGTGGCGTTCGGGAGCATAACGGTTTTGGACGGGAAGCAGATGGAGAGCATGGCGGCAGCGCTCAAGA
ACTCCGGCAAGCCGTTTCTGTGGGTGGTCAGGTCTCCGGCGAAAGATTCTGATGAGAAGAAGAGCAACGG
ACTTCCTGAGGGATTTATTGAAGAGATGAAAGGTAGGGGACTGGTGGTGCCGTGGTGCCACCAGGAGAAG
GTGCTGATGCACCCGGCGGTGGCGTGCTTCGTGAGCCACTGCGGCTGGAACTCCAGTTTGGAGGCGGTGG
TCGCCGGAGTTCCCGTGATCGCATTTCCGGAGTGGACCGATCAGCCGACCAATGCACAGATTGGGGTGAG
ATTGGGTGAGAACGACCGTGACTTTAGCAGTGAGGAGGTGGAAAGATGCATTCGTGCTGTCACTGACGGG
CCGGGAGCGGCGGAGATCAAGAGAAGGGCGGCGGAGCTGAAAGAAGCGGCCAGGAAGGCGGTGGCTGACG
GTGGTTCTTCGGAACGGAACCTGAATCGGTTTATTAGTGAAATCAGCGCCTAG
>GWHPAAAL006282
CGGCACCAGCGCAGGCGGGTGGTTCTATTTCCGTTACCGCTGCAGGGCCATATACCCATGGTTCAACTCG
CCAACATATTATACTCCAAAGGCTTCCAAATCTCCATAGTTCACACCCACTTCAATTGCCCCAGCCGATT
GAAATACCCCCACTTCACCTTCCACCAAATCTCCGACGGCCTGTCGGAAACTGAAGCTTCCACAGAGGAT
ATCATCGCTCTTGTCTCCCTCCTCAATAAGAAATGTGTCGGCCCGTTTCGCGATTGCTTGATTCAGTTAT
TATCGGACGACTCTTCGGATCCCATTGCCTGCTTGATCACCGACGCAATTTGGCACTTCAGTCAAGCTGT
GGCGGAGGGACTTAAGCTCCGGCGACTTGTTCTCCGGACGAGTAGCGTCTGTTCTTTTCTCGCTTTTGCG

GCCTTGAATCTTATGAGAGACAAAGGGTACCTCCCAAAGCAAGATTCCCGATTGGAAGAAATTGTTCCAA
AGCTTCCGCTACTCAAAATGAAAGACCTTTCATTCATCAAAACCAAAAACCCAGAAGATCTCTACCATTC
AGTAGGAGGCATGATAACCAACACTAGGGAATCTTCAGGGATCATCCTAAATTCTTTCAAAGAATTGGAA
GAATCTTCACTTTCGATTCTCAAGCATGATTTTCCGATCCCAAATTTCCCAATTGGATCTTTCCACAAAT
ATTTTTCAGCAACCTCGAGCAGCCTACTAGAACAAGACCGAAGATCTATCTCTTGGCTCGACACCCAAAA
ACCCAATTCAGTAATTTATGTGAGTTTCAGGAGCATTGCAACAATAGAAGAAACAGAATTCCTCGAAATT
GCTCACGGATTGGTCAACAGCAACCAACCTTTCCTATGGGTAGTTCAACTGAAATTAGTCCGCAGGTCGG
ATTGGGTCGAACTGTTACCTGACGGGTTAATAGAATGTATTAATCGGAGGGAACACATAGTTAAATGGGC
CCCACAACAGGAAGTACTGGCCCATCCTACTACAAGAGGATTCTGGACTCACTGCGGGTGGAATTCAACT
CTGGAGAGCATATGCGAAGGCGTTCCAATGATTTGTTCGCCGTGTTTTGGCGATCAATTTGTGAACTCAA
GGTATATAAGTGATGTTTGGAAGATTGGGGTGGAATTGAAGAATTTGGAGAAAGAAGACATCAAAAGGGC
TATTAGGAGAGTAATGGTGGAGAAAGAATGGGAAGACATGAGATTCAGAATCATGGAACTCAAAGAGAAG
GTAGATTGCAGTATAAAAAGAGGATCCTCAACAGAATCATTACAAAGTTTAATTAATTACATTTTGTCAT
TG
>GWHPAAAL006284
ATGGATCAGGAAACGACTCTACCTCCTCATGTCCTCATCTTCCCTCTACCGTTGCAAGGCCCAGTGAACT
CCATGTTAAAGCTAGCCGAGCTCCTCTCCATCTCCTCCCGTTTCCACATCACCTTCCTCGTCACCGACCA
CATCCATCGCCGCCTCCTTCAGCACACCGATATTAAATCCCGATTCGAAAAATACCCCGGTTTCCGCCTG
GAAACCATCTCCGATGGCCTTTCCGAAGAACACCCTCGCTCTGGAGACCGACTCATGGAGCTGTTCGATT
CTCTGAATACCGAAACTAAGAAGCTTTTCAGGGAGTTGCTGAACTCCGGCCGCCTGATCTCCGGTAATTG
CCGTCCCATAACTTGTATTATAGCCGATGGGATCATGGGTTTTACCTGTGATGTCGCGAACGAGTTCCGA
ATTCCGATTATTTATGCACGCACGGTCAGCGCTTGCTGTCTCTGGGTTTTTTTCTGTCTTCCCAAACTCA
TTGAAGTCGGAGAGATTCCCTTCCAAGGAAACGATCTGGACGTGCCAATTAGGAGTGTGCCTGGGATGGA
AGGCTATCTCCGGCGCCGCGATCTTCCATCTTTTTGTCGCTCCGGCAACGTGGCCGGCCCAAATGTCCAG
GTTTACTTGTCGGAGTCGCAAGAGAATCCCCGAGCCCATGGGCTCATTCTCAATACATTTGAAGATTTGG
AAGGTCCGATTCTCTCTCAAATCCGGACCCTCTGTCCAAATCTGTACACAATCGGACCGTTGCACGCCCA
CCTGAAGACAAAGCTCGCCGCCTTAAGATCTCTGGCGCCGGCATCTTCGAACAGCCTCTGGGAAGAAGAT
CGGAGCTGCTTGGCGTGGCTTGATGTCCAGCCGCCGAAGTCCGTTGTCTACGTCAGCTTCGGAAGCCTAG
CGGTAATGTCAAAAGACCAGCTCCTGGAATTCCGGCACGGGCTAGTCAACAGCGGCCAAAGATTCCTCTG
GGTTATAAGGCCGGACTCCGGCGCAGGCGAAGGCCTGATTCCGGCGGACACAAATAATCGTGGGTACATA
GTAGGATGGGCTCCACAGGAAGAGGTTCTATCGCATGCATCTGTTGGTGCGTTTCTGACTCACAGTGGGT
GGAATTCAACTTTAGAAGCAATCTACGAAGGCAAACCCATGATCTGTTGGCCTTATTTTTTGGACCAGCA
GGTGAACAGTAGGTTCGTGGAGAAAGTTTGGGGACTGGGGGTTGACATGAAAGATATTTGTGATAGAGTT
GTAATTGAGAAAATGGTGAGAGATCTTATGGATGAGAGAGGAGGTGGGTACAAACAAAATGCCGATCAGA
TTGCTAAATTGGCTAATGAATGCTTCAAAGCGGGTGGTTCATCGTACAAAAGCTTGGAGAATTTGGTTAA
GGATATAGAACTAATGATGAAATCTTATTGTGCTGAATTTGGTCACTGTGTGAAAGTGAGAACAAGAAAA
AACCAGCCGCACAAGAGAGAGCGAGAGCGAGAGGAAGAGAGAGTTGTCTTGAAACCTTGTGCATTGTTTC
GGTCAAATGCCGATTGGAGGACGATCCACCGTTTGATCAAGCTAAAATTTGGACAGCAGGTTCGCAACTC
AGTGCTCTACAATCTGAACGGTGGAAATCGGATTCTGGGGTCAGCAACTACTGTTTCGGCGGGACTGTTT
TGGGTTGTGTTTAAACCCGCGTTTTGGTGA
>GWHPAAAL007266
ATGGCGTCCAAAACCGATCGTCTTCAGTTCCTCTTGATTCCTCTTATGTCCCAGAGCCATCTAATTCCAT
TAACCGACTTCGCCAAACTTGTCGCTCGCCGCGGAATCTCTGTCACGATCATCACCACTCCCCTCAACGT
CCACCGCTTCAAGTCCATCATTGGCCGGACGCCGAACGCCGACCTCAAAATCCATCTGCTCTCGATTCCA

TTCCCCTGCAAGGAGGCCGGATTGCCGGAAGGCGTAGAGAACGTCGACTCTCTCGATTCTCCAGAATCTG
CCGGCAAATTCTTCTTAGCCTCTAGTTTGCTCAAATATCCGCTTGAGAAACTCGTTTCGGAAATGGACGC
CAAGCCTAATTGCATCGTCTCGAGCAATGCTTTGCCCTGGACGGTGAAATTAGGGGAGAAATTTGGAATT
CCAACCTACTTCTTCAATCCGATCTCGTGCTTCACGGTTGTCTGCTCGACGAACATTGCCATCGGAAAGC
CGCACGAGGCAGTGTCGTCGGATTCGGAGCCTTTTGCGGTCCCAGACATGCCTCACCGGATCGAGTTCAC
GAGGGCGCAGCTGCCGGAAAACATGAGGAAGGGAGACTCCACCGACGACGTGAAGGCCGTAATGCAACAG
ATTCGTGACGCGGTGAGCTCCGCCCGCGGGGTCCTGGTTAACAGCTTCGAGGAACTGGAGGCGCCATACA
TCCAATCCTACAAGAAAACGGTGAAGAACCTGTGGTGCGTCGGCCCAGTTTCCCTCTCCAACGACGAATC
GCCGCCCTCGTCAACCACTGATGAAAGAATCGACTGCCTGAAATGGCTCGATTCAATGAACCCTAGCTCC
GTCATCTACGCCTGCTTCGGAAGCATCTCCCGCATACCGGCTTCCCAACTGATCGAGATTGGATTAGGTC
TAGAAGCTTCTAGAACTCCGTTCCTCTGGATCATCAAAGAGAAAGACATCTCCGAGCCGGTGCAGAAGTG
GTTAGACGACGAGAAATTCGAAGAGAGAATCAGAGGGAGGGGCTTACTGATCAAGACTTGGGCCCCACAG
GTACAGATACTGGCCCACCCCGCCGTCGGTGGGTTCCTTACACACTGCGGATGGAACTCCACCTTAGAGG
GGGTCTGCGCTGGACTTCCGATGATCACGTGGCCGATGATCGCCGAGCAATTCTACAACGAGAAGTTTAT
TGTGAATGTTCTTGGCGTTGGCGTGAGGATAGGGGCGGAGGGCGGCATGGAAAGCGGTGCATTCGTGGGG
CGTGAGGAAATAAAGAAGGCGGTGGTGGAGTTGATGGGTGGGGAGGAGGAGAGAGAAGGGGGAGGAGATA
GAAGGAGGAAGGCGAGGGAGTTGGGGGAGATGGCGAAGAGGGCTGTGGCGGACGGCGGATCTTCTCACCT
GAATATCTCGTTTCTAATTCAAGATGTCATGCAACAAAGTTGA
>GWHPAAAL007326
ATGGGTAGCCAAAAACCTCATGTTCTGGTAGTCCCATTTCCAGCACAAGGTCACTTTAGTCCAGCGCTGC
AACTCTCAAAGCGCTTAGCCTCAAAAGGGGTTAGGGTCACTTTCGTCACCACCATGAGTTGGGCCAAGTC
CATTGGTTCCATTAGGATGGAGCAAATTAGCGATGGCTATGAAGAACGTGAAAACCCAGAGAGCATTGAA
GCGTTTGCCAAGCGTATCAAAACCGTAATCTCGCAAAGCCTACGTGATCTAATTGAGAAACAAAAACAAG
TTGGTTACCCTGTGAAACTAGTTGTTTACGATTCTGCTATGCCGTGGGCACTAGAGATAGCACATAAGTG
GGGCGTCTATGGAGCTCCATACTTCACTCAACACTGCACTGTATGTGCTATCTACTACCATATGCAACAA
GGAAATCTGAAGGTTCCTCTTGAAGGAACAAAAGTCTCCCTACCTTCATTGCCGCCATTAGAGAAAAATG
ATTTGCCATCTTTCATTTGTAACTTGTTCCCACACGAATTTCTCATGAAAGAGATGTTGAACCAATTCTC
CAATTTCCACAATGCAGATTGGATCTTATTCAACACATTTGACAAGCTAGAAGATAAGGTAGTGAAGTGG
ATGGCAAGCCAGTGGCCTATTAGAACCGTTGGACCTACTATTCCATCCATGTATTTAGACAAGCGACTGG
AGGGAGACAAAGAATATGGCCTCAGCCTCTTCACTCCAAACACCGAGGCTAGCATGAAGTGGCTGGACAC
GAAAGAACAAGGTTCGGTGGTTTATGTCTCATTTGGGAGCTTGGCCACCCTTGATGAAACTCAAATGGAG
GAATTATCATGGGGCCTTGTGCAGAGCAACAACTACTTCCTATGGGTGGTAAGAACTTCTGAAGAGAGCA
AGCTTCCTAGCAATTTCATGGACAAGACCTTTGAGAAAGGGTTGGTGGTCAATTGGTGCCCTCAGCTTGA
GGTCTTGGCTCACCAGGCGGTGGGATGTTTTGTGACTCACTGTGGTTGGAACTCGACGCTTGAAGCTTTG
TGCCTTGGGGTGCCAATGGTTGCTATGCCCCAGTGGGTTGATCAAACAACTAATGCGAAGTTTGTGGCCG
ATGTGTGGAGGGTAGGGGTTCGAGTGGAGGCTAATGATAAGGGAATTGTTACGAAAGAAGAGATTGAAAA
GCGAATCAGAGAAGTCATGGCGGGAGAAACGAGGGAAGAACTGAGAAAGAATGCTAGTGGTTGGAAAAAC
CTAGCTAGAGAGGCAGTGGATGAAGGTGGGAGTTCTGATAAGAATATTGAGGAATTTGTTTCTCAACTTG
TATGCTCTTAA
>GWHPAAAL007393
ATGGCCGACGACTCTAAGCTACACTTTGTGATGTTCCCATGGCTAGCCTTTGGTCACATTATTCCATTCC
TGGAGCTCTCCAAATTCCTAGCTCAAAAGGGTCACAAAATCTCTTTTATTTCCTCCCCAAGAAACATCGA
TCGCCTCCCCAAAATCCCCACAAATCTCCCAATTAAATTGGTAAAGATTCCATTGCCGCCGATCGAAGAG
CTCCCGGAAAACGCAGAGGCCACCATGGACGTCCGCACGGACGATATTCACTACCTCAAGAAGGCATACG

ATGGTCTCGAATCCGGTTTGACTCGTTTTCTCGAGTCAGAGTCCCCGGACTGGATCGTTATGGACTTTGC
TCCCCACTGGTTGCCGCCGATCGCTGCGAAGTTGGGTATCTCGCGTGTCTTCTTCTCGATCATCAACGCG
TGGTTCTTGGTCTTTCTTGGGGCCTCATCGGAGGCCATGATCAACGGCTCCGATGAGCGGACAGCGCCGG
AGGATTTCACGGTCCCGCCCAAGTGGGTACCCTTCCCGACGAAGGCGGCCTACAAGCTCTACGAGGCCAA
TTGGGCTTTCCAGTCCACCAAACTCAACGCTTCGGGATTCACCGATATGTATAGATGTGGATCTGTTATT
CAGGGGATCGACGCCATCCTCGTACGCCACTGCAATGAGTTCGAACCCGAATGGCTGACCCTCCTGGAAA
ACCTTCACGGCCGGCGGGTAATTCCGGTGGGTTTAATGCCACCTCGTCGGCAAAATAACCAGGACGACAC
AAACGAGACGTGGGTTTCAATCAGAGACTGGCTGGATTCGCAAAACAGATGCTCCGTCGTTTATGTGGCA
CTGGGTAGCGAAGTAGCACTGAGTCAAGAACAACTCAGTGAGTTAGCTCTAGGTCTAGAGCTATCCGGTT
TACCCTTCTTTTGGGCCCTAAGGATACCGCGGTCCAACCCGGTCGATCTTCCCGATGGGTTCGAGGAGAG
AATGAAATCGAAAAGGCAAGGAATGGTCTGGAAGGCTTGGGTACCTCAGGTGAAGATACTGAGTCATGAC
TCGGTGGGTGGGTTTTTGACACACTGCGGTTGGAGTTCCATAATAGAGGGGCTGATGTTTGGGAAACCGC
TTATTATGCTGCCATTTTTGGTGGATCAAGGGTTGAATGCTAGGCTACTGGCGGCGAAAGAGGCGGGCGT
GGAGGTTGACCGAGACGAAGAAAGCGGCGCGTTTACGAAGGAAACGGTGGCCGAGGCGTTGAGGTTGGTG
ATGGTGGAAAGCGAGAGGGGAAAGGTTTTGAACAGAGGAAAAGCCAAAGAAATGGGTGTCGTTTTCGGTG
ACAAGGATTTGCATGATCTGTACCTGGAAAACTTGGTGGTGTATTTTGAAAACCATAGACGTTTGTGCAA
GGAAAGTTGA
>GWHPAAAL007798
ATGGCGAGCCAAAAGAACGACAACGGAGCTCACGTACTGGCCATCCCATGGCCGGCCCCGGGTCACATGA
ACCCACTCCTCCAATTCTGCAAGCGTCTCGTCTTCCGAGGAGGCATCAAGGCCACTTTCGCCATCACCAA
GTTCCAATCCAAGTCCATGCACTCCCGCCTCGACTCCATTCAGATCGACACCATCTCCGACGGATACGAC
GACGGCGGTTTCTTCATCACCGCTGGCATCCAGGAATCCGTCGAGCAATTCAAGGCCGTCGGATCCAAAT
CCCTCGCCGAGCTCATCAAGAAACACGAGTCCATGGGCCAACCCTTCGACTGCATAATCTACGACGCCTT
CGTGCCCTGGGTTCTGGAGGTGGCCAAAGAACACGGCATAGCTGGGGCGGCGTTTTTCACACAAACCAGC
GCCGTGAATTGCGTGAACTATCACGTTTATCACAAATTGATACCCTACCCGTTCGACTCGCCACCTTATT
CTCTTCCCGGCTTGCCGCCGCTTGGGCTGCAGGACATGCCCGGGTTCATGACTGTGGATCCATATGTATA
TGAGCTTGTTTTGAGTCAATTTTCTACCCCTCAACAAGCGGATTATCTCTTTGTCAACACTTTCTATCAC
TTGGAAGCAGAGGCAGTGGAAGCAATGTTGAAGGTACATCCATTGCTGAAGACAATTGGACCAACAGTTC
CATCATACTACCTGGACAACCGAATCGAGAACGACAAAAGTTACGGTCTCCACGCCACCCACTTGATGTT
CCAAGAGAAGAATTCCTCCCAAACCGACCCTTCCCTCGTCCTCGATTGGCTAAACACCAAGCCCCACGCC
TCCGTCGTCTATGTATCGTTCGGCAGCATCGCATTGGCCAGTCCCAGCACCGACGAAATGAAGGAAATCG
CCTGGGGTTTGAAGAACACCGGCTGCTATTTCCTCTGGGTGGTGAAGGCCAACGAAGAGCCGAACCTCCC
AAAAAATTACAGGGAAGCGACGAGGGAGAAAGGGCTGATCGTGTCGTGGAGCCCGCAATTGGAGGTCCTG
GAACACGAGGCGTTGGGATGCTTCGTCACCCACTGCGGCTGGAATTCGACGATTGAGGCGCTGTGCCTGG
GAGTGCCGATGGTGGCGATGCCGAAATGGGCGGATCAGCCGACGAACGGGAAGTATGTTCAGGACGTGTG
GAAGATTGGGGAGAGAGTGAAGGTGGATGAAAATGGGGTCGCCGGAAAAGAAGAGGTGGAGAGGTGTATA
CGGGCGGTAATGGAGGGAGAGAGCGGGAAGGAGTTCAAGAAGAATGCTGCGAAATGGAAGGAGTTGGCTA
AGGAAGCGGTCAATGAGGGTGGGACTCAGGATAAGAACATTGACGAATTTGCTTCCAAATTGATAAGCTC
TTCCCACCGATCTTGA
>GWHPAAAL007799
ATGGCAACGCAAACGAAAGAATACGGAGCTCACGTATTGGCCATCCCATGGCCAGCACCAGGTCACATGA
ACCCTCTGCTTCAACTATGCAAACGCCTCGTATTGAAGGGTGGGATCAAAGCCACTTTCGCCATCACCAA
ATTCCAATCGAAGTCGATGTACTCAACCCTCGACTACATCCAAGTAGACACAATCTCCGACGGCTACGAC
GAAGGCGGCTTCTTCATCGTCGATGGCATCACCGCCTCCGTCGAAAAATTCAAGGCCGCCGGATCTGAAT

CCTTGGCCGAGCTCATCAGGAAGCACGAGGGATTAGGGCAACCCTTCGATTGCATCATCTACGATGCGTT
CTTGCCGTGGGTTCTGGATGTGGCCGGTGAATTTGGAATCTCTACGGCGGCGTTTTTCACGCAGAAATGT
TCTGTGAACTGCATCAACCACCATGTCTTAAAGAAATTGATTCCGTACCCTTTCACGTCGGAACCAATTT
CGATTCCCGGTTTGCCTCTTTTGAATATTGAGGACATGCCCGGATTCATACATGTGGATCCGGAGTTGTA
TGAGCTGGTGCAGAGTCAATTCTCCACGCCTGAGCTAGCTGATTATCGACTTGTGAACACATTCTACGAG
TTGGAGTCTGAGGCGGTGGAAGCAATGTCAAAGATCCATTCCCTGAGAACAATCGGACCAACAATTCCCT
CTTTCTACCTAGACAAGAGAATCGAAAACGACAGCAGCTACGGCCTCCACGCCACTCACTTGATGTTCCA
AGAGAAAAACGCCTCCCGTGTAGAGCCTTCTGTGGTCCTAAACTGGCTAGACGCGAAACCCACTGGCTCA
GTTGTGTACGTGTCCTTTGGCAGCGTCTCTATAGCCAGTCCAGGACCCGATGAAATGAAGGAAATCGCGT
TCGGCTTAAAAAACACCAATTTTCACTTCTTATGGGTCGTGAAGCCCAACGAAGAGCCGAATCTCCCTGA
AAATTTCAAGGAAGAGATGGCCGATAAAGGGTTGATTGTGTCGTGGAGTCCCCAGTTGGAAGTATTGGCA
CACAAGGCTCTCGGTTGTTTTGTCACTCATTGTGGGTGGAATTCAACTATTGAGTCGCTGTGTATGGGAG
TTCCGATGGTGGCAGTGCCCAAATGGGCTGATCAACCTACGAACGCCAAGTATGTGGAGGATGTTTGGAA
AGTGGGGATAAGGGTGAAGGTGGACGAGAATAGGTTTGCGGGAAGAGATGAGGTGGAGAGGTGTATTAGA
GCGGTGATGGAGGGGGAGAGGGCGGAGGATTTGAAGAGAAGCGCTCTCAAATGGAAGGAGTTGTGTATAA
AAGCGGTTGCTGAGGGTGGGAGTTCGGACAAAAACATGGAGGAGTTTGCGTCTGAATTGATTACGAGAAA
AAGTGCCAGCAAATAG
>GWHPAAAL007800
ATGGAGGAGAAGCAAGGAACGTCGGACAGAGTTCACGTTCTGGTCGTCCCTTTCCCGGTACAGGGTCACA
TCAATCCCATGCTTCAATTCGCCAAACGCTTGGCCTCCAGCGGCCTCAAAATCACCGTAATCGGCACCAC
CAAAACCACCGTCAGCCGCTCCATTCCAAACATTGAGATTGAGCGAATCCCCGACGCTCCTGGAGAAGCA
GCGGATCAACAAAACACGGAGACCATCGAAGAACACTGCGAACGACTCACGACGGTGCTCTCGAACTTCT
CCGTGAAACTTATCGAGAAACAGAGCGGCCTCGGCTGCCCTTACCGCGTCCTTGTGTACGACTCGGTTAT
GCCGTGGGCGCTGGAGTTGGCGCACCGGCGGGGCTTAAAGGCGGCGCCTTTTTTCACCCAGTCGTGCTCT
GTTTCTGCTCTGTACTACCATATGAATCGTGGGTCGCTGAAGGTTCCACCGGTAGGGTCGTCGGAGTTCG
TGGAGGTGCCTGCGCTACCGCCGTTGAAGGTCGGTGATCTGCCGTCGTTTATCTATGACATGGGCTCGTA
CCCCTTTATACTGAAAATGGTGGTCAATCAGTTCCTGAATTTTGAGAAAGCGGATTGCCTGTTGTTTAAC
ACTTTTGACAAGCTGGAAACTGAGATGGTGACTTGGATGGCAAACCAGTGTCCAACGATCAGGACAATCG
GACCAACTCTTCCGTCGGTGTACTTAGACAAACGATTGAACGAAGACACGGATTACGGACTCAGTCTCTT
CAACCCAGACCGCGAATCCAGCATCCAATGGCTCCACTCCAAACCCACCGCTTCCGTCGTCTACGTCTCC
TTCGGTAGCCTCGCCAACCTTTCAGAAACCCAAATGCATGAGCTAGCGTTCGGCCTCGAAAGAACGGACT
CTCACTTCCTCTGGGTTTGGACTGATCAGACGACGAATGCCATGTTAGCGGTAGAGGTTTGGGGAGTGGG
GGTACGGACGGCGGCGGACAAGAAGGGAATTGTGAGGAGAGAAGAAGTGGAGATGCGCGTGAGGGAAGTG
ATGGAGGGGAATGGGAGAGAGGGGTTGAAGAGTAATGCTGGGAGATGGAAGGAGTTGGCTAGGGAGGCTA
TGGACGGAGGTGGTAGCTCCGATGTGAATATTCAGGACTTTGTTACAAGCCTTGCATGCAACTAA
>GWHPAAAL007936
ATGGCCGAAAATACTCACGTCGTGATGCTTCCATGGTCGGCGTTCGGTCACATTATCCCCTTCTTCCATC
TCTCCATAGCCTTATCTAAAGCCGGAATCCACGTGTCCTTCGTCTCCACACCGAAAAACATCCGGAGACT
CCCCCAAATACCGCCGGATTCCGTTCCTTTCATTGACATCGTGGAGCTCCCGTTGCCGGAGATCGACCGG
AGTCTCTTGCCGGAAGATGCAGAAGCCACCGTCGACATTCCCACTGAAAATATTCAGTACCTTAAGATAG
CTTACGACCTCTTGAAAAAACCCTTCAGTCGGTTCGTTGCCGACCGGTCGCCGGACTGGATCGTCATCGA
CCTGATGCCTTACTGGGCCGCCGACGTTGCCAAGGAGTTCGACATTCCTGTCATTTACTTCAGCATGTTT
TGCGCCGCCGCTTCCGCATTCTTCTCTCGGCCGGAATTTATCGCTGGCGAAGGTCAGAAAATGAAACGTT
CGACTCCGGAATCCCTGACTTCGACTCCGGAGGGGGTCGATTTCCCGTCAACGGTTGCTCATCGGCATCA

TGAACCGCTGTCTTTCCACGGAGAAAACGCCTCCGGCATCAGTGACGCCAGCCGCTTTGCCAGAACACTC
CATTCATGTAGAGCCCTGGCCATTCGCAGTTGCCCCGAGTTCGAACACGATGACTTGAATCTACTTGAGA
AAATCCATCAAAAATCGGTGATTCCCGTCGGACTTCTCCTGCCGGAACAAAAATCGGAAGCTAGAAACTC
CGGCGATGAATTCAAAATCTTCAAATGGCTTGATCAACAAAAACCCAAATCAGTTATCTTCGTCGGATTC
GGAAGCGAATGCAAACTAACCGAAAATCAAATTCACGAAATAGCTTACGGTCTGGAGCTGTCCGAACTCC
CATTTCTATGGTCGCTCAGAAAACCAAGCTATTCAACCGACGAGGTCGACCCTCTTCCGCCGGATTTCCA
CCGCCGCATCGCCGAAAAAGGACTAGTACACGTCGGATGGGCGCCGCAGACGGAGATTCTCGCCCACCCA
TCCGTCGGAGGCTCTCTGTTTCACTCCGGCTGGGGATCGGTGATCGAGACTCTGCAATTCGGACACCGTC
TCGTCGTCTTGCCGTTGATTATCGATCAGCCGTTGAACGCGAGGTTGCTGGTGGAGAAGGGTTTGGCGAC
AGAAGTGGAGCGAGGCGGAGATGGTTCGTTTGGAAGGGACGCCATAGCTAAAGCTCTGAGAGAAGCCATG
GTTATGGAAGGAGGAGAGAGAGTGAGAGAAGCCATGGAGATATTCGGAGATCGGAAGTTGCAAGAGAACT
ACGTGGAGAGATTTGCCGAATACCTGAAAAATGGAGTGGCGAAAAAGAATCAGTAA
>GWHPAAAL007941
ATGGCCAAAAATACTCATGTCGTGATGCTTCCATGGTCGGCGTTCGGTCACATTATCCCCTTCTTCCAGC
TCTCCATAGCCTTAACTAAAGCCGGAATCCATGTGTCCTTCGTCTCCACACCGAAAAACATCCGGCGACT
CCCCCAAATACCGTCGGATTCCGTTCCTTTCATTGATTTGGTGGAGCTCCCGTTGCCGGAGATCGACCGG
AGTCTCTTGCCGGCAGATGCAGAGGCCACCGTCGACATACCCACTGAAAATATTCAGTACCTTAAGATTG
CTTACGACCTCTTGAAAAAACCCTTCAGTCGGTTCGTCGCCGACCGGTTGCCGGACTGGATCGTCATCGA
CATGATGCCTCACTGGGCCGTCGACGTGGCCAAAGAGTTCGACGTTCCGGTCATTTACTTCAGCGTGTTT
GGCGCCGCCGCTGCCGTATTCTGGGGGCCGCCGGAGTATATCGCTGGCGAAGGTCAGAAAATGAAACGTT
CGACCCCGGAATCCCTGACTTCGACTCCGGAGTGGGTCGATTTCCCATCGACGGTTGCTTATGGGCATAA
CGAAGCGGTGTCTTTCCACGCTGGAGCCTACGGAGAAAACGCCTCCGGCATCAGTGACGCCGGCCGTACT
GCCAGAATACTCCATTCATGTAGAGCCCTGGCCATTCGCAGTTGCCCCGAGTTCGAACACGATTACTTGA
ATCTACTCGAGAAAATCCTTCAAAAACCGGTGATTCCCGTCGGACTACTCCCGCCGGAACAAAAATCGGA
ACCTAGAAACTCCGGCTATGAATTCAAAATCTTCAAATGGCTTGATCAACAAAAACCCAAATCGGTTATC
TTCGTCGGATTCGGAAGCGAATGCAAACTAACCGAAAATCAAATTCACGAAATAGCTTACGGTCTGGAGC
TGTCCGAACTCCCATTTCTATGGTCGCTCAGAAAACCAAGCTATTCAACCGACCCTCTTCCGCCGGATTT
CCACCGCCGCATCGCCGAAAAAGGACTAGTACACATTGGATGGGCGCCGCAGACGGAGATTCTCGCCCAC
CCATCCGTCGGCGGCTCTCTGTTTCACTCTGGCTGGGGGTCCGTGATCGAGACTCTGCAATTTGGACACC
GTCTCATCGTCTCGCCGTTGATTATCGATCAGCCGTTGAACGCGAGGTTGCTGGTGGAGAAGGGTTTGGC
GAGAGAAGTGGAGAGAGGCGGAGATGGGTCGTTTGGAAGGGACGCCATAGCTAGAGCTCTGAGAGAAGCA
ATGGTTATGGAAGGAGGAGAGAGAGTGAGAGAAGCCATGGCGATATTCGGAGATCGGAAGTTGCAGGAGA
ACTATGTGGAGAGATTTGCCGAATACCTGAAAATTGGGGTGGCAAAAGAGAATTAA
>GWHPAAAL008406
ATGGGTGATGATCCCCATGTTCTTGTCGTACCTTATCCAGCACAAGGCCATGTAATTCCTCTAATGGAGC
TCGCCCATTGCTTAGCTCAGCATGAGATCCGGGTCACGTTCGTGAACTCGTACTTCACTCAGGAGCGAGT
GACGGAATCGTCGCCGGAGAAAAATGGGTTCGGAGAGGATTCGATTCGCCTGGTGTCAATCTCCGACGGG
TTGGACCCGTCGGAGGATCGGAACGAACTGGGGAATTTGTCCGAATCGATTTTTCGGGTGATGCCGGAGA
AACTGGAGGAGCTGATTAAGGAGATCAATGGATCGGAGGGCGACCAGGTAGCGTGTGTTATTGCGGATAT
CAGTATGGGGTGGGTGATGGACGTGGCGGCGAAGATGGGGATCCGGCGGGCGGCGTTTGGTCCGACGTCG
GCGGCGATATTGGGATTGATTCACGATGTGCCCAAGCTAATGGAGGATGGGATTATAAACGACGACGGAA
CTCCGACTAAGAACGACGTGATTAAGCTATCACCGGACATGCCCGCCATGAACACAGCCGACTTCCCTTG
GGCCTGCATTGGCGATTTGGCCACCCAGAAGAAGATTTTCGACATCATGTTAGCAGGTCGCCGCGCTCTG
GATCTCGCCGACCGGATCATTTGCAACTCGTCTTCCGGTTTGGAGCCCGCCGTCTTCGATCAGTACCCAC

AATTCCTCCCCGTCGGCCCACTCCTCCGTAGCTCCGGCGGAAACTTCTGGCCGGAAGATTCCACCTGCCT
GGACTGGCTAGACCAACAACCCCCCAAATCCGTCATCTACGTCGCGTTCGGAAGCTTCACCGTTTTCGAC
CCAACCCAGTAG
>GWHPAAAL008407
ATGGGTAATCCCCATGTTCTTGTCGTCCCTTATCCAGCACAAGGCCATGTAATTCCTCTAATGGAGCTTG
CCCATTCCTTGGCTCAGCATGAGTTCCGGGTCACGTTCGTGAACTCGGATTTTACCCAGGACCGAGTGAC
GGGATCATCGCCGGAGAAAAATGGGTTCGGAGAGGATTCGATACGCCTGGTGTCGATCTCCGACGGGTTG
GAGCCGTCAGAGGATCGGAACGAACTGGGGAACTTAACGGAATCGATTTTCCGGGTGATGCCGGCGAAAC
TGGAGGAGCTGATTATGGAGATCAATGGGTCTGACGGCGACGAGGTGGCGTGTGTTATTGCGGATATCAG
TATGGCGTGGGTGATGGAAGTGGCGGCGAAGATGGGGATCCGGCGGGCGGCGTTTTGTCCGATGTCGGCG
GCGATGCTGGGATTGGTTTACAATGTGCCTAAGCTAATGGAGGATGGGATTATAAACGACGACGGAACTC
CGACGAAGAACGAGGTGATTAAGTTAACGCCGGACATGCCCGCCATGAACACGGCCAACTTCCCGTGGGC
CTGTATCGGAGATTTGGCCACCCAGAAGAAGATTTTCGACGTCACGTTAACAGGTCGCCCCGCTCTGGAT
CTCGCCGACCGGATCATTTGCAACTCGTCTTCCGGTTTGGAGCCCGCCGTCTTCGATCAGTACCCACAAT
TCCTCCCCGTCGGCCCACTCCTCCGCAGCTCCGGCGGAAACTTCTGGCAGGAAGATTCCACCTGCCTCGA
CTGGCTCGACCAACAACCGCCCAAATCCGTCATCTACGTCGCGTTCGGAAGCTTCACCGTTTTCGACCCA
ACCCAGTTCCGTGAACTAGCCAAAGGGCTAGAACTCACTGACCGGCCGTTCCTTTGGGTTGTCCGGCGGG
ACTCCGCCGTCAAATACCCGGAAGGGTTTGAGAAGATAATCGGTACCGGTCGGCGGGGGAAAATAGTAGA
GTGGGCTCCACAGAAGAAGGTATTAAGCCATCCTTCGGTGGGGTGTTTTCTGAGCCACTGCGGTTGGAAC
TCTACAGTTGAAGCAGTTAGCAATGGGGTCCCACTTTTATGTTGGCCCTACTTTGCTGATCAGTCCTTCA
ACCAAAGCTATATTTGTGATGTTTGGAAAGTTGGATTAGGGTTTGGAAAAGACGAAAATGGCCTTATAAT
TTTTGAAGAAATTAAGAAAAAAGTGGATGAAATTTTTGGTGACGAGAATTATAGAGAGAGGGCTTTGGAT
TTGAAACAAAAAGTCATGGATGGTGTTAAAGAAGGTGGGTGCTCCAATAGAAATTTTAGCAAAATTATGG
GGTGGATTACGCAAATAAATAATGGTTTGTCTTCGTGA
>GWHPAAAL008878
ATGGCTTCCCCATCGCCTCACTTCGTGCTGCTTCCCTTTTTGGCTCAAGGCCACCTTATTCCCATGATAG
ACATTTCCAGATTGCTAGCAAAACGCGGAGTAGTAGTGACCATAATCACTACTCCCCTAAATGCCAAGAG
ATTCGAAAGAATCATCACCCGGGAAATAGATTCCGGCCTCCAAATCCGAGTAGTCCAACTCCACTTCCCT
TGCGTGGAAGCCGGTTTGCCCCAAGGGTGCGAGAATTTCGACCACTTGACCACAATGGCTTCCGCTGGAC
AATTCTTGGAGGCAACTCGAATGCTTCAAAAGCCAGTGGAAGATCTCTTCGAAGGGTTAAGCCCGCCCCC
AACTTGCTTGCTGTCCGACATGTGTTACCCATGGACTACCCTTGTTGCGGAGAAGTTTTCCGTTCCGAAG
CTTGTTTTCCACGGAATGTGTTGTTTTTCCCTCTTTTGCAATCATGTTTTGTACACGTCGAGGGTTTTCG
ACACAGTCGCCTCAGAAACCGAGCCTTTTGCAGTGCCCGGCATGCCTGATCATATCGAATTGACGAAAAC
TCAAGTTCCGACATTGGTCAACATGAATACATCGTCGGGGGTAAAAGACGTGCGGAAAGAGATCAAGGAA
GGAGAGCTGTCAGCCTATGGCGTCGTGGTCAACACTTTCGAGGAGATGGAGGAGAAGTACGTGAAGGAAT
ACGCAAAGGCGATGGGGAAGAAAGTTTGGTGCATTGGGCCGGTTTCATTGTGCAATAAGAACAACTTGGA
CAAGGCCGACAGAGGGAACAAGGCCTCCATCGACGAGCACCACTGCTTGAAGTGGCTCGACTCTTGGGAA
CAAGGCTCGGTGGTATACGTTTGCCTCGGGAGCCTAACTCGCCTAGCAACCTCACAAATGATAGAGCTCG
GGCTTGGGCTAGAGGCATCGAATAGGCCATTCGTATGGGTCATAAGGTATGAAATCGTTGAATTTGAGAA
ATGGATTTTGGAAGAAGGGTATGGGGAGAGGATTAAGGGAAGAGGGCTTTTAATCCACGGGTGGGCCCCA
CAAGTGTTGATATTATCGCACCGGGCGATCGGAGGGTTCCTGACGCACTGCGGGTGGAATTCCACAATGG
AAGGGATATGCGCGGGGGTTCCAATGCTGACGTGGCCGATATTCGCAGAGCAATTTGTTAACGAGAAGTT
GGTCGTGCATGTACTGAAGACTGCTGTGAGCGTCGGCGTGAAAATTCAAATCTATCCTGGGAACGAAGAG
GAGGTCGGCATTTTGTTGAGGAGGGAAGACGTCAAGATGGGTATAGATAAGGTGATGGAGGAAAAAGAAG

AAGGGGAAGAGAGGAGGAGGAGAGCCAGGAAGTTTGGGGAGATGGCAAAATGGGCGATAGAAGAAGGGGG
TTCTTCACACCACAATTTAACCATGTTAATTCAAGATGTCATGGAGAAATCAAATCAACTTCGAAAAGAT
GGGTGA
>GWHPAAAL008879
ATGGCTTCCCCATCACCTCACTTCGTTGTGCTACCCTTTTTGGCTCAAGGCCACCTTATTCCCATGATAG
ACATTTCCAGATTGCTAGCAAAACGCGGAGTAGTAGTGACCATAATCACTACTCCGCTAAATGCTAAGAG
ATTCGAAGGAATCATCGCCCGGGCAATAGATTCCGGCCTTCAAATCCGAGTAGTCCAACTCCACTTCCCT
TGCGTGGAAGCCGGTTTACCCCAAGGGTGCGAGAATTTCGACCACTTGATCTCAATGGCTTCCGCTGGAC
AATTCTTTGAAGCAACTCGAATGCTTCAAAAACCTGTGGAAGATCTCTTCGAAGGGTTAAACCCGCCCCC
AGCTTGCTTGCTGTCCGACATGTGTTACCCATGGACAACCCTTGTTGCGGAAAAGTTTTCTGTTCCGAAG
CTTGTTTTCCACGGAATGTGTTGTTTTTCCCTCTTTTGCACTCACGTTTTGCAAACGTCGAGGGTTTTTG
ACACAGTCTCCTCAGAAATCGAGCCTTTTGCGGTGCCTGGCATGCCTGATTTTGTCGAATTGACGAAAAA
TCAAGTTCCGGCATTCGTCAACATGAATATACCGTCGGGGCAAAAAGACGTGCGGAAAGAGATCAAGGAA
GGGGAGCTGTCGGCATATGGCGTCGTGGTCAATACTTTCGAGGCGATGGAGGAGAAGTACGTGAAGGAAT
ACGCCAGGGCAATGGGGAAGAAAGTTTGGTGTATTGGGCCGGTTTCATTGTGCAACAACAACAACTTGGA
CAAGGCCGACAGAGGGAACAAGGCCTCGATCGACGAGCACCATTGCTTGAAGTGGCTCGACCATTGGGAA
CAAGGTTCGGTGATATACGTTTGCCTTGGGAGCCTAACTCGGCTAGCAACTTCACAAATGATAGAGCTCG
GGCTAGGGCTAGAGGCATCAAATAGGCCCTTTGTATGGGTCATACGGTATGAAATCGTTGAATTTGAGAA
ATGGATTTTGGTAGAAGGGTTTGGAGAGAGGATTAAGGAAAGAGGGCTTTTAATCCGTGGGTGGGCCCCA
CAAGTGTTGATATTATCGCATCGGGCGATCGGAGGGTTCCTGACCCACTGCGGGTGGAATTCCACGATGG
AAGGAATATGCGCGGGTGTTCCAATGCTGGCGTGGCCGATGTTCGCAGAGCAATTTGTGAACGAGAAGTT
AGTCGTGCATGTGCTGAAGACTGCTGTGAGCATCGGTGCGAAAATTCAAATCAGTATTGGGAAGGAAGAG
GAGATCGGCATTTTGGTGAGGAGGGAGGACGTCAAGATGGGTGTAGATAAGGTAATGGAGGAAAACGAGG
AAGGGGAAGAGAGAAAGAGGAGAGCCAGGAAGTTTGGGGAGATGGCAAAGTGGGCAATAGAAGAAGGAGG
TTCTTCACACAACAGTTTGACTATGTTAATTCAAGATGTCGTGGAGAAATCAAATCAACTTAGGAAACAT
GGGTGA
>GWHPAAAL009069
ATGGCTTATCCTCCACCTTCTCCCGCACTGCACATTGCTCTCTTCCCCTTCATGTCCAAAGGCCACACCA
TTCCGATCATCCACCTAACAAGACTCCTCCTGAACCGCGGCCATAAAACCACCGTTTTCACCACCCCAGC
AAACCGCCCCTTCATCTCTGACTCCCTCACCGGCACCGACGCCTCCGTCATTGTTCTCCCCTTCCCGGAA
AACATCCCAGGAATACCGTCCGGCACCGAAAGCACCGACAAACTACCGTCAATGTCCCTCTTCGTCCAGT
TCGCCAACGCCACAAAACTCATCCAACCTCACTTCGAGCAAGCCATCAAGTCACTCCCCAACCTCAACTG
CATGATATCCGATGGCTTCCTCAGCTGGACCCTCCAATCGGCATCTAAATTCGGCATCCCAAGGCTAGTC
TTCTACGGTATGAACAACTACTCCATGGCACTAAACTACGACGTGACCGTCAATAGGCTCCTCTCGATAC
CCAAATCGGACAACGAGCCATTCGTCGTCCCTAACTTCCCCTGGATCAAACTCACCAAGAATGACTTCGA
AGAGCCCTTTAACGAGGTTGAACCAAAGGGACCTTATGTGGAATTCACCATCGAACAGAGTCTAGCCACG
TTTAATAGCTACGGTCTGATCGTGAACAGCTTCTTCGAGCTCGAGAAGCCGTTCGTGGAGTACTGGAATC
GCGCGAGCAAACCGAAAGCTTGGTGCATTGGGCCTCTCTGTTTCGCAGAACCGGAAATGATCATCGAAAG
AAAGCGCAAACCAAAGCCCAAGTGGATCGATTGGCTGGACCAAAAGCGAGACCAGGGAAAATCCGTTCTG
TACGTTGCGTTTGGGTCTCAGGCGGAAATTTCACAGAAGCAGCTGCATGAAATAGCAATGGGGTTAGAGA
AATCGGAGGTGAACTTTCTATGGGTGATGAGAAAGAAAGACACGGCCATTGAGGGAGTGCCGGAGAGAGG
AATGGTAGTGAGAGACTGGGTTGATCAGAGGGAGGTGTTGGCACACGAGGGGGTGCGGGGGTTTCTGACG
CACTGCGGTTGGAACTCGGTGTTGGAGAGCATATGGGCAAGGGTTCCGATACTGGCGTGGCCGATGATAG
CGGAGCAGCACCTGAACGCGAGGATGGTGGTGGAGGAGATCAAAATCGGATTAAGAGTGGAGACGATTGA

CGGCTCTGTAAGAGAGGGTTTGTGAAGAGGGAAAGCTTGGAGAAGATGGTGAGGGAGCTGATGGAGGGGGAT
ATGGGGAAGGAGGTGAGGAAGAAGGTGGAGGAGGTGGGAGTGGCGGCCGGAAAAGCAATGGCAGAGGGTG
GGTCCTCTTGGGATGCTCTGAATCAGTTTATTGATGAGATCAAAGCACAGGGATCTAATCCAATCAATTG
A
>GWHPAAAL009324
ATGGATCGATCCGCAAGCTCAAAGCTTCACATTGCCATGTTTCCATGGTTCGCCGTCGGACATTTAACGC
CGTTTCTCCATCTCTCCAACGAGATAGCTCAGAGAGGCCATAAAATCTCCTACTTATTACCCAGAAAAGC
TCATATTCAGTTGCAGAGCCAGAATCAACACCCGGATCTGATCACATTCTATCCGCTCGCCGTCCCTCCG
GTCGCCGGTCTTCCCTCCGGCACCGAGACGGCCTCCGATATACCCATTTCCCAGACCGGCCTCCTCGCCA
CCGCCATGGACCACATGCGCGGCGAGGTCGAAGAAATTTTTCGTTCTCTCAGGCCTGATTTCGTGTTCTA
CGACCTTGCTCACTGGATCACAGAGCTCGGATCGAGTATCGGGTTCAAAACCGTGTGCTACAATGTCGTT
TCCGCCGCCTCGCTGGCGATCGCGCTGGTTCCGGCGCGTGTGGTTCCCAAGGACCGTAAGGTAACGGTGG
ACGAACTGGCGGTGCCGCCGAAGGGATACCCATCTTCCTCCGTGGTGCTCCGCCCCCACGAGGCCCGCTC
CCTGCTCTTCATCTCGTCGGAGTTCGGCGCCGGGGGAGTCACGTTCTACGAGCGAGTCATCGCCGCCATG
AAAAACTGCGATGCGATTGGAATCAGAACCTGCCGCGAAACCGAAGGCTCCCTCTGCGACTACATGGGAA
CCCAGTACAACAAACCGGTGCTCCTCACTGGACCCATCTTGCCGGAACCGTCCAAAGCTCAACTCGACGA
CCGGTGGTCCGATTGGCTCAACAAATTCCCCCCGAAATCCGTCATCCTCTGCACATTCGGAAGCCAAATC
GTCCTCGAAAAAGGCCAATTCCATGAACTCGTCTTGGGCCTCGAATCAACAGCACTACCATTCCTGGCCG
CAGTAAGAACTTCCGCAGGAGAAGAAGCCCTGCCGGAGGGATTCGAAAACCGGGTAGGCGAAAGAGGGGT
GGTTTACGGCGGTTGGGTGCAGCAGCCATTGATATTGAACCACCCATCAGTCGGGTGCTTCGTCAGTCAC
TGTGGGTTCGGATCGATGTGGGAGTCTCTGATGAGCGATTGTCAGATAGTGGTGGTTCCGCATTTGGGTG
ATCAGATATTGAACGCGCGGCTGCTGGCGGAGGAGATGAAGGTGGCGGTGGAGGTGGAGAGAGATGAAAA
GGGGTGGTTTTCTAGAGAGAGATTGGGCGGAGCGATCAAGACGGTGATGGAGAGAGAGAGTGAGGCGGGA
GAGGTGGTGAGGAAGAACCATGCGAAGTGGAAGGAGATAATGGTAAAACCAGGGTTTATGAGTAATTACG
TGGAGAATTTCATAGAGAAACTTCACGAGCTATAA
>GWHPAAAL009436
ATGGGCTCGATTCCAGCCAGCGACAAGCCCCACGCCGTGTGCATACCGTACCCGGCACAGGGCCACATAA
ACCCAATGCTCAAGTTAGCAAAGATCCTCCATAGCCGAGGCTTCCACATCACCTTCGTCAACACCCACTT
CAACCGCAACCGCCTCCTCCATTCCCGTGGGCCCTCCGCCCTAGACGGCCTGCCCGACTTCCGATTTGAC
TCCATCCCCGATGGCCTCCCGCCGTCTGACGCCGACGCCACCCAGGACATCCCTTCGCTATGCGAGTCCA
CGCCGAAGCACTGTCTCGAACCCTTCTGCGACCTCATCGCCAGAACCAATGAAGATGCTGGAGTACCTCC
GGTAAGCTGTATCGTCTCCGATGGGGTGATGAGTTTTACGCTGAAGGCGGCGGAGAGGTTTGGGTTACCG
GAAGTGCTGTTCTGGACAACGAGCGCGTGTGGGCTGTTGGGGTATACACAATATCACAAGCTCGTTGAAA
GAGGATATACCCCACTCAAAGATGTGAGTTACGTGACAAATGGGTATATGGAAACAACAATGGATTGGAT
TGCCGGAATGAAAGACATCCGGTTAAGGGATCTACCCAGTTTTATTCGGACCACCGACCCGAAAGACACC
ATGCTCAATTTCCTCATCAGCGAATGCGCCGCCATCCCCACCGCCTCCGCCCTGATCCTCAACACCTTCG
ACGCCTTGGAACGCGAATCCGTCGACGCCCTCTCCGCCATCCACCCCCGGATCTACACCGTCGGCCCTCT
CAACCTGATGCTCAATCAGATTAACGACGACCGGATCAAGTCAATCGGGTCGAATCTCTGGAAAGAGGAT
CCGGGCTGCATCGAGTGGCTGGATTCGAAGGAACCCGGGTCGGTTGTGTACGTCAATTTCGGTAGCATCA
CCGTGGTGACGGCGGGTCAGCTCACTGAGTTCGCCTGGGGACTCGCCAACAGCGGCCGGCCCTTCCTATG
GATAGTCCGACCCGACATAGTTGCCGGCGATAAGGCGATGATTCCGCCGGAATTTCTGGCGGAGACGGAA
GGGAGGGGGAGGCTGTCAAGCTGGTGCCCGCAGGAGCAGGTGCTGAGGCATCCGGCGGTGGGTGGGTTCT
TAACCCACAGCGGGTGGAACTCCACCGTGGAGAGTCTATGCAGCGGCGTTCCGGTGATCTGCTGGCCCTT
CTTTGCAGAACAGCAGACGAACTGCCGGTACAGCTGTAGGGAATGGGGGGTGGGGATGGAGATAGATAAT

AAT
>GWHPAAAL010110
ATGGGGGAGAAGCAACCGGAGCTCCAAAAGCAACAGCGACAGGGGCTCCGATTGGTGCTCTTCCCACTTC
CATTCAAAGGCCACATCAACCCCATGCTTCAGCTCGCCGCCATTCTTCACGCCAATGGCTTCCCCATCTC
CATACTCCACACTACCTTCAACTCTCCCACCCCTTCCGACCACCCTCACTTCGACTTCCACCCAATCTCC
GTCGACGGTCTGTCGGAAACAGATTCTCCGACGCCGGATCTGGTTGATCTCCTCGAACTACTCAACCAAC
GATGCGCGGCGCCGTTCCGCGATTGCCTGGAGAAGTTGACATCGGAGGAGCCCGTCGCCTGTTTGATCTC
GGACGCCATTCTGCATTGCACCGCCACCGTCGCTGACAGTCTTCGTATTCCAAGGATCGTACTGAGGACC
GGCGGGGTCGCCTCCTTTCTTGCGTTTGCCTCCTTTCCACTTCTTCTGGATAAGGGTTATCTTCCCATTC
AAGAATCGAGGCTTGAAGAGGCAGTTCCAGAGCTTCCTCCACTGAGAATCAAAGATCTTCCGGTGATCAA
TACCGGCAAACCGGAGGCCCTATATCATCTTCTAGCAGAGATGATCAAGACGGCCAAGAACTCCTCAGGC
CTCATCTGGAACACCTTCGACGAACTTGAACAGCCGGAATTATCCAAACTCCGGCGTGAATTCCCCATCC
CAATCTTCCCAATCGGCCCTTTTCAGAAACACCTCCTCCTAACTTCCACACACGAAGACCGGAGTTCTTC
CATCTCTTGGCTTCAAAATCAACCCCCAAGCTCCGTCATCTACGTGAGCTTCGGCACCATTGCGTCAATG
GAGGAATCCCAATTCACGGAGATAGCCCATGGACTCGCCGACAGTCTCCATCCCTTCCTGTGGGTGGTCC
GGCCAGGTCTAATCCGTGGCTCGGAATGGCTCAAGAATTTGCCGGATGGGTTCTTGGAGAGGCTGGAAGG
AAGAGGGAAGATCGTGAAGTGGGCTCCACAGGCAGAGGTGTTGGCTCATCCGGCGGTCGGCGCATTCTGG
ACTCACAGCGGGTGGAATTCGACGGTGGAGAGTATCAGCGAAGGAGTTCCGATGATATGTATGCCGTGTT
TCACGGATCAGTTGGTGAATGCCAGGTATGTGAGCCATGTTTGGGGAGTTGGGTTGACGTTGGAAGGTGG
GATTGAGAGGGGGAAGATTGAGAGGGTGATGAGGAGAGTGATGGGTGAAGAAGGGAAAGAGATGAGGAAG
AGGATTTTGTGTATGAAGGAGAGAGTGAATGTTTGCTTGAGGAAAGGTGGATCCTCCCATCAATCTCTAC
ACAACTTGGTAAGTTACATCTCGTCACTCTAG
>GWHPAAAL010292
ATGGAGGATGTCCCTGCCGGCAAACCCCATGCGATCCTAGTAGCCTATCCTCTCCAAGGCCATGTAATCC
CAGCCGTCCATCTCGCCCTCAACCTCGCTTCACGAGGCTTCACGATCACCTTCGTCAACACCCAATCCAT
CCACCACCAGATCACTCACCGTCCATCCGGCAAAGCAGCCGCCGCCGCCGTTGACGATATCTTCGCCGCC
ATCCGCGAATCGGGTCTCGACATCCGATACACCACCGTTCCCGACGGGCTTCCGGCGGGGTTCGACCGGT
CGCTCAACCACGACCAGTTCATGGCGGCGCTGCTCCACGTGTTCTCGGCCCACGTAGAGGAGGCGGTGGA
TGAAATCGTGAGGGCAGGGCCGCCGGTAAGTTGTCTGATCGCCGACACGTTCTTTGTTTGGCCCGGAAAG
CTGGCGAAGAAGCTTGGGGTTGTGTATGCATCGTTCTGGACGGAGCCGGCTTTGGTTTTTACACTGTATT
ATCATCTTGATCTTCTAAGGATACATGGCCACTTCGGTTGTATTGAATTACGAGATGATATAATTGACTA
CATACCCGGTGTCAAATCCATCGAGCCAAAGGACTTGACATCATACCTTCAACAGTCGGATTCATCGACC
GTCTGTCACCAAATCATCTGCAATGCATTTAACGATGTCAAGACGGCGGATTTCGTCCTATGCAACACAG
TCCAAGAACTTGAACCCCAAACTATAACCGCCCTACAATCGAAAACCCCATTTTTCGCCATTGGACCCAT
CTTCCCATCCGGAGGCTTCACCACCAACAACGTGTCCACCAGCTTGTGGTCTGAGTCCGACTGCACCCAT
TGGCTCGACGCCAAGCCAGATGGTTCGATCTTGTACGTCTCGTTCGGGAGTTATGCCCACGTCGAAAAAA
ATGACCTTGTGGAGATCGCAAACGGACTATTGCTCAGCAAAATCGAATTCTTATGGGTTCTACGGCCTGA
TATAGTCAACTCTGATGATTCCGATCCACTGCCCGAGGTACTCAAGGAGGAGATCGGCAACCGTGGAATG
ATTATACCTTGGTGTAATCAAAGACAGGTTCTGGCTCACCGGGCGATCGGAGGGTTTTTAACTCATTGTG
GCTGGAACTCAGTACTGGAGAGTGTGTGGTGTCAAATCCCACTTCTTTGTCTGCCTTTGTTGACTGATCA
ATTCACTAATCGGAAATTAGTGGTCGATGATTGGAAGATTGGGACTAATCTATGCGATAAAAGGCCAATC
ACAAGGGGTGAAGTTTGTGAGAAGATTAAAGAAGTCATGAGTGAGAAATCAGGGAATGAGTTTAGGGAGA
AGATCAAGGAGGTGAAAAATAAATTGGAGAATGCATTAGGGGCAAATGGGTCATCCACCAAAAATATGGA
TCAATTTATCAAAGATCTAAAGGCCACTATACAAAAGAAACGTGGAGGGACATCCTAA

>GWHPAAAL011043
ATGGAAAATAACATTGAAAAGCCCCCCCATGCTATTATGTTCCCATATCCACTCCAAGGTCACTTAATCC
CCTTCGTCCACCTAGCCATCAAACTCGCCTCCAACGGCTTCGTCGTCACCTTCGTCAACACCCAATCCAT
CCACCACCAGATATCCCAGTCCCGAACTCCCGACGCCGGTGCAGACATCTTCGCCGGAGCTCGCAACTCC
GGTCTCGACATCCGGTACAAGACAGTGAGCGACGGCCTTCCGGTGGGCTTCGACCGGTCCCTCCACCACG
ACCAGTTCATGGAGAGCCTTCTGCACATTTTCTCGGCCCACGTCGACGAGCTCGTCGGAGAGATGGTCGC
GTCCGGAGACCCTCCGGTGAGTTGTTTGGTGGCGGATACTTTCTACGTGTGGCCGTCGACGGTGGCGAAG
AAATATAATCTCGTTAATGGTTCGTTCTGGACGGAACCGGCTCTGGTCCTAACTCTGTATTATCACCTCG
ACCTGCTCAGACAAAATGGTCATTTCGGCACTACTGATAGTTGCAAGGACAACATTGATTACATACCGGG
GGTCCCATCAATGAAACCAACAGACCTGATGTCGTATCTCCAGATCGATACATCAACGGTGGTGCACCGG
ATAATCTACAAGGCATTCAATGACGTAAAGAGTGCGGATTTTATCCTCTGCAACACGATACAAGAACTCG
AACCGGAGACGATCTCTGCCCTTCATAAAAAACAGCCCACCTACGCGATTGGGCCCATCTTCCCCGTGGC
CTTCACCAATAGCTCCGTGGCCACAAGCCTGTGGGCCGAGTCCGACTGCAGCCAATGGCTCAACACCAAG
CCTCATGGGTCTGTTTTGTACATCTCTTTTGGTAGTTATGCCCATACCACCAAACATGATATTATGGAGA
TAGCCTATGGGCTTTTGCTTAGTGGGGTGGGTTTTGTTTGGGTGCTTCGCCCCGACATTGTGAGTTCGGA
CGACGTTGACTTCCTACCCGTTGGATTTGATGAAAATATCAAGGGTCAAGGCTTGATCGTGCCCTGGTGT
CGTCAGATCGAGGTGATCTCGCATCCTGCGATTGGAGGGTTCTTAACACACTGCGGATGGAATTCGATAC
TGGAAAGTGTTTGGTATGAAATACCGTTGATTTGTTTTCCTCTGTTGACAGATCAGTTCACTAATCGAAA
ATTGGTTGTAGATGACTGGAAAATTGGGATCAATCTGCGCGATGAAAAATCAATCACGAGGGAAGAAGTA
TCAAAAAAGATTAACTATTTGATTAACGAAAAAACTTCTAATCAGCTAAGGAAGGAGATTAGGAAAGTGA
GAGTACTTTTAGAGGATGCTTTGGCAAACGATGGATCATCACAAAATAATTTTAATCAATTTATTAGGAA
TTTCAAGGATAAACTTAATGTAAAAAAGAAATAA
>GWHPAAAL011200
ATGACGATCGGCGGCGCCGGAGATCATATCCTGGTGTTCCCTTACCCAGCGCAGGGCCACATGATTCCTC
TCCTTGACCTCACCCATCAACTCGCCATCCGAGGCCTAACCATCACAGTATTGGTCACCCCCAAAAATCT
CCCTCTCCTCGATCCCCTTCTCTCCGAACACGAATCTGTCGCAACCTTAGTCTTGCCTTTCCCGGTGCAC
CCCTCCATCCCCGCCGGCGTAGAGAACGTCAAGGACCTTCCTGTCGGCGGATTCCGCGCTATGATGGTCG
CCTTGGGTGAACTCCACGATCCTCTCCTCAGATGGTTCCAGAACCAGCCTTCCCCACCGAAGGCCATATG
GTCGGATATGTTCTTAGGGTGGACCCATCACCTCGCCCGCCGTCTCGGCATCCGCCGATTCGTATTCTCT
CCATCTGGCGCTATGGCCTTGTCGGTAATTTACTCTCTGTGGCGTGAATTCCCAAGAAGAAGGGATTCAA
ACGACTACAACGAGAAATTTTCTTTCCCCAAAATCCCCAATTCTCCTATCTATCCCTGGTGGCAGCTCTC
CCCGATTTTCCGGAGTTATGTGGCCGGCGACCCTGATTCGGAATTCATCAAAGATGGGTTTCTCGCGAAC
ATAGCGAGTTGGGGACTCGTGATCAACTCGGTCAGCGAGTTGGAACGAGTATATTTGGAATATTTGAAGG
GGGAATTGGGTCATGATCGAGTATGGACGGTAGGACCGCTGCTCCCGCCGGATGGTGACCTCTCTAGGAC
CGTAGAGAGAGGTGGATCCAGCTCAGTATTAACCAATCAGATCTCATCATGGCTTGACAAGTGCAATGAT
CGAACGGTTGTGTACGTCTGCTTCGGAAGTCAAGCGGTGTTGAGCAATGATCAAATGGAGCATCTAGCCT
CGGGTTTGGAAAAGAGTGGGTCCAAGTTCGTGTGGTCCGTCAAAGGAGCTACAACGGGACAAGAGAAGGG
ATCATGCGGCCAGGTTCCTCTCGGATTCGAAGAGAGTGTGGGTGCGAGGGGGCTCGTGATTAGAGGATGG
GCACCGCAATTACTGATACTGAATCATCGAGCCGTGGGTGCGTTTTTAACCCACTGCGGATGGAACTCGT
TGCTGGAGGGATTAATCGCGGGGGTCCCTATGCTGGCCTGGCCTATGGGGGCAGATCAGTTTGCGAATGC
AAGCTTATTGGTGGACGAGCTCAGGGTGGCAATTCGTCTGTGCGAGGGTGCGCAGGTTGTGCCAAGTTCG
AATGAGTTGGCTCGACTAGTTACGGAAGCAACCGGGGGCGATTGGCCGGAAAGAGGACGAGCAGAGGAGC
TGAGAAAAGTTGCTTTGGGTGCGATCAAAGACGGTGGAAGTTCGTTCAACAATTTGAATGATTTGGTTAG
ACATATTTCTGAGATTGTGTAA

>GWHPAAAL012110
ATGGAGGAATCAAAGAAGGAGGAGCACATAGTGATGCTCCCTTTCATGGCGCAGGGCCACCTCATCCCAT
TTCTCGAGCTCGCCACCCAAATCCACCAATCCACCGCCTTCACCGTCACCATCGCCACCACCCCTCTCAA
CACCCAATACCTCAAATCCACCGCAGCTAAACAAAACCCATCTCCGGAATCTCCGCCGGCGCCGGCCATA
CACTTTGTCTCCCTCCCTTTCAACAGTTCCGACCATGGTTTACCCCCCAACACCGAGAACACCGAGTCAC
TGCCTCTGACCCAGATCATCTCCCTCTTCCACGCCTCCTCCTCTCTGAAACCACATTTTCAGGATCTTAT
CGCCGGAATCGCGGATAAAGAGGGTAAGCCTCCGATCTGCGTGATCTCCGACGTTTTCATGGGTTGGGCA
AACGAAGTTGCTTTGTCCTTCGGAACTGTGAATGTCAGTTTCACCACCGGCGGCGCGTACGGCACGGCGG
CGTACGTTTCGATTTGGCAGAATCTTCCTCACAGATCGAGGGATTCCAACGACGAGTTTAGTCTCCCGGG
TTTTCCCGAGTCCCGGCGATTCCATATCTCGCAGCTCCACCAGTTTCTCAGAGCCGCCGATGGCACGGAT
GAATGGTCTCTGTTTTTCCAGCCACAAATTTCGCTCTCGATGGGCTCTTTTGGGTGGTTATGTAATACGG
TGGAAGATATTGAGCCGCTGGGTTTGGAAATTCTCCGGCGATATACGAAGCTTCCGGTCTGGTGCATTGG
ACCTCTTCTCCCTCGAAAAATGCTCCAGAGATCGTCTTCTTCAAATTCCGGCGTAATCGGTCCCCGGAGC
GGAAAATTACCCGGAGTTTCTCCGGAAAAAATGCATCGAATGGCTTGAATTTCACCCTCCGGCGTCCGTTC
TCTACATCTCCTTCGGCTCACAGAACACGATCAGCGCAACCCAGATGATGGAACTCGCCCACGGTCTCGA
ATCGAGTGGCAAACCCTTCATCTGGGTCGTCCGGCCGCCGGTGGGTTCCGATCCAAACTCCCAATTCAAA
TCCTCCGAATATCTGCCGGAGGGATTCGAACGGCGAGCGGCCGAGAACAACAGAGGTCTACTAGTCCACA
AATGGGCACCCCAACTCGAAATCCTCTCCCACAAATCCACCGGAGCTTTTCTCAGCCACTGCGGCTGGAA
CTCGACGATGGAGAGCTTGAGCCAGGGCGTGCCGATCATAGGGTGGCCGTTGGCGGCGGAGCAATCCTAT
AACGCGAAGATGCTGGAGGAGGAGATGGGTGTCTGCGTGGAGCTGACCAGAGGAGTACAGAGTACCATTG
AGAGAGACAAAGTGAAGAAGGTGATCGAATTGGTGATGGGTGAGAAGGGAAAAGGTGAAGAGATGAGGAA
GAAGGCAGCCGCCATTGGAGAGGTGATCAGAGCTTCAGTCATGGAGGAGGGGGAAGGGAGGAAGAAGGGT
TCAAGTGTTCAAGCAATGGATGATTTTATATCAACACTCATCTCCAATAGAGAGCAAAGCTTTCCACACC
CACTTTGA
>GWHPAAAL012139
ATGGAGGAGAGGAGAGAGAACATAGTGATGTTCCCATTCATGGCGCAAGGCCACATGATTCCATTTTTGG
CCCTGGCCTTGCAATTGGAGAAAAAGGGTTACGGCATAACCTTTGTCAACACTCCTCTCAACGTCCTCAA
ACTCCGACAGTGTCTCCCACCGGACTCCTCCATTCGCATCCTTGAAATTCCGTTCAACAGCTCCGACCAT
GGTCTTCCTCCCAATTCTGAGAACACCGACGTCCTCCCCTACACCCTCATCGTCAACCTCCTCCACGCCT
CTCTCTCTCTGGAATCCTCCTTCAGGAAACTTCTCTCTGATCTTATCCACGAGCAGGGTGGCGTCCGTCC
GCTTTGCGTCATCGCGGATATATTCTTTGGGTGGTCGGCCGGCGTCGCGCGTGAGTTAGGCGTATTCCAC
GCGATTTTCAGCGGCGCCGGAGGGTTTGGATTGGCGTGTTACTATTCTGCGTGGTTGAGTTTGCCACACC
GGACAACGGATTCTATCGAATTCACGCTGCCGGATTTCCCTGAAGCCAGTAAATTTCATGTCACCCAGCT
GCCCGCGAGTATGCTGGCGGCTGACGGCACTGATCTGTGGTCGATTTTCCAGAGAAAAACCCTTCCGGCG
TGGACCAATTCGGACGGAGTTCTGTTTAACACAGTCGAAGGGTTCGACAAGCTCGGATTGGCATATTTCA
GTCGCAAAATCGGGAAGCCAATCTGGGCGATTGGACCAACACTTCTATCAGTGCACCACAGAGCTCGTGT
CGGCGGTAGAGAAGCCGCCATTACTCCAGAGCAATGCGCCGAATGGCTTGATTCGAAGCCACCCAACTCC
GTAATCTACGTATCATTCGGATCACAAAACACAATCTCAGCATCCCAGATGATGCAATTGGCGAAGGCTC
TGGACAAAAGCGAGAAGAATTTCATCTGGGTTGTTAGACCCCCACTTGGGTTCGACATAAATTCAGAGTT
TAAACCAGAAGAATGGCTGCCGGAAGGGTTTACAGACCGGATCAAAGATCAAAACAGAGGTCTAATCGTT
ATAAAATGGGCGCCCCAGGTGGAGATTTTGTCGCATAAATCTATAGGGGCGTTTATAAGCCACTGCGGAT
GGAATTCGGTGCTTGAAGCGTTGAGCAATGGGGTGCCGCTGATAGGGTGGGGCATGGCGGCGGACCAGTT
CTTCAACGTGAAGATGTTGGAGGAGGCGGTTGGAGTGTGCGTGGAGGTGGCGAGAGGGACCATTTTTGAG
GTGAGGCATGAAGATATAATGGAGAAGATAGAGGTGGTTATGGGGGAGAGTGAGAAGGGGAAGAGAATAA

GGAAGAGAGCTGGAGAATTGAAGGAGATAATCAAGGATGCAACGAGAGATGAGGAGGGCTTCAAGGGTTC
TTCCGTGAAAGCCATGGATGAATTTTTAAGTGCTGCTGCAATGTCGATGAAGGAGAAGAAGACATTATTG
GTTGAGACACAATGA
>GWHPAAAL013261
ATGGCTTCCGAATTCCACCATGCCCACTTTGTTTTAATACCACTTATGTCCCCTGGCCACATAATCCCCA
TGGTGGACATGGCGAAACTGTTGGCGAGACGTGGAGTGACAGTTACCCTAATCATGACCCAACATAGCGC
CACTAGATTTGCTTCTGTGGTGGACCGCGCCATTAAATCTGGACTCCCCATTCGGCTTCTCGAGTTTCGA
TTTCCCTCGAAAGAAGCCGGGTTGCCCGAGGGATGCGACAGTGTCGATGATATCCCTAGTTATGACTTGC
TACTAAACTTTTTTAATGCCATTGACATGTCACAAAACCCATTGGAGCAATTGATCGAACACCTAGAGCC
CAATCCTAGCTGCATAGTTTGCGATAAGCATATCGCATGGACGGCAGAAACTGCCAAAAAATTCAACATC
CCGAGAATTATTTTCGATGGGATGAGCTGTTTCAGTCAACTATGCGTCCATAATCTTCACGTTTCCAAGG
TCCACGAGAGATTTCAAGGGTCGGAGCCCTTCGTTCTTCCCGGATTGCCTGATGAAATCGTGATAACAAG
AGCTCAGCTTCCGGGGACATTCAACCCCGGATCGATGAAAAATTTGGACGAAATTCGTAAAAGAGTGAGA
GAAACTGAAAAGGAAGCGTATGGGGTTGTTGTGAATAGCTTCGAGGAGTTGGAACAAAGATATGTAAACG
AGTACAAAAAGGTAAGAGAAGATAGAGTCTGGTGCATTGGGCCGTTATCTCTGTGCAGCAATGAAACATC
GGACATGGCGAACAGGGGAAACACAGCGTCAATCGATGCGAACCATTGCTTCAAATGGCTCGATTCACGG
CAGCCAAGATCCGTAATCTACGCTTGTTTAGGAAGCCTGAGCCGCCTCACGCCTCCACAATTCATTGAGC
TCGCTCTAGGCCTTGAGTCAACAAATCAACCCTTTATTCTGGTGATCAAAGCAGGAGATAAAGTGAAAGA
AATAGAGACCTGGATTAGCGAAAATGGATTCGAGGAGAGAACCAGAGAGAGGGGCCTGTTGATTCGCGGA
TGGGCCCCCCAAGTGCTCATCTTGTCGCACGCAGCAGTTGGTGCTTTCCTGACCCATTGCGGTTGGAATT
CGACTCTGGAAGCGGTTTGCGCCGGCGTGCCTCTCATCACGTGGCCGCTCTTTTCGGAGCAGTTTCTGAA
CGAGAAGTTCGCAGTGCAGGTGTTGAAGATCGGGGAGAGTGTCGGAGCTAGGGTTGCAGTGGGCCTGGGG
GAGGAGGAGAAGTCGGGGGTGAAAGTGGAGAGGGAGGAGGTAGAGGCGGCGGCGAGGAGGCTAATGGAGG
AGGGAGAAGAAGGTGGGGGGAGAAGAAGACGGGCGAGAGAGTTGGCGGAGATGGCGAAGAGAGCGGTGGA
GGAAGGAGGATCTTCTCACCTTAACATGACAGTGATGATCCAAGATATCGTGTCCATCAAGAGAGAGGAG
ACATGA
>GWHPAAAL013365
ATGTCTACTCCTACTGATAATGGAGAAAAGCTACACATAGTTATGTTCCCATGGCTCGATTTCGATCACA
TGATCCCATTCTTAGAGCTCGCCAAGCTCATTGCCAAAAAGGGTCATAAACTCACCTACGTGTCGACCCC
CCAAAACATCAGATGGTTACCCAACCCTCCGCTAAATTGGGTCTTACTGCCACTACCAATAGTTGAAAGC
GTCCCTGATAATGCCAAGGCCACTGTTGATGTCCCCTCCGGCAAGGTGAGTTGCCTAGTGGATGCTTTCG
ATCGGCTTGAAACCGAGTTAACTCGGTTCTTGGAGGCATCAATGCCAGACTGGATTATTTACGATTTTGC
CCCTCCATGGCTTCCAACAGTTGTGGGTAATCTCGGAATTTCAAGGGCTTTCTTCAACGTAAGAAACGCT
TCCTTCGTATATTCGATGATAAAGGGTTGTGACTTGCCCAATGATATCATAGGCCCACTTGGGGTCCACC
GTCTCTGGTCCAGATGCGACGTGTTCGCTACCCGAGACTGCACCGAATTCGAGCCCCATTGCCTGAACAC
TCTCCGCAAGGTTACCCAAAAGCCTGTGATTCCGGTGGGCTTGATGCCCTCCTTGGTGCACGATAGCGAA
GACGACAAAACCTACACATGGCTAGCAATCAACGATTGGCTACAAAAACAGAACAAAGAGTCGGTCGCCT
ATGTGGCGCTCGGGATCGAAGTTTTCAAGAATCAAGAATCGCTCAGCGAGATGGCAATCGGGCTCGAATT
GTCTAGTTTGCCCTTCCTATGGGCCCTCAGAAGGCCCCATGGCTCAACCGAGCTCGGCTCCGTCAAGCTT
TCATTAGGGTTTGAGGAGCGAACCAAGGCACGTGGGATCGTCTGCAGGACCTGGGTCCCACAAGCCAAGG
TCTTGGCTCACGGCTTGGTGGGTTGTTTCATGACTGGGTGCGGGTGGAGCTCTGTGATAGAGGGGCTTCA
GTTTGGTCGATCCCTATTGATGATGCCATTCACAGAAGAACAAAGGTTGAATACTAGGGTGTGCGTAAAG
AAAAACATGGGAATAGAAATAGCTCAAGTTGGATCGTTGACAAGAAATTCGGTGGCTGACACGTTGAAAC
TGGTAATGTCAAATGATAATAATGAAGGGAAACTTATAAGAGAAAAGGTGAAGATGATGAATTGGATATT

TGGAGATAAGGCCAGACATGACCTTTACATGGACAATTTTGTTGATTATCTTCAAAACTACAGAAGTCTA
AACAAGGTTCCCTGA
>GWHPAAAL013653
ATGACTTCTCCTTCTTCTTCTCCTCCCTCACTTCCCCATGTCTTCATGGTCTCATTTCCAGGCCAAGGCC
ACGTCAACCCTCTCCTCCGACTCGGCAAATACCTCGCTTCAAAGGGTCTACTCATCACTCTTTGCACGCC
GGAAATCTTCGGCAAACAAATCCGAAAATCAAATCCCATAATCGCCGGCGAAAACCCCATTAAAATCGGC
GAAGGTTTCATCAGGTTCGAGTTCATCCAGGACGATTTGGAAGAAGATGACCCCATATTCAAGGACTTCG
ATCTTTACTTTCCCGACCTCGAGATGAAAGGGAAAGCGGCTCTTCAACGTATGATCGAGGAAAACAAGGT
AATGGGTCGTCCGGTGTCGTGTTTAATCAACAATCCGTTTATTCCATGGGTTTCGGATGTTGCAGAAGCT
ATGGCGATTCCAAATGCTATGCTTTGGGTTCAGTCTTGTGCTTGTTTTGCGGCGTATTATCATTATGGTA
AAGGGTCGGTGCCGTTTCCTAGTGAGAGAGAGCTTGAGCTCGATGTTCAGTTGCCGGCGATGCCGCTTCT
GAAGCACGACGAGATTCCAAGCTTTCTGCATCCAACGACTCCGTACACGGTTTTCCGGCGGGCGATTATG
GGGCAGTATCGGAATCTGTCGTACCCTTTTTGTATACTGATGGATACTTTTATGGAGCTTGAGGAGGAGT
TGATTGAGAATATGTCGGAGATTTGTCCGGTGAAGCCGGTGGGGCCGTTGTTCAGGAACGCCGGAGGTTA
TGGGTCGGAGATTCGCGGCGATATGATGAGGGCCGACGAGGAGTGCTTGAAATGGCTGGATTCGATGCCG
GAATGTTCAGTCGTGTACGTGTCTTTCGGTAGCATCGTGAGTTTAAAACAAACTCAGCTCGACGAATTCG
CCGCCGGGATTTTGAATTCCGGCGTTTCGTTCTTGTGGGTGGTGAAACCGGTGCCCGAAAAGCCGGTGAA
GTTGCCGGAGGTTTTTTTGGAGAAAGCCGGGGATAGAGGGAAAATCGTGGAGTGGAGTCCACAGGAGCAG
GTGTTGGCCCACCCGGCGGTCGCGTGTTTCGTCACCCACTGCGGCTGGAACTCGTCGATAGAGGCGGTGG
CGAACGGCGTTCCCGTGGTGGCGTTTCCTCAGTGGGGCGACCAGGTGACGAACGCCAAGTACTTGGTGGA
GGTGTTCGGAGTTGGGCTCCGACTGAGCAGAGGGGAGGCGGAGAACAGGACAGTCACGGCGAAGGAGGTG
GAGAGGTGTCTCCTGGAGGCAACGAGCGGCGGAAAGGCGGCGGAGTTGAAGGAGAAGGCGTTGAAGTGGA
AGAAAAAGGCGGAGGAAGCGGTGGCGGAAGGTGGCTCGTCTAACCGGAATATGAAGGACTTTGTGGAGGA
GGTCAAAAGAAGAAGTATTTAA
>GWHPAAAL013654
ATGGTTTCCCCTTTTCCTTCTTCACTTCCCCATGTCTTCATGGTCTCTTTTCCAGGCCAAGGCCATGTTA
ACCCTCTCCTCCGACTCGGCAAATACCTCGCTTCAAAGGGTCTACTCATCACTCTTTGCACGCCGGAAAT
CTTCGGCAAACAAATCCGAAAAGCAAACCCCATAATCGCCGGCGAAAACCCCATAAAAATCGGCGAAGGT
TTCATCAGGTTCGAGTTCATCCAGGACGGTTTGGAAGAAGGAGACCCCATATTCAAGGACCCCGATCTTT
ACTTTCCCGACCTCGAGATGAAAGGAAAGGCGGCCCTTCATCGTATGATCGAGGAAAATAAGGAAATGGG
TCGTCCTGTATCGTGTTTGATCAACAATTCGTTTATTCCATGGGTTTCGGATGTTGCAGAGGCTATGGCG
ATTCCTAATGCTGTGCTTTGGGTTCAGTCTTGTGCTTGTTTTGCGGCGTATTATCATTATGGTAAAGGGT
CGGTGCCGTTTCCTAGTGAGAGAGAGCTTGAGCTCGATGTTCAGTTGCCGGCGATGCCGCTTCTGAAGCA
CGACGAGATTCCGAGCTTTCTGCATCCAACGACTCCGTACACAGTTTTCCGGCGGGCGATTATGGGGCAG
TATCGGAATCTGTCGTACCCTTTTTGTATACTGATGGATACTTTTATGGAGCTTGAGGAGGAGTTGATTG
AGAATATGTCGGAGATTTGTCCGGTGAAGCCGGTGGGGCCGTTGTTCAGGAACGCCGGAGGTTATGGGTC
GGAGATTCGCGGCGATATGATGAGGGCCGACGAGGAGTGCTTGAAATGGCTGGATTTGATGCCGGAATGT
TCAGTCGTGTACGTGTCTTTCGGTAGCATCGTGAGTTTAAAACAAACTCAGCTCGACGAATTCGCCGCCG
GGATTTTGAATTCCGGCGTTTCGTTCTTGTGGGTGGTGAAACCGGTGCCCGAAAAGCCGGTGAAGTTGCC
GGAGGTTTTTTTGGAGAAAGCCGGGGATAGAGGGAAAATCGTGGAGTGGAGTCCACAGGAGCAGGTGTTG
GCCCACCCGGCGGTCGCGTGTTTCGTCACCCACTGCGGCTGGAACTCGTCGATAGAGGCGGTGACGAACG
GCGTTCCGGTGGTGGCGTTTCCTCATTGGGGCGACCAGGTGACGAACGCCAAGTACTTGGTGGAGGTGTT
CGGAGTTGGGCTCCGACTGAGCAGAGGGGAGGCGGAGAACAGGACAGTCACGGCGGAGGAGGTGGAGAGG
TGTCTCCGGGAGGCAACGAGCGGCGGAAAGGCGGCGGAGTTGAAGGAGAAGGCGTTGAAGTGGAAGAAGA

AGGCGGAGGAAGCGGTGGCGGAAGGTGGCTCGTCTGACCGGAATATGAAGGACTTTGTGGAGGAGATCAA
AAGAAGAAGTATTTAA
>GWHPAAAL013655
ATGGTTTCCCCTTTTCCTTCTTCACTTCCCCATGTCTTCATGGTCTCTTTTCCAGGCCAAGGCCATGTTA
ACCCTCTCCTCCGACTCGGCAAATACCTCGCTTCAAAGGGTCTACTCATCACTCTTTGCACGCCGGAAAT
CTTCGGCAAACAAATCCGAAAAGCAAACCCCATAATCGCCGGCGAAAACCCCATAAAAATCGGCGAAGGT
TTCATCAGGTTCGAGTTCATCCAGGACGGTTTGGAAGAAGGAGACCCCATATTCAAGGACCCCGATCTTT
ACTTTCCCGACCTCGAGATGAAAGGAAAGGCGGCCCTTCATCGTATGATCGAGGAAAATAAGGAAATGGG
TCGTCCTGTATCGTGTTTGATCAACAATTCGTTTATTCCATGGGTTTCGGATGTTGCAGAGGCTATGGCG
ATTCCTAATGCTGTGCTTTGGGTTCAGTCTTGTGCTTGTTTTGCGGCGTATTATCATTATGGTAAAGGGT
CGGTGCCGTTTCCTAGTGAGAGAGAGCTTGAGCTCGATGTTCAGTTGCCGGCGATGCCGCTTCTGAAGCA
CGACGAGATTCCGAGCTTTCTGCATCCAACGACTCCGTACACAGTTTTCCGGCGGGCGATTATGGGGCAG
TATCGGAATCTGTCGTACCCTTTTTGTATACTGATGGATACTTTTATGGAGCTTGAGGAGGAGTTGATTG
AGAATATGTCGGAGATTTGTCCGGTGAAGCCGGTGGGGCCGTTGTTCAGGAACGCCGGAGGTTATGGGTC
GGAGATTCGCGGCGATATGATGAGGGCCGACGAGGAGTGCTTGAAATGGCTGGATTTGATGCCGGAATGT
TCAGTCGTGTACGTGTCTTTCGGTAGCATCGTGAGTTTAAAACAAACTCAGCTCGACGAATTCGCCGCCG
GGATTTTGAATTCCGGCGTTTCGTTCTTGTGGGTGGTGAAACCGGTGCCCGAAAAGCCGGTGAAGTTGCC
GGAGGTTTTTTTGGAGAAAGCCGGGGATAGAGGGAAAATCGTGGAGTGGAGTCCACAGGAGCAGGTGTTG
GCCCACCCGGCGGTCGCGTGTTTCGTCACCCACTGCGGCTGGAACTCGTCGATAGAGGCGGTGACGAACG
GCGTTCCGGTGGTGGCGTTTCCTCATTGGGGCGACCAGGTGACGAACGCCAAGTACTTGGTGGAGGTGTT
CGGAGTTGGGCTCCGACTGAGCAGAGGGGAGGCGGAGAACAGGACAGTCACGGCGGAGGAGGTGGAGAGG
TGTCTCCGGGAGGCAACGAGCGGCGGAAAGGCGGCGGAGTTGAAGGAGAAGGCGTTGAAGTGGAAGAAGA
AGGCGGAGGAAGCGGTGGCGGAAGGTGGCTCGTCTGACCGGAATATGAAGGACTTTGTGGAGGAGATCAA
AAGAAGAAGTATTTAA
>GWHPAAAL013658
ATGGTTTCCCCTTTTCCTTCTTCACTTCCCCATGTCTTCATGGTCTCTTTTCCAGGCCAAGGCCATGTTA
ACCCTCTCCTCCGACTCGGCAAATACCTCGCTTCAAAGGGTCTACTCATCACTCTTTGCACGCCGGAAAT
CTTCGGCAAACAAATCCGAAAAGCAAACCCCATAATCGCCGGCGAAAACCCCATAAAAATCGGCGAAGGT
TTCATCAGGTTCGAGTTCATCCAGGACGGTTTGGAAGAAGGAGACCCCATATTCAAGGACCCCGATCTTT
ACTTTCCCGACCTCGAGATGAAAGGAAAGGCGGCCCTTCATCGTATGATCGAGGAAAATAAGGAAATGGG
TCGTCCTGTATCGTGTTTGATCAACAATTCGTTTATTCCATGGGTTTCGGATGTTGCAGAGGCTATGGCG
ATTCCTAATGCTGTGCTTTGGGTTCAGTCTTGTGCTTGTTTTGCGGCGTATTATCATTATGGTAAAGGGT
CGGTGCCGTTTCCTAGTGAGAGAGAGCTTGAGCTCGATGTTCAGTTGCCGGCGATGCCGCTTCTGAAGCA
CGACGAGATTCCGAGCTTTCTGCATCCAACGACTCCGTACACAGTTTTCCGGCGGGCGATTATGGGGCAG
TATCGGAATCTGTCGTACCCTTTTTGTATACTGATGGATACTTTTATGGAGCTTGAGGAGGAGTTGATTG
AGAATATGTCGGAGATTTGTCCGGTGAAGCCGGTGGGGCCGTTGTTCAGGAACGCCGGAGGTTATGGGTC
GGAGATTCGCGGCGATATGATGAGGGCCGACGAGGAGTGCTTGAAATGGCTGGATTCGATGCCGGAATGT
TCAGTCGTGTACGTGTCTTTCGGTAGCATCGTGAGTTTAAAACAAACTCAGCTCGACGAATTCGCCGCCG
GGATTTTGAATTCCGGCGTTTCGTTCTTGTGGGTGGTGAAACCGGTGCCCGAAAAGCCGGTGAAGTTGCC
GGAGGTTTTTTTGGAGAAAGCCGGGGATAGAGGGAAAATCGTGGAGTGGAGTCCACAGGAGCAGGTGTTG
GCCCACCCGGCGGTCGCGTGTTTCGTCACCCACTGCGGCTGGAACTCGTCGATAGAGGCGGTGACGAACG
GCGTTCCGGTGGTGGCGTTTCCTCATTGGGGCGACCAGGTGACGAACGCCAAGTACTTGGTGGAGGTGTT
CGGAGTTGGGCTCCGACTGAGCAGAGGGGAGGCGGAGAACAGGACAGTCACGGCGGAGGAGGTGGAGAGG
TGTCTCCGGGAGGCAACGAGCGGCGGAAAGGCGGCGGAGTTGAAGGAGAAGGCGTTGAAGTGGAAGAAGA

AGGCGGAGGAAGCGGTGGCGGAAGGTGGCTCGTCTGACCGGAATATGAAGGACTTTGTGGAGGAGATCAA
AAGAAGAAGTATTTAA
>GWHPAAAL014206
ATGGCAAAAAATACTCATGTCGTGATGCTTCCATGGTCGGCGTTCGGTCACATTATCCCCTTCTTCCAGC
TCTCCATAGCCTTAACTAAAGCCGGAATCCATGTGTCCTTCGTCTCCACACCGAAAAACATCCGGCGACT
CCCCCAAATACCGTCGGATTCCGTTCCTTTCATTGATTTGGTGGAGCTCCCGTTGCCGGAGATCGACCGG
AGTCTCTTGCCGGCAGATGCAGAGGCCACCGTCGACATACCCACTGAAAATATTCAGTACCTTAAGATTG
CTTACGACCTCTTGAAAAAACCCTTCAGTCGGTTCGTCGCCGACCGGTTGCCGGACTGGATCGTCATCGA
CATGATGCCTCACTGGGCCGTCGACGTGGCCAAAGAGTTCGACGTTCCGGTCATTTACTTCAGCGTGTTT
GGCGCCGCCGCTGCCGTATTCTGGGGGCCGCCGGAGTATATCGCTGGCGAAGGTCAGAAAATGAAACGTT
CGACCCCGGAATCCCTGACTTCGACTCCGGAGTGGGTCGATTTCCCGTCGGCGGTTGCTTATGGGCAAAA
CGAAGCGGTGTCTTTCCACGCTGGAGCCTACGGAGAAAACGCCTCCGGCATCAGCGACGCCGGCCGTGTT
GCCAGAATACTCCATTCATGTAGAGCCCTGGCCATTCGCAGTTGCCCCGAGTTCGAACACGATTACTTGA
ATCTACTCGAGAAAATCCATCAAAAACCGGTGATTCCCGTCGGACTACTCCCGCCAGAACAAAAATCGGA
AGCTAGAAACTCCGGCGATAAATTCAAAATCTTCAAATGGCTTGATCAACAAAAACCCAAATCGGTTATC
TTCGTCGGATTCGGTAGCGAATGCAAACTAACCGAAAATCAAATTCACGAAATAGATTACGGTCTGGAGC
TGTCCGAACTCCCATTTCTATGGTCGCTCAGAAAACCCAGCTATTCAACCGACGAGCTCGACCCTCTTCC
GCCGGATTTCCACCGCCGCATCGCCGAAAAAGGACTAGTACACGTCGGATGGGCGCCGCAGACGGAGATT
CTCGCCCACCCATCCGTCGGAGGCTCTCTGTTTCACTCCGGCTGGGGATCGGTGATCGAGACTCTGCAAT
TCGGACACCGTCTCGTCGTCTTGCCGTTGATTATCGATCAGCCGTTGAACGCGAGGTTGCTGGTGGAGAA
GGGTTTGGCAATAGAAGTGGAGAGAGGCGGAGATGGTTCGTTTGGCGGGGACGCCATAGCTAAAGCTCTG
AGAGAAGCCATGGTTATGGAAGGAGGAGAGAGAGTGAGAGAAGCTATGGCGATATTCGGAGATCGGAAGT
TGCAAGAGAACTACGTGGAGAGATTTGCCGAATACCTGAAAAATGGAGTGGCAAAAAAGAATCGGTAA
>GWHPAAAL014233
ATGGAGGAGAGGAGAGAGAACATAGTGATGTTCCCATTCATGGCGCAAGGCCACATGATTCCATTTTTGG
CCCTGGCCTTGCAATTGGAGAAAAAGGGTTACGGCATAACCTTTGTCAACACTCCTCTCAACGTCCTGAA
ACTCCGACAGTGTCTCCCACCGGACTCCTCCATTCGCATCCTTGAAATTCCGTTCAACAGCTCCGACCAT
GGTCTTCCTCCCAATTCTGAGAACACCGACGTCCTCCCCTACACCCTCATCGTCAACCTCCTCCACGCCT
CTCTCTCTCTGGAATCCTCCTTCAGGAAACTTCTCTCTGATCTTATCCACGAGCAGGGTGGCGTCCGTCC
GCTTTGCGTCATCGCGGATATATTCTTTGGGTGGTCGGCCGGCGTCGCGCGTGAGTTAGGCGTATTCCAC
GCGATTTTCAGCGGCGCCGGAGGGTTTGGATTGGCGTGTTACTATTCTGCGTGGTTGAGTTTGCCACACC
GGACAACGGATTCTATCGAATTCACGCTGCCGGATTTCCCTGAAGCCAGTAAATTTCATGTCACCCAGCT
GCCCGCGAGTATGCTGGCGGCTGACGGCACTGATCTGTGGTCGATTTTCCAGAGAAAAACCCTTCCGGCG
TGGACCAATTCGGACGGAGTTCTGTTTAACACAGTCGAAGGGTTCGACAAGCTCGGATTGGCATATTTCA
GTCGCAAAATCGGGAAGCCAATCTGGGCGATTGGACCAACACTTCTATCAGTGCACCACAGAGCTCGTGT
CGGCGGTAGAGAAGCCGCCATTACTCCAGAGCAATGCGCCGAATGGCTTGATTCGAAGCCACCCAACTCC
GTAATCTACGTATCATTCGGATCACAAAACACAATCTCAGCATCCCAGATGATGCAATTGGCGAAGGCTC
TGGACAAAAGCGAGAAGAATTTCATCTGGGTTGTTAGACCCCCACTTGGGTTCGACATAAATTCAGAGTT
TAAACCAGAAGAATGGCTGCCGGAAGGGTTTACAGACCGGATCAAAGATCAAAACAGAGGTCTAATCGTT
ATAAAATGGGCGCCCCAGGTGGAGATTTTGTCGCATAAATCTATAGGGGCGTTTATAAGCCACTGCGGAT
GGAATTCGGTGCTTGAAGCGTTGAGCAATGGGGTGCCGCTGATAGGGTGGGGCATGGCGGCGGACCAGTT
CTTCAACGTGAAGATGTTGGAGGAGGCGGTTGGAGTGTGCGTGGAGGTGGCGAGAGGGACCATTTTTGAG
GTGAGGCATGAAGATATAATGGAGAAGATAGAGGTGGTTATGGGGGAGAGTGAGAAGGGGAAGAGAATAA
GGAAGAGAGCTGGAGAATTGAAGGAGATAATCAAGGATGCAACGAGAGATGAGGAGGGCTTCAAGGGTTC

TTCCGTGAAAGCCATGGATGAATTTTTAAGTGCTGCTGCAATGTCGATGAAGGAGAAGAAGACATTATTG
GTTGAGACACAATGA
>GWHPAAAL014996
ATGGAGATAACGGATGGAGAAACAGGGTCGTCCCAAAATCGCGTCCTGGTTATTCCATTTCCGTCACAAG
GCCACATCAATCCCATGCTCCAATTCTCCAAGCGTTTAGCCGCCAAAGACCGCCGCCGCAGCCTTGGGAT
CACCTTAGTCACCACTTCCACCACCAAATTCTCCGCCCTATCTCAACCACCTACCTCCGTCTCTGTCGAA
TATCTACCCGATGACTCCGACGATGATCAACCGGATGACGTAGGAGCCTTCTTCCGGCGTTTCGAGAAAA
CAGTCACCGAGGGACTGCCGGGGCTAGTGGAGAGGCTAAAAAACTCCGGCAGCCCCGCGAGAGTGGTGGT
GTATGATTCCATCATTCCATGGGTTCTAGACATAGCCCATCCGCTGGGCTTGAAAGGGGCTTGCTTCTTC
ACTCAGACCTGTGCGGTGTGCGCTATCTACTACCACGTGCACCGAGGAACACTCACAGTTCCTTCCGAGG
AAGAGGAGGAAGACCATCGATCATCTGTCACGCTTCCTGGGATGCCGTTGATGGAGATCAAAGATCTTCC
TTCTTTTGTCTACGACATCGACCAGTATCCTTATGCTTTGCGCTTGCTTCTTGCTCAGTTCTCCAACACC
GGGGAAGCAGAATGGATCTTTTTCAACACTTTCGACAAGCTAGAACATGAGGTGATAGACAATTGGGCGG
CAAGAGAATGGCCAAGAATGAAGAGCATTGGACCAACAATTCCATTGGCGTACTTGGAAGAAGAAGACAA
CACACTCCAAAACGACATAGATTATGGGCTTAATCTCCTAGACTCAAACTTCGAAGCCTGCATGAATTGG
CTGGACGCCAAGGAAACCGCATCTGTCGCCTATGTATCGTTTGGCAGCGTCGCCAACCTCAACAACGAGC
AAATGGAGGAACTAGCTTGGGGCTTGATAAGTAGTGGAACCCACTTCCTGTGGGTCGTCAGAGCTTCCGA
AGAAAGCAAACTTCCACCCAATTTCACCGATCAGGCTTTAGAAAACGGTCTAATAGTGAATTGGTGTCCC
CAACTCCAAGTTTTGGCTCACCGGGCGGTTGGGTGTTTCGTGACACACTGCGGTTGGAACTCGACGCTGG
AAGCACTGAGTCTTGGGGTTCCGCTAGTGGCGATGCCTCATTGGTCGGATCAGACAACAAATGCAAAGTA
TGTTTCCGATGTTTGGAAAGTCGGGGTTCGGGCAAGAGGGGTCGAGAAAGGGGCAGGAGTTGTGGGGAGG
GCGGAAATAGAGAGGTGCGTAAAGGAAGTTATGGAGGGAGAGAGTGGGGATGAGGTGAGGAGGAATGCAG
TTAGGTGGAAGGAGTTGGCTAGAGAGGCTGTGGCTGCAGGGGGAAGCTCTGATAACAATATTCAAGAATT
TGTCTCTCAACTTGTTAAACTATGA
>GWHPAAAL015368
ATGACCAACACTAACAGAGTAGAGCTCGTCTTCATCCCTTCACCGGGGATGGGTCACCTCGTCTCTGCCG
TCGAGATGGCCAAGCTTCTCGTCGATCGAGACCAACGTCTCTCCATCACCCTCCTCATTATGAAAGCACC
TTTCAAAACCAAGGCCAATTACTCGCCGGCCCAATCGCTCTCCACAGCTGCGGCCTCTCGAATACGGTTC
GTGGATCTCCCAGGCGACGAATTGGAGTCCGACGCCAAATTCTCCCCTCACACCTTCCTTTCCGGCTTCA
TCTCCGGCCAGAAGACGCTGGTTAGGGACGCCGTTGCGCAAATCTCCTCTTCTCCAGAAGCTCCCAGACT
CGCCGGTTTCTTCATCGACATGTTCTGTACTTCCATGATCGACGTTGCCGATGAGTTCCGTGTCCCGACC
TACGTGTTCTTCACTTCCGGCGCCGCATTTTTGGGACTCGTGTTCCATCTCCAAACCATGAGAGACGACC
ACAATCACGACGTCACCGAGTTAAAGGACTCCGATGCCGACTTACTGGTCCCATGTTTCGTCAAATCGGT
CCCGACTAAAGTACTGCCGTCGGTGGTGCTCGATAAGGAAGGTGGGTCTGAGATGTTCCATGATCTCGTC
CGACGGATCAGGGAGACAAAGGGCATCTTGATCAACACGTTCTCCGAGCTCGAATCTCACGCGATCCATT
CCATCAACACCCCGCCTGTCTACCCGGTGGGACCCATCCTGAACGTCGCCGGCGACAATGACAACGAATC
CACCTCCGCCGCCGCGATCCTGAAGTGGCTCGACGATCAACCGCCGTCGTCGGTTGTCTTCCTATGTTTC
GGAAGCATGGGATGCTTCGGCGTTGACCAAGTAAACGAGATCGCCCACGCCCTGGAACGCAGCGGCCACC
GCTTCCTGTGGTCCCTACGGCGGCCGCCGCCCGAGGGGAAGCTAGAGTTCCCCGGAGATTACACGAATTT
GGAGGAGGTTTTACCGGAACGGTTTTTGGAAAGGACGGCGGAGATCGGGCGAGTGATAGGGTGGGCGCCG
CAGGCGGCAGTACTGGCTCACAGGGCAGTGGGAGGGTTTGTGTCGCACTGCGGTTGGAATTCAACGCTGG
AGAGTGTGTGGTACGGGGTTCCAATGGCGGCGTGGCCACAGTACGCCGAGCAACAGCTGAACGCCTTTCT
GATGGTGGAAGAGTTGGAGATGGCGGTGGAGATTAAGATGGATTATAGAAACGGATTTTACTCAAAGAGT
GGAGTTGCTTTGAGGAGCGAAGAGATAGAGAAGGGAATACGGCGGTTGATGGAGTGCGGTGAGAATGGGA

TGAGGAAGAAGGTGGAAGAGATGAGGGAGAAGAGCCGAGTGGCGGTGAGGGAAGGCGGATCTTCTTACCT
TTCTATTGGGCGTTTGATCGAGGATGTAATGAAAATAGTTGAACAATGA
>GWHPAAAL015516
ATGGCCTACCATCACCCCCGCCAAACGCAACCGCAGGTGGCGGTTGTGGTGGTTCCTTTCCCCGCACAAG
GCCATCTCAACCAGCTACTCCAACTCTCCCGCCTCATCTCCTTCTACCACATACCCGTCCACTACGTCGG
TTCCGCCACCCACAACCGCCAAGCCAAACTCCGCAACCACGGATGGGACCCTCTCTCCGCTACCAACATC
CATTTCCACGAATTCTCAACTCCCCATTTCCTCTCTCCGCCGCCGGATCCCCACGCCGCCGTCAAATTCC
CCTCCCATCTCCAACCCTCCTTCGAAGCCTCTTCTCATCTCCGCCGCCCCGTCGCCGCTTTAATGCGCGA
ACTCTCCCAAAAATTCCGACGAATCATCATCGTCAACGATTCCCTCATGGGATCGGTGGTTCAAGACTTC
GTTTCACTGCAGAACGCCGACGCCTACACTTTCCACAGCGTCTCCGCTTTCGCCATGGCTTTGTTCTTCC
GGGAAAGCACCGGAAAACATTTCTCCGTCCATGCAGAACTACTAAACGGCGTCCCGTCGCTCGACGGCTG
CTTCACCGCCGACTTCCTTAACTTCCTCAAAACCCAACATTACTACACCCAGTTCAGCTCCGGCCGAATA
TACAACGCGTGCAGATTAATCGAAGCTCCATTTCTCGACATACTTTCTCAAGATCACGACACCGCCATTA
ATAAGAACAAGAAGCATTGGGCTCTCGGCCCTTTCAATCCCATCTCCATAACCCATAATAAGAGACCCAA
TTCACGCCATAAATGCTTGGAATGGCTCGACAAACATGATCAGAACTCCGTCATATTGGTTTCTTTTGGC
ACCACGATATCCTTAAGCGAAGAACAGATCAAAGAACTCGCGACTGGGTTAGAACAAAGCGGTCAAAAAT
TCATCTGGGTATTAAGAGATGCAGACAAAGGAGATGTTTTCAACGAAGAAGATGAAGTTAGGAGGGCTGA
GCTTCCAAAAGGGTTTGAAGATAGAGTAGAGGGGAGGGGTTTGGTGGTGAGGGAATGGGCGCCGCAGTTA
GAGATTTTAGGCCACCCTTCCACCGGCGCTTTCATGAGTCACTGCGGTTGGAATTCTTGTATGGAGAGTA
TTACAATGGGAGTTCCCATGGCGGCTTGGCCGGTGCATTCCGACCAGCCGAGGAATGCAGTGTTGGTCAC
TAAGTTTCTCAAGATTGGGTTGCCGGTCAAGGACTGGGCTCGTCGGGATGAGGTGGTGATTGCTTTGACA
GTGGAGAACGCCGTGAAGAAGCTAATGGCGGCGGAGGAAGGGCGGGAGATGAGGAAGAGGGCGGAGGAAC
TCGGTGGTGCGGTCCGGAGATCGGCGGCGGAAGGCGGCGCAAGTTGTAAAGAAATGGATGCGTTCATCGC
CCACATTACCAGAATCTAG
>GWHPAAAL015754
ATGTCCGGAGATCGTAAGCTACACATAGTCATGTTCCCATGGCTTGCCTTTGGTCACATGATCCCATATT
TAGAGCTCGCCAAGCTCATTGCCGAAAAGGGTCACAAAATCTCCTTCCTTTCCACCTCCAGAAACATCGA
TCGTCTCCCCAAATTGCCCCCACATCTAACTCCCGTCATAGACTTCGTCAAGCTCCAGTTGCCCCACGTC
GAAGGCCTCCCAGAAAACGCCGAGGCCACCTCCGACGTTCCTTATAACAAAGTCAAATACCTCAAAGTGG
CCTACGATCTTCTCCAAGAACCCATCGCTCGATTTCTTCGAATCTCGTCTCCCGATTGGGTGCTTTTCGA
TTTTGTTTCTTACTGGTTGGGTCCGATTGCTTCTGAATTGAGGATTCCAAGCGCTTTCTTCAGTATATTT
ATTCCGGCTTTCTTGGGGATTTTCGGCCCCGTACCGGTACTAAAGGGTACGGATGAGGCTCGAACGAAAC
CCGAGGATTTCACGGTCTCGCCTAAATGGGTTCCTTTCAAATCGACGGTCGCGTTCCGGCTATTTGAGAT
TCAGCGTATTTTCGACAGCGTTACCGGCGACGAAATGAACGTTCCCGACACCTATCGCGCTGGATGCACC
ATCGAGAGCTGTGACATAATTGCTATAAGAAGCTGTCACGAGTTCGTACCGGAATGGATAAAGGTTCTCG
AGGAGATCCACCAGAAACCGGTTATACCGGTGGGTCAACTGCCAACCACGGCGTACGATGGTGGCGAAGA
CGACAAGGACGGCCAGTGGGAAAAGATGAAAGAGTGGCTCGACACAAAGGAAAAAAAGTCCGTCGTCTAC
GTTGCGTTTGGCAGTGAGGCGAAACCGAGTCAAGCTGAACTCACTGAGATAGCTCTCGGGTTAGAGCTAT
CCAGGTTGCCATTCTTTTGGGTCCTCCGGAACCGACGGGGTAACGCCGACACCGAGCCGATCCAGCTGCC
AGAAGGGTTCGAGGAGCGAACCAGAGGTCGCGGGTGGGTCTGCACGAAATGGGCTCCTCAACTGAAGATA
CTAAGTCACGACTCGGTGGGAGGTTTCCTGACTCATTCGGGCTGGAGTTCGGTGGTGGAGGCCGTACAGT
TCGAGAGAGCTCTGATACTGTTGACCTTCTTGGCGGACCAAGGGCTGAATGCTAGGGTTCTGGAGGAGCA
GAAGATGGGTTATTCGATTCCGAGAGACGATCGAGATGGGTCGTTCACGCGGGACTCGGTGGCTAACTCA
GTGAGGCTGGTGATGGTGGAAGAAGGAGGAAAGATTTACAGAGAGAAGATGAAGGAGATGCTAGGAGTGT

TTGGAGACAGGGATAGACAAGATGGTTACGTCGAGAATTTTCTACGTTATCTCCAGAATTATAAAATACC
GACAAGCGAAGATCCAAACAAGAAAGATTCAATTTTTATTTTTATTTTTCTCCATTCTCCCTCGTTCGTC
TTTTCTCTGCCCCTCGCCATTCTCTTCTCCAAGCCAGCCAACGCCCCTCTAGTCGGCTTTGGGGGGGATG
TTGTGAACGCTGCCGGGTGGGTCTCACTTCCCGTGACCGTCGGAGCCCATCCACGAACCTCAACAGTTTG
GACCAAATTTTTGATCGTGGACAAGCCGTCGCCCTATAACGCGATCATGGGTCGACCCACGATGGCGGCC
CTCAAGGCCGCCTATTCACCATACCACCAGTCGATGAAATTCCCCACGGAGGGAGGTGTGGGAGTAGTGA
AGGGTGACCAAGAGTCCTCTCGGTTTTGTGCTCGGAGGGCGTTAGAGAAAAAGAAATAG
>GWHPAAAL015755
ATGGAATTAATAATCCCCAGCAACGGCCGAAAGCTTCACATAGCCATGTTCCCATGGTTAGCTTTCGGAC
ACTTGATCCCTTTCTTAGAGCTGGCCAAGCTCATAGCCCAAAAGGGTCACCAAATCTCCTTCATTTCCAC
ACCCCAAAACGTCCACCGCCTCCCAAAACTCCCTCCAAATCTCACTCCCCTCATAAATTTCGTTCAAATC
CCACTGCCGGCCGTCGAAAACCTCCCGGACGGAGCAGAAGCCACCATCGATGTTCCCTACGATAAAGTCA
AATACCTGAAAATCGCCTATGACGGCCTCCAAGAACCCATCACCAAGTTTCTTCATTATTCATCTCCGGA
TTGGATAATTTTCGATTTCGCTTCTTATTGGGTAGGCCCGGTAGCCTCTGAACTCGAAGTTTCCTCTGCT
TTTTTCAGTATTTTCACGGCCGCTTTCATGGGTTTCTGCGGGCCCGCAGAAATTCTAAAGGGGACTGTGG
AAGGACGCACCAAACCGGAGGACTTCATCGTCAAGCCGAATTGGGTGCCATTCGAATCGAGGGTTGCGTT
CCGGCTGTTTGAGATTTTGCGAGTTTTTGACGGCGTAACAGGGGATGAAGACAACGTATCGGATGGGTTG
CGGATGGGTTGCACAATAGAGGGCTGCGATTTAATCATCATCAGGAGTTGTTATGAGTTCGAGTCCGATT
GGTTAAGGGTCATTGAAGAGATTCACCGAAAACCGCTAATTCCGGTGGGTCAGCTGCCGACCACCGTCTA
TGATGGCGGAGACGTTGACAAAGACGGCGCGTGGAGAGAAATCAAGGATTGGTTCGACCAGCAGGAAAAA
GGATCAGTGGTTTATGTTGCGTTCGGAAGCGAGGCGAAACCCAGTCAAACCGAACTCACAGAGATAGCTC
TGGGTTTGGAGGTATCCGGGTTGCCTTTCTTCTGGGTTTTGAGAAAGCAACGAGGATCAGCCGACATCGA
GTTGACGCAGTTGCCGGAAGGGTTCGAAGGGAGAACCAGAGGGCGCGGAATCGTGTGCACCACTTGGGCT
CCGCAGTTGAAAATACTGAATCATGTCTCGGTGGGGGGGTTCTTGACTCACTCCGGGTGGAGTTCGGTGG
TGGAGGCCATTTCTCTCGAGAAGGCACTTATTTTGCTGACGTTCTTAGCAGACCAAGGACCGAATGCTAG
GCTTCTGGAGGAGAAGAAGATGGGTTACTCAGTACCCAGAGACGAGCGAGACGGTTCGTTCTCGAGAGAC
TCGGTGGCTGACGCACTGAGGTTGGTGATGGCGAGCGAAGAAGGGAAGATTTACAGAGACAAGGTCAAGG
AAATGAAATGGGCGTTCGGAGACAGGGATTTACAGGACCGTTATGTTCACAATTTATTGGGTTATCTTCA
AACTCATAATAGGAAGAAGGGGAGATGA
>GWHPAAAL016884
ATGGCAGCATCAACTTTCCACATAGCAATGTACCCCTGGTTTGCTCTTGGCCACCTCACCGCTTTCCTCC
ACCTATCCAACAAACTAGCCAAAAAGGGTCACAAAATCTCCTTCCTCATCCCCACCAAAACACAGCCCAA
ATTAAACCCTTTCAACCTCTACCCCAACCTCATCACCTTCATCCCAATCACCATTCCTCATGTGGACGGC
CTTCCTCCTGGCGCCGAGACCACTTCCGACATCCCGTATCTATCAGGGACACATCTCATGACAGCCATGG
ACCAGACTCAAGCCCATATCGAACTCCTCCTCCGTGACTTAAAAGTGGACATTGTCATCTTCGATTTTGC
TTACTGGATACCGGCAGTTGCAAAGTGGTTAGGGATCAAGTCGATTCACTACTGCACCATCAGTCCAGCT
ACAGTAGCTTACACGATGGTACCGGCTAGAAATCTTAAAGGAAACAAAGTAACGGAAACTGACTTGATGC
AGCCGCCAGAAGGATACCCAGTTTCATCGATCAAGCTTCATGCTCACGAGGCACCTGCGTTCGTTGCTCG
GAGAAGTATGAGATTCGGGGGCGACAAGCAATTCTATGAGCGACAATTCCTCAGTTTCAGCCAATGCGAC
GCGTTGGGTTTCAGGGCATGTAGAGAAATCGAGGGCCAATTTTGTGACTACATTGAAAGCCAATTCAGAA
AGTCAGTGCTGCTTTCAGGACCTGTTATACCAGAGCCGCCGACATCTCCTTTGGAAGAAAAATGGGTCAA
ATGGCTAGGCAAATTCGAGGCAGGAACAGTTGTTTACTGCACATTCGGGAGCGAATGCACCTTGAAGAAA
GACCAATTCCAAGAACTGGTGCTGGGTTTGGAGCTCGCCGGAAAACCGTTCCTGGCAGCACTTAAACCAC
CTGCCGGAGTTGAATCTACAGAAGCAGCATTGCCAGAGAACTTCAAGGAGAGAGTTCAAGGGCGAGGGAT

AGTTCACGGGGGATGGGTTCAGCAACAACTGATTCTGGAACACCCTTCGGTTGGGTGCTTCATTACACAC
TGTGGGTCGGGTTCTGTGTCTGAAGCACTGGTGAATAAGTGTCAGATGGTGTTATTACCACACGTAGGTG
ACCAGATTATTAACGCGAGGATGATGAGTCGGGATCTAAAGGTGGGCGTGGAAGTAGAGAAAGGAGAAGA
AGATGGGGTATTCACGAGGGAGAGCGTGTGCAGAGCTGTGAGGGCCGCAATGGAAGAAGGAAGTGAGGTT
GGAAAAGAGATCAGAGCCAACCACGCCAAGCTGAGAGAATTCTTGTTGCACAAAGACTTGGAATCCTCTT
ACATTGAAAATTTTCTTATGAAACTCCAAGAATTACTACAATGA
>GWHPAAAL016885
ATGGCAGCATCAAGTTTCCACATAGCAATGTACCCCTGGTTTGCCCTCGGCCACCTTACCCCTTTCCTCC
ACCTCTCCAACAAACTAGCCAAAAAGGGTCACAGAATCTCCTTCCTCATCCCCACCAAAACACAGCACAA
ACTAAACCCTTTCAACCTCCACCCGGACCTCATCACCTTCATTCCGATCACAGTCCCTCACGTCGACGGC
CTTCCTCCTGGGGCCGAAACCACTTCCGACACCCCTCCCCAATCTCTCACACACCTCATGACCGCCATGG
ACCAAACCCAACACCACATTGAACACCTCCTCCGTGACTTAAAACCGGACGTCGTCTTCTTTGATTTCAC
ATACTGGATACCAGAAGTAGCAAAGCGGTTGGGGATCAAGTCGATTCACTACTGCATCATCAGTCCGGCC
ACAATAGGATACACGATGGTACCCACGAGAAATCTCAAAAGGAAAAACGAAGTGATGGAAATTGACATGA
TGCGGCCACCGGAAGGGTACCCGGCTTCGTCGATCGAGCTTCATGCTCACGAGGCACGGGCGTTTGTAGC
TCGTCGGAGACGTGTAGCGAAATCGAGGGCCAATTTTGTGACTACATTGAAAGCCAATTCGGAAAGNACA
TCTCCTTTGGAAGAAAAATGGGTCAAATGGCTAGGCAAATTCGAGGCAGGTACAGTTGTTTACTGCGCAT
TCGGGAGCGAATGCACCTTGAAGAAAGACCAATTCCAAGAACTGGTTCTGGGTTTGGAGCTCGCCGGAAA
ACCATTCCTGGCAGCCCTTAAACCACCTGCCGGAGTTGAATCTACAGAAGCGGCATTGCCAGAGAACTTC
AAGGAGAGAGTTCAAGGGCGAGGGGTAGTTCATGGGGGATGGGTTCAGCAACAACTCATTCTGGAACACC
CTTCGGTTGGGTGCTTCATTACACACTGTGGGTCGGGTTCTTTGTCTGAAGCACTGGTGAATAAGTGTCA
GCTGGTGTTATTACCACACGTAGGTGACCAGATTATTAACGCGAGGTTGATGAGTCGGGATCTAAAGGTG
GGCGTGGAAGTAGAGAAAGGAGAAGAAGATGGGGTATTCACGAAGGAGAGCGTGTGCAGAGCTGTGAGGG
CCGCAATGGAAGAAGGAAGTGAGGTTGGAAAAGAGATCAGAGCCAACCACGCCAAGCTGAGAGAATTCTT
GTTGCACAAAGACTTGGAATCCTCTTACATTGATAATTTTCTTATGAAACTCCAAGAATTACCACAATGA
>GWHPAAAL017403
ATGGAGAGAGAAATAGATGAAATGGGTAGCCAAAAACCTCATGTTCTGGTAGTCCCATTTCCAGCACAAG
GTCACTTCAGTCCAGCGCTGCAACTCTCCAAACGCTTAGCCTCGAAAGGGATTAGGGTCACTTTCGTCAC
CACCATTAGCAGCGCCGAGTCCAGTGGCTCCATTAGGATGGAGCACATTAGCGACGGCTATGAAGAAGGT
GAAAAACCAGAGAGCATTGAAGCCTTTACCGACCGCATCAAAACTGTGTTCTCGCAAAACCTTGGCGATC
TAATTGAGAAGCAAAAGCGAGTCGGTTACCCAGTCAAGCTTCTAATTTATGATTCTGTGATTCCATGGGC
ACTAGAGATAGCACACAGTTTAGGGGTTTATGGAGCTCCATTCTTCACTCAAACCTGTGCTGTCTGTGCT
ATCTACTACCATGCGCAAAAAGGGAATCTGGAGGTTCCTCTTGAAGGAACCAAATTCTCACTACCTTCGT
TGCCACCATTGGAGAGAAATGATCTTCCATCTTTCCTTTCTGACATGAACTCCTATCAATTTTTCTTGAA
AATGGTCTTGGCCCAATTCTCAAATTTTCAAAACGCAGATTGGATCTTATTCAACACCTTCGACAAGCTG
GAAGATGAGATAGTGAAGTGGATGGCAAGCCAATGGCCTATCAGAACCGTCGGACCAACTATTCCATCCA
TGTATTTAGACAAGCGACTGAAAGAAGACAAAGAATATGGCCTCAGCCTCTTCACTCCAAACATCGAGTC
TAGCATGAAGTGGCTGGACTCTAAGGAACCAGGTTCGGTGGTTTATATCTCGTTTGGGAGCTTGGCCAAC
CTTGACGAAGGTCAAATGAAGGAACTATCATGGGGCCTTGTGAAGAGCAACTATTACTACCTGTGGGTGG
TCAGAGATTCCGAAGAGAGCAAGCTTCCCAACAATTTCATGGCTGAGACTTCAGAGAAAGGGCTCGTGGT
CAGTTGGTGCCCTCAGCTTGAGGTCTTGGCTCACCAGGCAGTGGGGTGTTTCGTAACTCATTGTGGTTGG
AACTCGATGCTTGAAGCTTTGTGCCTCGGGGTGCCAATGGTTGCAATGCCCCAGTGGACTGATCAAACGA
CGAATGCAAAGTTTGTGGCCGATGTGTGGAGGGCAGGGGTTCGAGTGGAGGCCAATGATGAGGGGGTTGT
CACGAGAGAAGAAATTGAGAAACGAATCAGAGAAGTCATGGAGGGAGAAACAAGGGAAGAACTGAGAAAG

AATGCTAGCAATTGGAAAAACCTAGCGAGAGAGGCAGTGGATGAAGAAGCAGTAATTTTCCCAGCAAATC
CTTCTCAGAAAAACAATCCCATGACCCAATTCTCCAAGTGTTTAGCCACCACTGGCCACCGCCTTGCGGT
CACCCTAGTCAACACCACCGCCACCAAATTCTCCTACCTATCCCAACCACCTAGCTCCATCTCTATCGAA
CGGATGTATCTAATGATGATCAACTAG
>GWHPAAAL017405
ATGGAGATAACGGATAGAGAAACAGGGTCGTCCCAAAATCGCGTCCTGGTTATTCCATTGCCAGTCCAAG
GCCACATAAATCCCATGCTCCAATTCTCCAAGCGCTTAGCCGCCAAAGACCGCCACTGCCGCCTTGGGAT
CACCCTAGTCACTACTACCGCTACCAAATTCTTCGCCCTATCCCAACCACCTAACACCATCTCTATTGAA
TCTCTACCCGATGACTCCGACGATGATCAACTGGTTGACACGGTAGCCCTCATCCGGCGTTTCGTAAAGA
CAGTCACCGAGGGACTGCCGAGGCTAGTGGAGAGACTGAAAAACTCCGGCAGCCCCGTAAGAGTGGTGGT
CTATGATTCCTTCATTCCGTGGGTTCTAGACATAGCCCATCAGCTGGGCTTGAAAGGGGCTTGCTTCTTC
ACTCAGACCTGTGCGGTGTCCGCTGTCTACTACCACGTGCACCAAGGAACACTCACAGTTCCTTCTGAAG
AAGAGCAAGACCATCGATCATCTGTGACGCTGCCTGGGATGCCGTTGATGGAGATCAACGATCTTCCTTC
TTTTGTCTACGTCGTCTACGACCAGTATCCTGCTGCCTTGAGGTTCCTTCTTGCTCAGTTCTCCAACGCC
GGGAAAGCAGATTGGATCCTTTTCAACACTTTCGACAAGCTAGAACATGAGGTGATAGAAAATTGGGCGG
CAAGAGAATGGCCAAGAATGAAGAGCATTGGACCAACAATTCCATCTGCATACTTGGAAGAAGAAGATAA
AACACTCCAAAACGACAAAGATTATGGGCTTAATCTCCTAGACCCAAACATCGAAGCCTGCATGAATTGG
CTGGACGCCAAGGAAACCGCATCAGTTGCCTACGTGTCGTTCAGCAGCGTAGCCGACCTCAAAAAAGAGC
AAATGGAGGAACTAGCTTGGGGCTTAATAGGTAGCGGAACCCACTTCTTGTGGGTCGTCAGAGCTTCCGA
AGAAAGCAAACTTCCACCCAATTTCACCGATCAGGCTTTAGAAAACGGCCTAATAGTGAATTGGTGTCCC
CAACTCCGAGTTTTGGCTCACCGGGCGGTTGGGTGTTTCGTGACACACTGCGGTTGGAACTCGACGCTGG
AAGCACTAAGTCTTGGGGTTCCGCTAGTGGCGATGCCTCAGTGGTCCGATCAGACGACAAATGCGAAGTA
TGTTTCCGATGTTTGGAAAGTCGGGGTTCGGGCAAGAAGGGTCGAGAAAGGGGAAGGAGTTGTGGGGAGG
GAGGAAATAGAGAGGTGCGTTAAGGAAGTTATGGAGGGAGAGAGCGGGGATGAGGTGAGGAGGAATGCTG
TTAGGTGGAAGGAGTTGGCTAGAGAGGCTGTGGCTGCAGGGGGAAGCTCTGATAACAATATTCAAGAATT
TGTTTCTCAACTTGTTAAACTATGA
>GWHPAAAL017961
ATGGAAACAAGAAGAGGTCCGAAAGAGGTGGTGTTAGTCCCCTATCCAGCACAAGGCCACATGAGCCCCA
TGCTCCAACTCGGCACCCTCCTCCATTCCCTTGGCTTCTCCATCACCGTCGCCTACGCCAGCCTCAACTC
GCCGGACCCGTCGACCCACCCTGAGTTTGATTTCCTACCCATATCGGAGGACTTGTCGGATCGAGACACC
TCATCCGGGGCCGTGTTCTCCCTCATCACGAGTCTCAACGCCGACTGCGCAGAGCCACTCCGGGAATGTT
TGGTTCGGGTGACGGAGGATCGGGGAGCAGTCGCCTGCGTGGTGTACGACTCGCTCATATATTTTCCCGA
GGAGGTGGCGCACCGGATGAAGATCCCCAGCATGGTGTTGCAGACGTGTAGCGCTGCTTATGTGTTGGCT
TGCCGGCTTTTTCCCGAGCTCAAGGAAGAAGGCTATCTTCCTGCTGAATCTGAAGATCCTTCAATGGAGG
ACCTTGTGCCGAAGCTTCATCCCCTCAGATTCAAGGATTTACCCATTACTAAATTCGACACAACCGCCCT
ACAAATACTCATGGATCTAAACGACGTTGGCACGTCTTCCGCAGTCATCTGGAACACCACCGACTACCTC
GAGCACCCCTCCTTATGCCGACTCCGGCAGCATTACCAAGTACCCATCTTCCCGTTAGGCCCCTTGCAAG
CAATGGCTCCCACCTCTTCCACCAGCTTCCTTAAAGAGGAAAACGGTTGTCTAAATTGGCTCGAAAACCA
GGCACCAAAATCGGTTATTTATGTGAGTTTCGGAAGCAACGCGACAATGGAGCCAAAAGAGCTGAGCGAG
GTGGCCTGCGGTTTAGCCAATAGCGACCAACCATTTTTATGGGTGGTTAGACCTGGTTCGGTTAAAGGGT
CGAAATGGGTGGAGCACTTGCCGGAGGGGTTTATAGAGGCTGTGGGAGAGAGAGGGTGTATCGTGAAGTG
GGCTCCACAGAAGAAAGTGTTGGCGCATGGGGCGGTGGGAGGATTCTGGAGTCATTGCGGCTGGAATTCG
AGCTTGGAGGGTCTTTCCGAAGGGTTACCGATGATTTGCAGGCCGTGTTTTGGTGATCAGAGAGCAAATG
CTAGGTATTTGGCTCATGTTTGGAGGGTGGGATTGGAATTGGAACTTGAATTGGAGAGAGGAGCCATTGA

AAGAGCTGTGAGAAGGCTTATGACGGGTAAAGAAGGGGAAGAATTAAAGAGGAGGGCGATGGATATGAAG
CAAAACATTGAAGAAAGTGTGCAGAAAGATGGATCTTCTTACAATTCCCTGATTGAGTTGGCAGATTTCA
TCTCTGATTTTCACAAGACACGGCAATGTGTTTAA
>GWHPAAAL018171
ATGGGTAAGCAGCTTCATGTTGTCTTCTTCCCGTTCATGGCGCACGGCCACATGATTCCGACGCTGGACA
TGGCCAAGCTCTTCTCCTCCAGGGGTGTCAAAACCACCATCATCACCACCCCTCTCAACGCCCCTGTTTT
CTCCAAATCAATCCAGAGGTCCAACCAATTGGGTCTCGATATCAGCTTAAAAATCTTTGATTTTCCGGCG
GTTGCCGCCGGCTTGCCGGAAGGATGCGAGAGCGCTGACCAAATCACCTCCGAGGACATGTTGCCAAAAT
TCTTCGTCGCCACGGCGTTGCTCCGGGAGCCGCTGGAGCGGCTGCTGAGTGACCACCGCCCGGATTGTTT
AATCGCCGGCATGTTTTTCCCTTGGACGACGGAGTCGGCGGCGAAATTCGGCATTCCAAGGTTGGTTTTC
CACGGGACCAGTTTCTTCGCGCTCTGCGCCGGGGAGTCTCTGAGGACGCACAAGCCTTTCCGGCAAGTCA
CGTCGGATTCCGAACCCTTTGTCCTGCCAGAGCTCCCGCACGAGATAAAGCTGACGAGAACGGAGGTGTC
GCCGTTTGAGCTGCACGATACCGAAACGGCCATATCCGGAGTCTTGAAACAAGTGAGCGAGTCGGAGAAA
ACTTGTTATGGCATTATCGCAAACACCTTCTATGCACTCGAACCAGATTACGCCGATCACTACAGAAACG
CCATGGGGAGAACGGCGTGGCACGTCGGACCTCTCTCTCTCTGCAACAGAGGAATCGAAGACAAAGCAGA
GAGAGGAAAAAAATCTTCCATCGACGAACACGAATGTCTTCAATGGCTTGATTCAAAGAAACCCAAATCC
GTCCTCTACGTCTGTTTCGGAAGCATGGCCAAATTCGCCGATTCCCAACTGTACGAAATCGCCGCCGGTC
TTGAATCCTCCGGCCAACAGTTCATCTGGGTGGTGAGAAAATCAAAAGACGAAGAAGAACAGAAGGAAGA
GACATGGCTGCCCCATGGATTCGAGGAGAGAACTCAGGGGAAGGGCCTGATCGTCAGAGGGTGGGCCCCC
CAGATGTTGATTCTCGACCACGAAGCAATCGGCGGATTCGTGACTCACTGCGGCTGGAACTCCACGCTGG
AGGGAGCCTGCGCCGGACTTCCGATGGTGACTTGGCCGGCGTTCGCAGAGCAATTCTACAACGAGAAGCT
GGTGACGGATGTTCTGAAGATCGGGGTTTCCGTCGGAGCTAAAGAGTGGAAAACAGGGCCGAGGGAGGGG
GTGAAGAGGGAGGCAATCGAGAAGGCGGTGAGGAGCGTAATGGAGGGAGAAAAAGCGGAGGAAATGAGGA
GGAGAGCGGCGGCGCTGAAGGAGGCGGCCATGGAAGCTGTTGAAGAAGGTGGATCTTCATACTCTGATTT
GACTTCTCTCATTGAGGAGTTGACTTCAAGCCAAAAGATAAATGGAACATAA
>GWHPAAAL018716
ATGGAGATGGAGATGGAGAAGAGAGAGTACAGAGCCCATGTCCTGGCAATTCCATTTCCGGCACCAGGCC
ATGTTAACCCACTTCTCCAATTCTGCAAGCGCCTGGAATTCAAGGGCTTGAAGGCGAGTCTCGCCATCAC
CAAATTCCAGTCCAAATCCATGCAATCCAGACTCGGGTCGATCCAGATCCACACGATCTCCGACGGGTAC
GATGAGATTGGACGGCACAACGCGGAGAGCGTCGGCGTATGGATCTCGACCTTCAAGGAAGTAGGTTCGA
AATCCGTGGCGAATCTCATCCAAAAGTTTCGGAGCTTGGGTCATCCGATCGATTGCATCGTCTACGATTC
GTTTCTCCCTTGGGTTCTGGATGTCGCCAAGGAGAACGGAGTTGCCGGAGCTTCGTTCATGACCCACAAG
TGTGCGGTGAATCACATCAACTATCATGTCTACCATAAGAAGATCCCCTACCCCTTTTCTTCTCCGACGT
ACTCGATTCCGGGATTACCGTCGCTCGATCTCGAGTACATGCCCGGATTTATGCACGTCCACCCCGATTA
CTACGACCTGGTTCTCAGCCAATTCTCGACTGTGGATCGCGCTGATTACGTGTTTGTCAATACTTTCTAC
AAATTGGAGGCTGAGGTATTGGATGAAATGTCAAAGACCCTTCCAGTGAAACCAATAGGACCAACAGTCC
CATCTTTCTACTTGGACAACAGGGTTGAAGATGACAAAAACTATGGGCTCAACCTCCAACAACTGGACTC
GTCGGTTTGCCTCAATTGGCTCAGCTCTAAGCCTGCAAGGTCGGTGGTTTACGTGTCGTTTGGCAGCGTG
GCCACCGCCAGTCCAGCAGCCAACGAAATGGAGGAGATAGCGTGGGGGTTGAAGAACAGCAATTGCTACT
TCCTGTGGGTGGTGAATGCTAACGAAGAGCCGAATCTCCCCCAGAATTTCAAGGAGGAGATGTCCGAAAA
AGGGTTGATTGTGGCGTGGAGTCCCCAGCTGGAGGTACTATCGCACCATTCCGTTGGTTGCTTCCTCACT
CACTGCGGGTGGAATTCAACCATCGAAGCTATTTGTCTGGGCGTTCCGATGGTGGCTGTGCCCAAATGGG
CGGATCAAACCACCAACTCGAAGTTCATACAGGATGTTTGGAAAGTTGGGGTCAAAGTGATGCCATCTGA
AAACGGGATTGCTAGAAGAGAAGAGATTGAGCGGTGTATTAATGTT

>GWHPAAAL019177
ATGGACCACCACCGCCGCCGCCACCACTTCCTCCTTATCTCTCTCCCTGCCCAAGGCCACTTCAACCCCA
CTCTCCAGCTCGCCAAAGTCCTTGCACGCGCCGGCGTTACTGTTACCTTCGCCACCACCGTCTACGGCCT
AAACCAGCTAAAGGTTAACTCATTCACCGTCGACGGCATAGCCTACGCCTCCTTCTCCGATGGTCACGAC
GAAGACCGCTCCAAAGCTAGCAGCGACTTTGCCGGCTACCTGGAGGATCAAAGGCGCGTGGGGTCCCAAA
ACCTGATAAAACTCCTCGAAAACCTCGCCAACGACGGCCGTCCCGTTACCTTCATAGTCTACACCGTTCT
CCTTCCATGGGTTGCCCAGGTGGCCCGTGAGATGCACGTGCCGTCGGCATTCTTCGCCATACAGTGTGCC
GCCACTTTGGCCGTCTATCACCGCTTCTTCAACAGCAGCGACGGACTCCTCGGCGGCGAAAACGAAATCA
ACGAGTCGCTTTCCGTTGAATTACCTGGACTGCCGCCGCTGAGCTCCGGCGAGATCCCTTCATTTCTAAT
GCCGGCCGATCAGTATCATTCATCTGTTAGTCCGTCTTTCCGAGAACACATACTCACCCTCGAAGAAGAT
CCAAATCCGACGGTTCTGATCAACACCTTCGATGCCTTAGAGGAAGATTCAATCAGAGCCGTCCGGAATA
TGAAGATCATCGCCGTCGGCCCCTTAGTTCCTTCGGCCTTCTCCGACGGAATCGACGGGTCGGACAAATC
ATTCGGCTGCGATCTGTTTCGAAACTCCGACGACCATCACCTCCGGTGGCTCGATTCGAAATCGGAATCC
TCCGTCATCTACGTTTCCTTCGGGAGCCTAGCCATTCTTGGGAAACCCCAGAAGGAAGAGATCCTGAACG
GACTGATCGACACCGAGAGACCTTTCCTCTGGGTCATCCGCGACACCGCCGGAGAAGAAGAAGAGATGGC
GGTGCCGGAAAATGGACTGGTGGTGCCTTGGTGTACGCAGGTGGAGGTGCTCGCCCATTCATCGATAGGG
TGCTTCGTCACCCACTGCGGGTGGAATTCGACGATAGAGAGCCTGGTCGCCGGAGTTCCGGTGGTGGCGA
TGCCGCAATTCTCCGACCAGTTGACGAACGCGAAGCTGGTGGAGGAGGTGTGGGGGAATGGGGTTAGAGC
GAGAAAGAGTGAAGAGAAGGGGGTTGTAGAGAGAGAGGAAATCAGGAGGTGTGTGGAGGTGGTGGTGGGG
GGAGGAGAGAGGGGAGAGGAGATAAGAGGAAGTGCTCGGAAATGGAGAGGTCTGGCCATGGCTGCCGTCA
GGGAGGGGGGTTCTTCAAGCAAAAATCTTAGAGATTTTTTGGATAGCTTGGGATAA
>GWHPAAAL019178
ATGGACCACCACCGCCGGCGCCGCCTCCACCACTTCCTCCTCATCTCTCTCCCTGCCCAAGGCCACATCA
ACCCCACTCTACAGTTCGCCAAAGTCCTCGCACGCGCCGGCGTCACTGTCACCTTCGCCACCACCGTCTA
CGGCCTTAGCCAGCTTAAGGCTAACTCATCCACCGTCGACGGCATAGCCTACACCTCCTTCTCCGACGGT
TACGACGAAGGCGGTGCCAAAGCAAACATCATCTTCCCCGACTACATGGAGGATCTAAAGCGCCTGGGGT
CCCAAAACCTGATAAAACTCCTCAAAAACCTCGCCGGCGAGGGTCGTCCCGTCACCTTCTTAGTCTACAC
GGTTCTCTGTCCCTGGGTTGCCCATGTGGCTCGGGAGATGCACGTGCCGTCGGCATTCTTCGCCATACAG
TGTGCCGCCACTTTGGCCGTCTATCACCGCTTCTTCAACAGCAGCGACGGACTCCTCGGCGGCGAAAACG
AAATCAACGAGTCGCTTTCCGTCGACTTACCTGGACTGCCGCCGCTGAGCTCCGGCGAGATCCCTTCATT
TCTAATGCCGGCCAATCAGTATCATTCATCTATTAGTCCGTCTATCCGAGAACACATACTCACCCTCGAA
GAAGATCCAAATCCGACGGTTCTGATCAACACCTTCGATGCCTTAGAGGAAGATTCAATCAGAGCCGTCC
GGAATATGAAGATCATCGCCATCGGCCCCTTAGTTCCGTCGGCCTTCTCCGACGGAATCGACTCGTCGGA
CAAATCATTCGGCTGCGATCTGTTTCGAAACTCCGGCGACCATTACCTCCGATGGCTGGATTCGAAATCG
GAATCCTCCGTGATCTATGTTTCCTTCGGGAGCCTAGCCGTTCTTGGGAAACCCCAGAAGCAAGAGATCC
TGAACGGACTGATCGACACCGGGAGACCTTTCCTTTGGGTCATCCGCGACACCGCCGGAGAAGAAGAAGA
AGAAGAAGAGACGGCAGTGCCGGAAAATGGACTGGTGGTGCCATGGTGTACGCAGGTGGAGGTGCTGGCC
CATTCATCGATCGGGTGCTTCGTCACCCACTGCGGGTGGAATTCGACGATGGAGAGCCTGGTCGCCGGAG
TTCCGGTGGTGGCGATGCCGCAATTCTCCGACCAGTTGACGAACGCGAAGCTGGTGGAGGAGGTGTGGGG
GAATGGGGTTAGAGCGAGAAAGAGTGAAGAGAAGGGGGTTGTAGAGAGAGAGGAAATCAGGAGGTGTGTG
GAGGTGGTGGTGGGGGGAGGAGAGAGGGGAGAGGAGATAAGAGGAAGTGCTCGGAAATGGAGAGGTCTGG
CCATGGCTGCCGTCAGGGAGGGGGGTTCTTCAAGCAAAAATCTTAGAGAATTTTTGGATAGCTTGGGATA
A
>GWHPAAAL020336

ATGTTTACCAGTAACAGAGTAGAGCTCGTGTTCATCCCTTCGCCGGGAATGGGCCACCTCGTCTCCGCCG
TCGAGATGGCCAAGCTTCTCGTCGATCGAGACCAACGTCTCTCCATCACCATCCTCATCATGAAAGTACC
CTCCAAAACCAAGGCCAATTACTCGCCGTCCCAATCCCTTTCCACTGCCGCCGCCTCACGTATACGGTTC
GTAGACCTTCCCGAATCAGAATCCACCCCCCAATTCTCCCCGCAATCCTTCATTTCCGGCTTCATCCCCA
GTCAGAAGACCCGCGTTAGGGACGCCGTCGCCAAAATCGCCGCTGCTCCGGGAGCTCACAGACTCGCCGG
TTTCTTCATCGACATGTTCTGTACTTCGATGACCGACGTCGCCGACGAGTTTGGCGTCCCGACTTACGTG
TTCTTCACCTCCGGCGCCGGCTTATTGGGACTGATGTTCCATCTCCAAACCATGAGAGATGACTACAATC
ATGACATCACCGAATTGATGGACTCGGACACTGACTTAATAGTCCCGAGTTGGATCAAACCTGTCCCGCT
TAAATTCCTACCATCTGTATTTGTCAACAAGGAAGAAGGGTCCCACATGTTCCTTGATCTCACCCGTCGG
ATCATGAAGACAAAAGGTATCGTGATCAACACATTCTTCGAGCTGGAATCACAGGCAATCAATTCCCTTA
ACACCCTGCCGGTATATCCAGTCGGACCCATCCTGAACGTCGCCGGCGACAACAACTCCGCTTCTGCTGG
CGCAATCATGAAGTGGCTCGACAGTCAACCCCCTTCGTCGGTGGTTTTCCTATGCTTCGGAAGCATGGGA
TGCTTCGACGTTGACCAAGTGAAGGAGATTGCATGTGCCCTAGAACACAGTGGTCATCGCTTCTTGTGGT
CTCTTCGACGACCACCGTCAAATGAAACGTCAGAGTTACCGGAGGAGTACACAAATCTCGAAGAAGTCCT
TCCAGAAGGATTCCTAGAACGAACGACCAAGATAGGGAAAGTGATCGGGTGGGCGCCACAGACGGCAATA
TTGTCCCACAGGGCAGTGGGAGGGTTTGTATCGCACTGCGGGTGGAACTCGACGCTGGAGAGCGTGTGGT
ACGGCGTCCCACTGGCAACATGGCCACTGTTTGCGGAGCAACAAGTGAACGCATTTATGATGGTGGAAGA
GTTGGAGATGGCAGTGGAGATTAAGATGGATTATCATAAGTCATTTTACTTGAAAAATGGAATAGTTTTG
AAGAGCGAAGAGATAGAGAAGGGAATACGGCGGTTGATGGAGAGCGGTGACATTGAGATCAGGCAAAAGG
TGGAACAAATGAGAGACAAGAGCCGGGTGGCGGTGAAGGAAGATGGATCTTCTTTTCGTTCGATTGAGCG
TTTGATCGAGGATGTAATCAATGGAGCAAAAATGAAATAA
>GWHPAAAL020411
ATGGTCATCCCAGACTTTCTCATTGTTGAAGATAACAACAGCTCTGAAGCTCTGAGCTGGGTTGATTCAT
GTACCGGTGATGGGTATGGTGGCCAAATGCACCATGAAAGCAGCGAATCCAATTGGGAGTGGAGCCAAAG
CCTGCCCTCGAAAACACTGAAAGGGATTCATGGCGAAGAAGACGGTGTAGACAAGCATGAAAGTTCCAAA
CAGTCCAATAACACCATACTGATCAAGAGCTTCAAGGAAATTGAAGGTAAGTACTATGATTATCACTCTC
TTTTGGCCAAGAAAAAGTTTGTCCCTGTCGGCCCAGTGGTTCAGGAACCTGTCTGTGAAGAAGGGTTTTC
TAAGATCAATGAATGGCTAAACAAAAAAGATTACGGTTCAACTGTGCTCGTCTCATTTGGGAGTGAATAT
TTTCTCAAAAAAGAAGATAACATAGAAATAGCACATGGGCTTGAGCTTAGCAACGTGAATTTTATCTGGG
TTTTAAGGTTTCCCAAAGGTGAGAAACTTGTTGCACAAGAAACCTTTCCATTAGGGTTTTTCTATAGGGT
TGGAGATAGAGGGATGGTGGTGGAGGGATGGGCGCCACAGGTAAGGATACTAGGTCACCCCAGTACCGGT
GGATTCGCTAGCCACTGTGGGTGGGGGTCTGTGATTGAGAGCATGAGTTTTGGAATTCCAATTATAGCCA
TGCCAATGCACCTTGATCAAACCATCAATGCTAGGGTTGTGGAGGAAGTGGGTATTGGTGCGGAGGCCAC
CATGTACATCAGTGGAAAACTGAAGAGAGAAAATTGCGGCGGTGATCAGGGAGGTGGTGGTGGAGACAGA
AGGGGAAGGCGTGAGGAAAAAGGCAAGAGAGATGAGGGATGA
>GWHPAAAL021381
ATGACAATACCACATGTTCTTGCAATACCATATCCAGCACAAGGTCATGTAATGCCATTCATGGAGCTCT
CCCAGTGCCTCGTCCAGCATGGTGTCAGAGTCACATTTGTAAACACAGACTTCAATCACAAGAGGGTCAC
AAAATCAATGTCCGAGAAAGAGAGTTTGACGGGCATGATGCATTTGGTTTCAATCCCAGATGGGTTAGAA
CCATGGGAGGACAGAAACGATGTAGGAAAACTATTTGAATCAATCTTTCAAGTGATGCCCTCAAAGCTGG
AGCAGCTGATAGAAAAGATCAATGCATCAGAAGGGAAAAAAATAAGCTGCGTTATTTCTGATCAGTGCCT
GGGGTGGGCTCTCGAAGTTGCAGAGAAGATGGGAATCAAGCGCGTCACCTTTTGGCCAGCATCAACAGCC
ACGATGGCCTTAACAATGAACATCCCAAAACTAATCGATGACGGGGTCATAAATGAAAATGGCACGAGCT
GCTCCTTCAGCTCATGGAATAGATACGAAATAACTCATCTGTACCATTTCTTTTTCATTTTGAAATTTCA

CACTGCAAGAACTCCCATCAGCAAACAGATAATTCAACTGTTACCAACCATGCCGGCCATGAACACCGCA
AACTTCTACTGGGCCTGTTTTGGCGACTTAAGCACGGAAAAAAGTATTTTTCAAATGATGGTGAAAAACA
ATAAATTCCTAAAATTGGCAGACCGGGTGATTTGCAACTCAATCCACGAGCTGGAGCCTGCAGCCTTTAC
TCTATGCCCAGAGGTATTCCCAATAGGGCCACTCCCCTTGATCAATAAGGTCGGCCGTTCAGGAGCCAAC
TTTTGGCCAGAAGACCCAACCTGTCTAAAATGGCTTGACCAACAGCCACCACAATCAGTGATTTACCTTG
CATTTGGAAGCTTCACGATTTTTGATCAAACCCAATTCAGAGAACTCGCTCTTGGGCTTGAACTCACCAA
CAGGCCATTCCTTTGGGTTGTGCGTCCAGACATAACCGACCAGACCAAAGATCCATACCCAGAAGGATTT
ATACAGAGAGTAAGCGATCGGGCCCAAATTGTGGGTTGGGCCCCGCAACAGAGCATCCTAGGCCACCCTT
CTGTGGCTTGTTTCATGAGCCACTGTGGTTGGAATTCCACCATAGAGGGTGTCAGCAATGGAGTCCCTTT
CCTATGCTGGCCCTATTTCGCCGACCAATTTTGGAATCAGCAGTATATATGTGATGTATGGAAGGTGGGA
TTGGGTTTTGCGAGAGATGAAAGTGGGATCATTGTTCAAGATGAAATAAGGAATAAGGTCGAACACTTGT
TGGGCGATGAAAGCTTTAGAGCAAGGGCCACGGTTCTTAAGGAAAAAGCTATGGAGACCGTTAAAGAAGG
TGGTTCCTCTCACAGGAATTTCAGCGATTTAATTGGATGGATGAAAGAGATCAAACATGACTAA
>GWHPAAAL021402
ATGTCTGATGTCGCCGGCCGACCGCCCCATATAGCTCTCTTCCCAGGCGCCGGCATGGGTCATCTCACCC
CATTCCTCCGGCTGGCCGCCATGCTTGCTTCCCATAACTGCAAAGTCACCCTTATTGCCGTCCATCCGAC
AGTTTCCGCCGCTGAATCCTCCCAAATCTCCGCCTTCTTTGCCGCCAACCACCACATCAACCGCCTTGAT
TTTCACATTCTTCCTCACAATCCGCCGGATTCCGAGGTCAATGACCCTTTCTTCGTCCAATTCGAAGCCA
TTAGCCGCTCCGTTCATCTCCTTGGTCCCCTCTTGTCGGCTGCATCTCCGCCAATCTCCGCCGTCTTTTC
TGACTTCCCGGTTGCCGCCAGTATGGCTCAAATAACCGACGACCTCACCATACCCAACTACATAGTATCG
ACCACGTCCGCCAGATTCTTCTCTCTCATTGCCTACTTCCCTCTCTTGAACCTTCAAAACCACAATAAGT
CCCAAATCGAAATCCCGGGTATATCCCCGCTACCATTTTCAACTCTTCCTCCGCCATTATTCAGTCCAAA
CAACCTCTTCACGACTGGGTTGGCAACCAATTCCAAGTACCTGCCAAAAGCAAAGGGCATATTAGTCAAT
TCCTTTCACGGGTTCGAACCAGAAACAATCGAAGCTTTCAACAATGGCAAAATTCAAGAAAATCTCCCCC
CTCTACTCCCAATCGGTCCTTTACTTCAACCCCATGAACACATTCCTCAATGCCATGAAATTTCATGGCT
AGACAAACAGGCTGAAGAGTCTGTAGTGTACGTTAGTTTCGGAAGCAGAACCGCCATGGCAAAGGAACAA
GTAAGAGAACTGGGAGATGGGTTAGAGAGAAGCGGATGTCCATTTTTGTGGGTTTTAAAAACTAAAATCT
TGGACAAAGACGAGAGTGAAGAGGTAGAAGAATTTCTGAGTAATTCATTTCTCGAGAGAACGAGGAGAAA
GGGTGTAGTTTTGAAAGAATGGGTTAACCAGGAGAGGATACTTTCGCACCCTGCGATTGGAGGTTTTGTT
AGTCATTGCGGGTGGAACTCAGTGATGGAAGCGGCTAAAGAAGGGGTGCCCATTTTGGCATGGCCGATAG
GGGAGAAGATTAGAGAGGTGATGGAGGATGAGAAGTTGAGGGCAAAGGCAAGGAAGGTGGGAGAGGAGGC
AAGGAAGGCTGTTGGAATGGTTGGTGGGAGCTCTGAAATGGTGCTGCAGGGGATCCTTCAAAGTATTGCA
GAAACATGCCATTAG
>GWHPAAAL021780
ATGGCCTCCAAACCTCTTGTTCATGTCTTTCTCGTCTCTTTCCCCGGCCAAGGCCACGTCAACCCACTTC
TCCGTCTCAGCAACCTCCTCGCCTCCGGTGGCTTTTTCGTCACTTTCTCCACCACCGTCTCCTTCGGCAA
GCAAATGCAGAGCGTGAATCCTGCCGTCACCGGTGAACCCACTCCCGTCGGCGACGGCTTCATCCGGTTC
GAATTCTTCGACGAAGAGTTGGCCCTGGACGACCCCCGTCGGAGGGACCCAAATCTGTTTTTCTATCACC
TGGAGCTCGCCGGAAAGGAGAATCTCCGCCGTTTGGTGGAGAACCAATCGTCGGAGGACGGTCGAACGCC
GGTGTCGTGCCTCATCAACAACCCGTTTCTCCCGTGGGTTGCAGACGTGGCGGATAGCTTGGGCATTCCC
TGTGCGATCTTGTGGGTGGAATCATGCGCCTCCTTGTCGGCTTATTACCACTACTATCACGGGCTGGTGC
CTTTTCCAGACGGGGAGCAGCCGGAACTCGACGTCGAAATACCCTTTTTGCCGTTGCTGAAACACCATGA
GATCCCTAGCTATTTGCACCCCAATGATCCATGCACATTCTTGAAAAAATTAGTGTTGGGGCAAATCAAG
CGACTACCCAAGATTTTTTGTGTATTGATTGATACTTTTGAAGAGCTAGAGCCTCAAATCATTCGACACG

TGTCCGATCTCATCCCGATAAGGTCTGTCGGCCCTCTGTTGAGATACCGGAAATCGAGTTCCTGGATCAC
CGGAGACCGTTTAAAAACCGACGACTGCATCGAATGGCTTGACTCAAAACCGCCGTCGTCGGTTCTGGTC
ATGTCGTTCGGGAGCGTCGCCCAGGTCAAACAAGAGCAAATCGACGAAATTGCCCACGGACTGTTGAGCT
CATGTGTGTCGTTCTTGTGGGTCATGACGCCGCCGATGGCAGGCTCGCCGGATAAGCCGCGGATCCTGCC
GGATGGGTTCTCTGAGAAAATGGGCGATCGCGGTAAGGTCGTAGAGTGGAGTCCACAGGAGCGAGTCCTG
GCTCACCCGTCGGTGGCCTGTCTCGTGACACACTGCGGCTGGAACACGACGGTGGAAGTGTTGTCCAACG
GGAAGCCGGTGCTGGCTTTTCCTCAGTGGGGAGACCAGGTGAACAACGCCAAATTCTTGGTCGACGTTTT
TGGGGTTGGGATCAGAGTTTACAGGGGGGAAGACAGGGGCAGTATCGTCTCGAGGGATGAGTTTGAGAAA
TGCGTGCGGGAGGCGATGAGTGGCCCTAAAGCGGTAGATATCAAGGAAAACGCGATGAGGTGGAAGAAGG
TGGCGGAGGAAGCGGTGGCGGAAGGCGGCTCCTCCCGCCGGAATATGTTGGATTTCATGGAAGAGATCAA
GAGGAGGAGTGTGGATTGA
>GWHPAAAL021819
ATGGGGGAGAAGCAACCGGAGCTCCAAAAGCAACAGCGACAGGGGCTCCGATTGGTGCTCTTCCCACTTC
CATTCAAAGGCCACATCAACCCCATGCTTCAGCTCGCCGCCATTCTTCACGCCAATGGCTTCCCCATCTC
CATACTCCACACTACCTTCAACTCTCCCACCCCTTCCGACCACCCTCACTTCGACTTCCACCCAATCTCC
GTCGACGGTCTGTCGGAAACAGATTCTCCGACGCCGGATCTGGTTGATCTCCTCGAACTACTCAACCAAC
GATGCGCGGCGCCGTTCCGCGATTGCCTGGAGAAGTTGACATCGGAGGAGCCCGTCGCCTGTTTGATCTC
GGACGCCATTCTGCATTGCACCGCCACCGTCGCTGACAGTCTTCGTATTCCAAGGATCGTACTGAGGACC
GGCGGGGTCGCCTCCTTTCTTGCGTTTGCCTCCTTTCCACTTCTTCTGGATAAGGGTTATCTTCCCATTC
AAGAATCGAGGCTTGAAGAGGCAGTTCCAGAGCTTCCTCCACTGAGAATCAAAGATCTTCCGGTGATCAA
TACCGGCAAACCGGAGGCCCTATATCATCTTCTAGCAGAGATGATCAAGACGGCCAAGAACTCCTCAGGC
CTCATCTGGAACACCTTCGACGAACTTGAACAGCCGGAATTATCCAAACTCCGGCGTGAATTCCCCATCC
CAATCTTCCCAATCGGCCCTTTTCAGAAACACCTCCTCCTAACTTCCACACACGAAGACCGGAGTTCTTC
CATCTCTTGGCTTCAAAATCAACCCCCAAGCTCCGTCATCTACGTGAGCTTCGGCACCATTGCGTCAATG
GAGGAATCCCAATTCACGGAGATAGCCCATGGACTCGCCGACAGTCTCCATCCCTTCCTGTGGGTGGTCC
GGCCAGGTCTAATCCGTGGCTCGGAATGGCTCAAGAATTTGCCGGATGGGTTCTTGGAGAGGCTGGAAGG
AAGAGGGAAGATCGTGAAGTGGGCTCCACAGGCAGAGGTGTTGGCTCATCCGGCGGTCGGCGCATTCTGG
ACTCACAGCGGGTGGAATTCGACGGTGGAGAGTATCAGCGAAGGAGTTCCGATGATATGTATGCCGTGTT
TCACGGATCAGTTGGTGAATGCCAGGTATGTGAGCCATGTTTGGGGAGTTGGGTTGACGTTGGAAGGTGG
GATTGAGAGGGGGAAGATTGAGAGGGTGATGAGGAGAGTGATGGGTGAAGAAGGGAAAGAGATGAGGAAG
AGGATTTTGTGTATGAAGGAGAGAGTGAATGTTTGCTTGAGGAAAGGTGGATCCTCCCATCAATCTCTAC
ACAACTTGGTAAGTTACATCTCGTCACTCTAG
>GWHPAAAL022080
ATGACTTTTCTCTGTTCATCCTTCCTTGTTGCCTACGTTTGGCCATCACCTGAAACACCCGGTAGCCAAA
AACCTCATGTTCTGGTAGTCCCATTTCTAGCACAAGGTCACTTTAGTCCAGCGCTGCAACTCTCCAAGCG
CTTAGCCTCAAAAGGTCTTAGGGTCACTTTTGTCACCACCATTAGCTGCCCCAAGTCCAATGGTTCCATT
AGGATGGAGCAAATTAGCGATGGCTACGAACAACGTGAAAAACCAGAGAGCATTGAAGCCTTTGCCGAAG
GTATCAAAACCGCAATCTCGCAAGGCCTACATGATCTAATGGAGAAACAAAAACAAGTCGGTTACCCTGT
GAAACTAGTTGTTTATGATTCTGGTATGCCATGGGCACTAGAGATGGTACATAAGTCGGGTGTTTATGGA
GTTCTATTCTTCACTCAACCCTGCGTTGTATGTGCCCTCTACTACCATATGCAACAAGGGACTCTGGAGG
TTCCATTTGAAGGAACAAAAGTCACCCTACCTTCATTGCCGCCATTGGAGAAAAATGATCTGTCATCTTT
TATCTCTAACTTGTTCCCACACGAATTCCTCGTGAAAGGGTTATTGGACCAATTCTCCAATATCCACAGA
GCAAATTGGATCTTATTCAACACATTCGACAAACTGGAAAAAGAGGTAGTGAAGTGGATGGCAAGCCAGT
GGCCTATCAGAACCGTCGGACCAACTATTCCACCCATGTATTTAGACAAACAACTGAAGAGAGACAAAGA

ATACGGCCTTAGCCTCTTCACTCCAAACACCGAGGCTAGCATGAAGTGGCTGGACACGAAAGAACAAGGT
TTAGTGGTTTATGTCTCATTTGGGAGCATGGCCATCCTTGATGAAACTCAAATGGAGGAATTATCATGGG
GCCTCGTGCAGAGCAACAACTATTTCTTGTGGGTGATCAGAGCTTCTAAAGAGAGCAAGCTTTCCAGCAA
GTTCATGGCTAAGACCTTAGAGAAAGGGCTGATGGTTAATTGGTGCCCTCAACTTGAGGTTTTGGCTCAC
CAAGCGGTGGGATGTTTTGTGGCTCACTGTGGTTGGAACTCGACGCCTGAAGCTTTGTGCCTTGGGGTGC
CAATGGTTGCTATGCCACAGTGGGTTGATCAAACAACTAATGCGAAGTTCGTGGCCGATGTGTGGGGGGG
CAGGGGTTCGAGAGGAGGCCAATGA
>GWHPAAAL022859
ATGGAGATGGAGATGGAGAAGAGAGAGTACAGAGCCCATGTCCTGGCAATTCCATTTCCGGCACCAGGCC
ATGTTAACCCACTTCTCCAATTCTGCAAGCGCCTGGAATTCAAGGGCTTGAAGGCGAGTCTCGCCATCAC
CAAATTCCAGTCCAAATCCATGCAATCCAGACTCGGGTCGATCCAGATCCACACGATCTCCGACGGGTAC
GATGAGATTGGACGGCACAACGCGGAGAGCGTCGGCGTATGGATCTCGACCTTCAAGGAAGTAGGTTCGA
AATCCGTGGCGAATCTCATCCAAAAGTTTCGGAGCTTGGGTCATCCGATCGATTGCATCGTCTACGATTC
GTTTCTCCCTTGGGTTCTGGATGTCGCCAAGGAGAACGGAGTTGCCGGAGCTTCGTTCATGACCCACAAG
TGTGCGGTGAATCACATCAACTATCATGTCTACCATAAGAAGATCCCCTACCCCTTTTCTTCTCCGACGT
ACTCGATTCCGGGATTACCGTCGCTCGATCTCGAGTACATGCCCGGATTTATGCACGTCCACCCCGATTA
CTACGACCTGGTTCTCAGCCAATTCTCGACTGTGGATCGCGCTGATTACGTGTTTGTCAATACTTTCTAC
AAATTGGAGGCTGAGGTATTGGATGAAATGTCAAAGACCCTTCCAGTGAAACCAATAGGACCAACAGTCC
CATCTTTCTACTTGGACAACAGGGTTGAAGATGACAAAAACTATGGGCTCAACCTCCAACAACTGGACTC
GTCGGTTTGCCTCAATTGGCTCAGCTCTAAGCCTGCAAGGTCGGTGGTTTACGTGTCGTTTGGCAGCGTG
GCCACCGCCAGTCCAGCAGCCAACGAAATGGAGGAGATAGCGTGGGGGTTGAAGAACAGCAATTGCTACT
TCCTGTGGGTGGTGAATGCTAACGAAGAGCCGAATCTCCCCCAGAATTTCAAGGAGGAGATGTCCGAAAA
AGGGTTGATTGTGGCGTGGAGTCCCCAGCTGGAGGTACTATCGCACCATTCCGTTGGTTGCTTCCTCACT
CACTGCGGGTGGAATTCAACCATCGAAGCTATTTGTCTGGGCGTTCCGATGGTGGCTGTGCCCAAATGGG
CGGATCAAACCACCAACTCGAAGTTCATACAGGATGTTTGGAAAGTTGGGGTCAAAGTGATGCCATCTGA
AAACGGGATTGCTAGAAGAGAAGAGATTGAGCGGTGTATTAATGTT
>GWHPAAAL023009
ATGATTCCGACGCTGGACATGGCCAAGCTCTTCTCCTCCAGGGGTGTCAAAACCACCATCATCACCACCC
CTCTCAACGCCCCTGTTTTCTCCAAATCAATCCAGAGGTCCAACCAATTGGGTCTGGAAATCAGCTTAAA
AATCTTTGATTTTCCGGCGGTTGCCGCGGGCTTGCCGGAAGGTTGCGAGAGCGCTGACCAAATCACCTCC
GAGGACATGATTCCAAAATTCTTCGTCGCCACGGCGTTGCTCCGGGAGCCGCTGGAGCAGCTGCTAAGTG
ACCACCGCCCGGATTGTTTAGTCGCCGACATGTTCTTCCCTTGGACGACGGAGTCGGCGGCGAAATTCGG
CATTCCGAGATTGCTTTTCCACGGGATCAGTTTCTTCGCGCTCTGCGCCGGGGAGTCTTTGAGGACGCAC
AAGCCTTTCCGGCAAGTCACGTCGGATTCCGAACCCTTTGTCCTGCCAGAGCTCCCGCACGAGATAAAGC
TGACGAGAACGGAAGTGGCGCCGTTTGACCTGTACGATACCCAAACGGCCATATCCGGAGTCTTGAAACA
AGTGAGGGAGTCGGAGAAAACTTGTTATGGCATTATCGCAAACACCTTCTATGCACTCGAACCCGATTAC
GCCGATCACTACAGAAACGCCATGGGGAGAACGGCGTGGCACGTCGGACCTCTCTCTCTCTGCAACAGAG
GAATCGAAGACAAAGCAGAGAGAGGAAAAAAAGCTTCCATCGACGGACACGAATGTCTTCAATGGCTTGA
TTCAAAGAAACCCAAATCCGTCCTCTACGTCTGTTTCGGAAGCATGGCCAAATTCGCCGATTCCCAACTG
TACGAAATCGCCGCCGGTCTTGAATCCTCCGGCCAACAGTTCATCTGGGTGGTGAGAAAATCAAAAGACG
AAGAAGAACAGAAGGAAGAGACATGGCTGCCCCATGGATTCGAGGAGAGAACTCAGGGGAAGGGCCTGAT
CGTCAGAGGGTGGGCCCCCCAGATGTTGATTCTCGACCACGAAGCAATCGGCGGATTCGTGACTCACTGC
GGCTGGAACTCCACGCTGGAGGGAGTCTGCGCCGGACTACCGATGGTGACTTGGCCGGCGTTCGCAGAAC
AATTCTACAACGAGAAGCTGGTGACGGATGTTCTGAAGATCGGGGTTTCCGTCGGAGCTAAAGAGTGGAA

CAGAGGGCCGAGGGAGGGGGTGAAGAGGGAGGCGATCGAGAAGGCGGTGAGGAGCGTAATGGAGGGAGAA
GAAGTGGAGGAAATGAGGAGGAGAGCGGCGGCGCTGAAGGAGGCGGCCATGGAAGCGGTTGAAGAAGGTG
GTTCTTCCTACTCTGCTTTGACTTCTCTCATTGAAGAGTTGAGCTCCCGCCAAAAGATTAATGGAAAATA
A
>GWHPAAAL023788
ATGGAGAGAAAAACCAGCATCTTAATGTTCCCATGGATTGCCTATGGCCATATATCTCCATTTCTAGAGC
TTGCCAAAAAGCTCTCACAGAGAGACTTCTTTATATACCTGTGTTCAACTCCAGCCAATCTTGCCTCCGT
CAAGCCCAAAATTGATGAGAAATATTCTCTCTTAATAAAGCTTGTGGAGTTCCACCTCCCCACTCTCCCC
GACCTTCCACCGTGCTTCCACACCTCCAACGGCCTCCCGCCCCACCTCATGCACACCCTCATGACCGCCG
TTGACATGTCAAACCCAAGTTTCTCCGACATTCTCGAAACCCTAAAACCAGATTTACTCATATACGACTT
TGTCCCGCATTGGGCTCCGGCCACCGCGGCAGCCCACAACATCCCGGCGGTGGAGTTCATCACCAGCAGC
GCCACTGTCTATTTTCGACATTACGAGAAAGCACACCAGGATGAAATGATTGAATCCGACAGATATCAGT
ACAATAAGAATCTCTCCGTAAAGTCCCCCCAACAATCCAACAATATCATACTGATCAAGAGCTTCAAGGA
AATCGAAGGTAAGTACTATGATTATCTCTCTCTTTTGGCCAAGAAAAAGATTGTCCCTGTCGGCCCAGTG
GTTCAGAAACCTTTCTGTGAAGAAGGGTTTTCTGAGATCATTGAATGGCTAAACCAAAAAGATTACGGTT
CAACCGTGTTCGTTTCATTTGGGAGTGAATGTTATCTCAAAAAAGAAGATATCATAGAAATAGCACATGG
GCTTGAGCTTAGCAACGTGAATTTCATCTGGGTTTTAAGGTTTCCTAAAGGTGAGAAACTAGGTGCACAA
GAAACACTTCCATTAGGGTTTCTCGATAGGGTTAGAGAGAGAGGGATGGTGGTGGAGGGATGGGCGCCAC
AGGCAAGGATACTAGCTCACCCCAGTACAGGTGGATTCGCCAGCCACTGTGGGTGGGGGTCTGTGATTGA
GAGCATGAGTTTTGGGGTTCCAATTATAGCCATGCCGATGCAGTATGATCAACCAATCAATGCTACGGTT
GTGGAGGAAGTGGGTCTTGGTGTGGAGGCCACCAGGGACATAACCGGAAAACTGCAGAGAGAGAAAATTG
CGGCGGTGCTCAGGAAGGTGATGGTGGAGAGAGAAGGGGAAGGCGTGAGGAAGAAGGCAAGAGAGATGAG
GGATGATATCAGAATCAAAGGGGACGAGGAGATTGACAACGTGGTGCTGGAATTACAGCAGCTATGCAAG
ATAAAATAA
>GWHPAAAL023789
ATGGAGAGAAAAACCAGCATCTTAATGTTCCCATGGATTGCCTATGGCCATATATCTCCATTTCTAGAGC
TTGCCAAAAAGCTCTCACAGAGAGACTTCTTTATATACCTGTGTTCAACTCCAGCCAATCTTGCCTCCGT
CAAGCCCAAAATTGATGAGAAATATTCTCTCTTAATAAAGCTTGTGGAGTTCCACCTCCCCACTCTCCCC
GACCTTCCACCGTGCTTCCACACCTCCAACGGCCTCCCGCCCCACCTCATGCACACCCTCATGACCGCCG
TTGACATGTCAAACCCAAGTTTCTCCGACATTCTCGAAACCCTAAAACCAGATTTACTCATATACGACTT
TGTCCCGCATTGGGCTCCGGCCACCGCGGCAGCCCACAACATCCCGGCGGTGGAGTTCATCACCAGCAGC
GCCACTGACTATTTTCGACATTACGAGAACGCACACCAGGATGAAATGATTGAATCCGACAGATATCAGT
ACAATAAGAATGTCTCCGTAAAGTCCCCCCAACAATCCAACAATATCATACTGATCAAGAGCTTCAAGGA
AATCGAAGGTAAGTACTATGATTATCTCTCTCTTTTGGCCAAGAAAAAGATTGTCCCTGTCGGCCCAGTG
GTTCAGAAACCTTTCTGTGAAGAAGGGTTTTCTGAGATCATTGAATGGCTAAACCAAAAAGATTACGGTT
CAACCGTGTTCGTTTCATTTGGGAGTGAATGTTATCTCAAAAAAGAAGATATCATAGAAATAGCACATGG
GCTTGAGCTTAGCAACGTGAATTTCATCTGGGTTTTAAGGTTTCCTAAAGGTGAGAAACTAGGTGCACAA
GAAACACTTCCATTAGGGTTTCTCGATAGGGTTAGAGAGAGAGGGATGGTGGTGGAGGGATGGGCGCCAC
AGGCAAGGATACTAGCTCACCCCAGTACAGGTGGATTCGCCAGCCACTGTGGGTGGGGGTCTGTGATTGA
GAGCATGAGTTTTGGGGTTCCAATTATAGCCATGCCGATGCAGTATGATCAACCAATCAATGCTACGGTT
GTGGAGGAAGTGGGTCTTGGTGTGGAGGCCACCAGGGACATAACCGGAAAACTGCAGAGAGAGAAAATTG
CGGCGGTGCTCAGGAAGGTGATGGTGGAGAGAGAAGGGGAAGGCGTGAGGAAGAAGGCAAGAGAGATGAG
GGATGATATCAGAATCAAAGGGGACGAGGAGATTGACAACGTGGTGCTGGAATTACAGCAGCTATGCAAG
ATAAAATAA

>GWHPAAAL023791
ATGGAGAGCAAAACCAGCATCTTGATGTTCCCATGGATTGCCCATGGCCATATATCTCCATTTCTAGAGC
TTGCCAAAAAGCTCTCAAAGAGAAACTTTTTGATTTACCTGTGTTCAACTCCGGCGAATCTTGTCTCCGT
CAAGCCCAAAATTGATGAGAAGTTTTCTCTCTCTATAAAGCTTGTGGAGCTCCACCTCCCCGCTCTCCCC
GACCTGCCGCCGTGCTTTCACACCACGAATGGCCTCCCGCCCCACCTCATGTTCACCCTCAAGGACGCCT
TTGACATGTCGAACCAAAATTTCTCCGACATTCTCGAAACCCTGAAACCAGATTTACTCATTTACGATTT
TCTCCAGCCGTGGGCTCCGGCCACTGCGGCCGCACACAACATCCCGGCGGTGGAGTTCATCACCAGCAGC
GCCACCATGACGTCCCTGATGCTCCACGTGTTCAACGACCCAGACAAAGACTTCCCCTTCTCGGACACTG
TCTATTTTCGAGATAACGAGAGAGCCCACCTGGATCGAATGATTGAATCCGCCAGAAATCAGCACAAGAA
GAAGAATCCCGCCATAGAGTGTCTCAAACAATCCAACAATATCGTACTGATCAAGAGTTTCAAGGAAATC
GAAGGTAAGTACTGTGATTATCTCTCTCTTTTGGCCAAGAAAAAGCTTGTCCCTGTCGGCCCTGTGGTTC
AGGAACCTGTCTGTGAAGAACAGTTTTCTGATATCATTGAATGGCTAAACAAAAAAGATCACGGTTCAAC
TGTGTTCGTCTCATTTGGGAGTGAATATTTTCTCAAAAAAGAAGATATCACTGAGATAGCACATGGGCTT
GAGCTTAGCAACGTGAATTTTATCTGGGTTTTAAGGTTTCCCAAAGGTGAGAAACTTGCTGCACAAGAAA
CCCTTCCATTAGGGTTTCTCGATAGGGTTGGAGAGAGAGGGATGGTGGTGGAGGGCTGGGCGCCACAGGC
AAGGATACTAGGTCACCCCAGTACCGGTGGATTCGCCAGCCACTGTGGGTGGGGGTCTGTGATTGAGAGC
ATGAGTTTTGGAATTCCGATTATAGCCATGCCGATGCACCTTGATCAACCCATCAATGCTAGGGTTGTGG
AGGAAGTGGGTATTGGCGTGGAGGCCACCAGGGACATCACCGGAAAACTAAAGAGAGAGAAAATTGCGGC
GGTGATCAGGGAGGTGGTGGTGGAGACAGAAGGGGAAGGCGTGAGGAAGAAGGCAAGAAAGATGAGGGAT
GATATCAGAATCAAAGGGGAGGAGGAGATTGACAATGTGGTGCTGGAATTACTTCAGCTATGCAAGAAAA
AATAA
>GWHPAAAL023813
ATGGATACTACTAGAGAGAGAAGCAGTGAGAGAATGAGAATACTGATGTTCCCATGGATAGCCCATGGTC
ACATATCTCCATACCTAGAACTAGCCAAGAGACTCAACCAGAGAAACATCAAAACCTACCTGTGTTCAAC
CCCCATTAATCTTAACTCAGTCAAGAACAAGATTGACAACAACTCTGATTCACTACTACAACTGGTTGAG
CTCCACCTTCCGTCCACACCGGAGCTTCCTCCGCAGTACCACACCGCCAATGGCCTCCCACGCCACCTCA
TGATCCCCCTCAAAGCCACCATCGAAGCCAGCGGCCCACCCAAGTTCTCCGCCATGCTCGAAACCCTAAA
ACCGGACTTGGTGTTGTATGATTTTAATTTGCCCTGGGGCGCCACTGTAGCTGCGTCGCACAACATCCCT
GCTGTTCAGTTCATAATCGTTGGAGCGGTGATGATTTCTTTCGCCCTCCACACGCTGGAGAAACCGGCGA
CCGAAAAGTACCCATTTCCGGCAATAACACTGTCGGAGTCCCAATCTCACGAAATGCGCAAATTATTGGA
CGAATCGGTGCTTGGGATTTCCGACAAAGACCGGTTCCGGGAAGGCGTCGATCGTTCTCAAGACATTATT
CTGATCAAAACCTCAACAGAAATTGAGAGTAACTACCTGAATTATCTCTCGGAGCTCGCCGGAAATAAGA
AGATTGTTCCGGTGGGCCAACTTGTACAGCAAGCCGATGATCGAAGACGTGATTTTAACGATGAGGACCA
GATCATCAAATGGTTGGACAAAAAGGAGAAAGCTAATTCAGTCGTGTTTGTCTCATTCGGATCGGAATGT
TTTTTATCAAAGGAGGAGATTCAAGAAATGGGTCGCGGTTTAGAGCTAAGCAGAGTTGATTTCATCTGGG
TCGTCAGATACCCAGTTGGGGACAAAACTAGGGTTCTTGAAGATGAACTGCCCGAAGGGTTTAGAGAGAG
AGTACTCGGAGGAGAGAGAGGCATGGTGGTGGAGGGATGGGCCCCACAGAGTAAAATACTAGGTCACCCA
AACCTAGGTGGGTTTTTGAGCCATTGTGGGTGGAGCTCGGTCATGGAGAGCATGAAAATGGGCGTTCCGA
TCATAGGGATGCCAATGGGGGTTGATCAGCCATTGAATTGTAAGGTGGTGGAGGACGCCGGCGTCGGGGT
GGAGGTTAAGAGAGACAGCGATGGAAAAATAAAAGGTGAAGTGATTGGTGAGGCGATAAGGAGGGTGGTG
GCGGAGATGGGCGGCGAGAGTGTGAGGAAGAAAGCAAAGGAGTTGGGTGAGAAGTTGAGGGAGACTGGAG
AGAGAGAGGTGGAGGCGGCGGTGCAGGAGATCTTGAAATTCAGTTCCAATAAACAATAA
>GWHPAAAL023819
ATGGCGGCGAAAAACGACGATGAATCAGGGGACAATCAAACGGTTGCGGAGTACGGCGAAAAACGACGAT

GGGGAGGATGTTCGTTGCCGGTGAGGCGGCGGCGATGGAGACGGGGAATTGGTGGGGATGAAAGAGAGAG
TGNGAACTCCGGCGAGTTTTCTGAATGGCTGGACGGGAAGGAGGAGCTATCGACAGTGTTCGTCTCCTTC
GGGAGCGAGTACTTCTTGTCTAAGGAAGAGATAGTGGAGATAGCACATGGGTTGGAGCTAAGCGAAGTTA
ATTTCATCTGGGTAATCAGGTTCCCAATGGGGGAGGAGAAGAAAACTAGGGTTGGGGAGGCACTGCCATT
AGGGTTTAAGGAGAGAGTAAGGGAGAGAGGGATGGTGGTGGAGGGATGGGCACCACAGGCGAAGATATTA
GGGCACAAAAGTGTAGCTGGGTTTGTGTCTCACTATGGGTGGAACTCGGTTTTGGAAGGGATGAAGTTTG
GGGTACCATTGGTGGCCATGCCCATGCACCTTGATCAGCCGGTGAACGCCAGGCTTGCGGTGGAGGTAGG
TGTTGCGGTGGAGGCTTTGAGGGATGAGGACGGCGGCGTTCAAAGGAAGGAGGTGGCGAGAGTGATAAGG
GAGGTGGTGGTTGGGGAAAATTGTGGAGGAGTTAGAGAGAGAGCAAGGGAGTTGAGTGAGAACATGAGAT
TGAGAGGTGATGTGGAGGTACATGGGCTTGTGGAAGTCCTAGCACAACTTCATCAGAAGAATAAAGCACA
ATCATGTTAG
>GWHPAAAL024115
ATGGCCGAGGATAGGAATGAGGGAAACATTGTTATGTTTCCATTCATGGCCCAAGGCCATCTCATCCCTT
TCTTAGCACTTGCGAGACAGATAGAGCAAAGGCACGGCGGCTACACAATCACCATCGTCAACACACCTCT
AAACATTTGTAAGCTCAAATGCTGGCTCCCGCCGGAAACAACCATACGCCTGGTAGAGATTCCCTTCTCC
GCCGCCGACCACGGCCTCCCTCCAGATTCGGAAAACACCGACATGCTTTCCCGTGAGCAAGCCCTCTACC
TCTTTGAGGCTTCGGAGAGTCTCGAAAAACCGTTCGAGAATCTCATATCAGAGATTGCCACCGGCGATGG
TCGCCAGCCAATATGCATCGTGTCGGATATGTTCTTGGGGTGGACGGTCAATGTCGCGAAAAAGCTCGGA
ATCTTTCATTCCGTGTTTATCGCCGGTGACGGTTACGGCATGGGATTGTACTTTTCTTTGTCTCTGAATG
AAAATTTGTGGACGGTTGAGGCGGCGGATGAGTTTCTTCCAGTTGACTTTCCTGGAGCAGCAAGGATTCA
CTGGTCACGGATTCCGATAGACCTCAAATATGAAGAGGCGGCCGAGTCTTGGTTTCGATTTCGAAAACGC
CAATTTTCTTACTGTTTGTGCTCCGATTCGATCTTGTTGAACAGTATCAAGGAGTTGGGGCAAAAGGGCG
TGGAATATTTCCGCCGGAAAACGGGAGGAAAGCCTGTATGGACAATAGGCCCAGTTTCCAATCTCACGGT
GAAGAACAAAATATTCACCAAAATCAGCACAAACCCTATCTCCGACTGGCTCGATCTTCACCCTCCGGGT
TCGGTTCTATACATATCGTTTGGATCACAGTGCAGCCTCTTGGCCTCACAAATGAAGGAATTGGCTATGG
GTTTGGAGGAGAGCAGGTGTCCATTCATATGGGTGGTTCGGCCGCCGGTCGAATTTGACCTGACCGGAGA
TTTCAGGAGTGAATGGCTACCGGTGGGGTTCGAGGAGAGAATGAGGCGGTTGAACCAGGGTTTTTTGATT
CACGGATGGGGCCCACAGCTAGAGATTCTCTCTCACCGGTCGACCGGCGGGTTTCTCAGCCACTGCGGGT
GGAACTCCGTGGTGGAGGGGTTGTGCGGTGGAGTGGTGATATTGGGCTGGCCAATGGCCGGAGAGCAGTT
TTTCAACTCGGAGATGCTGGCGGAAGAGGTGGGCGTTTGCGTTGAGGTGAGAGAAAAGAATAACGGGTGC
GGGATTGTCGGCCATCAAAGGATTGCAGAGGTGATCGAAATGGCCATGGGGAAGTCAGAGAAAGGGGAGG
AAATGAGGAGAAAAGCAAGAGAGATGAGCGAGAAAATGGAAGCTGCAGTAAAAGTGGAAGAAGGGTTTAG
AGGATCATCTGTCAAAGTCATGGACGAATTTATCAGCACAGCAATATCAAAGAAGAAGAGATAA
>GWHPAAAL024577
ATGGCGGCCAACCATCAGAATGGCGGTAGCCTAACTTTCACCGGTCCGGTTTCCATCGTTGCCGTTCCTT
TTCCAGCACACGGCCATCTCAACCAGCTCCTCCACCTCTCCTGCTTCTTCTCCTCCGCCGGTCTTCCCGT
TCATTTCGCCAGCACCGCCACCCACAACCGCCAAGCCAGGCTCCGCGCCACCAGCCTCAACTTCGAGAAG
ATCCATTTCCACGACCTCCCTCACCCGCATTTCCAATCTCCGCCACCGAATCCGACCGCCGCCATCAAAT
TCCCGGCCCATTTACAGCCGTCGTTCGAGGCCGCCCTCCATCTCCGGCAACCCCTCGCCGCCCTCCTGCG
AGAAATCTCCACCGTTTCGAGAAGGGTCATCGTCATTCATGACCCACTCATGAGTTCGGTGGCGCAAGAA
GCTGCGTCGATTCTCAACCCCGAAACCTACGTCTTCAGCTGCCTCTCCGTCTTGTTTCAGCTCGCGCACC
TCTGTGAAAATAAATTTCAGGCGGAACTTCCGGCGAACTTACCCACCATGGATGGGTGCTTAACGCCGGA
ATTCATGGAGTTTTCAGCTTCTCAATTTCAGCTACTGGGGACTGAAGAAGGAAAATTCAGAGCTGGGTAT
CTCTATAACTCCTGCAAATTAATCGAAGGTAGGTACATTGATTTACTGTCGAGCTATGATCAGTATCAAG

ACAAGAAAATATGGGCAATCGGACCGATCCTGCCGGTAAAATCAGTGACCAAAACCTCAAATTGGAGCCA
TAAATCCATTGAATGGCTCGATAAACAAGAACCCAGATCGGTGATTTATGTTTCTTTCGGGACGATGACG
TCAATTTCCGACGAACAGATCAGAGAGTTGGCGGTGGGGTTAGAACAGAGTAAACAGAGGTTCATCTGGG
TTTTGAGAGAAGCAGACAGGGCAGACATTTTTTCCGGCGAAGAACAGAGGAAGGTTGAGGTGCCGGAGGG
GTTTGAAGAGAGAGTCAAAGGGATAGGGATGGTGGTGAGAGACTGGGCGCCGCAGCCGGAGATTTTAGGC
CACCGGTCGACCGGCGGATTCATGACTCACTGCGGTTGGAATTCGAGTTTGGAGAGTGTGACCATGGGAG
TGCCGGTGGCCGCCTGGCCGATGCACTCCGACCAGCCGAGAAACGCCGTTTTGTTGACGGAAGTACTGAA
AGTCGGCGTTCCGGTGAGGGAGTGGACGGGAAAAGGAGAGATTGTGGGTTCGTCGGAGATCGAGGAAGCT
GTGAGAAGATTAATGGCGTCTGAAGAAGGGGAGGAAATAAGGAAGAGGGCGGAGGAACTCGGCGGAGAAA
TCCGCCGATCTACGGCGGAAGGCGGCGTTTGCAGGGTGGAGTTAGATTCATTTGTAGCCCATATCACCAG
AAATTAG
>GWHPAAAL024578
ATGGAAGCCTTTGGATTTTCCGGCAGAGACCACTCAAGCCGCCACGACGTGGTGGTGGTGATGGTGCCAT
TTCCGGCGCAGGGCCATCTGAACCAGCTCCTCAACCTCTCCCGCCTCCTCACCTCCCACGGCATTCCCGT
TTACTTCGCCGGCACCGCCATTCACAACCGCCAGGCACGGCTGCGTGCCCACGGCTGGGATCCACTCACC
ACCCCCAACATGCACTTCCACGACTTCCCCACCCCTTCGTTCCAATCTCCGCCGCCGAACCCTAATGACG
CCTCCACCAGCTTCCCCACCCACCTCCTCCCTTCCTTCAACGCCTCCCTCGACCTCCGCCAGCCTTTCGC
CGATCTTCTCCGCGATTTCTCGCCGGAGGCAAGGAGAGTCGTCGTCATTCACGATTCTCTGATGGGTTCC
GTTGTTCAGGACGTCGGTTCGACTCCGAATGCCGAGTCCTACACTTTCCACAGCGTCTCCGCCTTCACCA
TTTTCTGGTACCTCTGGGAAGCGAAGGGCAGGCCCTTTCCGGTGGAAGGAGAGATTCTCAAAGACATTCC
GCCGACCGACGGCTGCTTCGCGTCGGAGTTCCCGGAGTTCATACAGAACCAACATGCATACATGAAGTTC
AACTCCGGCGATCTTTACAACACCAGTAAAGTCATCGAAGAAACCTACATCGATTACTTGTGTAAAGAAC
CGATTAAAGGAAACAAGAAGCAATGGCCGATCGGCCCGTTTAATCCGGTAGAGCCCGTTGAGACGAAGAA
ACCCAACGGCCGCCATTTTTGCCTGAACTGGCTCGACAAACAGGCAATAAACTCTGTGATTTTCGTTTCG
TTCGGAACGTCGACTTCATTGAAAGACGATCAGATCGAAGAACTGGCAAATGGGTTGGATCAGAGCGAGC
ATAGATTCGTATGGGTGGTGAGAGACGCCGACAGAGGTGACGTCTTCGACGGAATGCCGAGGAGAGTCGA
ACTTCCGGTAGGGTTCGAAGAGAGGGTGAGAGAGAAGGGAATTGTAGTGAGAGATTGGGCGCCGCAGCCC
GAGATTCTCCGGCACCCGTCGACGGGAGGTTTCATGAGTCACTGCGGTTGGAATTCTTGTATGGAGAGCA
TCACCATGGGGGTGCCGATCGCGGCGTGGCCGATGCACTCCGACCAGCCGAGAAATGCCGTGCTGATCGC
CAAGTTGCTGAAGATTGGGATCATCGTGAAGGACTGGGGGCGGCGGGGGGAGCGAGTGGTGGCGGAGACG
GTTGAGAAGGCCGTCAAGTCGGAAGAGGGTGACAAGATACGGCACGGCGGCGGTTTGAGCGACGCGGTGA
GGGAATCGGTGGCGGAAGGTGGGTCCACGCGGTTGGAGTTGGATTCGTTCGTTGCTCACATCACTAGGAG
TTAA
>GWHPAAAL025080
ATGGAGGATGCCATAGTTCTCTATCCTTCTCCAGGAATTGGGCACCTCATCTCCATGGTGGAGCTGGGGA
AGCTCATACTCAACCACCACCGGTCCTTCTCCATCAACATCCTCATCACCACCCCACCCTACAATACCGG
CCGCACTGCTCCCTACATCGACCGAGTCTCCGCCGCCAATCCCTCCATCCACTTTCACCACCTCCCCTCC
GTCCCCCTCTCAGTCTCCACCACCTCCCTCAACCACGAAACCCTGGCCTTCCAACTCCTCCTCCTCAACA
ACCCCCATGTCCACCAAGCCCTTACCTCCATATCCCAAACCTCCACTCCCTGCGCCTTCATCATTGACTT
CTTCTGCGCCTCTGCTCTCTCTGTTGCCAAAGATCTCAATATTCCGACATACTTTTTCTACACTTCCGGT
GCCGGAGCCCTCGCTTCCTTCCTTTATCTCCCCACCATTCACCGGAGCACTGCCGGAAACCTGAAAGACC
TTAAAACCTCCCTCCACATCCCCGGACTCCCGCCATTTCGGGCGTCTGACATGATGAAACCGGTGCTCGA
CCGATCTGACAAAGCGTACGAATTCTTCTTGGACGTATCCTTAAACTTCCCGAAAGCTTCCGGAATCATC
ATCAACACTTTCGAATATCTTGAGCCTCGATCCATCAAGGCAATATCCAGCGGTCTCTGTGTTTCGAATT

CGGACGGTCCGCCGCCGCCGATCTATTGCATCGGACCTTTGATTGCAGCGGGAGACAGATCCGACGGCAA
AACCTTGAACAGACACGAATGCTTAAGGTGGCTCGACTCGCAGCCTAGTAAAAGTGTCGTGTTTCTTTGT
TTTGGGAGCTTAGGGTTGTTTCCGATGGACCAGTTGAGAGAGATCGCTATTGGGTTAGAGAGAAGCGGTT
GTCGGTTCTTGTGGGTGGTGAGGAGTCCTCCGCCGCCACCTGAAAACCAAACAGAGCGGTTTTTGGCTCC
TGCAGACCCGGATTTGGGCGCATTATTGCCGGACGGTTTCATGGACCGGACCGAGGGGAGGGGGTTCGTG
GTGAAGTCGTGGGCCCCACAGGTGGAGGTGCTAAGTCATGACTCTGTTGGTGGGTTCGTGACTCACTGTG
GGTGGAACTCGGTTTTGGAGGCGGTGTGTGCCGGCGTGCCAATGGTGGCGTGGCCGCTCTATGCGGAGCA
GAAGTTCAATAGGGTGGTGCTGGTGGAGGAGATGAAGTTGGCTCTACCGATGGAGGAGTCCGAAGATGGG
TTCGTTTGCGCTGGCGAGGTCGAGAAGCGAGTTACCGAGTTGATGGACTCGGTGGACGGGAGATTGGTGA
GGAAACATGTGTTGGCTGCAAGAGATGCCGCTAAGGCGGCGATTGGCGATGGCGGGTCATCTCGACTCGA
TTTGGGTCAATTAATTGAGTTGTGGACTCAGAACTGA
>GWHPAAAL025081
ATGGAGGATGCCATAGTTCTCTATCCTTCACCAGCAATTGGGCACCTCATCTCCATGGTGGAGCTGGGGA
AGCTCATACTCAACCACCAACCTACCTTCTCAATCATCATCCTGATCGCCACCCCACCCTACAACGCCGG
AAACACTGCTCCCTACATAAACCAAGTCTCCGCCACCACCCCCTCCATCCACTTCCACCACCTCCCCTCC
GTCCCCCTCTCACTATCCACCGCCTCCCGGAACCACGAAACCCTGGCCTTCCAACTCCTCCTCCTCAACA
ACCCCCATGTCCACCAAGCCCTTACCTCCATATCCCAAACCTCCACTCCCCGCGCCTTCATCATGGACTT
CTTCTGCACCCCTGCTCTCTCTGTCGCCAGAGATCTCAATATTCCGGCGTACTTTTTCTATACTTCCGGC
GCCGGAGGCCTCGCTTCTTTCCTTTATTTCCCCACCATTCACCGGAGCACTGCTGGAAACTTGAAAGACC
TTAAAACCTTCCTCCACATCCCCGGACTCCCGCCGTTTCGGGCGACTGACATGCCGAAACCGGTGCTCGA
CCGAGCTGACAAAGCATACGAATTCTTCTTGGATGTATCCTTAAACTTCCCAAAAGCTTCTGGAATCATT
GTCAACACTTTCGAATATCTTGAGCCTCGATCAATCAAGGCAATATCCGATGGTCTCTGCGTTTCGGATT
CGGACGGTCCCACGCCGCCGATCTATTGCATTGGACCTTTGATCGCTTCGGGAGATAGATCCGATGGCGA
AATCGTGAACAAACACGAATGTTTAAGGTGGCTCGACTTGCAACCTAGCAAAAGTGTCGTGTTTCTTTGT
TTTGGGAGCTTAGGGTTGTTTTCTTTGGAGCAGTTGAGAGAGATTGCTGTTGGGTTAGAGAGAAGCGGTT
GTCGGTTCTTGTGGGTGGTGAGGAGTCCACCGCTGCCACTGGAAAACCAAACCGAGCGGTTTTTGGCTCC
TGCGGACCCGGATTTGGGTGCGTTATTGCCGGACGGTTTCATGGACCGGACCGAGGGGAGGGGGCTCGTG
GTGAAGTCGTGGGCCCCGCAGATGGAGGTGTTGAATCATGACTCGGTGGGTGGGTTCGTGACTCACTGCG
GGTGGAACTCGGTTCTGGAGGCGATGTGTGCCGGCGTGCCGATGGTGGCGTGGCCGCTCTATGCGGAGCA
GAGGTTCAATAGGGTGGTCTTGGTGGAGGAGTTGAAGTTGGCTCTACCAATGGAGGAGTCCGAAGACGGG
TTCGTTTGCGCCAGCGAGGTAGAGAAGCGAGTTACCGAGTTAATGGACTCGGTGGAAGGGAGATCAGTAA
GGACGCATGTCTTGGCGGCGAGAGATGCCGCTAAGGCGGCGATTGGTGATGGCGGGTCGTCTCGACTCAA
TTTGGGTCGATTAGTTGATTCATGGACTCGGAACTGA
>GWHPAAAL025228
ATGTCTGTCCATGTCTTGATCTTCCCATACCCCGCCCAAGGCCATCTCCTCCCACTTCTGGACCTCACCC
ACCAACTAGCCCTCCATGGCCTAACCATAACCATCCTGGTCACACCCAAAAACCGCCCCACCCTTGACCC
ACTTCTCTCGACCCACCCGTCGATCGGAACCCTGGTCTTGCCCTTCCCGCACCACCCCGCCATCCCCTCC
GGCGTCGAAAACGTCAAGGACCTTGGAAATTCCGGAAATGTCCTCGTCATCAACGCTTTATCCAAGCTTA
GGGACCCGATTATCCAGTGGTTCAAGGCACACCCTAATCCCCCTGTTGCCATCGTCTCCGATTTCTTCCT
CGGGTGGACCAACGATCTGGCCGGTCAACTGGGTATTCCCCGCGTCGCTTTTTACTCCTCCGGTGCGTTT
TTAACCTCCATTTTGTTTCATTTTTACCGCGAGTTGAGATCTGCTGCCCCTGTCTTGACCGCGGTAAAAT
TCACTGATTTACCGCGGTCGCCGAGTTTTACTGAGGCGCATCTTCCCACCATAGTCCGGCACTACAGGGA
ATCGAATCCCGAGTGGGAACTTTTGAAGGAGGGCGTAATTGCAAATTCGTGGAGTTGGGGGTCCATTTTA
AACACATTTGATGATTTGGAGGGGGAGTTTCTGGAATACTTGAAAAAGAAAATGGGTCACAACCGGGTTT

ATGGAGTCGGGCCGCTCAATTTGGGTCGGGTCAATACGGATCCGGATGCGGATCCAAGTAATACCGTTTT
GGGATGGCTTGATGGTTGCCCCGATGGGTCTGTGATATACGTTTGTTTTGGGAGTCAAAAGTTTCTCAAG
AAGGCCCAAATAGAGGCTTTGGCTCTCGGGCTTGAACGGAGCAAGGCCCGATTTATATGGGTAGTCAAGC
CCATAACGGCCCAACAAGAGGCGGACGGGTACGGGTCAATCCTGGACGGGTTTGAAGAACGGGTATCCGG
GCGGGGCATGGTGATAAAAGGTTGGGCCCCACAGGTGAAAATACTGAGTCACCGAGTTGTTTACGGGTTT
CTGAGTCACTGCGGATGGAACTCGGTGCTCGAGGCGATAGCGGCGGGGGTGATGATACTGGGTTGGCCGA
TGGAGGCGGACCAATTTGTGAATGCTAGGCTATTGGTGGACTACATGGGGGCAGCAATCCGAGTTTGTGA
GGGGGCGGACTCGGTGCCTGACTCGGATGAGTTGGCCCGGACGATCGCCGAGTCAGTAGATGGGAACAAA
CAAGAGAAAGGGAGAGCAAGGGAATTGAGAGATAAAGCATGGGAAGCAGTTGGGCCTTGTGGGAGCTCTA
CAAAGGATTTGGTGGGCTTTGTGAAGGATCTGGCCCAACTTGATGAGAATGGTGTTTGA
>GWHPAAAL025545
ATGATTCCGACGCTGGACATGGCCAAGCTCTTCTCCTCCAGGGGTGTCAAAACCACCATCATCACCACCC
CTCTCAACGCCCCTGTTTTCTCCAAATCAATCCAGAGGTCCAACCAATTGGGTCTGGAAATCAGCTTAAA
AATCTTTGATTTTCCGGCGGTTGCCGCGGGCTTGCCGGAAGGTTGCGAGAGCGCTGACCAAATCACCTCC
GAGGACATGATTCCAAAATTCTTCGTCGCCACGGCGTTGCTCCGGGAGCCGCTGGAGCAGCTGCTAAGTG
ACCACCGCCCGGATTGTTTAGTCGCCGACATGTTCTTCCCTTGGACGACGGAGTCGGCGGCGAAATTCGG
CATTCCGAGATTGCTTTTCCACGGGATCAGTTTCTTCGCGCTCTGCGCCGGGGAGTCTTTGAGGACGCAC
AAGCCTTTCCGGCAAGTCACGTCGGATTCCGAACCCTTTGTCCTGCCAGAGCTCCCGCACGAGATAAAGC
TGACGAGAACGGAAGTGGCGCCGTTTGACCTGTACGATACCCAAACGGCCATATCCGGAGTCTTGAAACA
AGTGAGGGAGTCGGAGAAAACTTGTTATGGCATTATCGCAAACACCTTCTATGCACTCGAACCCGATTAC
GCCGATCACTACAGAAACGCCATGGGGAGAACGGCGTGGCACGTCGGACCTCTCTCTCTCTGCAACAGAG
GAATCGAAGACAAAGCAGAGAGAGGAAAAAAAGCTTCCATCGACGGACACGAATGTCTTCAATGGCTTGA
TTCAAAGAAACCCAAATCCGTCCTCTACGTCTGTTTCGGAAGCATGGCCAAATTCGCCGATTCCCAACTG
TACGAAATCGCCGCCGGTCTTGAATCCTCCGGCCAACAGTTCATCTGGGTGGTGAGAAAATCAAAAGACG
AAGAAGAACAGAAGGAAGAGACATGGCTGCCCCATGGATTCGAGGAGAGAACTCAGGGGAAGGGCCTGAT
CGTCAGAGGGTGGGCCCCCCAGATGTTGATTCTCGACCACGAAGCAATCGGCGGATTCGTGACTCACTGC
GGCTGGAACTCCACGCTGGAGGGAGTCTGCGCCGGACTTCCGATGGTGACTTGGCCGGCGTTCGCAGAGC
AATTCTACAACGAGAAGCTGGTGACGGATGTTCTGAAGATCGGGGTTTCCGTCGGAGTTAAAGAGTGGAA
AGCAGGGCCGAGGGAGGGGGTGAAGAGGGAGGCGATCGAGAAGGCGGTGAGGAGCGTAATGGAGGGAGAA
GAAGCGGAGGAAATGAGGCGGAGAGCGGCGGCGCTGAAGGAGGCGGCCATGGAAGCGGTTGAAGAAGGTG
GCTCTTCCTACTCTGATTTGACTTCTCTCATTGAAGAGTTGAGCTCCCGCCAAAAGATTAATGGAAAATA
A
>GWHPAAAL025754
ATGGAAAACCAAAAGAAAGGTAATGGCGGAGCGCACATACTGGCGATTCCTTTCCCTGCCCCAGGGCACA
TGAACCCTCTGATCCAGTTCTGCAAGCGCCTCGTCTTCAAGGGCGGAGTGAAAGCCACTGTTGCCATCAC
CAGGTACCAGGCCAAGTCCATCCAGACGAGACTCGACACCATACAGATCGACACGATCTCCGACGGCTAT
GACGAAGGCGGCTTCTACGTAATCGACAGCATCGAAGACTCCATCGAGAAATTCAAGGTTGCGGGATCTC
AAACCTTGGCCGAACTCATCGAGAAGTACGAGAAATTGGGGAATCCCATTGATTGCATCATCTACGATGC
GTTCTTGCCTTGGGTTCTTGATGTCGCCAAGAAGTTCGGGAAAATCGTGGCTGCTTTTTTCACCCAACCC
TGTGCCGTGAATTGCATCAATTATCACGTGTATCATAAATTGCTACCCTACCCTTTCTCTTCGCCGCCGA
TCTCCGTTTCCGGATTGCCTCCGCTTAATCTCGAGGATATGCCCGGGTTTATCTACGTTGATCCGGGTCA
CTACGTGCTGGTACAAAGCCAGTTCTCAAAAGCTGATGAAGCTGACTACCGTTTCGTCAATACATTCTAC
GATCTGGAGGCTGAGGCAGTGGATGCGATGAAGAAGATCCATTCTCTGAGAACAATAGGACCGACAGTCC
CGTCATTCTACCTGGACAAAAGAGTGCCAAATGACAGCAACTACGGGCTTCATTCCTCCAACTTGATCAT

GCAACAAAAGAAGAGCACGAGCACCCCCGACGCGGACCCCTTGGCCTGCTTGGACTGGCTGAGCACTAAA
CCACCCCGTTCAGTTGTGTATGTGTCGTTTGGTAGCGTGGCCGTGGCCAGCCCAGTGAGCGAAGAAATGA
AGGAAATCGCATGGGGTTTGAGGAACAGCAATTGCTTCTTCTTGTGGGTTGTGAAGGGTAACGAAGAGCC
GAATCTCCCTGAGAATTTCAAGGAAGAGACAGCTGACAAGGGGATGATAATTCCGTGGAGTCCCCAGTTG
GAAGTGTTGGCGCATGAAGCTACTGGGTGCTTTATAACACATTGCGGGTGGAATTCAACTATTGAATCGT
TGTGTTTGGGTGTGCCGATGGTGGCGATGCCTAAATGGGCGGATCAGACAACGAATGCCAAGTACATTGA
GGATGTTTGGAAAGCCGGGGTGAGGGCAAAGGTTGGTGAGAATGGAGTTGCTTCAAGGGAAGAAATCGAG
AGGTGTATAAGGGCGGTTTTGGAGGGAGAGAGTGGGAAGTATTACAAGAGCAATGCTATGAAATGGAAGG
ATTTGGCTACACATGCTGTGAGCGAGGGCGGGACTTCGGACAAGAACATTGCCGAGTTTTTGTCTGAATT
GAAGGCCCGTTGA
>GWHPAAAL025999
ATGGAGAAAACAACCCACGTAGCAATTTTGCCGACGCCGGGGATGGGTCATCTTATTCCGCTGGTGGAAT
TCGCCAAGAGACTCATCGACGGTTACAATTTTTCTTTCACTTTGATTCTTCCCACCGACGGGCCTCTGTC
GAAAGCCCACAAGGAGTTTGTGGATTCCCTTCCGGCGGGGATTGACCACGTTCTGCTGCCGCCGGTGAGT
CATGACGACATGACGGTGGAGAACCGGATTGCGCTCACCATCAGCCGGTCTCTCCCCGCTTTTCGCGACG
CGTTCAAGACGTTGGTCGACGAGAAGAAAGTGGCGGCGCTGGTGATCGATTTGTTCGGTACGGACGGTTT
TGACGTTGCGATTGAGTTCGGCGTGTCGCCGTTCATCTTCTTCCCGTCGACGGCGATGGCTCTGTCGTTG
TTTCTTCACTTGTCGAAGCTCGACGCTGAGACCGTCGGCGAGTACAGAGACCAGCCGGAACCGATTCGGA
TTCCGGGTTGCATACCGATTCAGGGGAAAGATATGCTGGACCCGGTTCAGGACAGGAAGAACGACGCCTA
CAAATGGCTGCTCCACCACGCCGGGAGGTACAAATTGGCGGAGGGAATCATGGTGAACAGCTTCAGGGAG
GTGGAACCCGGACCCATAACCGCTCTTCTGAAGAAAGAACCCGGTAAACCACCGATTTACCCCGTCGGAC
CGCTCATAAAGATGAGTTCGAACGCCGACGACGCCGAGTCGGAGTGTTTGAAATGGCTGAACGGGCAGCC
GAGTGGGTCCGTCCTCTTCGTCTCCTTCGGCAGCGGCGGGACCCTCTCATCCGTCCAGCTCAATGAACTC
GCCATGGGACTGGAAATGAGCGAGCAGAGGTTCATCTGGGTAGTCCGGAGCCCGAACGACGGAACCTCCG
CCGCCACCTACTTCAACGTCAACAGCCAAACCGACCCTTACAGTTTCCTACCGGAGGGATTCGTCGGCCG
AACCAGGGGGCGCGGCCTGCTCGTGCCGTCGTGGGCCCCGCAGGCTGCTATCCTCAGCCACGGCTCCACC
GGCGGGTTCTTGACCCACTGCGGCTGGAACTCCACCCTTGAGAGCGTCTACAACGGCGTGCCGCTCATCG
CCTGGCCGCTCTACGCCGAACAGAGGATGAACGCCGTGATGCTGACGGAGGATCTGAAGGTGGCGTTGAG
GCCCAAAACTGGAGAAAACGGGATCGTAGGCCGCCTCGAAATCGCGAACGTGGTGAAGGGTCTGATGGAA
GGGGAGGAAGGGAAGGGGATTCGGAGCAGAATGAGAGATCTGAAGGACGCGGCGGCGAAAGCCATCGGAG
AAGACGGTGCTTCTACAAAAGCACTTTCTGAAGTGGCATGCATATGGAAAAATAAGATTAATTACTGCTT
TAATTAG
>GWHPAAAL026143
ATGGACCACCACCACTTCCTCCTCATCTCTCTCCCTGGCCAAAGCCATTTAAACCCCACTATCCAGCTCG
GCAAAGTCCTCGCACGCGCCGGCGTCACTGTCACCTTCGCCACCACCGTCTACGGCCTTAACCAGCTAAA
GACTAAATCAACCACCGTCGACGGCATAGCCTACACCTCCTTCTCTGACGGTCACGACGAAGACGGCCAC
AAAGCTAGAAGCAACTTCGCCGGCTACATGGACGATCTAAAGCGCGTGGGGTCCCAAAACCTGATAAAAC
TCCTCGAAAACCTCGCCGGCGAGGGTCGTCCCGTCACCTTTATAGTCTACACCGTTCTCCTTCCATGGGT
TGCCCAGGTGGCCCGTGAGATGCACCTGCCGTCGGCATTCTTCGCCATACAGTGTGCCGCCGCTTTGGCC
GTGTATCACCGCTTCTTCAACAGCAGCGACGGACTCCACGGCGGCGAAAACGAAATAAACGACTCGATTT
CCGTCGAATTACCGGGACTACCGCAGCTGAGCTCCGGCGAGATCCCTTCGTTTCTACTGCCGGCCGATCC
GCAACATTCTTCTATCAATCAGCCTTGCCGAGAACACATACTCACCCTCGAAGAAGATCCAAATCCGACG
GTTCTGATCAACACATTCGATGCCTTAGAGGAAGATTCAATCAGAGCCGTCCGGAATATGAAGATCATCG
CCGTCGGCCCCTTAGTTCCGTCGGCCTTCTCCGACGGAATCGACGCGTCCGACAAATCATTCGGCTGCGA

TCTGTTTCGAAACTCCGGCGACCAATACCTCCCGTGGCTGGATTCGAAATCGGACTCCTCCGTCATCTAC
GTTTCCTTCGGGAGCCTAGTCGTTCTTGAGAAACCCCAGAAGGAAGAGATCCTGAACGGACTGATCGACA
CCGGGAGACCTTTCCTCTGGGTCATCCGCGACACCGCCGGAGAAGAAGAAGAAACGGCGGTGCCGGAAAA
TGGGCTGGTGGTGCCGTGGTGTACGCAGGTGGAGGTGCTGGCCCATTCATCGATCGGATGCTTCGTCACC
CACTGCGGATGGAATTCGACGATGGAGAGCCTGGTGGCCGGAGTTCCGGTGGTGGCGATGCCGCAATTCT
CCGACCAGTTGACGAACGCGAAGCTGGTGGAGGAGGTGTGGGGGAATGGGGTTAGAGCGGGAAAGAGTGA
AGAGAAGGGGGTTGTAGAGAGAGAGGAAATCAGGAGGTGCGTGGAGGTGGTGGTGGGGGGAGGAGAGAGG
GGAGAGGAGATAAGAGGAAGTGCTCGGAAATGGAGAGGTCTGGCCATGGCTGCCGTCAGGGAGGGGGGTT
CTTCAAGCAAAAATCTTAGAGAATTTTTGGATAGCTTGGGATAA
>GWHPAAAL026144
ATGCACCTGCCGTCGGCATTCTTCGCCATACAGTGTGCCACCGCTTTGGCCGTCTATCACCGCTTCTTCA
ACAGCAGCGACGGACTCCTCGGCGGGGAAAACGAAATCAACGAGTCGCTTTCCGTTGAATTACCTGGACT
GCCGCCGCTGAGCTCCGGCGAGATCCCTTCATTTCTAATGCCGGCCAATCAGTATCATTCATCTGTTAGT
CCGTCTTTCCGAGAACACATACTCACCCTCGAAGAAGATCCAAATCCAACGGTTCTGATCAACACCTTCG
ATGCCTTAGAGGAAGATTCAATCAGAGCCGTCCGGAATATGAAGATCATCGCCGTCGGCCCCTTAGTTCC
GTCGGCCTTCTCCGACGGAATCGACTCGTCGGACAAATCATTCGGCTGCGATCTGTTTCGAAACTCCGGC
GACCAATACCTCCGGTGGCTGGATTCGAAATCGGAATCCTCCGTCATCTACGTTTCCTTCGGGAGCATAG
TCGTTCTTGAGAAACCCCAGAAGGAAGAGATCCTTAACGGACTGATCGACACCGGGAGACCTTTCCTCTG
GGTCATCCGCGACACCGCCGGAGAAGAAGAAGAGACGGCGGTGCCGGAAAATGGGCTGGTGGTGCCGTGG
TGTACGCAGGTGGAGGTGCTGGCCCATTCATCGACCGGGTGCTTCGTCACCCACTGCGGGTGGAATTCGA
CGATGGAGAGCCTGGTCGCCGGTGTCCCGGTGGTGGCGATGCCGCAATTCGCCGACCAGTTGACGAGCGC
GAAGCTGGTGGAGGAGGTGTGGGGGAATGGAGTTAGAGCAAGAAAGAGTGAAGAGAAGGGCGTTGTAGAG
AGAGAGGAAATCAGGAGGTGTGTGGAGGTGGTGGTGGGGGAAGGAGAGAGAGGAGAGGACATAAGAAGAA
ATGCTCTGAAATGGAGAGGTCTGGCCATGGCCGCCGTCAGGGAAGGGGGTCCTTCGTGCAAAAATCTTAG
AGATTTTCTGGATAGCTTGGGATAA
>GWHPAAAL026243
ATGGAAAAGCTGACGGAAACAAGAAGATATCCAAAAGAGGTGGTGTTAGTCCCTTATCAAGCACAAGGCC
ACATGAGCCCCATGCTCCAACTCGGCACCCTCCTCCACTCCCTAGGCTTCTCCATCACCGTTGCGCACGC
CCGGATCAACTCGCCTGACCCCTTGACCCACCCGGATTTCGATTTCCGACCCATATCGGAGGACCTATCG
GATCGCGACACCTCATTCCATGGCCTACTCTCTCTCATCACGACTCTCAACACCGGCTGCAAAGAGCTGC
TCCGGGAGTGTTTGGTGCGGTTGAAGGAAGAACGGGGACCGGTCGCATGCGTGGTGTACGACTCGGTCAT
GTATTTTGCTGATGAGGTGGTGAATCAGATGAAGATTCCGAGCATGGTGCTTCAGACTTGTAACGCTGCT
TATGTTTTGCCCTGCCAGCTTCTTCCCCAGCTTAAGGCAGAAGGCTATCTTCCCTCTGAATCGGAAGATC
CTGCCGGGGACCACCTCGTGCCGAAGCTTTATCCCCTCAGATTCAAGGATTTACCCATTCTTAAATTTAA
CACACCCACTCTACAACTACTCACGACTCTAAGCGACATAAGAACATCCTCCGCCGTAATTTGGAACACC
ATTGACCACCTCGAGCACCCCTCTTTATGCCGACTCCGGCAACATTACAAAGTACCCATCTTCCCATTAG
GCCCCTTGCAAAAGATGGCACCCTCCTCTTCCGCCAGCTTTCTTAAAGAGGATTACGACTGTCTAACTTG
GCTTGAAAAACAAGCTCCAAAATCTGTTATTTATGTGAGTTTCGGAAGCATCGCAATGGTGGAGCCAAAT
GAGCTAAGTGAGGTGGCGTGGGGCCTAGCCAACAGCGGCCAACCGTTCTTATGGGTGGTTCGACCCGGTT
TGGTTGAAGGGTCAGAAAGGATCGAGCACTTACCGGAGGGTTTTAAAGAGACTGTTGGAGAGAGGGGGTG
TATTGTGAAGTGGGCCCCGCAGAAGGAGGTGTTGGCACACGAGGCGGTGGGAGGATTTTGGAGCCATTGC
GGGTGGAATTCGAGTTTGGAGAGCATTTCTGAAGGGGTGCCGATGATTTGTAGGCCGTGTTTTGGCGATC
AGAGAGTGAATGCTAGGTATTTGACTCATGTTTGGAGGGTAGGATTGGAATTGGAAAATAAATTGGAGAG
AGGGGCCATTGAAAGTGCTGTGAGAAGGCTTATGATAGGTAAAGAAGGGAAAGAAATAAGGCGGAAAGTG

TTGGATATGAAGCAAAACATTGAAGAAATAGTGCAGAAAGGCGGCTCTTCTTACAATACCCTGATTGAGT
TGGCAGATTTCATTTCTGATTTTCACAAAACAGGGCAATATTAA
>GWHPAAAL026244
ATGGAAAACCAGAAGGAAACAAGATATCCAAAAGAGGTGGTGTTAGTTCCCTTTCCAGTACAAGGCCACA
CAAGCCCCATGCTCCAACTCGGCACCCTCCTCCACTCCCTTGGCTTCTCGATCACCGTCGCACACGCCCG
TATCAATTCACCTCACCCGTCGACCCACCCGGAGTTCGATTTCCAACCCATATCGGAGGACCTAGCGGAT
CGCGACACCTCATTCAACGGCCTATTCTCTGTCATCATGACTCTCAACACCGACTGCGGAGAGCCGCTCC
GGGAGTGTTTGGTTCGGTTAACGGAAGATCGGGGGCCGGTCGCATGCGTGGTGTACGACTCGCTCATGTA
TTTTGCCGATGAGGTTGCGAATCAGATGAAGATTCCGAGCATGGTGCTTCAGACTTGTAATGCTGCTTAT
ATTTTGCCATGCCAGCTTCTTCCCCAGCTGAAGGCAGAAGGCTATCTTCCCTCCGAATCTGAAGATCCTT
CATGGGAGGACCTCGTGCCGAAGCTTCACCCCCTCAGATTCAAGGATTTACCCTTTCCTAAATTTGACAC
ACCCACTCTACAAGTACTCATGACTCTATGCGATGTAAGAACGTCCTCCGCCATAATTTGGAACACCACC
GACCACCTTGAGCACCCCTTCTTATGCCGACTCCGGCAGCATTATAAAGTACCCATCTTCCCATTAGGCC
CCTTGCAAAAAATGGCTCCCTCATCTTCCACCAGTTTTCTTAAAGAGGAATATGACTGTCTAACTTGGCT
CGAAAAACAGGCTCCAAAATCTGTTATTTATGTGAGTTTCGGAAGCATCGCGACGGTGGAGCCAAATGAG
CTAAGCGAGGTGGCGTGGGGCCTGGTCAACAGTGGCCTGCCGTTTTTATTGGTTGTTCGACCCGGTTTGG
TAAATGGGTCAGAATGGATCGAGCACTTGCCGGAGAGTTTTAAAGAGTTTGTTGGAGAGAGGGGGTGTAT
TGTGAAGTGGGCCCCACAAAAGGAGGTATTGGCACACGAGGCGGTGGGAGGATTTTGGAGCCATTGCGGG
TGGAATTCGAGTTTGGAGAGCATTTCTGAAGGGGTGCCGATGATTTGCAGACCGTGTTTTGGCGATCAGA
GAGTGAATGCTAGGTATTTGACTCATGTTTGGAGGGTAGGATTGGAATTGGAAGATAAATTGGAGAGAGG
AGCCATTGAAAGAGTACTGAGAAGGCTTATGACGGGTAAAGAAGGGGAAGAAATAAGGCGGAGAGCGATG
GACATGAAGCAAAACATTGAAGAAGTTGTGCAGAAAGGCGGCTCTTCTTATAATTCCCTGATTGAGTTGG
CAGGTTTCATCTCTGATTTTCACACGACAAAGCAATGTGTTTAA
