# Supplementary material for: Genomic-Wide Identification and Characterization of the Uridine Diphosphate Glycosyltransferase Family in Eucommia ulmoides Oliver
Source: Plants (Basel). 2021 Sep 17;10(9):1934. doi: 10.3390/plants10091934 (PMC8471388; doi:10.3390/plants10091934)
Supplement: Supplementary file 1 [file plants-10-01934-s001.zip › plants-1355377-supplementary/Supplementary File 2 (aa).pdf]

List of the amino acid sequences of *Eu*UGTs

>GWHPAAAL000151
MVPISTEPIRKCHVVAVPYPGRGHVNPMMNICKLIASKFDPDRLVITFVVTEEWLGLIGSEIKPANVRFA
AIPNVVPSERVRAADMVGFVTAVMTKMEQPFEEVLDRMQSPASVIIADTFLRWAVEVGNRRSIPVASLWT
MPAMVFSVFYNFELVVENGQFPIELCERGEERVDYLPGIPSIRLSDLPTIIHRRDQNLLRLCLTAFANVS
RARYLLFTTCSDLEPQVIAALKTQFPFPLYCLGPTIPYFNLQQPPPITPNHHDYLKWLDSKPPNSVLYVS
LGSFLSVSTSQMDEIEAGLGGSGVNYLWVNRGHAGGVGGGGRMVVRWCDQLRVLCHPSVGGFWTHCGWNS
TMEGIYAGKPMLTLPILMDQVPNSKCVVEDWKIGWEVKREEGNLVSREKIVEVVRRFMDEGSEEREEMMR
RAEELRRLCRTALVRGGSSHENLQAFVDGIAKF
>GWHPAAAL000153
MAERGEERVDYLPGIPSIRLADLPTIIHQSDQNLLRLCLTAFANVSRAQYLLFTTCSDLEPQVIATLKTQ
FPFPLYCLGPTIPYFNLQQPPQTTPNHHDYLKWLDSKPPNSVLYVSLGSFLSVSTSQMDEIEAGLGGSSV
NYLWVNRGDAGGVGGGGGMVVRWCDQLRVLCHPSVGGFWTHCGWNSTMEGIYAGKPMLTLPIVMDQVPNS
KCVVEDWKIGWEVKREEGNLVRREKIVEVVRRFMDEGSEEREELVRRAEELRRLCRTALGRGGSSHENLQ
AFVDGIAKF
>GWHPAAAL000493
MEQREYRAHVLAIPFPAPGHVNPLLQFCKRLEFKGLKATLAITKFLSKSMQSRLGSIKIDTISDGYDEIG
RHNAESLGVWISTFKEVGSKSVADLIHKFRSLGHPIDCIVYDSFLPWVLDVAKENGVAGASFMTQKCAVN
HINYHVYHKKIPYPFSSPTYSIPGLPSLDLEYMPGFMLVHPDYYELVLSQFSTVDRADYVFVNTFYKLEA
EVLDEMSKVLPVKPIGPTVPSFYLDNRVEDDKNYGLNLHQLDSSVCLNWLSSKPARSVVYVSFGSVAISS
PAANEMEEIAWGLKNSNCSFLWVVKANEEPNLPQNFKEEMSEKGLIVAWSPQIEVLSHHSVGCFLTHCGW
NSTIEAICLGVPMVAVPKCTDQTTNSKSIQDVWKVGVKVKASEDGIARREEIERCIKVVMEGESGEELKK
NAAKWSELAKEAVSEGGTSDENINEFVSQLTNSSSTQS
>GWHPAAAL000531
MERDLDEMGSKKPHVLVIPFPAQGHFSPALQLSKRLASKGLRVTFVTSMSSAKSSSSIRMEQIGDGYEEG
EEPESKEAFTEHIQTVFSQSLGDIIEKQKQIGYPIKLVIYDSVMPWALEIAHKLGIYGAPFFTQPCAVCA
IYYHMQQGTLEVSLEGTKVSLPSLPPLEKNDLPSFISNMFSHEFLTKTILGQFSNFHEADWILFNTFDKL
EDEVVKWMASQWPIKTMGPTIPSLYLDKRLKEDKEYGLSLFTPNTEASMRWLDTKEQGSVVYVSFGSLAI
LDETQMEELSWGLLQTNNYFLWVVRASEESKLPNNFMAKTSEKGLVVDWCPQLEVLAHQAVGCFVTHCGW
NSTLEALCLGVPMVAMPQWVDQTTNAKFVADVWKTGVRVEVNDKGIVTREEIKKRIGEVMVGETREELRN
NASRWKNLTREAVDEGGSSDKNIEEFISKLVCS
>GWHPAAAL000532
MERDLDEMGSKNPHVLVVPFPAQGHFSPALQLSKRLASKGLRVTFVTTISNAKSRGSIRMERITDDYEEG
EEPESIEAFIERMKVVISQSLGDIIEKQKHAGYPVKLVVYDSIMPWALEIAHKLGVYGAPFFTNPCTVCA
IFYHMQQGTVEVPLEGTKVSLPSLPPLEENDLPSFISNLVAPEFIKKMSLGQFSNFHKADWILLNTFDKL
EDEVVKWMASQWPIRTIGPTIPSMYLDKRLKEDKEYGLSLFTPNTKASMKWLDTKEQDSVVYVSFGSLAT
LDETQIEELSWGLVNSNNYFLWVVRASEESKLPSDFMAKTLEKGLVVNWCPQLEVLAHQAVGCFVTHCGW
NSTLEALCLGVPVVAMPQWGDQTTNAKFVADVWKVGVRVEANDKGIVTREEIEKQIGVVMAGETREELKK
NASSWKNLAREAADEGGSSDKNIEEFVYELVCS
>GWHPAAAL000534
MARDLDEMGSKKPHVLVVPFPAQGHFSPTLRLSKRLASKGHRVTFVTTISSAKSSGSIRMEQITNDYEEE
IAHKSGVYRALFFTQPYDVCAIFYHMQQGTLEVPLEEIKVSLPSLPLLEENDMPSFISNLFSHEIIKKTV

LGQFSNFHKADWILLNTFDKLEDEVVKWMANQWPIKTIGLTIPSMYLDKQLKEDKEYGLSLFTPNIETSM
KWLDTKEQGSVSNNYFLWVVRASEDSKFPNNFMAKTSEKRLVVNWGPQLEVLAHQAVGCFVTHCGWNSTL
EVLCLGVPMVAMPQWTDQTTNAKFVADVWKAGVRVEVNDEGIITREEINKRIGQVMAGETREELRKNASS
WRNLAREAVDEGGSSDKNIEEFVSKLVQMPMYNL
>GWHPAAAL000847
MASKPHFILVPLLAQGHMIPMIDTAKLLAHQGVLITIITTPVNADSIKPTVNRAVNTGLEIRVARLRFPC
AEAGLPEGCENLDMLPSPEAGIKFFNALGMLKAPMENLLGEIEPSATCIISDICPPWTMHVAKKFASREL
FSPAPIELTKVQVPCTTNPTSAGLKDLSDQIVEAEQAAYGMVVNSFEEMEPEYVQYYKQVTGKKVWCIGP
VSLCNKLDSDKAERGKRASIEDTDCLKWLDSHNPDSVIFACLGSLHYLSTSQLIELGLALESSNRPFIWV
IGFVSDELGKWLSEQRFEERNKGKGLIIHGWAPQVLILSHPSIGGFLTHCGWNSMLEGLSAGVPMITWPL
HSEHFYNEKLILQVLRVGVRVGVEFPVLIGEALNVGFIVKKEEIEAAVERLVGGKEAGEIRKRVGELQGK
AKMAMKDGGSSHMRLEMFIQDMVEQSSLQKI
>GWHPAAAL001759
MENKELVFIPIPAMGHLISMLETAKLLVNRDQRLSATVLLFKLPQDAGFATYMQSLSSSAFAARLRLVIL
PVPEKPPSESSKPDIIESHMPNVRNAVREIGESRVAGFVVDMVCYKMIDVADEFGFPTYTYFTSSAGYLG
LLFHFQSLKDPYNLASNYKDWDSDKKELLVPSFVNPVPEKILPLEMLDHQVPLVLELARRLVEPKGVLVN
TFSELESHALHSLSGGNNPPVYPIGPILLASEPRYNCLQGNEESIMKWLGKQLPSSVVFLCFGSAGSFPE
AQIKEIAYALENSGHRFLWSLRRPQAEGQRYPTEYDDPSEVLPEGFLERTINTGRVIGWAPQVAVLSHPS
IGGFISHCGWNSLLESLWFGVPVAAWPIYAEQHVNAFQMVRELGLAMEIKMDYRKTNSVLVTAEEIESGI
RRLMAEECGGGGGGIREKLRDMKENGRMAVAEGGSSYISLSRFIENVLGPHTQ
>GWHPAAAL001760
MENKELVFIPMPAMGHLVSMLETAKLLIKRDQRLSVTVLIFKLPFDTGIDTYIQSLSSSSSATRLKLVNL
PDPQKPPSEEEEEEIPKSFLSDLIDSHKPHVRNAVRDIGESRVAAFVVDMVCYNMIDVAGEFGLPTYTFFP
SSAGFLGLLFHLQILMDHHNRDVSDYRDWDLDKELSVPSYINPVPAKVLPLAMLDKQEGGVSPVVTLARR
LRETKGILINTFPELESHALHYLLSGNNPPVYPIGPVLLVPERSFNGVHGYNEGIMIWLENQRPSSVLFL
CFGSAGSFNEAQIKEIAHALENSGHRFLWSLRRPPPEEKKIELPTDYTDPLEVLPEGFLERTVNTGRLIG
WAPQVAVLSHPSVGGFISHCGWNSVLESLWFGVPMAAWPNYAEQQINAFQMVRDLGLAVDIKMEYRKENP
VLVKAEEIESGIRRLMTEESGGGIREKLREMKEKGRMAVAEGGSSYTALAQFIENVLRPSQMIDRKW
>GWHPAAAL001761
MENNELVFIPMPVMGHLLSMLETAKLLINRDQRLSVTVLLFKLPFDTGVDNYIQSLSSSASTSRLRFINL
PDTEKPSSEEIPKTFMIDLIKSLKPHVRNAVREIGESRVAGFVVDMFCHDMIEVAEEFGLPTYAFFTSSA
GFLGLVFHVQILKDHHNQDISDYKDCDKELSVPSFVNPVPAKVLPTTMLEKEEGGLSIVLSLARRLRETK
GILVNTFAELESHAFNSLSCGNNPPHYPIGPILLEPERSDESVQGYEESIMNWLDDQPPSSVLFLCFGSM
GSFKEAQIKEIAHALENSGHRFLWSLRRPPPEGKKIGFPTDYDDPSEVLPEGFIERTADNGRVIGWAPQV
AVLSHPSIGGFVSHCGWNSTLESLWFGVPIAAWPIYAEQQINAFQMVRDLGLAVDIKMEYRKENPVLVTA
EEIESGIRRLMAEESGGGIREKVREMKEKGRMAVAEGGSSYTALSRFIENVLQPHT
>GWHPAAAL002229
MKSDAIVLYPAPSIGHLVSMVELGKLILNRYSHKLGFSIIVFLTTGNLSDNETTSYIHHVSNTNPSITFR
RFPFVSVDTSPTRSPAAIHFDFIRLNADNVLHAIREIPNVIRALVIDFFCASAIPIARELRHPVYFFFTS
GAYALAFYLYFPTVHKQVDQSFKHLNKTFLQFPGFPPIRASHVPEPVLDRDDPAYADMVCFCDHLPKSDG
IIVNTFDALEPMACEAIASGACVPDATTPPIYCIGPLIANTSEGGGENGNASLGRSSEREAKHEWLSWLD
AQPKGSVVFLCFGSRGSFTRRQLKEMAHGLERSGQRFMWVLKSPPCDAAAPATEFCLEDVLPEGFLERTK
KTGMVVRSWVPQVAVLNHGSVGGFVTHCGWNSVLEAVVAGVPMVAWPLYAEQHVNGAALVENMKMAIPLG
QEKEEDDDGFVSAAELEKALRELMESEGGRERSLKMREMAMAAWENNGSSTTSLAKFVEWLEGS

>GWHPAAAL003558
MALIPASDKPHAVCIPLPAQGHINPMLKLAKILHSRGFHITFVNTHFNRNRLLRSRGPSALDGLPDFRFD
SIPDGLPPSDADATQDIPSLCESTPKHCLEPFCDLITRTNEDAGVPPVSCIVSDAVMSFTLKAAERFGLP
EVLFWPTSACGLLGYTQYHKLVERGYTPLKDISYVTNGYMETTIDWIAGMKDIRLRDLPSFIRTTDPKDT
MLNFLISECAAIPTASALILNTFDALERESIDALSAVHPRIYTVGPLNLMRNQINDDRIESIGSNLWKED
PGCIEWLDSKEPGSVVYVNFGSITVVTTGQLTEFAWGLANSGRPFLWILRPDIVAGDKAMIPPEFLAETK
GRGMLSSWCLQEQVLRHQAVGGFLTHSGWNSTVESLCGGVPVICWPFFAEQQTNCRYSCREWGVGMEIDN
NVKRDEVEATVRELMEGEKGKEMKRRAAEWKKKAEEATGAGGSSQVNLEKLFSEVLLVNSDE
>GWHPAAAL004075
MSDSGNKRPHVLVFPYPAPGHIIPLLDLTQLLLSSGLTVTVIVTPEFVSLLDPLLSSHPSSSIQPLVLSL
PKAPASLVPSLRATGELYDPILQWFKSHPSPPVAIVSDFFLGWTEKLGKELGVPRLVFWASCALSSLISS
SIWRDRPKVDDPADENFLLSFPEIPNSPKYPLWQMSTLFRMFKEGDPDWEFIREGMVANTVSWGVVFNSF
TELEGVYIDHTKKEMGHDRVWAVGPLLPLDGAPTSRGGSSAVAPHKVTVWLDDKPDDSVVYICFGSRMEL
TTAQRDALAAALESSGVHFIWIVKAGIDDGDPIPNGFEDRVAGKGLIIRGWAPQVPILMHRATGAFVTHC
GWNSVLEGISAGVMMLTWPMGADQFYNANLLVDELHVAARACEGGDEKVPDSSELARLLAESVSGKRLER
SRVVQLRDAAVEAVRGGSSSKDIDDLVKELGELGCGKA
>GWHPAAAL004076
MAISGSGKHVLIFPYPSQGHLIPLLHLAHRLAARGLTITILVSPQTLPLLHPLLSDHPSMETLVLPFSTH
PSIPAGIYDCRGHLPDSVRSTLFAISDLYEPVLDWFRNHPSPPVAILSDKFMGWTKISHANSEFIDWRSP
LSAPWPSHWFIPIGEIYRRETSAAVKMTHWFHFMRSYVEGDPISEFIKEGMQANMASWGLVINTFNELEG
VYLDHLKEKMGHDRVWAQMEAIAIGLEKSEAYFLWSIRGSTEGVIDGEFGEVPAGFEDRTSARGLVVKGW
APQLSILRHRAVGSFLTHNGWNSTLEALVAGVPMLAWPMGAEQFMNATLLVDQAKVAVRVAEGARDVPNP
FELARAVTRVMDGRSVERVQAVRLREEALNAVKEGGSSYQNFDGLAMHLFNNE
>GWHPAAAL005635
MATFQSESSPHILLFPFMSKGHTIPILHLARLLIHRNAAVTIFTTPANRPFVCHSLSDTTATVVDLPFPE
NIPGVLPGVESTDKLPRLPWLDRGIGGRIRHPAARYYGMNNFSGAICHDVMTHRDLIEPGSGDELFPVPG
FPWIKVAGNDFEPALSASPPAGDGYGLQWEFTIEVVEATSKSYGVVVNSFYELEPAYLNYWNSNIRPKAW
CVGPLCLAERPRVKPENHRKPSWIQWLDEKLEEGKPVLYVAFGSQAEISPEQFREIKTGLEKSESNFLWV
VRGGLSELIDDDGFEERVRERGLVVREWVDQREILGHGSVKGFLSHCGWNSALESICAAVPILAWPMMAE
QPLNARMVVDEIKVGLRVEACDGSVRGFVKWESLEKMVRELMEGEMGKEVRKKVKEVGAAAVAAVRGGGS
SWKTLNELINELS
>GWHPAAAL005636
MATFQSESSPHILLFPFMSKGHTIPILHLARLLIHRNAAVTIFTTPANRPFVCHSLSDTTATVVDLPFPE
NIPGVLPGVESTDKLPRLPWLDRGIGGRIRHPAARYYGMNNFSGAICHDVMTHRDLIEPGSGDELFPVPG
FPWIKVAGNDFEPALSASPPAGDGYGLQWEFTIEVVEATSKSYGVVVNSFYELEPAYLNYWNSNIRPKAW
CVGPLCLAERPRVKPENHRKPSWIQWLDEKLEEGKPVLYVAFGSQAEISPEQFREIKTGLEKSESNFLWV
VRGGLSELIDDDGFEERVRERGLVVREWVDQREILGHGSVKGFLSHCGWNSALESICAAVPILAWPMMAE
QPLNARMVVDEIKVGLRVEACDGSVRGFVKWESLEKMVRELMEGEMGKEVRKKVKEVGAAAVAAVRGGGS
SWKTLNELINELS
>GWHPAAAL005637
MAYPPPSPALHIALFPFMSKGHTIPIIHLTRLLLNRGHKTTVFTTPANRPFISDSLTGTDASVIVLPFPE
NIPGIPSGTESTDKLPSMSLFVQFANATKLIQPHFEQAIKSLPNLNCMISDGFLSWTLQSASKFGIPRLV
FYGMNNYSMALNYDVTVNRLLSIPKSDNEPFVVPNFPWIKLTKNDFEEPFNEVEPKGPYVEFTIEQSLAT
FNSYGLIVNSFFELEKPFVEYWNRASKPKAWCIGPLCFAEPEMIIERKRKPKPKWIDWLDQKRDQGKSVL

YVAFGSQAEISQKQLHEIAMGLEKSEVNFLWVMRKKDTAIEGVPERGMVVRDWVDQREVLAHEGVRGFLT
HCGWNSVLESIWARVPILAWPMIAEQHLNARMVVEEIKIGLRVETIDGSVRGFVKRESLEKMVRELMEGD
MGKEVRKKVEEVGEAAGKAMTEGGSSWDALNQLIDEIKST
>GWHPAAAL005638
MASPPRSPTPHIALFPLMSKGHTIPIIHLTRLFLNHGFKTTVFTTPTNRPFISDSLASTDASVVILPFPE
NIPRIPSGTQSTNKLSSIGSHQVSPQSRLHDIGRLPHKNDFDEPFNEIEPKGPYAKFIMEQGVADSNSYG
MIVNSFYELERPFVEYWNREGKLKAWCVGPLCFAEPEMIIKRQSNPKPKWIDWLDKKRDQGKSVLYVAFG
SQAEISPEQLHKIVMGLEKLEVNFLWVKRMKDTYIVGMTERGMVVRVWVDRREALAHEGVRGFLTHCGWN
SVLESIWARVLILVWPMMAEQQLNTRMVAQEIKIGLRVEMIDGSLRGFVKRESLEKMVRELMEGDMGKEV
RKKVEEVGRVLLGCSESVY
>GWHPAAAL006268
MTRKEVHVLVVTLPFQGHINPMLRLAKRLVSKGLYVTFATTETARQRLLGGSSAATSITRFQFEFFKDGL
GPEVDRDKNLDILLLEALPTKGVENFSNLIADLSKRGRKFSCVINQPFVPWSAAVAAEHGIPTATLFIQP
FSLFSIYYRYHKGLNLFPSLENPNNTLKLPGLPLMKVSDLPTFVRPESPYHFRKLLAESVKSLEKVKWVL
GNSFQDLEKGVLEAMEEDSINNWLPVGPFLASALLGEKEEGNVAVGGADMWKAEDSCLEWLDGKPVSSVV
YVAFGSITVLDGKQMESMAAALKNSGKPFLWVVRSPAKDSDEKKSNGLPEGFIEEMKGRGLVVPWCHQEK
VLMHPAVACFVSHCGWNSSLEAVVAGVPVIAFPEWTDQPTNAQIGVRLGENDRDFSSEEVERCIRAVTDG
PGAAEIKRRAAELKEAARKAVADGGSSERNLNRFISEISA
>GWHPAAAL006282
RHQRRRVVLFPLPLQGHIPMVQLANILYSKGFQISIVHTHFNCPSRLKYPHFTFHQISDGLSETEASTED
IIALVSLLNKKCVGPFRDCLIQLLSDDSSDPIACLITDAIWHFSQAVAEGLKLRRLVLRTSSVCSFLAFA
ALNLMRDKGYLPKQDSRLEEIVPKLPLLKMKDLSFIKTKNPEDLYHSVGGMITNTRESSGIILNSFKELE
ESSLSILKHDFPIPNFPIGSFHKYFSATSSSLLEQDRRSISWLDTQKPNSVIYVSFRSIATIEETEFLEI
AHGLVNSNQPFLWVVQLKLVRRSDWVELLPDGLIECINRREHIVKWAPQQEVLAHPTTRGFWTHCGWNST
LESICEGVPMICSPCFGDQFVNSRYISDVWKIGVELKNLEKEDIKRAIRRVMVEKEWEDMRFRIMELKEK
VDCSIKRGSSTESLQSLINYILSL
>GWHPAAAL006284
MDQETTLPPHVLIFPLPLQGPVNSMLKLAELLSISSRFHITFLVTDHIHRRLLQHTDIKSRFEKYPGFRL
ETISDGLSEEHPRSGDRLMELFDSLNTETKKLFRELLNSGRLISGNCRPITCIIADGIMGFTCDVANEFR
IPIIYARTVSACCLWVFFCLPKLIEVGEIPFQGNDLDVPIRSVPGMEGYLRRRDLPSFCRSGNVAGPNVQ
VVYLSESQENPRAHGLILNTFEDLEGPILSQIRTLCPNLYTIGPLHAHLKTKLAALRSLAPASSNSLWEED
RSCLAWLDVQPPKSVVYVSFGSLAVMSKDQLLEFRHGLVNSGQRFLWVIRPDSGAGEGLIPADTNNRGYI
VGWAPQEEVLSHASVGAFLTHSGWNSTLEAIYEGKPMICWPYFLDQQVNSRFVEKVWGLGVDMKDICDRV
VIEKMVRDLMDERGGGYKQNADQIAKLANECFKAGGSSYKSLENLVKDIELMMKSYCAEFGHCVKVRTRK
NQPHKREREREEERVVLKPCALFRSNADWRTIHRLIKLKFGQQVRNSVLYNLNGGNRILGSATTVSAGLF
WVVFKPAFW
>GWHPAAAL007266
MASKTDRLQFLLIPLMSQSHLIPLTDFAKLVARRGISVTIITTPLNVHRFKSIIGRTPNADLKIHLLSIP
FPCKEAGLPEGVENVDSLDSPESAGKFFLASSLLKYPLEKLVSEMDAKPNCIVSSNALPWTVKLGEKFGI
PTYFFNPISCFTVVCSTNIAIGKPHEAVSSDSEPFAVPDMPHRIEFTRAQLPENMRKGDSTDDVKAVMQQ
IRDAVSSARGVLVNSFEELEAPYIQSYKKTVKNLWCVGPVSLSNDESPPSSTTDERIDCLKWLDSMNPSS
VIYACFGSISRIPASQLIEIGLGLEASRTPFLWIIKEKDISEPVQKWLDDEKFEERIRGRGLLIKTWAPQ
VQILAHPAVGGFLTHCGWNSTLEGVCAGLPMITWPMIAEQFYNEKFIVNVLGVGVRIGAEGGMESGAFVG
REEIKKAVVELMGGEEEREGGGDRRRKARELGEMAKRAVADGGSSHLNISFLIQDVMQQS

>GWHPAAAL007326
MGSQKPHVLVVPFPAQGHFSPALQLSKRLASKGVRVTFVTTMSWAKSIGSIRMEQISDGYEERENPESIE
AFAKRIKTVISQSLRDLIEKQKQVGYPVKLVVYDSAMPWALEIAHKWGVYGAPYFTQHCTVCAIYYHMQQ
GNLKVPLEGTKVSLPSLPPLEKNDLPSFICNLFPHEFLMKEMLNQFSNFHNADWILFNTFDKLEDKVVKW
MASQWPIRTVGPTIPSMYLDKRLEGDKEYGLSLFTPNTEASMKWLDTKEQGSVVYVSFGSLATLDETQME
ELSWGLVQSNNYFLWVVRTSEESKLPSNFMDKTFEKGLVVNWCPQLEVLAHQAVGCFVTHCGWNSTLEAL
CLGVPMVAMPQWVDQTTNAKFVADVWRVGVRVEANDKGIVTKEEIEKRIREVMAGETREELRKNASGWKN
LAREAVDEGGSSDKNIEEFVSQLVCS
>GWHPAAAL007393
MADDSKLHFVMFPWLAFGHIIPFLELSKFLAQKGHKISFISSPRNIDRLPKIPTNLPIKLVKIPLPPIEE
LPENAEATMDVRTDDIHYLKKAYDGLESGLTRFLESESPDWIVMDFAPHWLPPIAAKLGISRVFFSIINA
WFLVFLGASSEAMINGSDERTAPEDFTVPPKWVPFPTKAAYKLYEANWAFQSTKLNASGFTDMYRCGSVI
QGIDAILVRHCNEFEPEWLTLLENLHGRRVIPVGLMPPRRQNNQDDTNETWVSIRDWLDSQNRCSVVYVA
LGSEVALSQEQLSELALGLELSGLPFFWALRIPRSNPVDLPDGFEERMKSKRQGMVWKAWVPQVKILSHD
SVGGFLTHCGWSSIIEGLMFGKPLIMLPFLVDQGLNARLLAAKEAGVEVDRDEESGAFTKETVAEALRLV
MVESERGKVLNRGKAKEMGVVFGDKDLHDLYLENLVVYFENHRRLCKES
>GWHPAAAL007798
MASQKNDNGAHVLAIPWPAPGHMNPLLQFCKRLVFRGGIKATFAITKFQSKSMHSRLDSIQIDTISDGYD
DGGFFITAGIQESVEQFKAVGSKSLAELIKKHESMGQPFDCIIYDAFVPWVLEVAKEHGIAGAAFFTQTS
AVNCVNYHVYHKLIPYPFDSPPYSLPGLPPLGLQDMPGFMTVDPYVYELVLSQFSTPQQADYLFVNTFYH
LEAEAVEAMLKVHPLLKTIGPTVPSYYLDNRIENDKSYGLHATHLMFQEKNSSQTDPSLVLDWLNTKPHA
SVVYVSFGSIALASPSTDEMKEIAWGLKNTGCYFLWVVKANEEPNLPKNYREATREKGLIVSWSPQLEVL
EHEALGCFVTHCGWNSTIEALCLGVPMVAMPKWADQPTNGKYVQDVWKIGERVKVDENGVAGKEEVERCI
RAVMEGESGKEFKKNAAKWKELAKEAVNEGGTQDKNIDEFASKLISSSHRS
>GWHPAAAL007799
MATQTKEYGAHVLAIPWPAPGHMNPLLQLCKRLVLKGGIKATFAITKFQSKSMYSTLDYIQVDTISDGYD
EGGFFIVDGITASVEKFKAAGSESLAELIRKHEGLGQPFDCIIYDAFLPWVLDVAGEFGISTAAFFTQKC
SVNCINHHVLKKLIPYPFTSEPISIPGLPLLNIEDMPGFIHVDPELYELVQSQFSTPELADYRLVNTFYE
LESEAVEAMSKIHSLRTIGPTIPSFYLDKRIENDSSYGLHATHLMFQEKNASRVEPSVVLNWLDAKPTGS
VVYVSFGSVSIASPGPDEMKEIAFGLKNTNFHFLWVVKPNEEPNLPENFKEEMADKGLIVSWSPQLEVLA
HKALGCFVTHCGWNSTIESLCMGVPMVAVPKWADQPTNAKYVEDVWKVGIRVKVDENRFAGRDEVERCIR
AVMEGERAEDLKRSALKWKELCIKAVAEGGSSDKNMEEFASELITRKSASK
>GWHPAAAL007800
MEEKQGTSDRVHVLVVPFPVQGHINPMLQFAKRLASSGLKITVIGTTKTTVSRSIPNIEIERIPDAPGEA
ADQQNTETIEEHCERLTTVLSNFSVKLIEKQSGLGCPYRVLVYDSVMPWALELAHRRGLKAAPFFTQSCS
VSALYYHMNRGSLKVPPVGSSEFVEVPALPPLKVGDLPSFIYDMGSYPFILKMVVNQFLNFEKADCLLFN
TFDKLETEMVTWMANQCPTIRTIGPTLPSVYLDKRLNEDTDYGLSLFNPDRESSIQWLHSKPTASVVYVS
FGSLANLSETQMHELAFGLERTDSHFLWVVRSSELTKLPPGFPTAPEKCAVVNWCPQLEVLSHPAVGCFV
THCGWNSMLEALSLGVPVVAMPQWTDQTTNAMLAVEVWGVGVRTAADKKGIVRREEVEMRVREVMEGNGR
EGLKSNAGRWKELAREAMDGGGSSD
>GWHPAAAL007936
MAENTHVVMLPWSAFGHIIPFFHLSIALSKAGIHVSFVSTPKNIRRLPQIPPDSVPFIDIVELPLPEIDR
SLLPEDAEATVDIPTENIQYLKIAYDLLKKPFSRFVADRSPDWIVIDLMPYWAADVAKEFDIPVIYFSMF
CAAASAFFSRPEFIAGEGQKMKRSTPESLTSTPEGVDFPSTVAHRHHEPLSFHGENASGISDASRFARTL

HSCRALAIRSCPEFEHDDLNLLEKIHQKSVIPVGLLLPEQKSEARNSGDEFKIFKWLDQQKPKSVIFVGF
GSECKLTENQIHEIAYGLELSELPFLWSLRKPSYSTDEVDPLPPDFHRRIAEKGLVHVGWAPQTEILAHP
SVGGSLFHSGWGSVIETLQFGHRLVVLPLIIDQPLNARLLVEKGLATEVERGGDGSFGRDAIAKALREAM
VMEGGERVREAMEIFGDRKLQENYVERFAEYLKNGVAKKNQ
>GWHPAAAL007941
MAKNTHVVMLPWSAFGHIIPFFQLSIALTKAGIHVSFVSTPKNIRRLPQIPSDSVPFIDLVELPLPEIDR
SLLPADAEATVDIPTENIQYLKIAYDLLKKPFSRFVADRLPDWIVIDMMPHWAVDVAKEFDVPVIYFSVF
GAAAAVFWGPPEYIAGEGQKMKRSTPESLTSTPEWVDFPSTVAYGHNEAVSFHAGAYGENASGISDAGRT
ARILHSCRALAIRSCPEFEHDYLNLLEKILQKPVIPVGLLPPEQKSEPRNSGYEFKIFKWLDQQKPKSVI
FVGFGSECKLTENQIHEIAYGLELSELPFLWSLRKPSYSTDPLPPDFHRRIAEKGLVHIGWAPQTEILAH
PSVGGSLFHSGWGSVIETLQFGHRLIVSPLIIDQPLNARLLVEKGLAREVERGGDGSFGRDAIARALREA
MVMEGGERVREAMAIFGDRKLQENYVERFAEYLKIGVAKEN
>GWHPAAAL008406
MSVHVLIFPYPAQGHLLPLLDLTHQLALHGLTITILVTPKNRPTLDPLLSTHPSIGTLVLPFPHHPAIPS
GVENVKDLGNSGNVLVINALSKLRDPIIQWFKAHPNPPVAIVSDFFLGWTNDLAGQLGIPRVAFYSSGAF
LTSILFHFYRELRSAAPVLTAVKFTDLPRSPSFTEAHLPTIVRHYRESNPEWELLKEGVIANSWSWGSIL
NTFDDLEGEFLEYLKKKMGHNRVYGVGPLNLGRVNTDPDADPSNTVLGWLDGCPDGSVIYVCFGSQKFLK
KAQIEALALGLERSKARFIWVVKPITAQQEADGYGSILDGFEERVSGRGMVIKGWAPQVKILSHRVVYGF
LSHCGWNSVLEAIAAGVMILGWPMEADQFVNARLLVDYMGAAIRVCEGADSVPDSDELARTIAESVDGNK
QEKGRARELRDKAWEAVGPCGSSTKDLVGFVKDLAQLDENGV
>GWHPAAAL008407
MGNPHVLVVPYPAQGHVIPLMELAHSLAQHEFRVTFVNSDFTQDRVTGSSPEKNGFGEDSIRLVSISDGL
EPSEDRNELGNLTESIFRVMPAKLEELIMEINGSDGDEVACVIADISMAWVMEVAAKMGIRRAAFCPMSA
AMLGLVYNVPKLMEDGIINDDGTPTKNEVIKLTPDMPAMNTANFPWACIGDLATQKKIFDVTLTGRPALD
LADRIICNSSSGLEPAVFDQYPQFLPVGPLLRSSGGNFWQEDSTCLDWLDQQPPKSVIYVAFGSFTVFDP
TQFRELAKGLELTDRPFLWVVRRDSAVKYPEGFEKIIGTGRRGKIVEWAPQKKVLSHPSVGCFLSHCGWN
STVEAVSNGVPLLCWPYFADQSFNQSYICDVWKVGLGFGKDENGLIIFEEIKKKVDEIFGDENYRERALD
LKQKVMDGVKEGGCSNRNFSKIMGWITQINNGLSS
>GWHPAAAL008878
MASPSPHFVLLPFLAQGHLIPMIDISRLLAKRGVVVTIITTPLNAKRFERIITREIDSGLQIRVVQLHFP
CVEAGLPQGCENFDHLTTMASAGQFLEATRMLQKPVEDLFEGLSPPPTCLLSDMCYPWTTLVAEKFSVPK
LVFHGMCCFSLFCNHVLYTSRVFDTVASETEPFAVPGMPDHIELTKTQVPTLVNMNTSSGVKDVRKEIKE
GELSAYGVVVNTFEEMEEKYVKEYAKAMGKKVWCIGPVSLCNKNNLDKADRGNKASIDEHHCLKWLDSWE
QGSVVYVCLGSLTRLATSQMIELGLGLEASNRPFVWVIRYEIVEFEKWILEEGYGERIKGRGLLIHGWAP
QVLILSHRAIGGFLTHCGWNSTMEGICAGVPMLTWPIFAEQFVNEKLVVHVLKTAVSVGVKIQIYPGNEE
EVGILLRREDVKMGIDKVMEEKEEGEERRRRARKFGEMAKWAIEEGGSSHHNLTMLIQDVMEKSNQLRKD
G
>GWHPAAAL008879
MASPSPHFVVLPFLAQGHLIPMIDISRLLAKRGVVVTIITTPLNAKRFEGIIARAIDSGLQIRVVQLHFP
CVEAGLPQGCENFDHLISMASAGQFFEATRMLQKPVEDLFEGLNPPPACLLSDMCYPWTTLVAEKFSVPK
LVFHGMCCFSLFCTHVLQTSRVFDTVSSEIEPFAVPGMPDFVELTKNQVPAFVNMNIPSGQKDVRKEIKE
GELSAYGVVVNTFEAMEEKYVKEYARAMGKKVWCIGPVSLCNNNNLDKADRGNKASIDEHHCLKWLDHWE
QGSVIYVCLGSLTRLATSQMIELGLGLEASNRPFVWVIRYEIVEFEKWILVEGFGERIKERGLLIRGWAP
QVLILSHRAIGGFLTHCGWNSTMEGICAGVPMLAWPMFAEQFVNEKLVVHVLKTAVSIGAKIQISIGKEE

EIGILVRREDVKMGVDKVMEENEEGEERKRRARKFGEMAKWAIEEGGSSHNSLTMLIQDVVEKSNQLRKH
G
>GWHPAAAL009069
MAYPPPSPALHIALFPFMSKGHTIPIIHLTRLLLNRGHKTTVFTTPANRPFISDSLTGTDASVIVLPFPE
NIPGIPSGTESTDKLPSMSLFVQFANATKLIQPHFEQAIKSLPNLNCMISDGFLSWTLQSASKFGIPRLV
FYGMNNYSMALNYDVTVNRLLSIPKSDNEPFVVPNFPWIKLTKNDFEEPFNEVEPKGPYVEFTIEQSLAT
FNSYGLIVNSFFELEKPFVEYWNRASKPKAWCIGPLCFAEPEMIIERKRKPKPKWIDWLDQKRDQGKSVL
YVAFGSQAEISQKQLHEIAMGLEKSEVNFLWVMRKKDTAIEGVPERGMVVRDWVDQREVLAHEGVRGFLT
HCGWNSVLESIWARVPILAWPMIAEQHLNARMVVEEIKIGLRVETIDGSVRGFVKRESLEKMVRELMEGD
MGKEVRKKVEEVGVAAGKAMAEGGSSWDALNQFIDEIKAQGSNPIN
>GWHPAAAL009324
MDRSASSKLHIAMFPWFAVGHLTPFLHLSNEIAQRGHKISYLLPRKAHIQLQSQNQHPDLITFYPLAVPP
VAGLPSGTETASDIPISQTGLLATAMDHMRGEVEEIFRSLRPDFVFYDLAHWITELGSSIGFKTVCYNVV
SAASLAIALVPARVVPKDRKVTVDELAVPPKGYPSSSVVLRPHEARSLLFISSEFGAGGVTFYERVIAAM
KNCDAIGIRTCRETEGSLCDYMGTQYNKPVLLTGPILPEPSKAQLDDRWSDWLNKFPPKSVILCTFGSQI
VLEKGQFHELVLGLESTALPFLAAVRTSAGEEALPEGFENRVGERGVVYGGWVQQPLILNHPSVGCFVSH
CGFGSMWESLMSDCQIVVVPHLGDQILNARLLAEEMKVAVEVERDEKGWFSRERLGGAIKTVMERESEAG
EVVRKNHAKWKEIMVKPGFMSNYVENFIEKLHEL
>GWHPAAAL009436
MGSIPASDKPHAVCIPYPAQGHINPMLKLAKILHSRGFHITFVNTHFNRNRLLHSRGPSALDGLPDFRFD
SIPDGLPPSDADATQDIPSLCESTPKHCLEPFCDLIARTNEDAGVPPVSCIVSDGVMSFTLKAAERFGLP
EVLFWTTSACGLLGYTQYHKLVERGYTPLKDVSYVTNGYMETTMDWIAGMKDIRLRDLPSFIRTTDPKDT
MLNFLISECAAIPTASALILNTFDALERESVDALSAIHPRIYTVGPLNLMLNQINDDRIKSIGSNLWKED
PGCIEWLDSKEPGSVVYVNFGSITVVTAGQLTEFAWGLANSGRPFLWIVRPDIVAGDKAMIPPEFLAETE
GRGRLSSWCPQEQVLRHPAVGGFLTHSGWNSTVESLCSGVPVICWPFFAEQQTNCRYSCREWGVGMEIDN
N
>GWHPAAAL010110
MGEKQPELQKQQRQGLRLVLFPLPFKGHINPMLQLAAILHANGFPISILHTTFNSPTPSDHPHFDFHPIS
VDGLSETDSPTPDLVDLLELLNQRCAAPFRDCLEKLTSEEPVACLISDAILHCTATVADSLRIPRIVLRT
GGVASFLAFASFPLLLDKGYLPIQESRLEEAVPELPPLRIKDLPVINTGKPEALYHLLAEMIKTAKNSSG
LIWNTFDELEQPELSKLRREFPIPIFPIGPFQKHLLLTSTHEDRSSSISWLQNQPPSSVIYVSFGTIASM
EESQFTEIAHGLADSLHPFLWVVRPGLIRGSEWLKNLPDGFLERLEGRGKIVKWAPQAEVLAHPAVGAFW
THSGWNSTVESISEGVPMICMPCFTDQLVNARYVSHVWGVGLTLEGGIERGKIERVMRRVMGEEGKEMRK
RILCMKERVNVCLRKGGSSHQSLHNLVSYISSL
>GWHPAAAL010292
MEDVPAGKPHAILVAYPLQGHVIPAVHLALNLASRGFTITFVNTQSIHHQITHRPSGKAAAAAVDDIFAA
IRESGLDIRYTTVPDGLPAGFDRSLNHDQFMAALLHVFSAHVEEAVDEIVRAGPPVSCLIADTFFVWPGK
LAKKLGVVYASFWTEPALVFTLYYHLDLLRIHGHFGCIELRDDIIDYIPGVKSIEPKDLTSYLQQSDSST
VCHQIICNAFNDVKTADFVLCNTVQELEPQTITALQSKTPFFAIGPIFPSGGFTTNNVSTSLWSESDCTH
WLDAKPDGSILYVSFGSYAHVEKNDLVEIANGLLLSKIEFLWVLRPDIVNSDDSDPLPEVLKEEIGNRGM
IIPWCNQRQVLAHRAIGGFLTHCGWNSVLESVWCQIPLLCLPLLTDQFTNRKLVVDDWKIGTNLCDKRPI
TRGEVCEKIKEVMSEKSGNEFREKIKEVKNKLENALGANGSSTKNMDQFIKDLKATIQKKRGGTS
>GWHPAAAL011043
MENNIEKPPHAIMFPYPLQGHLIPFVHLAIKLASNGFVVTFVNTQSIHHQISQSRTPDAGADIFAGARNS

GLDIRYKTVSDGLPVGFDRSLHHDQFMESLLHIFSAHVDELVGEMVASGDPPVSCLVADTFYVWPSTVAK
KYNLVNGSFWTEPALVLTLYYHLDLLRQNGHFGTTDSCKDNIDYIPGVPSMKPTDLMSYLQIDTSTVVHR
IIYKAFNDVKSADFILCNTIQELEPETISALHKKQPTYAIGPIFPVAFTNSSVATSLWAESDCSQWLNTK
PHGSVLYISFGSYAHTTKHDIMEIAYGLLLSGVGFVWVLRPDIVSSDDVDFLPVGFDENIKGQGLIVPWC
RQIEVISHPAIGGFLTHCGWNSILESVWYEIPLICFPLLTDQFTNRKLVVDDWKIGINLRDEKSITREEV
SKKINYLINEKTSNQLRKEIRKVRVLLEDALANDGSSQNNFNQFIRNFKDKLNVKKK
>GWHPAAAL011200
MTIGGAGDHILVFPYPAQGHMIPLLDLTHQLAIRGLTITVLVTPKNLPLLDPLLSEHESVATLVLPFPVH
PSIPAGVENVKDLPVGGFRAMMVALGELHDPLLRWFQNQPSPPKAIWSDMFLGWTHHLARRLGIRRFVFS
PSGAMALSVIYSLWREFPRRRDSNDYNEKFSFPKIPNSPIYPWWQLSPIFRSYVAGDPDSEFIKDGFLAN
IASWGLVINSVSELERVYLEYLKGELGHDRVWTVGPLLPPDGDLSRTVERGGSSSVLTNQISSWLDKCND
RTVVYVCFGSQAVLSNDQMEHLASGLEKSGSKFVWSVKGATTGQEKGSCGQVPLGFEESVGARGLVIRGW
APQLLILNHRAVGAFLTHCGWNSLLEGLIAGVPMLAWPMGADQFANASLLVDELRVAIRLCEGAQVVPSS
NELARLVTEATGGDWPERGRAEELRKVALGAIKDGGSSFNNLNDLVRHISEIV
>GWHPAAAL012110
MEESKKEEHIVMLPFMAQGHLIPFLELATQIHQSTAFTVTIATTPLNTQYLKSTAAKQNPSPESPPAPAI
HFVSLPFNSSDHGLPPNTENTESLPLTQIISLFHASSSLKPHFQDLIAGIADKEGKPPICVISDVFMGWA
NEVALSFGTVNVSFTTGGAYGTAAYVSIWQNLPHRSRDSNDEFSLPGFPESRRFHISQLHQFLRAADGTD
EWSLFFQPQISLSMGSFGWLCNTVEDIEPLGLEILRRYTKLPVWCIGPLLPRKMLQRSSSSNSGVIGPRS
GKLPGVSPEKCIEWLEFHPPASVLYISFGSQNTISATQMMELAHGLESSGKPFIWVVRPPVGSDPNSQFK
SSEYLPEGFERRAAENNRGLLVHKWAPQLEILSHKSTGAFLSHCGWNSTMESLSQGVPIIGWPLAAEQSY
NAKMLEEEMGVCVELTRGVQSTIERDKVKKVIELVMGEKGKGEEMRKKAAAIGEVIRASVMEEGEGRKKG
SSVQAMDDFISTLISNREQSFPHPL
>GWHPAAAL012139
MEERRENIVMFPFMAQGHMIPFLALALQLEKKGYGITFVNTPLNVLKLRQCLPPDSSIRILEIPFNSSDH
GLPPNSENTDVLPYTLIVNLLHASLSLESSFRKLLSDLIHEQGGVRPLCVIADIFFGWSAGVARELGVFH
AIFSGAGGFGLACYYSAWLSLPHRTTDSIEFTLPDFPEASKFHVTQLPASMLAADGTDLWSIFQRKTLPA
WTNSDGVLFNTVEGFDKLGLAYFSRKIGKPIWAIGPTLLSVHHRARVGGREAAITPEQCAEWLDSKPPNS
VIYVSFGSQNTISASQMMQLAKALDKSEKNFIWVVRPPLGFDINSEFKPEEWLPEGFTDRIKDQNRGLIV
IKWAPQVEILSHKSIGAFISHCGWNSVLEALSNGVPLIGWGMAADQFFNVKMLEEAVGVCVEVARGTIFE
VRHEDIMEKIEVVMGESEKGKRIRKRAGELKEIIKDATRDEEGFKGSSVKAMDEFLSAAAMSMKEKKTLL
VETQ
>GWHPAAAL013261
MASEFHHAHFVLIPLMSPGHIIPMVDMAKLLARRGVTVTLIMTQHSATRFASVVDRAIKSGLPIRLLEFR
FPSKEAGLPEGCDSVDDIPSYDLLLNFFNAIDMSQNPLEQLIEHLEPNPSCIVCDKHIAWTAETAKKFNI
PRIIFDGMSCFSQLCVHNLHVSKVHERFQGSEPFVLPGLPDEIVITRAQLPGTFNPGSMKNLDEIRKRVR
ETEKEAYGVVVNSFEELEQRYVNEYKKVREDRVWCIGPLSLCSNETSDMANRGNTASIDANHCFKWLDSR
QPRSVIYACLGSLSRLTPPQFIELALGLESTNQPFILVIKAGDKVKEIETWISENGFEERTRERGLLIRG
WAPQVLILSHAAVGAFLTHCGWNSTLEAVCAGVPLITWPLFSEQFLNEKFAVQVLKIGESVGARVAVGLG
EEEKSGVKVEREEVEAAARRLMEEGEEGGGRRRRARELAEMAKRAVEEGGSSHLNMTVMIQDIVSIKREE
T
>GWHPAAAL013365
MSTPTDNGEKLHIVMFPWLDFDHMIPFLELAKLIAKKGHKLTYVSTPQNIRWLPNPPLNWVLLPLPIVES
VPDNAKATVDVPSGKVSCLVDAFDRLETELTRFLEASMPDWIIYDFAPPWLPTVVGNLGISRAFFNVRNA

SFVYSMIKGCDLPNDIIGPLGVHRLWSRCDVFATRDCTEFEPHCLNTLRKVTQKPVIPVGLMPSLVHDSE
DDKTYTWLAINDWLQKQNKESVAYVALGIEVFKNQESLSEMAIGLELSSLPFLWALRRPHGSTELGSVKL
SLGFEERTKARGIVCRTWVPQAKVLAHGLVGCFMTGCGWSSVIEGLQFGRSLLMMPFTEEQRLNTRVCVK
KNMGIEIAQVGSLTRNSVADTLKLVMSNDNNEGKLIREKVKMMNWIFGDKARHDLYMDNFVDYLQNYRSL
NKVP
>GWHPAAAL013653
MTSPSSSPPSLPHVFMVSFPGQGHVNPLLRLGKYLASKGLLITLCTPEIFGKQIRKSNPIIAGENPIKIG
EGFIRFEFIQDDLEEDDPIFKDFDLYFPDLEMKGKAALQRMIEENKVMGRPVSCLINNPFIPWVSDVAEA
MAIPNAMLWVQSCACFAAYYHYGKGSVPFPSERELELDVQLPAMPLLKHDEIPSFLHPTTPYTVFRRAIM
GQYRNLSYPFCILMDTFMELEEELIENMSEICPVKPVGPLFRNAGGYGSEIRGDMMRADEECLKWLDSMP
ECSVVYVSFGSIVSLKQTQLDEFAAGILNSGVSFLWVVKPVPEKPVKLPEVFLEKAGDRGKIVEWSPQEQ
VLAHPAVACFVTHCGWNSSIEAVANGVPVVAFPQWGDQVTNAKYLVEVFGVGLRLSRGEAENRTVTAKEV
ERCLLEATSGGKAAELKEKALKWKKKAEEAVAEGGSSNRNMKDFVEEVKRRSI
>GWHPAAAL013654
MVSPFPSSLPHVFMVSFPGQGHVNPLLRLGKYLASKGLLITLCTPEIFGKQIRKANPIIAGENPIKIGEG
FIRFEFIQDGLEEGDPIFKDPDLYFPDLEMKGKAALHRMIEENKEMGRPVSCLINNSFIPWVSDVAEAMA
IPNAVLWVQSCACFAAYYHYGKGSVPFPSERELELDVQLPAMPLLKHDEIPSFLHPTTPYTVFRRAIMGQ
YRNLSYPFCILMDTFMELEEELIENMSEICPVKPVGPLFRNAGGYGSEIRGDMMRADEECLKWLDLMPEC
SVVYVSFGSIVSLKQTQLDEFAAGILNSGVSFLWVVKPVPEKPVKLPEVFLEKAGDRGKIVEWSPQEQVL
AHPAVACFVTHCGWNSSIEAVTNGVPVVAFPHWGDQVTNAKYLVEVFGVGLRLSRGEAENRTVTAEEVER
CLREATSGGKAAELKEKALKWKKKAEEAVAEGGSSDRNMKDFVEEIKRRSI
>GWHPAAAL013655
MVSPFPSSLPHVFMVSFPGQGHVNPLLRLGKYLASKGLLITLCTPEIFGKQIRKANPIIAGENPIKIGEG
FIRFEFIQDGLEEGDPIFKDPDLYFPDLEMKGKAALHRMIEENKEMGRPVSCLINNSFIPWVSDVAEAMA
IPNAVLWVQSCACFAAYYHYGKGSVPFPSERELELDVQLPAMPLLKHDEIPSFLHPTTPYTVFRRAIMGQ
YRNLSYPFCILMDTFMELEEELIENMSEICPVKPVGPLFRNAGGYGSEIRGDMMRADEECLKWLDLMPEC
SVVYVSFGSIVSLKQTQLDEFAAGILNSGVSFLWVVKPVPEKPVKLPEVFLEKAGDRGKIVEWSPQEQVL
AHPAVACFVTHCGWNSSIEAVTNGVPVVAFPHWGDQVTNAKYLVEVFGVGLRLSRGEAENRTVTAEEVER
CLREATSGGKAAELKEKALKWKKKAEEAVAEGGSSDRNMKDFVEEIKRRSI
>GWHPAAAL013658
MVSPFPSSLPHVFMVSFPGQGHVNPLLRLGKYLASKGLLITLCTPEIFGKQIRKANPIIAGENPIKIGEG
FIRFEFIQDGLEEGDPIFKDPDLYFPDLEMKGKAALHRMIEENKEMGRPVSCLINNSFIPWVSDVAEAMA
IPNAVLWVQSCACFAAYYHYGKGSVPFPSERELELDVQLPAMPLLKHDEIPSFLHPTTPYTVFRRAIMGQ
YRNLSYPFCILMDTFMELEEELIENMSEICPVKPVGPLFRNAGGYGSEIRGDMMRADEECLKWLDSMPEC
SVVYVSFGSIVSLKQTQLDEFAAGILNSGVSFLWVVKPVPEKPVKLPEVFLEKAGDRGKIVEWSPQEQVL
AHPAVACFVTHCGWNSSIEAVTNGVPVVAFPHWGDQVTNAKYLVEVFGVGLRLSRGEAENRTVTAEEVER
CLREATSGGKAAELKEKALKWKKKAEEAVAEGGSSDRNMKDFVEEIKRRSI
>GWHPAAAL014206
MAKNTHVVMLPWSAFGHIIPFFQLSIALTKAGIHVSFVSTPKNIRRLPQIPSDSVPFIDLVELPLPEIDR
SLLPADAEATVDIPTENIQYLKIAYDLLKKPFSRFVADRLPDWIVIDMMPHWAVDVAKEFDVPVIYFSVF
GAAAAVFWGPPEYIAGEGQKMKRSTPESLTSTPEWVDFPSAVAYGQNEAVSFHAGAYGENASGISDAGRV
ARILHSCRALAIRSCPEFEHDYLNLLEKIHQKPVIPVGLLPPEQKSEARNSGDKFKIFKWLDQQKPKSVI
FVGFGSECKLTENQIHEIDYGLELSELPFLWSLRKPSYSTDELDPLPPDFHRRIAEKGLVHVGWAPQTEI
LAHPSVGGSLFHSGWGSVIETLQFGHRLVVLPLIIDQPLNARLLVEKGLAIEVERGGDGSFGGDAIAKAL

REAMVMEGGERVREAMAIFGDRKLQENYVERFAEYLKNGVAKKNR
>GWHPAAAL014233
MEERRENIVMFPFMAQGHMIPFLALALQLEKKGYGITFVNTPLNVLKLRQCLPPDSSIRILEIPFNSSDH
GLPPNSENTDVLPYTLIVNLLHASLSLESSFRKLLSDLIHEQGGVRPLCVIADIFFGWSAGVARELGVFH
AIFSGAGGFGLACYYSAWLSLPHRTTDSIEFTLPDFPEASKFHVTQLPASMLAADGTDLWSIFQRKTLPA
WTNSDGVLFNTVEGFDKLGLAYFSRKIGKPIWAIGPTLLSVHHRARVGGREAAITPEQCAEWLDSKPPNS
VIYVSFGSQNTISASQMMQLAKALDKSEKNFIWVVRPPLGFDINSEFKPEEWLPEGFTDRIKDQNRGLIV
IKWAPQVEILSHKSIGAFISHCGWNSVLEALSNGVPLIGWGMAADQFFNVKMLEEAVGVCVEVARGTIFE
VRHEDIMEKIEVVMGESEKGKRIRKRAGELKEIIKDATRDEEGFKGSSVKAMDEFLSAAAMSMKEKKTLL
VETQ
>GWHPAAAL014996
MEITDGETGSSQNRVLVIPFPSQGHINPMLQFSKRLAAKDRRRSLGITLVTTSTTKFSALSQPPTSVSVE
YLPDDSDDDQPDDVGAFFRRFEKTVTEGLPGLVERLKNSGSPARVVVYDSIIPWVLDIAHPLGLKGACFF
TQTCAVCAIYYHVHRGTLTVPSEEEEEDHRSSVTLPGMPLMEIKDLPSFVYDIDQYPYALRLLLAQFSNT
GEAEWIFFNTFDKLEHEVIDNWAAREWPRMKSIGPTIPLAYLEEEDNTLQNDIDYGLNLLDSNFEACMNW
LDAKETASVAYVSFGSVANLNNEQMEELAWGLISSGTHFLWVVRASEESKLPPNFTDQALENGLIVNWCP
QLQVLAHRAVGCFVTHCGWNSTLEALSLGVPLVAMPHWSDQTTNAKYVSDVWKVGVRARGVEKGAGVVGR
AEIERCVKEVMEGESGDEVRRNAVRWKELAREAVAAGGSSDNNIQEFVSQLVKL
>GWHPAAAL015368
MTNTNRVELVFIPSPGMGHLVSAVEMAKLLVDRDQRLSITLLIMKAPFKTKANYSPAQSLSTAAASRIRF
VDLPGDELESDAKFSPHTFLSGFISGQKTLVRDAVAQISSSPEAPRLAGFFIDMFCTSMIDVADEFRVPT
YVFFTSGAAFLGLVFHLQTMRDDHNHDVTELKDSDADLLVPCFVKSVPTKVLPSVVLDKEGGSEMFHDLV
RRIRETKGILINTFSELESHAIHSINTPPVYPVGPILNVAGDNDNESTSAAAILKWLDDQPPSSVVFLCF
GSMGCFGVDQVNEIAHALERSGHRFLWSLRRPPPEGKLEFPGDYTNLEEVLPERFLERTAEIGRVIGWAP
QAAVLAHRAVGGFVSHCGWNSTLESVWYGVPMAAWPQYAEQQLNAFLMVEELEMAVEIKMDYRNGFYSKS
GVALRSEEIEKGIRRLMECGENGMRKKVEEMREKSRVAVREGGSSYLSIGRLIEDVMKIVEQ
>GWHPAAAL015516
MAYHHPRQTQPQVAVVVVPFPAQGHLNQLLQLSRLISFYHIPVHYVGSATHNRQAKLRNHGWDPLSATNI
HFHEFSTPHFLSPPPDPHAAVKFPSHLQPSFEASSHLRRPVAALMRELSQKFRRIIIVNDSLMGSVVQDF
VSLQNADAYTFHSVSAFAMALFFRESTGKHFSVHAELLNGVPSLDGCFTADFLNFLKTQHYYTQFSSGRI
YNACRLIEAPFLDILSQDHDTAINKNKKHWALGPFNPISITHNKRPNSRHKCLEWLDKHDQNSVILVSFG
TTISLSEEQIKELATGLEQSGQKFIWVLRDADKGDVFNEEDEVRRAELPKGFEDRVEGRGLVVREWAPQL
EILGHPSTGAFMSHCGWNSCMESITMGVPMAAWPVHSDQPRNAVLVTKFLKIGLPVKDWARRDEVVIALT
VENAVKKLMAAEEGREMRKRAEELGGAVRRSAAEGGASCKEMDAFIAHITRI
>GWHPAAAL015754
MSGDRKLHIVMFPWLAFGHMIPYLELAKLIAEKGHKISFLSTSRNIDRLPKLPPHLTPVIDFVKLQLPHV
EGLPENAEATSDVPYNKVKYLKVAYDLLQEPIARFLRISSPDWVLFDFVSYWLGPIASELRIPSAFFSIF
IPAFLGIFGPVPVLKGTDEARTKPEDFTVSPKWVPFKSTVAFRLFEIQRIFDSVTGDEMNVPDTYRAGCT
IESCDIIAIRSCHEFVPEWIKVLEEIHQKPVIPVGQLPTTAYDGGEDDKDGQWEKMKEWLDTKEKKSVVY
VAFGSEAKPSQAELTEIALGLELSRLPFFWVLRNRRGNADTEPIQLPEGFEERTRGRGWVCTKWAPQLKI
LSHDSVGGFLTHSGWSSVVEAVQFERALILLTFLADQGLNARVLEEQKMGYSIPRDDRDGSFTRDSVANS
VRLVMVEEGGKIYREKMKEMLGVFGDRDRQDGYVENFLRYLQNYKIPTSEDPNKKE
>GWHPAAAL015755
MELIIPSNGRKLHIAMFPWLAFGHLIPFLELAKLIAQKGHQISFISTPQNVHRLPKLPPNLTPLINFVQI

PLPAVENLPDGAEATIDVPYDKVKYLKIAYDGLQEPITKFLHYSSPDWIIFDFASYWVGPVASELEVSSA
FFSIFTAAFMGFCGPAEILKGTVEGRTKPEDFIVKPNWVPFESRVAFRLFEILRVFDGVTGDEDNVSDGL
RMGCTIEGCDLIIIRSCYEFESDWLRVIEEIHRKPLIPVGQLPTTVYDGGDVDKDGAWREIKDWFDQQEK
GSVVYVAFGSEAKPSQTELTEIALGLEVSGLPFFWVLRKQRGSADIELTQLPEGFEGRTRGRGIVCTTWA
PQLKILNHVSVGGFLTHSGWSSVVEAISLEKALILLTFLADQGPNARLLEEKKMGYSVPRDERDGSFSRD
SVADALRLVMASEEGKIYRDKVKEMKWAFGDRDLQDRYVHNLLGYLQTHNRKKGR
>GWHPAAAL016884
MAASTFHIAMYPWFALGHLTAFLHLSNKLAKKGHKISFLIPTKTQPKLNPFNLYPNLITFIPITIPHVDG
LPPGAETTSDIPYLSGTHLMTAMDQTQAHIELLLRDLKVDIVIFDFAYWIPAVAKWLGIKSIHYCTISPA
TVAYTMVPARNLKGNKVTETDLMQPPEGYPVSSIKLHAHEAPAFVARRSMRFGGDKQFYERQFLSFSQCD
ALGFRACREIEGQFCDYIESQFRKSVLLSGPVIPEPPTSPLEEKWVKWLGKFEAGTVVYCTFGSECTLKK
DQFQELVLGLELAGKPFLAALKPPAGVESTEAALPENFKERVQGRGIVHGGWVQQQLILEHPSVGCFITH
CGSGSVSEALVNKCQMVLLPHVGDQIINARMMSRDLKVGVEVEKGEEDGVFTRESVCRAVRAAMEEGSEV
GKEIRANHAKLREFLLHKDLESSYIENFLMKLQELLQ
>GWHPAAAL016885
MAASSFHIAMYPWFALGHLTPFLHLSNKLAKKGHRISFLIPTKTQHKLNPFNLHPDLITFIPITVPHVDG
LPPGAETTSDTPPQSLTHLMTAMDQTQHHIEHLLRDLKPDVVFFDFTYWIPEVAKRLGIKSIHYCIISPA
TIGYTMVPTRNLKRKNEVMEIDMMRPPEGYPASSIELHAH
EARAFVARRRRVAKSRANFVTTLKANSEXTSPLEEKWVKWLGKFEAGTVVYCAFGSECTLKKDQFQELVL
GLELAGKPFLAALKPPAGVESTEAALPENFKERVQGRGVVHGGWVQQQLILEHPSVGCFITHCGSGSLSE
ALVNKCQLVLLPHVGDQIINARLMSRDLKVGVEVEKGEED
GVFTKESVCRAVRAAMEEGSEVGKEIRANHAKLREFLLHKDLESSYIDNFLMKLQELPQ
>GWHPAAAL017403
MEREIDEMGSQKPHVLVVPFPAQGHFSPALQLSKRLASKGIRVTFVTTISSAESSGSIRMEHISDGYEEG
EKPESIEAFTDRIKTVFSQNLGDLIEKQKRVGYPVKLLIYDSVIPWALEIAHSLGVYGAPFFTQTCAVCA
IYYHAQKGNLEVPLEGTKFSLPSLPPLERNDLPSFLSDMNSYQFFLKMVLAQFSNFQNADWILFNTFDKL
EDEIVKWMASQWPIRTVGPTIPSMYLDKRLKEDKEYGLSLFTPNIESSMKWLDSKEPGSVVYISFGSLAN
LDEGQMKELSWGLVKSNYYYLWVVRDSEESKLPNNFMAETSEKGLVVSWCPQLEVLAHQAVGCFVTHCGW
NSMLEALCLGVPMVAMPQWTDQTTNAKFVADVWRAGVRVEANDEGVVTREEIEKRIREVMEGETREELRK
NASNWKNLAREAVDEEAVIFPANPSQKNNPMTQFSKCLATTGHRLAVTLVNTTATKFSYLSQPPSSISIE
RMYLMMIN
>GWHPAAAL017405
MEITDRETGSSQNRVLVIPLPVQGHINPMLQFSKRLAAKDRHCRLGITLVTTTATKFFALSQPPNTISIE
SLPDDSDDDQLVDTVALIRRFVKTVTEGLPRLVERLKNSGSPVRVVVYDSFIPWVLDIAHQLGLKGACFF
TQTCAVSAVYYHVHQGTLTVPSEEEQDHRSSVTLPGMPLMEINDLPSFVYVVYDQYPAALRFLLAQFSNA
GKADWILFNTFDKLEHEVIENWAAREWPRMKSIGPTIPSAYLEEEDKTLQNDKDYGLNLLDPNIEACMNW
LDAKETASVAYVSFSSVADLKKEQMEELAWGLIGSGTHFLWVVRASEESKLPPNFTDQALENGLIVNWCP
QLRVLAHRAVGCFVTHCGWNSTLEALSLGVPLVAMPQWSDQTTNAKYVSDVWKVGVRARRVEKGEGVVGR
EEIERCVKEVMEGESGDEVRRNAVRWKELAREAVAAGGSSDNNIQEFVSQLVKL
>GWHPAAAL017961
METRRGPKEVVLVPYPAQGHMSPMLQLGTLLHSLGFSITVAYASLNSPDPSTHPEFDFLPISEDLSDRDT
SSGAVFSLITSLNADCAEPLRECLVRVTEDRGAVACVVYDSLIYFPEEVAHRMKIPSMVLQTCSAAYVLA
CRLFPELKEEGYLPAESEDPSMEDLVPKLHPLRFKDLPITKFDTTALQILMDLNDVGTSSAVIWNTTDYL
EHPSLCRLRQHYQVPIFPLGPLQAMAPTSSTSFLKEENGCLNWLENQAPKSVIYVSFGSNATMEPKELSE

VACGLANSDQPFLWVVRPGSVKGSKWVEHLPEGFIEAVGERGCIVKWAPQKKVLAHGAVGGFWSHCGWNS
SLEGLSEGLPMICRPCFGDQRANARYLAHVWRVGLELELELERGAIERAVRRLMTGKEGEELKRRAMDMK
QNIEESVQKDGSSYNSLIELADFISDFHKTRQCV
>GWHPAAAL018171
MGKQLHVVFFPFMAHGHMIPTLDMAKLFSSRGVKTTIITTPLNAPVFSKSIQRSNQLGLDISLKIFDFPA
VAAGLPEGCESADQITSEDMLPKFFVATALLREPLERLLSDHRPDCLIAGMFFPWTTESAAKFGIPRLVF
HGTSFFALCAGESLRTHKPFRQVTSDSEPFVLPELPHEIKLTRTEVSPFELHDTETAISGVLKQVSESEK
TCYGIIANTFYALEPDYADHYRNAMGRTAWHVGPLSLCNRGIEDKAERGKKSSIDEHECLQWLDSKKPKS
VLYVCFGSMAKFADSQLYEIAAGLESSGQQFIWVVRKSKDEEEQKEETWLPHGFEERTQGKGLIVRGWAP
QMLILDHEAIGGFVTHCGWNSTLEGACAGLPMVTWPAFAEQFYNEKLVTDVLKIGVSVGAKEWKTGPREG
VKREAIEKAVRSVMEGEKAEEMRRRAAALKEAAMEAVEEGGSSYSDLTSLIEELTSSQKINGT
>GWHPAAAL018716
MEMEMEKREYRAHVLAIPFPAPGHVNPLLQFCKRLEFKGLKASLAITKFQSKSMQSRLGSIQIHTISDGY
DEIGRHNAESVGVWISTFKEVGSKSVANLIQKFRSLGHPIDCIVYDSFLPWVLDVAKENGVAGASFMTHK
CAVNHINYHVYHKKIPYPFSSPTYSIPGLPSLDLEYMPGFMHVHPDYYDLVLSQFSTVDRADYVFVNTFY
KLEAEVLDEMSKTLPVKPIGPTVPSFYLDNRVEDDKNYGLNLQQLDSSVCLNWLSSKPARSVVYVSFGSV
ATASPAANEMEEIAWGLKNSNCYFLWVVNANEEPNLPQNFKEEMSEKGLIVAWSPQLEVLSHHSVGCFLT
HCGWNSTIEAICLGVPMVAVPKWADQTTNSKFIQDVWKVGVKVMPSENGIARREEIERCINV
>GWHPAAAL019177
MDHHRRRHHFLLISLPAQGHFNPTLQLAKVLARAGVTVTFATTVYGLNQLKVNSFTVDGIAYASFSDGHD
EDRSKASSDFAGYLEDQRRVGSQNLIKLLENLANDGRPVTFIVYTVLLPWVAQVAREMHVPSAFFAIQCA
ATLAVYHRFFNSSDGLLGGENEINESLSVELPGLPPLSSGEIPSFLMPADQYHSSVSPSFREHILTLEED
PNPTVLINTFDALEEDSIRAVRNMKIIAVGPLVPSAFSDGIDGSDKSFGCDLFRNSDDHHLRWLDSKSES
SVIYVSFGSLAILGKPQKEEILNGLIDTERPFLWVIRDTAGEEEEMAVPENGLVVPWCTQVEVLAHSSIG
CFVTHCGWNSTIESLVAGVPVVAMPQFSDQLTNAKLVEEVWGNGVRARKSEEKGVVEREEIRRCVEVVVG
GGERGEEIRGSARKWRGLAMAAVREGGSSSKNLRDFLDSLG
>GWHPAAAL019178
MDHHRRRRLHHFLLISLPAQGHINPTLQFAKVLARAGVTVTFATTVYGLSQLKANSSTVDGIAYTSFSDG
YDEGGAKANIIFPDYMEDLKRLGSQNLIKLLKNLAGEGRPVTFLVYTVLCPWVAHVAREMHVPSAFFAIQ
CAATLAVYHRFFNSSDGLLGGENEINESLSVDLPGLPPLSSGEIPSFLMPANQYHSSISPSIREHILTLE
EDPNPTVLINTFDALEEDSIRAVRNMKIIAIGPLVPSAFSDGIDSSDKSFGCDLFRNSGDHYLRWLDSKS
ESSVIYVSFGSLAVLGKPQKQEILNGLIDTGRPFLWVIRDTAGEEEEEEETAVPENGLVVPWCTQVEVLA
HSSIGCFVTHCGWNSTMESLVAGVPVVAMPQFSDQLTNAKLVEEVWGNGVRARKSEEKGVVEREEIRRCV
EVVVGGGERGEEIRGSARKWRGLAMAAVREGGSSSKNLREFLDSLG
>GWHPAAAL020336
MFTSNRVELVFIPSPGMGHLVSAVEMAKLLVDRDQRLSITILIMKVPSKTKANYSPSQSLSTAAASRIRF
VDLPESESTPQFSPQSFISGFIPSQKTRVRDAVAKIAAAPGAHRLAGFFIDMFCTSMTDVADEFGVPTYV
FFTSGAGLLGLMFHLQTMRDDYNHDITELMDSDTDLIVPSWIKPVPLKFLPSVFVNKEEGSHMFLDLTRR
IMKTKGIVINTFFELESQAINSLNTLPVYPVGPILNVAGDNNSASAGAIMKWLDSQPPSSVVFLCFGSMG
CFDVDQVKEIACALEHSGHRFLWSLRRPPSNETSELPEEYTNLEEVLPEGFLERTTKIGKVIGWAPQTAI
LSHRAVGGFVSHCGWNSTLESVWYGVPLATWPLFAEQQVNAFMMVEELEMAVEIKMDYHKSFYLKNGIVL
KSEEIEKGIRRLMESGDIEIRQKVEQMRDKSRVAVKEDGSSFRSIERLIEDVINGAKMK
>GWHPAAAL020411
MVIPDFLIVEDNNSSEALSWVDSCTGDGYGGQMHHESSESNWEWSQSLPSKTLKGIHGEEDGVDKHESSK

QSNNTILIKSFKEIEGKYYDYHSLLAKKKFVPVGPVVQEPVCEEGFSKINEWLNKKDYGSTVLVSFGSEY
FLKKEDNIEIAHGLELSNVNFIWVLRFPKGEKLVAQETFPLGFFYRVGDRGMVVEGWAPQVRILGHPSTG
GFASHCGWGSVIESMSFGIPIIAMPMHLDQTINARVVEEVGIGAEATMYISGKLKRENCGGDQGGGGGDR
RGRREEKGKRDEG
>GWHPAAAL021381
MTIPHVLAIPYPAQGHVMPFMELSQCLVQHGVRVTFVNTDFNHKRVTKSMSEKESLTGMMHLVSIPDGLE
PWEDRNDVGKLFESIFQVMPSKLEQLIEKINASEGKKISCVISDQCLGWALEVAEKMGIKRVTFWPASTA
TMALTMNIPKLIDDGVINENGTSCSFSSWNRYEITHLYHFFFILKFHTARTPISKQIIQLLPTMPAMNTA
NFYWACFGDLSTEKSIFQMMVKNNKFLKLADRVICNSIHELEPAAFTLCPEVFPIGPLPLINKVGRSGAN
FWPEDPTCLKWLDQQPPQSVIYLAFGSFTIFDQTQFRELALGLELTNRPFLWVVRPDITDQTKDPYPEGF
IQRVSDRAQIVGWAPQQSILGHPSVACFMSHCGWNSTIEGVSNGVPFLCWPYFADQFWNQQYICDVWKVG
LGFARDESGIIVQDEIRNKVEHLLGDESFRARATVLKEKAMETVKEGGSSHRNFSDLIGWMKEIKHD
>GWHPAAAL021402
MSDVAGRPPHIALFPGAGMGHLTPFLRLAAMLASHNCKVTLIAVHPTVSAAESSQISAFFAANHHINRLD
FHILPHNPPDSEVNDPFFVQFEAISRSVHLLGPLLSAASPPISAVFSDFPVAASMAQITDDLTIPNYIVS
TTSARFFSLIAYFPLLNLQNHNKSQIEIPGISPLPFSTLPPPLFSPNNLFTTGLATNSKYLPKAKGILVN
SFHGFEPETIEAFNNGKIQENLPPLLPIGPLLQPHEHIPQCHEISWLDKQAEESVVYVSFGSRTAMAKEQ
VRELGDGLERSGCPFLWVLKTKILDKDESEEVEEFLSNSFLERTRRKGVVLKEWVNQERILSHPAIGGFV
SHCGWNSVMEAAKEGVPILAWPIGEKIREVMEDEKLRAKARKVGEEARKAVGMVGGSSEMVLQGILQSIA
ETCH
>GWHPAAAL021780
MASKPLVHVFLVSFPGQGHVNPLLRLSNLLASGGFFVTFSTTVSFGKQMQSVNPAVTGEPTPVGDGFIRF
EFFDEELALDDPRRRDPNLFFYHLELAGKENLRRLVENQSSEDGRTPVSCLINNPFLPWVADVADSLGIP
CAILWVESCASLSAYYHYYHGLVPFPDGEQPELDVEIPFLPLLKHHEIPSYLHPNDPCTFLKKLVLGQIK
RLPKIFCVLIDTFEELEPQIIRHVSDLIPIRSVGPLLRYRKSSSWITGDRLKTDDCIEWLDSKPPSSVLV
MSFGSVAQVKQEQIDEIAHGLLSSCVSFLWVMTPPMAGSPDKPRILPDGFSEKMGDRGKVVEWSPQERVL
AHPSVACLVTHCGWNTTVEVLSNGKPVLAFPQWGDQVNNAKFLVDVFGVGIRVYRGEDRGSIVSRDEFEK
CVREAMSGPKAVDIKENAMRWKKVAEEAVAEGGSSRRNMLDFMEEIKRRSVD
>GWHPAAAL021819
MVPISTEPIRKCHVVAVPYPGRGHVNPMMNICKLIASKFDPDRLVITFVVTEEWLGLIGSEIKPANVRFA
AIPNVVPSERVRAADMVGFVTAVMTKMEQPFEEVLDRMQSPASVIIADTFLRWAVEVGNRRSIPVASLWT
MPAMVFSVFYNFELVVENGQFPIELCERGEERVDYLPGIPSIRLSDLPTIIHRRDQNLLRLCLTAFANVS
RARYLLFTTCSDLEPQVIAALKTQFPFPLYCLGPTIPYFNLQQPPPITPNHHDYLKWLDSKPPNSVLYVS
LGSFLSVSSSQMDEIEAGLGGSGVNYLWVNRGHAGGVGGGGRMVVRWCDQLRVLCHPSVGGFWTHCGWNS
TMEGIYAGKPMLTLPILMDQVPNSKCVVEDWKIGWEVKREEGNLVSREKIVEVVRRFMDEGSEEREEMMR
RAEELRRLCRTALVRGGSSHENLQAFVDGIAKF
>GWHPAAAL022080
MTFLCSSFLVAYVWPSPETPGSQKPHVLVVPFLAQGHFSPALQLSKRLASKGLRVTFVTTISCPKSNGSI
RMEQISDGYEQREKPESIEAFAEGIKTAISQGLHDLMEKQKQVGYPVKLVVYDSGMPWALEMVHKSGVYG
VLFFTQPCVVCALYYHMQQGTLEVPFEGTKVTLPSLPPLEKNDLSSFISNLFPHEFLVKGLLDQFSNIHR
ANWILFNTFDKLEKEVVKWMASQWPIRTVGPTIPPMYLDKQLKRDKEYGLSLFTPNTEASMKWLDTKEQG
LVVYVSFGSMAILDETQMEELSWGLVQSNNYFLWVIRASKESKLSSKFMAKTLEKGLMVNWCPQLEVLAH
QAVGCFVAHCGWNSTPEALCLGVPMVAMPQWVDQTTNAKFVADVWGGRGSRGGQ
>GWHPAAAL022859

MEMEMEKREYRAHVLAIPFPAPGHVNPLLQFCKRLEFKGLKASLAITKFQSKSMQSRLGSIQIHTISDGY
DEIGRHNAESVGVWISTFKEVGSKSVANLIQKFRSLGHPIDCIVYDSFLPWVLDVAKENGVAGASFMTHK
CAVNHINYHVYHKKIPYPFSSPTYSIPGLPSLDLEYMPGFMHVHPDYYDLVLSQFSTVDRADYVFVNTFY
KLEAEVLDEMSKTLPVKPIGPTVPSFYLDNRVEDDKNYGLNLQQLDSSVCLNWLSSKPARSVVYVSFGSV
ATASPAANEMEEIAWGLKNSNCYFLWVVNANEEPNLPQNFKEEMSEKGLIVAWSPQLEVLSHHSVGCFLT
HCGWNSTIEAICLGVPMVAVPKWADQTTNSKFIQDVWKVGVKVMPSENGIARREEIERCINV
>GWHPAAAL023009
MIPTLDMAKLFSSRGVKTTIITTPLNAPVFSKSIQRSNQLGLEISLKIFDFPAVAAGLPEGCESADQITS
EDMIPKFFVATALLREPLEQLLSDHRPDCLVADMFFPWTTESAAKFGIPRLLFHGISFFALCAGESLRTH
KPFRQVTSDSEPFVLPELPHEIKLTRTEVAPFDLYDTQTAISGVLKQVRESEKTCYGIIANTFYALEPDY
ADHYRNAMGRTAWHVGPLSLCNRGIEDKAERGKKASIDGHECLQWLDSKKPKSVLYVCFGSMAKFADSQL
YEIAAGLESSGQQFIWVVRKSKDEEEQKEETWLPHGFEERTQGKGLIVRGWAPQMLILDHEAIGGFVTHC
GWNSTLEGVCAGLPMVTWPAFAEQFYNEKLVTDVLKIGVSVGAKEWNRGPREGVKREAIEKAVRSVMEGE
EVEEMRRRAAALKEAAMEAVEEGGSSYSALTSLIEELSSRQKINGK
>GWHPAAAL023788
MERKTSILMFPWIAYGHISPFLELAKKLSQRDFFIYLCSTPANLASVKPKIDEKYSLLIKLVEFHLPTLP
DLPPCFHTSNGLPPHLMHTLMTAVDMSNPSFSDILETLKPDLLIYDFVPHWAPATAAAHNIPAVEFITSS
ATVYFRHYEKAHQDEMIESDRYQYNKNLSVKSPQQSNNIILIKSFKEIEGKYYDYLSLLAKKKIVPVGPV
VQKPFCEEGFSEIIEWLNQKDYGSTVFVSFGSECYLKKEDIIEIAHGLELSNVNFIWVLRFPKGEKLGAQ
ETLPLGFLDRVRERGMVVEGWAPQARILAHPSTGGFASHCGWGSVIESMSFGVPIIAMPMQYDQPINATV
VEEVGLGVEATRDITGKLQREKIAAVLRKVMVEREGEGVRKKAREMRDDIRIKGDEEIDNVVLELQQLCK
IK
>GWHPAAAL023789
MERKTSILMFPWIAYGHISPFLELAKKLSQRDFFIYLCSTPANLASVKPKIDEKYSLLIKLVEFHLPTLP
DLPPCFHTSNGLPPHLMHTLMTAVDMSNPSFSDILETLKPDLLIYDFVPHWAPATAAAHNIPAVEFITSS
ATDYFRHYENAHQDEMIESDRYQYNKNVSVKSPQQSNNIILIKSFKEIEGKYYDYLSLLAKKKIVPVGPV
VQKPFCEEGFSEIIEWLNQKDYGSTVFVSFGSECYLKKEDIIEIAHGLELSNVNFIWVLRFPKGEKLGAQ
ETLPLGFLDRVRERGMVVEGWAPQARILAHPSTGGFASHCGWGSVIESMSFGVPIIAMPMQYDQPINATV
VEEVGLGVEATRDITGKLQREKIAAVLRKVMVEREGEGVRKKAREMRDDIRIKGDEEIDNVVLELQQLCK
IK
>GWHPAAAL023791
MESKTSILMFPWIAHGHISPFLELAKKLSKRNFLIYLCSTPANLVSVKPKIDEKFSLSIKLVELHLPALP
DLPPCFHTTNGLPPHLMFTLKDAFDMSNQNFSDILETLKPDLLIYDFLQPWAPATAAAHNIPAVEFITSS
ATMTSLMLHVFNDPDKDFPFSDTVYFRDNERAHLDRMIESARNQHKKKNPAIECLKQSNNIVLIKSFKEI
EGKYCDYLSLLAKKKLVPVGPVVQEPVCEEQFSDIIEWLNKKDHGSTVFVSFGSEYFLKKEDITEIAHGL
ELSNVNFIWVLRFPKGEKLAAQETLPLGFLDRVGERGMVVEGWAPQARILGHPSTGGFASHCGWGSVIES
MSFGIPIIAMPMHLDQPINARVVEEVGIGVEATRDITGKLKREKIAAVIREVVVETEGEGVRKKARKMRD
DIRIKGEEEIDNVVLELLQLCKKK
>GWHPAAAL023813
MDTTRERSSERMRILMFPWIAHGHISPYLELAKRLNQRNIKTYLCSTPINLNSVKNKIDNNSDSLLQLVE
LHLPSTPELPPQYHTANGLPRHLMIPLKATIEASGPPKFSAMLETLKPDLVLYDFNLPWGATVAASHNIP
AVQFIIVGAVMISFALHTLEKPATEKYPFPAITLSESQSHEMRKLLDESVLGISDKDRFREGVDRSQDII
LIKTSTEIESNYLNYLSELAGNKKIVPVGQLVQQADDRRRDFNDEDQIIKWLDKKEKANSVVFVSFGSEC
FLSKEEIQEMGRGLELSRVDFIWVVRYPVGDKTRVLEDELPEGFRERVLGGERGMVVEGWAPQSKILGHP

NLGGFLSHCGWSSVMESMKMGVPIIGMPMGVDQPLNCKVVEDAGVGVEVKRDSDGKIKGEVIGEAIRRVV
AEMGGESVRKKAKELGEKLRETGEREVEAAVQEILKFSSNKQ
>GWHPAAAL023819
MGMAAKNDDESGDNQTVAEYGEKRRWGGCSLPVRRRRWRRGIGGDERESGNSGEFSEWLDGKEELSTVFV
SFGSEYFLSKEEIVEIAHGLELSEVNFIWVIRFPMGEEKKTRVGEALPLGFKERVRERGMVVEGWAPQAK
ILGHKSVAGFVSHYGWNSVLEGMKFGVPLVAMPMHLDQPVNARLAVEVGVAVEALRDEDGGVQRKEVARV
IREVVVGENCGGVRERARELSENMRLRGDVEVHGLVEVLAQLHQKNKAQSC
>GWHPAAAL024115
MAEDRNEGNIVMFPFMAQGHLIPFLALARQIEQRHGGYTITIVNTPLNICKLKCWLPPETTIRLVEIPFS
AADHGLPPDSENTDMLSREQALYLFEASESLEKPFENLISEIATGDGRQPICIVSDMFLGWTVNVAKKLG
IFHSVFIAGDGYGMGLYFSLSLNENLWTVEAADEFLPVDFPGAARIHWSRIPIDLKYEEAAESWFRFRKR
QFSYCLCSDSILLNSIKELGQKGVEYFRRKTGGKPVWTIGPVSNLTVKNKIFTKISTNPISDWLDLHPPG
SVLYISFGSQCSLLASQMKELAMGLEESRCPFIWVVRPPVEFDLTGDFRSEWLPVGFEERMRRLNQGFLI
HGWGPQLEILSHRSTGGFLSHCGWNSVVEGLCGGVVILGWPMAGEQFFNSEMLAEEVGVCVEVREKNNGC
GIVGHQRIAEVIEMAMGKSEKGEEMRRKAREMSEKMEAAVKVEEGFRGSSVKVMDEFISTAISKKKR
>GWHPAAAL024577
MAANHQNGGSLTFTGPVSIVAVPFPAHGHLNQLLHLSCFFSSAGLPVHFASTATHNRQARLRATSLNFEK
IHFHDLPHPHFQSPPPNPTAAIKFPAHLQPSFEAALHLRQPLAALLREISTVSRRVIVIHDPLMSSVAQE
AASILNPETYVFSCLSVLFQLAHLCENKFQAELPANLPTMDGCLTPEFMEFSASQFQLLGTEEGKFRAGY
LYNSCKLIEGRYIDLLSSYDQYQDKKIWAIGPILPVKSVTKTSNWSHKSIEWLDKQEPRSVIYVSFGTMT
SISDEQIRELAVGLEQSKQRFIWVLREADRADIFSGEEQRKVEVPEGFEERVKGIGMVVRDWAPQPEILG
HRSTGGFMTHCGWNSSLESVTMGVPVAAWPMHSDQPRNAVLLTEVLKVGVPVREWTGKGEIVGSSEIEEA
VRRLMASEEGEEIRKRAEELGGEIRRSTAEGGVCRVELDSFVAHITRN
>GWHPAAAL024578
MEAFGFSGRDHSSRHDVVVVMVPFPAQGHLNQLLNLSRLLTSHGIPVYFAGTAIHNRQARLRAHGWDPLT
TPNMHFHDFPTPSFQSPPPNPNDASTSFPTHLLPSFNASLDLRQPFADLLRDFSPEARRVVVIHDSLMGS
VVQDVGSTPNAESYTFHSVSAFTIFWYLWEAKGRPFPVEGEILKDIPPTDGCFASEFPEFIQNQHAYMKF
NSGDLYNTSKVIEETYIDYLCKEPIKGNKKQWPIGPFNPVEPVETKKPNGRHFCLNWLDKQAINSVIFVS
FGTSTSLKDDQIEELANGLDQSEHRFVWVVRDADRGDVFDGMPRRVELPVGFEERVREKGIVVRDWAPQP
EILRHPSTGGFMSHCGWNSCMESITMGVPIAAWPMHSDQPRNAVLIAKLLKIGIIVKDWGRRGERVVAET
VEKAVKSEEGDKIRHGGGLSDAVRESVAEGGSTRLELDSFVAHITRS
>GWHPAAAL025080
MEDAIVLYPSPGIGHLISMVELGKLILNHHRSFSINILITTPPYNTGRTAPYIDRVSAANPSIHFHHLPS
VPLSVSTTSLNHETLAFQLLLLNNPHVHQALTSISQTSTPCAFIIDFFCASALSVAKDLNIPTYFFYTSG
AGALASFLYLPTIHRSTAGNLKDLKTSLHIPGLPPFRASDMMKPVLDRSDKAYEFFLDVSLNFPKASGII
INTFEYLEPRSIKAISSGLCVSNSDGPPPPIYCIGPLIAAGDRSDGKTLNRHECLRWLDSQPSKSVVFLC
FGSLGLFPMDQLREIAIGLERSGCRFLWVVRSPPPPPENQTERFLAPADPDLGALLPDGFMDRTEGRGFV
VKSWAPQVEVLSHDSVGGFVTHCGWNSVLEAVCAGVPMVAWPLYAEQKFNRVVLVEEMKLALPMEESEDG
FVCAGEVEKRVTELMDSVDGRLVRKHVLAARDAAKAAIGDGGSSRLDLGQLIELWTQN
>GWHPAAAL025081
MEDAIVLYPSPAIGHLISMVELGKLILNHQPTFSIIILIATPPYNAGNTAPYINQVSATTPSIHFHHLPS
VPLSLSTASRNHETLAFQLLLLNNPHVHQALTSISQTSTPRAFIMDFFCTPALSVARDLNIPAYFFYTSG
AGGLASFLYFPTIHRSTAGNLKDLKTFLHIPGLPPFRATDMPKPVLDRADKAYEFFLDVSLNFPKASGII
VNTFEYLEPRSIKAISDGLCVSDSDGPTPPIYCIGPLIASGDRSDGEIVNKHECLRWLDLQPSKSVVFLC

FGSLGLFSLEQLREIAVGLERSGCRFLWVVRSPPLPLENQTERFLAPADPDLGALLPDGFMDRTEGRGLV
VKSWAPQMEVLNHDSVGGFVTHCGWNSVLEAMCAGVPMVAWPLYAEQRFNRVVLVEELKLALPMEESEDG
FVCASEVEKRVTELMDSVEGRSVRTHVLAARDAAKAAIGDGGSSRLNLGRLVDSWTRN
>GWHPAAAL025228
MSVHVLIFPYPAQGHLLPLLDLTHQLALHGLTITILVTPKNRPTLDPLLSTHPSIGTLVLPFPHHPAIPS
GVENVKDLGNSGNVLVINALSKLRDPIIQWFKAHPNPPVAIVSDFFLGWTNDLAGQLGIPRVAFYSSGAF
LTSILFHFYRELRSAAPVLTAVKFTDLPRSPSFTEAHLPTIVRHYRESNPEWELLKEGVIANSWSWGSIL
NTFDDLEGEFLEYLKKKMGHNRVYGVGPLNLGRVNTDPDADPSNTVLGWLDGCPDGSVIYVCFGSQKFLK
KAQIEALALGLERSKARFIWVVKPITAQQEADGYGSILDGFEERVSGRGMVIKGWAPQVKILSHRVVYGF
LSHCGWNSVLEAIAAGVMILGWPMEADQFVNARLLVDYMGAAIRVCEGADSVPDSDELARTIAESVDGNK
QEKGRARELRDKAWEAVGPCGSSTKDLVGFVKDLAQLDENGV
>GWHPAAAL025545
MIPTLDMAKLFSSRGVKTTIITTPLNAPVFSKSIQRSNQLGLEISLKIFDFPAVAAGLPEGCESADQITS
EDMIPKFFVATALLREPLEQLLSDHRPDCLVADMFFPWTTESAAKFGIPRLLFHGISFFALCAGESLRTH
KPFRQVTSDSEPFVLPELPHEIKLTRTEVAPFDLYDTQTAISGVLKQVRESEKTCYGIIANTFYALEPDY
ADHYRNAMGRTAWHVGPLSLCNRGIEDKAERGKKASIDGHECLQWLDSKKPKSVLYVCFGSMAKFADSQL
YEIAAGLESSGQQFIWVVRKSKDEEEQKEETWLPHGFEERTQGKGLIVRGWAPQMLILDHEAIGGFVTHC
GWNSTLEGVCAGLPMVTWPAFAEQFYNEKLVTDVLKIGVSVGVKEWKAGPREGVKREAIEKAVRSVMEGE
EAEEMRRRAAALKEAAMEAVEEGGSSYSDLTSLIEELSSRQKINGK
>GWHPAAAL025754
MENQKKGNGGAHILAIPFPAPGHMNPLIQFCKRLVFKGGVKATVAITRYQAKSIQTRLDTIQIDTISDGY
DEGGFYVIDSIEDSIEKFKVAGSQTLAELIEKYEKLGNPIDCIIYDAFLPWVLDVAKKFGKIVAAFFTQP
CAVNCINYHVYHKLLPYPFSSPPISVSGLPPLNLEDMPGFIYVDPGHYVLVQSQFSKADEADYRFVNTFY
DLEAEAVDAMKKIHSLRTIGPTVPSFYLDKRVPNDSNYGLHSSNLIMQQKKSTSTPDADPLACLDWLSTK
PPRSVVYVSFGSVAVASPVSEEMKEIAWGLRNSNCFFLWVVKGNEEPNLPENFKEETADKGMIIPWSPQL
EVLAHEATGCFITHCGWNSTIESLCLGVPMVAMPKWADQTTNAKYIEDVWKAGVRAKVGENGVASREEIE
RCIRAVLEGESGKYYKSNAMKWKDLATHAVSEGGTSDKNIAEFLSELKAR
>GWHPAAAL025999
MEKTTHVAILPTPGMGHLIPLVEFAKRLIDGYNFSFTLILPTDGPLSKAHKEFVDSLPAGIDHVLLPPVS
HDDMTVENRIALTISRSLPAFRDAFKTLVDEKKVAALVIDLFGTDGFDVAIEFGVSPFIFFPSTAMALSL
FLHLSKLDAETVGEYRDQPEPIRIPGCIPIQGKDMLDPVQDRKNDAYKWLLHHAGRYKLAEGIMVNSFRE
VEPGPITALLKKEPGKPPIYPVGPLIKMSSNADDAESECLKWLNGQPSGSVLFVSFGSGGTLSSVQLNEL
AMGLEMSEQRFIWVVRSPNDGTSAATYFNVNSQTDPYSFLPEGFVGRTRGRGLLVPSWAPQAAILSHGST
GGFLTHCGWNSTLESVYNGVPLIAWPLYAEQRMNAVMLTEDLKVALRPKTGENGIVGRLEIANVVKGLME
GEEGKGIRSRMRDLKDAAAKAIGEDGASTKALSEVACIWKNKINYCFN
>GWHPAAAL026143
MDHHHFLLISLPGQSHLNPTIQLGKVLARAGVTVTFATTVYGLNQLKTKSTTVDGIAYTSFSDGHDEDGH
KARSNFAGYMDDLKRVGSQNLIKLLENLAGEGRPVTFIVYTVLLPWVAQVAREMHLPSAFFAIQCAAALA
VYHRFFNSSDGLHGGENEINDSISVELPGLPQLSSGEIPSFLLPADPQHSSINQPCREHILTLEEDPNPT
VLINTFDALEEDSIRAVRNMKIIAVGPLVPSAFSDGIDASDKSFGCDLFRNSGDQYLPWLDSKSDSSVIY
VSFGSLVVLEKPQKEEILNGLIDTGRPFLWVIRDTAGEEEETAVPENGLVVPWCTQVEVLAHSSIGCFVT
HCGWNSTMESLVAGVPVVAMPQFSDQLTNAKLVEEVWGNGVRAGKSEEKGVVEREEIRRCVEVVVGGGER
GEEIRGSARKWRGLAMAAVREGGSSSKNLREFLDSLG
>GWHPAAAL026144

MDRRRHRHFLLISLPAQSHLNPTIQLGKVLARAGVTVTFATTVYGLNQLKTKSTTVDGIAYTSFSDGHDE
DGHKARSNFAGYMDDLKRVGSQNLIKLLENLAGEGRPVTFIVYTVLLPWVAQVAREMHLPSAFFAIQCAT
ALAVYHRFFNSSDGLLGGENEINESLSVELPGLPPLSSGEIPSFLMPANQYHSSVSPSFREHILTLEEDP
NPTVLINTFDALEEDSIRAVRNMKIIAVGPLVPSAFSDGIDASDKSFGCDLFRNSDDHHLRWLDSKSESS
VIYVSFGSLAILGKPQKEEILNGLIDTGRPFLWVIRDTAGEEEETAVPENGLVVPWCTQVEVLAHSSTGC
FVTHCGWNSTMESLVAGVPVVAMPQFADQLTSAKLVEEVWGNGVRARKSEEKGVVEREEIRRCVEVVVGE
GVRGEDIRRNALKWRGLAMAAVREGGPSCKNLRDFLDSLG
>GWHPAAAL026243
MEKLTETRRYPKEVVLVPYQAQGHMSPMLQLGTLLHSLGFSITVAHARINSPDPLTHPDFDFRPISEDLS
DRDTSFHGLLSLITTLNTGCKELLRECLVRLKEERGPVACVVYDSVMYFADEVVNQMKIPSMVLQTCNAA
YVLPCQLLPQLKAEGYLPSESEDPAGDHLVPKLYPLRFKDLPILKFNTPTLQLLTTLSDIRTSSAVIWNT
IDHLEHPSLCRLRQHYKVPIFPLGPLQKMAPSSSASFLKEDYDCLTWLEKQAPKSVIYVSFGSIAMVEPN
ELSEVAWGLANSGQPFLWVVRPGLVEGSERIEHLPEGFKETVGERGCIVKWAPQKEVLAHEAVGGFWSHC
GWNSSLESISEGVPMICRPCFGDQRVNARYLTHVWRVGLELENKLERGAIESAVRRLMIGKEGKEIRRKV
LDMKQNIEEIVQKGGSSYNTLIELADFISDFHKTGQY
>GWHPAAAL026244
MENQKETRYPKEVVLVPFPVQGHTSPMLQLGTLLHSLGFSITVAHARINSPHPSTHPEFDFQPISEDLAD
RDTSFNGLFSVIMTLNTDCGEPLRECLVRLTEDRGPVACVVYDSLMYFADEVANQMKIPSMVLQTCNAAY
ILPCQLLPQLKAEGYLPSESEDPSWEDLVPKLHPLRFKDLPFPKFDTPTLQVLMTLCDVRTSSAIIWNTT
DHLEHPFLCRLRQHYKVPIFPLGPLQKMAPSSSTSFLKEEYDCLTWLEKQAPKSVIYVSFGSIATVEPNE
LSEVAWGLVNSGLPFLLVVRPGLVNGSEWIEHLPESFKEFVGERGCIVKWAPQKEVLAHEAVGGFWSHCG
WNSSLESISEGVPMICRPCFGDQRVNARYLTHVWRVGLELEDKLERGAIERVLRRLMTGKEGEEIRRRAM
DMKQNIEEVVQKGGSSYNSLIELAGFISDFHTTKQCV
