# Supplementary figures and images for: Genomic-Wide Identification and Characterization of the Uridine Diphosphate Glycosyltransferase Family in Eucommia ulmoides Oliver
Source: Plants (Basel). 2021 Sep 17;10(9):1934. doi: 10.3390/plants10091934 (PMC8471388; doi:10.3390/plants10091934)

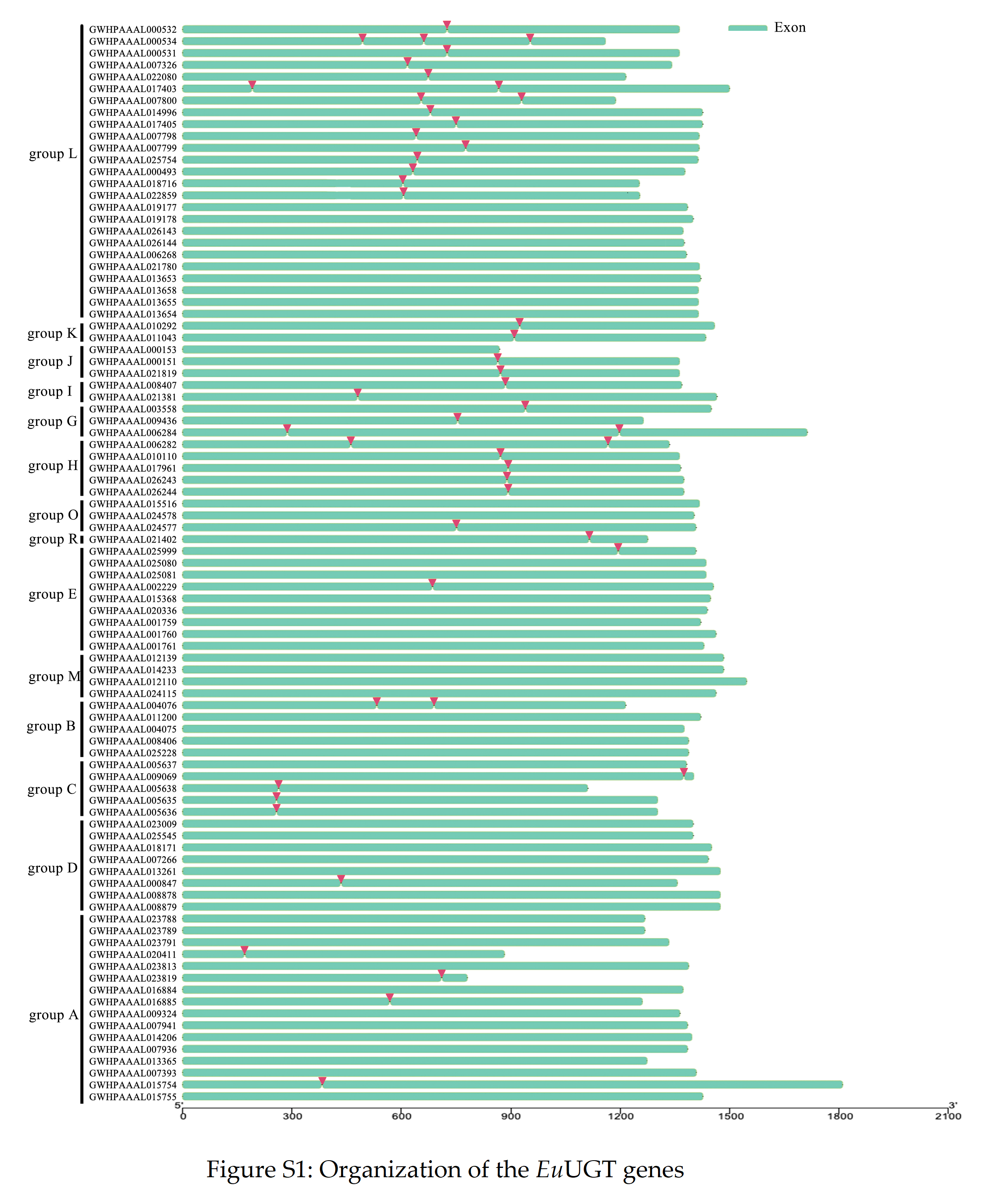

Supplement: Supplementary file 1 [file plants-10-01934-s001.zip › plants-1355377-supplementary/Figure S1_R.png]
